# Distinct transcriptional MYCN/c-MYC activities are associated with spontaneous regression or malignant progression in neuroblastomas

**DOI:** 10.1186/gb-2008-9-10-r150

**Published:** 2008-10-13

**Authors:** Frank Westermann, Daniel Muth, Axel Benner, Tobias Bauer, Kai-Oliver Henrich, André Oberthuer, Benedikt Brors, Tim Beissbarth, Jo Vandesompele, Filip Pattyn, Barbara Hero, Rainer König, Matthias Fischer, Manfred Schwab

**Affiliations:** 1 Department of Tumor Genetics, German Cancer Research Center, Im Neuenheimer Feld 280, Heidelberg, 69120, Germany; 2 Department of Biostatistics, German Cancer Research Center, Im Neuenheimer Feld 280, Heidelberg, 69120, Germany; 3 Theoretical Bioinformatics, German Cancer Research Center, Im Neuenheimer Feld 280, Heidelberg, 69120, Germany; 4 Department of Pediatric Oncology, University Children's Hospital of Cologne, Kerpener Strasse 62, Cologne, 50924, Germany; 5 Division of Molecular Genome Analysis, German Cancer Research Center, Im Neuenheimer Feld 580, Heidelberg, 69120, Germany; 6 Center for Medical Genetics, Ghent University Hospital, De Pintelaan 185, Ghent, 9000, Belgium

## Abstract

**Background:**

Amplified *MYCN *oncogene resulting in deregulated MYCN transcriptional activity is observed in 20% of neuroblastomas and identifies a highly aggressive subtype. In *MYCN *single-copy neuroblastomas, elevated *MYCN *mRNA and protein levels are paradoxically associated with a more favorable clinical phenotype, including disseminated tumors that subsequently regress spontaneously (stage 4s-non-amplified). In this study, we asked whether distinct transcriptional MYCN or c-MYC activities are associated with specific neuroblastoma phenotypes.

**Results:**

We defined a core set of direct MYCN/c-MYC target genes by applying gene expression profiling and chromatin immunoprecipitation (ChIP, ChIP-chip) in neuroblastoma cells that allow conditional regulation of *MYCN *and c-*MYC*. Their transcript levels were analyzed in 251 primary neuroblastomas. Compared to localized-non-amplified neuroblastomas, MYCN/c-MYC target gene expression gradually increases from stage 4s-non-amplified through stage 4-non-amplified to *MYCN *amplified tumors. This was associated with MYCN activation in stage 4s-non-amplified and predominantly c-MYC activation in stage 4-non-amplified tumors. A defined set of MYCN/c-MYC target genes was induced in stage 4-non-amplified but not in stage 4s-non-amplified neuroblastomas. In line with this, high expression of a subset of MYCN/c-MYC target genes identifies a patient subtype with poor overall survival independent of the established risk markers amplified *MYCN*, disease stage, and age at diagnosis.

**Conclusions:**

High MYCN/c-MYC target gene expression is a hallmark of malignant neuroblastoma progression, which is predominantly driven by c-MYC in stage 4-non-amplified tumors. In contrast, moderate MYCN function gain in stage 4s-non-amplified tumors induces only a restricted set of target genes that is still compatible with spontaneous regression.

## Abstract 2

Differences in MYCN/c-MYC target gene expression are associated with distinct neuroblastoma subtypes and clinical outcome.

## Background

Neuroblastoma is the most common extracranial malignant solid tumor in early childhood. Clinical courses are highly variable, ranging from spontaneous regression to therapy-resistant progression. Clinical and biological features, such as age at diagnosis, disease stage, numerical (ploidy) and structural chromosomal alterations (*MYCN *gene amplification; 1p, 3p, 11q deletions; 17q gain), are associated with patient outcome [1,2]. Amplified *MYCN *oncogene identifies a subtype with poor prognosis [3] and is consistently associated with high *MYCN *mRNA and protein levels. There is strong experimental evidence (ectopic *MYCN *expression in cell lines, N-*myc *transgenic neuroblastoma mouse model) that increased MYCN activity is involved in tumor initiation and progression of at least a subset of neuroblastomas [4,5].

The *MYC *gene family members, c-*MYC*, *MYCN *and *MYCL*, are involved in the biology of many cancer types. They encode basic helix-loop-helix leucine zipper proteins that are found as heterodimers with their obligate partner protein, MAX [6]. The MYC-MAX heterodimer binds to DNA consensus core binding sites, 5'-CACGTG-3' or variants thereof (E-boxes), which preferentially leads to transcriptional activation of target genes. Repression of target genes by MYC proteins has also been described [7]. This seems to be independent of the binding of MYC proteins to E-boxes, but involves a cofactor, Miz-1, that tethers MYC-MAX to gene promoters, such as *p15 *and *p21*. Enhanced activity of MYC transcription factors contributes to almost every aspect of tumor formation: unrestricted cell proliferation, inhibition of differentiation, cell growth, angiogenesis, reduced cell adhesion, metastasis, and genomic instability [6,8]. In contrast, MYC transcription factors, including MYCN, also sensitize cells for apoptosis, a function that should inhibit tumor formation and that could also be involved in spontaneous tumor regression [9].

Spontaneous tumor regression does occur in neuroblastoma, at a higher frequency than in any other cancer type. This process resembles the physiological concurrence of massive cellular suicide (apoptosis) and differentiation of a few neurons along the sympathoadrenal cell lineage in the normal development of the sympathetic nervous system. Spontaneous regression is most frequently observed in a subset of disseminated *MYCN *single-copy neuroblastomas (non-amplified (NA)), termed stage 4s (stage 4s-NA) [10]. However, population-based screening studies for neuroblastomas in Japan, Quebec and Germany suggest that spontaneous regression also occurs in other neuroblastoma subtypes, predominantly localized (stages 1, 2, 3) neuroblastomas (localized-NA) [11-13]. Paradoxically, *MYCN *mRNA and protein levels are higher in favorable localized-NA and, particularly, in stage 4s-NA tumors than in stage 4-NA tumors with poor outcome [14-16], but they do not reach the levels observed in *MYCN *amplified tumors. In line with this, neuroblastoma cells with elevated *MYCN *expression retain their capacity to undergo apoptosis [17] or neuronal differentiation [18]. Thus, it has been speculated that MYCN does not only mediate malignant progression in *MYCN *amplified tumors, but is also either involved or at least compatible with spontaneous regression in favorable neuroblastomas. In contrast, a functional role of MYCN in stage 4-NA tumors with low *MYCN *levels is questionable. Here, other transcription factors or pathways within or outside the MYC family of transcription factors could be more relevant. Neuroblastoma-derived cell lines that lack amplified *MYCN *generally express c-MYC rather than MYCN, often at higher levels than normal tissues [19,20]. However, transcriptional activity of MYCN or c-MYC as reflected by the transcript levels of direct MYCN/c-MYC target genes in relation to *MYCN *and c-*MYC *levels has not yet been defined in neuroblastoma subtypes.

Here, we defined a core set of MYCN and c-MYC target genes by using oligonucleotide microarrays and a neuroblastoma cell line that allows conditional expression of *MYCN *or c-*MYC*. Direct regulation of these target genes by MYCN/c-MYC was assessed by analyzing the binding of MYCN and c-MYC protein to target gene promoters using PCR- and array-based chromatin immunoprecipitation (ChIP and ChIP-chip, respectively) in different neuroblastoma cell lines. We further investigated the expression of these direct MYCN/c-MYC target genes in relation to *MYCN *and c-*MYC *expression in different clinical neuroblastoma subtypes. In addition, the association of MYCN/c-MYC target gene expression with overall survival independent of the well-established markers - amplified *MYCN*, disease stage and age at diagnosis - was demonstrated.

## Results

### Inverse correlation of *MYCN *and c-*MYC *expression in neuroblastoma subtypes

**Figure 1:**
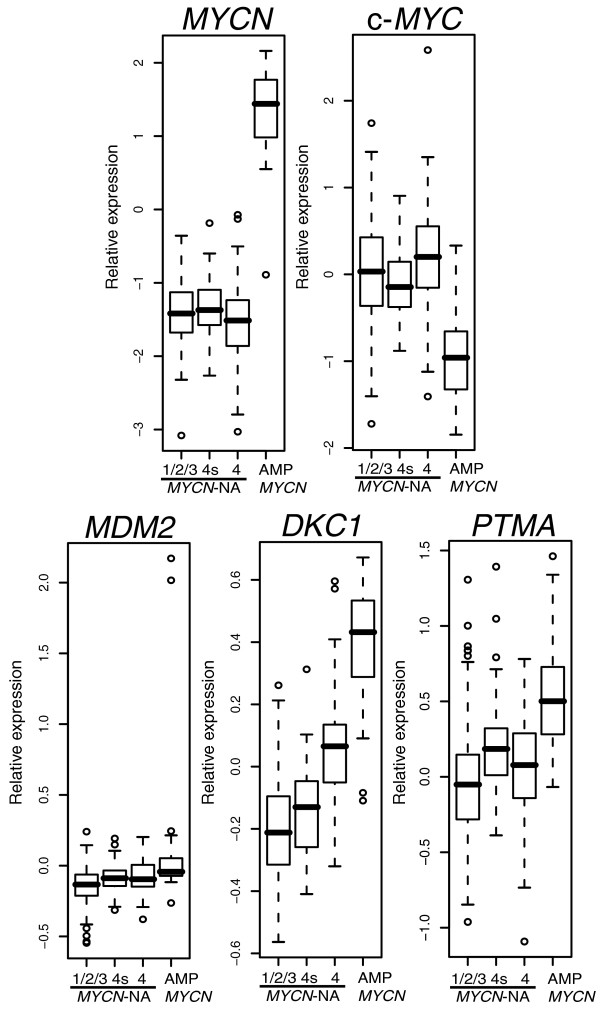
Inverse correlation of *MYCN *and c-*MYC *mRNA levels in neuroblastoma subtypes. Relative mRNA expression is shown for *MYCN *and c-*MYC *as well as for *MDM2*, *DKC1*, and *PTMA*, three direct targets of MYCN/c-MYC. Data are represented as box plots: horizontal boundaries of boxes represent the 25th and 75th percentile. The 50th percentile (median) is denoted by a horizontal line in the box and whiskers above and below extend to the most extreme data point, which is no more than 1.5 times the interquartile range from the box. A set of 251 primary neuroblastoma tumors was analyzed consisting of 138 localized-NA (stage 1/2/3), 30 stage 4s-NA, 52 stage 4-NA and 31 *MYCN *amplified (AMP) neuroblastoma tumors. Gene expression levels from stage 4s-NA, stage 4-NA, and *MYCN *amplified tumors were compared pair-wise with those of localized-NA tumors as reference. Differential gene expression was assessed for each gene by using the Mann-Whitney test (cut-off of *p *< 0.05).

c-*MYC *mRNA levels are very low in *MYCN *amplified tumors (Figure 1), which is due to high MYCN protein repressing c-*MYC *mRNA expression [20]. Previous quantitative PCR analyses in a cohort of 117 neuroblastoma patients revealed that mRNA levels of *MYCN *are significantly lower in stage 4-NA than in stage 4s-NA (*p *= 0.008) and localized-NA neuroblastomas (stages 1, 2, 3; *p *= 0.03) [14]. To test whether this lower expression of *MYCN *in stage 4-NA tumors is due to elevated c-MYC activity that represses *MYCN *expression, we analyzed c-*MYC *and *MYCN *mRNA levels in a cohort of 251 primary neuroblastoma tumors using a customized 11K oligonucleotide microarray (other *MYC *gene family members were not differently expressed (data not shown)). Although c-*MYC *mRNA levels were not significantly higher in stage 4-NA (n = 52) than in localized-NA tumors (n = 138), we found an inverse correlation of *MYCN *and c-*MYC *expression between stage 4s-NA (n = 30) and stage 4-NA tumors. Stage 4-NA tumors showed lower expression of *MYCN *and higher expression of c-*MYC*, whereas stage 4s-NA tumors showed lower expression of c-*MYC *and higher expression of *MYCN *(Figure 1; *p *= 0.008 for c-*MYC*, *p *= 0.07 for *MYCN*).

Because increased activity of MYCN in stage 4s-NA or c-MYC in stage 4-NA tumors should both result in high expression of shared target genes compared to localized-NA neuroblastomas, we analyzed known direct MYCN/c-MYC target genes, namely *MDM2 *[21], *DKC1 *[22], and *PTMA *[23], in neuroblastoma subtypes. As expected, the highest expression of all three transcripts was observed in *MYCN *amplified tumors (Figure 1; *p *< 0.001 for all three transcripts, n = 31). *MDM2 *mRNA levels were higher in stage 4-NA (*p *= 0.005) and stage 4s-NA (*p *= 0.03) than in localized-NA tumors (the expression range of *MDM2 *is large because of two *MYCN *amplified tumors with non-syntenic co-amplification of *MDM2 *(data not shown)). Similarly, *DKC1 *and *PTMA *expression was higher in stage 4-NA (*p *< 0.001 for *DKC1*, *p *= 0.02 for *PTMA*) and in stage 4s-NA (*p *= 0.03 for *DKC1*, *p *= 0.007 for *PTMA*) than in localized-NA tumors. These results suggest an increased MYCN/c-MYC activity also in stage 4s-NA (MYCN) and in stage 4-NA (predominantly c-MYC) compared to localized-NA tumors. However, higher *DKC1 *mRNA levels in stage 4-NA tumors and higher *PTMA *mRNA levels in stage 4s-NA tumors also suggest differential regulation of MYCN/c-MYC target genes in these subtypes. To further analyze MYCN/c-MYC activity as well as differential regulation of MYCN/c-MYC target genes in neuroblastoma subtypes, we thought to define a comprehensive set of target genes directly regulated by MYCN and/or c-MYC in neuroblastoma cells.

### Repression of endogenous c-*MYC *by targeted expression of a *MYCN *transgene in SH-EP^*MYCN *^cells defines c-MYC- and MYCN-regulated genes

**Figure 2:**
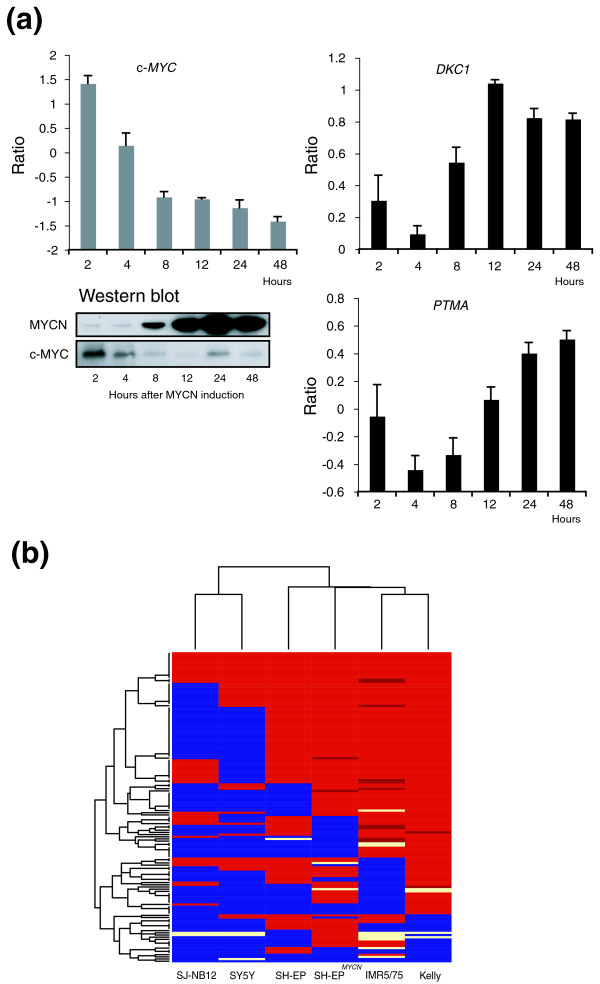
Identification and validation of MYCN/c-MYC target genes in neuroblastoma cell lines. **(a) **Repression of endogenous c-MYC by targeted expression of a *MYCN *transgene in SH-EP^*MYCN *^cells defines MYCN/c-MYC-regulated genes. MYCN and c-MYC protein levels were monitored in a time series after removing tetracycline in exponentially growing SH-EP^*MYCN *^cells that stably express a tetracycline-regulated *MYCN *transgene. Mean and standard deviation of the relative mRNA levels of *MYC*, *DKC1 *and *PTMA *are given from two time series experiments as measured by a customized neuroblastoma oligo microarray. **(b) **Hierarchical clustering of MYCN- and c-MYC binding to 140 target gene promoters as measured by ChIP-chip in 6 neuroblastoma cell lines. ChIP-chip results of 140 MYCN/c-MYC target genes from 5 neuroblastoma cell lines that preferentially express either high levels of MYCN (SH-EP^*MYCN*^, IMR5/75 (approximately 75 copies of *MYCN*) and Kelly (approximately 100-120 copies of *MYCN*)) or c-MYC (SJ-NB12 and SY5Y). Additionally, as an intermediate type, parental SH-EP cells were analyzed. SH-EP cells preferentially express c-MYC, but also low levels of MYCN. ChIP-chip experiments were performed with a monoclonal antibody against human MYCN and a polyclonal antibody against human c-MYC for each neuroblastoma cell line. A cut-off for positive binding was set for both transcription factors to >4-fold enrichment for one and >2-fold enrichment of at least one of the two neighboring probes. MYCN/c-MYC-binding is color-coded as follows: blue, c-MYC binding; red, MYCN/c-MYC binding; dark red, MYCN binding; light yellow, lack of MYCN/c-MYC binding. Hierarchical clustering was used to group neuroblastoma cell lines according to their MYCN/c-MYC-binding pattern. Differentiation between MYCN and c-MYC-binding was mainly achieved through the monoclonal MYCN antibody. The polyclonal antibody against c-MYC also gave positive binding signals for a large set of analyzed target gene promoters in neuroblastoma cell lines with high MYCN that lack c-MYC expression (SH-EP^*MYCN*^, IMR5/75 and Kelly).

To identify MYCN/c-MYC-regulated genes in neuroblastoma cells, we employed the experimental system SH-EP^*MYCN*^, which stably expresses a tetracycline-regulated *MYCN *transgene [23]. Exponentially growing SH-EP^*MYCN *^cells cultured with tetracycline express c-MYC but almost no MYCN protein (Figure 2a). Induction of MYCN by removing tetracycline from the medium is associated with a rapid reduction of c-MYC at the mRNA and protein levels. c-MYC reduction occurs prior to the full expression of ectopically induced MYCN protein (Figure 2a). Accordingly, mRNA levels of direct MYCN/c-MYC targets, such as *PTMA *and *DKC1*, initially decline before accumulating MYCN protein leads to the re-induction of these genes. Similar profiles were observed with direct MYCN target genes, such as *MDM2 *and *MCM7 *(Additional data file 1).

We used SH-EP^*MYCN *^cells for a global search of MYCN and c-MYC target genes in neuroblastoma cells using a customized neuroblastoma oligonucleotide microarray (11K, Agilent) that was enriched with probes for genes differentially expressed in neuroblastoma subtypes and for direct MYCN/c-MYC target genes [14,24]. Gene expression profiles of SH-EP^*MYCN *^cells at 2, 4, 8, 12, 24, and 48 hours after targeted *MYCN *expression were generated. Self-organizing maps (SOMs) were used to capture the predominant pattern of gene expression. This analysis yielded 504 clusters (best matching units (BMUs)) consisting, on average, of 20 clones per cluster (Additional data file 1). We searched for clusters with characteristic gene expression profiles of direct MYCN/c-MYC target genes. In addition, known c-MYC target genes from a public database [25] and known MYCN target genes from a literature search were mapped to the 504 clusters (Additional data file 2). A significant enrichment of known MYCN/c-MYC targets was found in 6 clusters (clusters 140, 168, 195, 280, 308, and 336; *p *< 0.05, adjusted for multiple testing), consisting of 167 genes. The genes in these six clusters were induced by MYCN and c-MYC in SH-EP^*MYCN *^cells. Based on their average gene expression profiles, we grouped the clusters into two subgroups, I and II. Subgroup I genes (clusters 140, 168, and 195) were expressed at equal levels in SH-EP^*MYCN *^cells expressing endogenous c-MYC (2 hours) and in those fully expressing ectopic MYCN (24 and 48 hours), despite the fact that the maximum protein level of MYCN was significantly higher than that of endogenous c-MYC (Figure 2a; Additional data file 1). This indicates that subgroup I genes are regulated by MYCN, and also suggests that they are less responsive to MYCN than to c-MYC in SH-EP^*MYCN *^cells. The mRNA levels of subgroup II genes (clusters 280, 308, and 336) were highest in SH-EP^*MYCN *^cells fully expressing ectopic MYCN and followed the combined absolute c-MYC and MYCN protein levels during the time course experiment. We also found clusters with MYCN and c-MYC repressed genes (for example, subgroup III; Additional data file 1). However, enrichment of known MYCN/c-MYC repressed genes from the literature/database in defined clusters was not found using our statistical cut-off (after adjustment for multiple testing, no cluster showed *p *< 0.05). This was at least partly due to the fact that in SH-EP^*MYCN *^cells, some genes were repressed by MYCN but not by c-MYC (subgroup IV). In addition, c-MYC repressed genes from different experimental systems compiled in the c-MYC target gene database were not necessarily repressed by MYCN and/or c-MYC in SH-EP^*MYCN *^cells.

Therefore, we focused on genes for further validation that were induced by both MYCN and c-MYC proteins in SH-EP^*MYCN *^cells and grouped into subgroup I and II. We extracted all available promoters from the genes represented on the array and scanned for canonical E-boxes (CACGTG) and for the 12 bp MYCN position-weight matrix [26] within -2 kb and +2 kb of the transcriptional start site. We ranked all 504 clusters according to the relative number of putative MYCN/c-MYC binding sites in each cluster. All clusters from subgroups I and II were among the 15 top-ranked clusters with enrichment of predicted MYCN/c-MYC binding sites (data not shown).

To further validate target gene regulation by MYCN/c-MYC in neuroblastoma cells, we performed ChIP-chip using a 244K oligonucleotide promoter microarray (Agilent). We analyzed the binding of MYCN and c-MYC to the promoters of the 147 subgroup I and II genes that were represented on the 244K promoter microarray. We used five neuroblastoma cell lines that either preferentially express high levels of MYCN (SH-EP^*MYCN*^, IMR5/75 (approximately 75 copies of *MYCN*), and Kelly (approximately 100-120 copies of *MYCN*)) or c-MYC (SJ-NB12 and SY5Y). Additionally, as an intermediate type, parental SH-EP cells were analyzed, which preferentially express c-MYC, but also MYCN at low level [20,23]. ChIP-chip experiments were performed with a monoclonal antibody against human MYCN and a polyclonal antibody against human c-MYC for each of the neuroblastoma cell lines. A cut-off for positive binding was defined as >4-fold enrichment for one probe together with >2-fold enrichment for at least one of the two neighboring probes compared to input control. In addition, we manually inspected each of the MYCN and c-MYC-binding profiles from the 147 genes. Seven genes were excluded from the analysis because the probe sets for the genes mapped within the genes but outside the target gene promoter regions (all profiles for Kelly and SJ-NB12 cell lines are given in Additional data files 3 and 4, respectively; MYCN- and c-MYC-binding results are given in Additional data files 5-7). We also performed PCR-based ChIP for selected candidate genes (n = 13; Additional data file 8), which all showed analogous results to ChIP-chip (data not shown). Almost all 140 target gene promoters showed binding of MYCN and/or c-MYC in the six analyzed neuroblastoma cell lines as measured by ChIP-chip (Figure 2b). Intriguingly, hierarchical clustering of neuroblastoma cell lines according to the MYCN/c-MYC-binding pattern clearly separated MYCN- and c-MYC-expressing neuroblastoma cell lines. Differentiation between MYCN and c-MYC binding was mainly achieved through the monoclonal anti-MYCN antibody. The polyclonal antibody against c-MYC also gave positive binding signals for a large set of target gene promoters in neuroblastoma cell lines with high MYCN that lack detectable c-MYC expression (SH-EP^*MYCN*^, IMR5/75 and Kelly). This was most likely due to unspecific binding of the polyclonal c-MYC antibody to MYCN in these cells. Nevertheless, the lack of binding of MYCN to a large set of target gene promoters in the c-MYC-expressing cells, SJ-NB12 and SY5Y, and the positive binding of c-MYC to almost all of these target gene promoters in these cells allowed the distinction between MYCN and c-MYC. Taken together, these results indicate that the genes from subgroups I and II represent a core set of target genes directly regulated by either MYCN or c-MYC in neuroblastoma cells dependent on which MYC protein is expressed.

### Gradual increase of MYCN/c-MYC target gene expression from stage 4s-NA through stage 4-NA to *MYCN *amplified tumors

**Figure 3:**
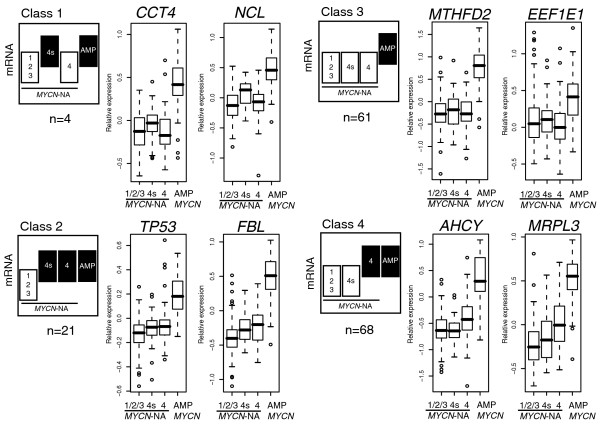
Expression of MYCN/c-MYC target genes in neuroblastoma subtypes. Differential expression was analyzed for each of the genes (n = 154) in *MYCN *amplified (AMP), stage 4s-NA and stage 4-NA tumors using localized-NA (stage 1/2/3) tumors as reference in pair-wise comparisons (Mann-Whitney test, cut-off *p *< 0.05, black). We grouped each of these 154 genes into one of four classes based on their relative expression in clinically relevant neuroblastoma subtypes. These classes were, compared to localized-NA tumors: overexpressed in *MYCN *amplified and in stage 4s-NA tumors (class 1; *CCT4 *and *NCL*); overexpressed in *MYCN *amplified, stage 4-NA and stage 4s-NA tumors (class 2; *TP53 *and *FBL*); overexpressed in *MYCN *amplified tumors (class 3; *MTHFD2 *and *EEF1E1*); and overexpressed in *MYCN *amplified and stage 4-NA tumors (class 4; *AHCY *and *MRPL3*).

To determine transcriptional activity of MYCN/c-MYC proteins in primary neuroblastomas (n = 251), we analyzed differential expression of subgroup I and II genes in neuroblastoma subtypes using the Global test as proposed by Goeman *et al*. [27]. Almost all these genes (154 of 167; 92%) showed highest expression in *MYCN *amplified tumors, suggesting that regulation of these genes by MYCN is similar in neuroblastoma cell lines and tumors. Compared to localized-NA tumors (stages 1, 2, 3), expression of subgroup I and II genes was significantly associated with stage 4s-NA (*p *= 0.002), stage 4-NA (*p *< 0.001) and *MYCN *amplified tumors (*p *< 0.001). Global test results further indicated that an increasing number of MYCN/c-MYC target genes was induced from stage 4s-NA through stage 4-NA to *MYCN *amplified tumors (Additional data files 9-11). To further illustrate this, we grouped each of the 154 genes into one of four classes based on pair-wise comparisons (Mann-Whitney test, cut-off *p *< 0.05). These were, compared to localized-NA tumors: overexpressed in *MYCN *amplified and in stage 4s-NA tumors (class 1); overexpressed in *MYCN *amplified, stage 4-NA and stage 4s-NA tumors (class 2); overexpressed in *MYCN *amplified tumors (class 3); overexpressed in *MYCN *amplified and stage 4-NA tumors (class 4) (Figure 3). Compared to localized-NA tumors, 25 (16%) of the 154 MYCN/c-MYC target genes, including *CCT4*, *FBL*, *MDM2*, *NCL*, *NPM1*, *PTMA*, and *TP53*, were expressed at higher levels in stage 4s-NA tumors (Table 1). Eighty-eight (57%) of the 154 MYCN/c-MYC target genes, including 21 of those overexpressed also in stage 4s-NA tumors, were expressed at higher levels in stage 4-NA than in localized-NA tumors (Table 1, class 2; Additional data file 5). Accordingly, stage 4-NA tumors shared overexpression of 68 of 154 direct MYCN/c-MYC target genes (44%), including *AHCY*, *RUVBL1*, *PHB*, *CDK4*, and *MRPL3*, with *MYCN *amplified tumors. Together, this indicates that besides *MYCN *amplified tumors, stage 4-NA tumors, and to a lesser extent stage 4s-NA tumors, also show higher MYCN/c-MYC activity compared to localized-NA tumors. In line with this, we also found lower mRNA levels of an increasing number of MYCN/c-MYC repressed genes from stage 4s-NA (10 out of 68 (15%) *in vitro *validated repressed genes that are also lower in *MYCN *amplified tumors) through stage 4-NA (34 out of 68 (50%)) to *MYCN *amplified tumors (68 out of 102 *in vitro *validated repressed genes had the lowest expression levels in *MYCN *amplified tumors (67%)). Based on the relative expression of *MYCN *and c-*MYC *in neuroblastoma subtypes, we propose that elevated MYCN activity in stage 4s-NA tumors induces only a restricted set of MYCN/c-MYC target genes, whereas elevated c-MYC activity in stage 4-NA tumors induces a larger set of MYCN/c-MYC target genes.

**Table 1:** MYCN/c-MYC target genes overexpressed in stage 4s-NA compared to localized-NA tumors (classes 1 and 2)

Probe	Gene name	Class	BMU	Group	MYCN/c-MYC-fold change*	c-MYC target DB^†^	Validated by ChIP^‡^
A_24_P311604	*C4orf28*	1	195	I	1.38		+
A_23_P102420	*CCT4*	1	168	I	1.31		+
A_23_P5551	*NCL*	1	308	II	1.69	Up	+
A_23_P44836	*NT5DC2*	1	140	I	1.40		+
A_32_P139196	*C13ORF25V_1*	2	308	II	3.83		ND
A_24_P133488	*CDCA4*	2	140	I	1.45		+
A_23_P137143	*DKC1*	2	308	II	1.93	Up	+
A_23_P216396	*EXOSC2*	2	308	II	1.83		+
A_23_P78892	*FBL*	2	195	I	1.93	Up	+
A_24_P228796	*GAGE7B*	2	195	I	1.27		ND
A_23_P41025	*GNL3*	2	308	II	1.80	Up	ND
A_32_P8120	*GNL3*	2	308	II	1.81	Up	ND
A_23_P398460	*HK2*	2	280	II	1.71	Up	+
Hs172673.9	*Hs172673.9*	2	168	I	1.73		+
A_23_P502750	*MDM2*	2	336	II	1.19	ChIP	+
A_23_P92261	*MGC2408*	2	280	II	2.14		+
A_23_P50897	*MKI67IP*	2	280	II	1.97	Up	+
A_23_P214037	*NPM1*	2	140	I	1.61	Up	+
A_23_P57709	*PCOLCE2*	2	308	II	2.40		+
A_24_P34632	*PTMA*	2	308	II	2.21	Up	+
A_23_P126825	*SLC16A1*	2	195	I	1.22		+
A_23_P126291	*SNRPE*	2	336	II	1.49		+
A_23_P117068	*SNRPF*	2	336	II	1.44		+
A_23_P31536	*SSBP1*	2	336	II	1.24		+
A_23_P26810	*TP53*	2	140	I	1.44	Up	+

*Fold change expression in SH-EP^*MYCN *^cells after *MYCN *induction. ^†^c-MYC target gene database entry [25]: Up, upregulated; ChIP, validated by ChIP. ^‡^Validation of MYCN/c-MYC binding using ChIP in this study (Additional data files 5-7). BMU, best matching unit; ND, not determined.

### High expression of MYCN/c-MYC target genes is a robust marker of poor overall survival independent of genomic *MYCN *status, age at diagnosis and disease stage

**Figure 4:**
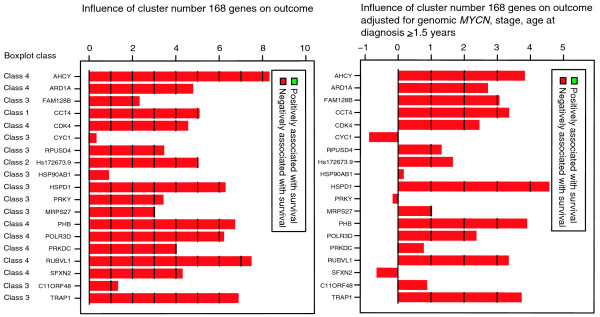
Association of cluster 168 genes with overall survival. The two gene plots illustrate the influence on overall survival of each gene from cluster 168. The gene plot gives the influence on overall survival without (left) and with (right) adjustment for the variables genomic *MYCN *status, age at diagnosis (≥1.5 years), and disease stage (stages 1, 2, 3, 4s versus stage 4). The gene plot shows a bar and a reference line for each gene tested. In a survival model, the expected height is zero under the null hypothesis that the gene is not associated with the clinical outcome (= reference line). Marks in the bars indicate by how many standard deviations the bar exceeds the reference line. The bars are colored to indicate a negative (red) association of a gene's expression with overall survival. In addition, the boxplot class is given for each gene.

**Figure 5:**
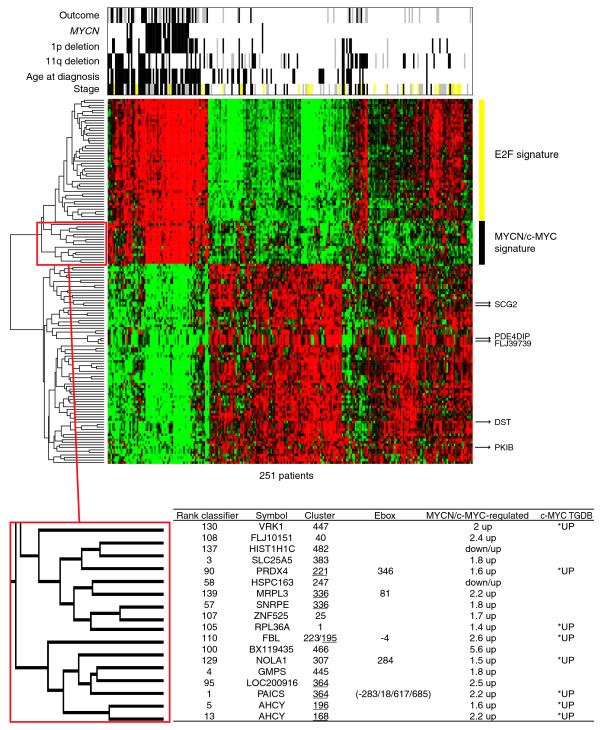
Representation of MYCN/c-MYC target genes in a gene expression-based neuroblastoma risk stratification system. Two-way hierarchical cluster analysis using 144 oligonucleotide probes from the gene expression-based classifier and the 251 patients from the entire cohort. Clinical characteristics (outcome, white = no event, gray = relapse/progression, black = death due to neuroblastoma; genomic *MYCN *status, white = NA, black = amplified; chromosome 1p status, white = normal, black = 1p deleted, gray = not available; chromosome 11q status, white = normal, black = 11q deleted, gray = not available; age at diagnosis, white <1.5 years, black ≥1.5 years; disease stage, white = stage 1, 2, gray = stage 3, yellow = stage 4s, black = stage 4) are added to the heatmap of gene expression. The gene expression cluster with direct MYCN/c-MYC target genes is highlighted. The Rank Classifier column gives the classifier rank found by the Prediction Analysis for Microarrays algorithm and a complete 10-times-repeated 10-fold cross validation. The Cluster column gives the results from the SOM analysis using gene expression profiles from SH-EP^*MYCN *^cells. The MYCN/c-MYC regulated column gives the fold changes after MYCN induction. The Ebox column gives the position of a canonical E-box in the promoter. The c-MYC TGDB column gives the entries in the public c-MYC target gene database. *UP, upregulated.

Having shown that MYCN/c-MYC target gene activation is also associated with distinct neuroblastoma subtypes, we wanted to test whether MYCN/c-MYC activity as determined by the expression levels of their target genes is associated with overall survival and improves outcome prediction independent of known risk markers. We used the Global test to test the influence of each of the 504 experimentally defined gene clusters on overall survival directly, without the intermediary of single gene testing. The *p*-values for each cluster were adjusted for multiple testing and ranked according to their association with overall survival. Table 2 gives the association with overall survival of the six MYCN/c-MYC target gene clusters and the rank in relation to all other clusters. In a separate analysis, we determined the association with overall survival for each of the 504 experimental gene clusters adjusted for amplified *MYCN*, stage 4 versus stages 1, 2, 3, and 4s, and age at diagnosis ≥1.5 years (Table 2). These well-established risk markers highly correlated with poor outcome in univariate analyses (*p *< 0.001 for each of these three markers). As expected, the Global test without adjustment for co-variables indicated that all MYCN/c-MYC target gene clusters were significantly associated with poor overall survival (*p *< 0.001). Intriguingly, all six MYCN/c-MYC target gene clusters remained significantly associated with overall survival after adjusting for amplified *MYCN*, stage 4 versus stages 1, 2, 3, and 4s, and age at diagnosis ≥1.5 years. Of note, two of the MYCN/c-MYC target gene clusters (clusters 168 and 140, both from subgroup I showing a higher responsiveness to c-MYC than to MYCN in SH-EP^*MYCN*^) revealed the strongest association with overall survival of all 504 clusters after adjusting for co-variables (Table 2). Figure 4 shows the association with overall survival for each gene from cluster 168 with and without adjustment for co-variables. Most of the genes within this cluster, such as *AHCY*, *ARD1A*, *CDK4*, *HSPD1*, *PHB*, *RUVBL1*, and *TRAP1*, remained associated with overall survival after adjustment for co-variables. A less significant association with overall survival was observed for clusters with MYCN/c-MYC repressed genes: clusters 454, 482, 484, and 486 were associated with poor overall survival without adjustment for co-variables in the Global test (*p *< 0.001, adjusted for multiple testing), but they showed no significant association with poor overall survival when adjusting for the co-variables amplified *MYCN*, stage 4 versus stages 1, 2, 3, and 4s, and age at diagnosis ≥1.5 years. We also asked whether direct MYCN/c-MYC target genes as defined by our analyses are represented in previously published gene expression-based classifiers that distinguish low-risk from high-risk neuroblastomas independent of other risk markers. Gene lists from these studies hardly overlapped, making interpretation difficult. The overlap with our MYCN/c-MYC target gene list was defined by using the gene names as common identifiers. Indeed, different genes defined by our study as direct MYCN/c-MYC target genes were represented in the gene expression classifier gene lists: from the 44 genes overexpressed in high-risk neuroblastomas independent of other markers described by Schramm *et al*. [28], we identified 10 genes directly regulated by MYCN/c-MYC (*DDX21*, *SCL25A3*, *EIFA4A2*, *NME1*, *NME2*, *TKT*, *LDHA*, *LDHB*, *HSPD1*, *HSPCB*); from the 20 genes overexpressed in high-risk neuroblastomas independent of other markers described by Ohira *et al*. [29], we identified 5 genes directly regulated by MYCN/c-MYC (*EEF1G*, *AHCY*, *TP53*, *ENO1*, *TKT*); and from the 66 genes overexpressed in high-risk neuroblastomas independent of other markers described by Oberthuer *et al*. [24], we identified 7 genes directly regulated by MYCN/c-MYC (*PRDX4*, *MRPL3*, *SNRPE*, *FBL*, *LOC200916*, *PAICS*, *AHCY*; Figure 5). Together, these results show that MYCN/c-MYC activity as determined by the expression status of a subset of MYCN/c-MYC target genes is significantly associated with poor overall survival independent of other established markers and is a consistent element of gene expression-based neuroblastoma risk classification systems.

**Table 2:** Association of MYCN/c-MYC target gene clusters with overall survival in primary neuroblastomas (n = 251)

Cluster	Number of genes	Rank OS*	*p*-value OS^†^	Rank OS with CV*	*p*-value OS with CV^†^
168 (I)	19	3	<0.0001	1	0.0004
140 (I)	38	4	<0.0001	2	0.0006
195 (I)	21	31	<0.0001	12	0.0060
308 (II)	33	18	<0.0001	26	0.0161
280 (II)	32	29	<0.0001	37	0.0232
336 (II)	26	51	<0.0001	45	0.0280

*Rank of all 504 clusters tested for association with overall survival (OS) using the Global test without and with adjustment for co-variables (CV; amplified *MYCN*, stages 1, 2, 3, 4s versus 4, age at diagnosis ≥1.5 years). ^†^*p*-value from Global test adjusted for multiple testing. In the Cluster column, I or II gives the cluster group as defined by the SOM analysis using SH-EP^*MYCN *^cells.

## Discussion

In this study, we analyzed MYCN and c-MYC activity as reflected by the expression levels of a core set of direct MYCN/c-MYC targets in neuroblastoma subtypes. As expected, the highest expression levels of MYCN/c-MYC targets were observed in *MYCN *amplified tumors. However, we found that besides *MYCN *amplified tumors, subtypes of *MYCN *single-copy tumors, namely stage 4-NA and, to a lesser extent, stage 4s-NA, also showed increased MYCN/c-MYC target gene activation compared to localized-NA tumors. In general, low *MYCN *mRNA and protein levels are found in most stage 4-NA tumors [14-16], which does not explain the high mRNA levels of MYCN/c-MYC target genes in this subtype. Here, we describe an inverse correlation of *MYCN *and c-*MYC *expression levels in stage 4-NA and stage 4s-NA tumors. From experiments in neuroblastoma cell lines, it is known that MYCN and c-MYC control their expression via autoregulatory loops and via repressing each other at defined promoter sites [20]. Neuroblastoma cell lines with high expression of MYCN as a result of amplification lack c-MYC expression. Whenever MYCN and c-MYC are co-expressed in neuroblastoma cell lines, c-MYC expression predominates. Together, this suggests that increased activity of c-MYC represses MYCN in a substantial number of stage 4-NA tumors. In contrast, an inverse regulation, namely the repression of c-*MYC *by MYCN, is found in *MYCN *amplified and, to a lesser extent, in stage 4s-NA tumors. It is important to note that localized-NA tumors also express *MYCN *as well as c-*MYC *and it is likely that they are active because these tumors frequently show high tumor cell proliferation indices [14]. Nevertheless, in localized-NA tumors, we did not observe that one MYC transcription factor dominates over the other, such as in the other neuroblastoma subtypes.

Our findings further indicate that MYCN/c-MYC target gene activation gradually increases from stage 4s-NA through stage 4-NA to *MYCN *amplified tumors. High expression of a large number of MYCN/c-MYC target genes was found in stage 4-NA and *MYCN *amplified tumors, but not in stage 4s-NA tumors, which is probably involved in the divergent clinical outcome of these subtypes. This also suggests that MYCN in stage 4s tumors is a weaker transactivator than c-MYC in stage 4-NA tumors. Whether this effect is due to the cellular context in which they are expressed and/or due to different functions of the two MYC proteins in neuroblastoma cells is unclear. In favor of a cellular context factor, we observed that promoter constructs from the *PTMA *gene, which is highly expressed in stage 4s NA and MYCN amplified tumors, showed a strong activation in N-type but not S-type neuroblastoma cell lines despite similar MYCN protein levels (unpublished data). In favor of different functions of the two MYC proteins, our analyses in SH-EP^*MYCN *^cells suggest that a large number of MYCN/c-MYC target genes (subgroup I genes) are less responsive to MYCN than to c-MYC. Another unsolved question is which molecular mechanisms induce elevated MYCN activity in stage 4s-NA tumors or elevated c-MYC activity in stage 4-NA tumors. Candidate pathways involved in differential regulation of MYC proteins are the Sonic hedgehog pathway (Shh) for MYCN activation [30] and the Wnt/beta-catenin pathway for c-MYC activation [31,32]. However, we observed that c-*MYC *mRNA levels are not significantly higher in stage 4-NA than in localized-NA tumors. This suggests that molecular mechanisms that increase c-MYC protein abundance/stability or simply c-MYC activity are involved in MYCN/c-MYC target gene activation in stage 4 tumors.

Our data are in line with a model where stage 4s-NA tumors exhibit a moderate MYCN function gain compared to localized-NA tumors. Both subtypes usually have favorable outcome. Most localized-NA tumors are cured by surgery alone or even regress spontaneously. Stage 4s-NA tumors frequently regress spontaneously but regression can also be induced by a 'mild' chemotherapy. We found that stage 4s-NA tumors express, on average, the highest *MYCN *mRNA levels of all non-amplified tumors [14]. From the experimentally defined direct MYCN target genes, only a restricted set of 25 genes, including *CCT4*, *FBL*, *MDM2*, *NCL*, *NPM1*, *PTMA*, and *TP53*, was overexpressed in stage 4s-NA compared to localized-NA tumors, indicating that elevated *MYCN *in stage 4s-NA tumors only partially activates its downstream target genes. On the one hand, this suggests that moderate MYCN function gain in stage 4s-NA tumors is involved in the metastatic phenotype. On the other hand, moderate MYCN function gain in this subtype is still compatible with, or might even favor, spontaneous regression. From the list of MYCN target genes overexpressed in stage 4s-NA tumors, *TP53 *as a pro-apoptotic gene, and *MDM2*, coding for the direct inhibitor of p53 and mediating pro-tumorigenic activities, are strong candidates to be involved in the unique phenotype of stage 4s-NA tumors. However, it is important to note that *TP53 *and *MDM2 *are co-expressed at higher levels also in stage 4-NA and *MYCN *amplified tumors. Both subtypes initially respond to therapy, but rapidly acquire resistance and frequently show progression/relapse, suggesting that additional conditions activating MDM2 and/or suppressing TP53 functions are acquired. In line with this, alterations disrupting the p14-MDM2-p53 pathway, such as *MDM2 *amplification, *p14 *methylation/deletion, and *TP53 *mutations are found in neuroblastoma cell lines that were established from relapsed patients [33]. In this context, it remains to be shown whether small compounds that selectively inhibit MDM2, such as nutlin-3, and that induce proliferation arrest and apoptosis in neuroblastoma cell lines [34,35] represent a new therapeutic option for high-risk neuroblastomas.

## Conclusions

High expression of a defined subset of direct MYCN/c-MYC target genes turned out to be a robust marker for poor overall survival independent of the established markers, amplified *MYCN*, disease stage (stage 4 versus stages 1, 2, 3, and 4s) and age at diagnosis (≥1.5 years). Recently, several gene expression-based neuroblastoma risk stratification systems have been developed that predict outcome more accurately than established risk markers [24,28,29]. Unfortunately, the classifier gene lists emerging from these studies hardly overlap, which has been ascribed to the different composition of the investigated cohorts and the different high-throughput gene expression platforms used. Our data show that markers of increased MYCN/c-MYC activity are consistently represented in these classifier gene lists, indicating that a gene expression-based classifier that reflects MYCN/c-MYC function should make an attractive tool for neuroblastoma classification and risk prediction.

## Materials and methods

### Patients

All patients from this study (n = 251) were enrolled in the German Neuroblastoma Trials NB90-NB2004 with informed consent and diagnosed between 1989 and 2004 (patient characteristics are in Additional data files 2 and 12). Tumor samples were collected prior to any cytoreductive treatment. The only criterion for patient selection was availability of sufficient amounts of tumor material. Tumor specimens were checked for at least 60% tumor content.

### Neuroblastoma sample preparation and gene expression analysis

Gene expression profiles from the tumors were generated as dye-flipped dual-color replicates using customized 11K oligonucleotide microarrays as previously described [24]. The 11K Agilent microarray was constructed in our laboratory based on extensive neuroblastoma transcriptome information from different whole-genome analyses from primary tumors and neuroblastoma cell lines. These also include comparative transcriptome analysis of *MYCN *amplified versus not amplified tumors as well as of neuroblastoma cell lines with variable/conditional MYCN/c-MYC expression that allowed the enrichment with MYCN/c-MYC-regulated genes [14,24] (unpublished data). The reference for each tumor RNA was an RNA pool of 100 neuroblastoma tumor samples. Data normalization and quality control is described in Additional data file 2. All raw and normalized microarray data are available at the ArrayExpress database (Accession: E-TABM-38) [36].

### Neuroblastoma cell line experiments and SOM analysis

The SH-EP^*MYCN *^cell line, previously also denoted as TET21N [23], expressing a *MYCN *transgene under the control of a tetracycline-repressible element was used to generate gene expression profiles from different time points after *MYCN *induction showing variable MYCN and c-MYC levels. RNA isolation from SH-EP^*MYCN *^cells was performed as previously described [14]. Gene expression profiles were generated as dye-flipped dual-color replicates using the same customized 11K oligonucleotide microarray platform used for the tumor samples. The reference for RNA from SH-EP^*MYCN *^cells after *MYCN *induction was RNA from SH-EP^*MYCN *^cells cultured in parallel that lack *MYCN *expression. Gene expression profiles from SH-EP^*MYCN *^cells with variable MYCN and c-MYC levels were taken for a SOM analysis (Additional data file 2). Protein expression was assessed by immunoblotting using 50 μg of total cell lysates from the cell line experiments as previously described [37]. Blots were probed with antibodies directed against MYCN (SantaCruz, sc-53993, Santa Cruz, CA, USA) and c-MYC (SantaCruz, sc-764, Santa Cruz, CA, USA).

### ChIP, ChIP-chip and protein analysis

Chromatin immunoprecipitation was performed as described previously [38,39] using 10 μg of MYCN (SantaCruz, sc-53993), c-MYC (SantaCruz, sc-764) [40,41] and normal mouse IgG (SantaCruz, sc-2025) antibodies and Dynabeads ProteinG (Invitrogen, Carlsbad, CA, USA). Eluted and purified MYCN-ChIP-DNA (1 μl) of *IMR5/75 *and *SH-EP*^*MYCN *^was used as a template in PCR reactions running for 35 cycles. The primer sequences are given in Additional data file 8. In addition, ChIP-DNA templates from *SH-EP*^*MYCN*^, *SH-EP*, *Kelly*, *IMR5/75*, *SJNB-12 *and *SY5Y *cells using MYCN and c-MYC antibodies were amplified for DNA microarray analysis (Agilent Human Promoter ChIP-chip Set 244K) using the WGA (Sigma-Aldrich, St. Louis, MO, USA) method [42]. DNA labeling, array hybridization and measurement were performed according to Agilent mammalian ChIP-chip protocols. For the visualization of ChIP-chip results, the cureos package v0.2 for R was used (available upon request). The *in silico *promoter analysis for the identification of putative MYC binding sites (canonical and non-canonical E-boxes) is described in Additional data file 2.

### Differential gene expression and survival analysis

Differential gene expression of *MYCN*/c-*MYC *and their target genes in neuroblastoma tumors was evaluated for stage 4s-NA, stage 4-NA and *MYCN *amplified using localized-NA tumors (stages 1, 2, 3) as reference using Goeman's Global test and the Wilcoxon rank sum test. A result was judged as 'statistically significant' at a *p*-value of 0.05 or smaller. Differential expression of *MYCN *was evaluated in two partially overlapping cohorts, one measured by quantitative PCR [14] and the other by oligo microarray (the overlap was 101 patients). To test the association of *MYCN in vitro *clusters with overall survival (death due to neuroblastoma disease), Goeman's Global test was used [27]. To evaluate the influence of gene expression on outcome independent of established markers, the Global test was adjusted for the following co-variables: genomic *MYCN *status, stage of the disease (stage 4 versus stages 1, 2, 3, and 4s), and age at diagnosis (≥1.5 years versus <1.5 years). Because of multiple testing of probably dependent gene clusters, *p*-values were adjusted according to Benjamini and Yekutieli [43] to control the false discovery rate of 5%.

## Abbreviations

ChIP, PCR-based chromatin immunoprecipitation; ChIP-chip, array-based chromatin immunoprecipitation; NA, non-amplified; SOM, self-organizing map.

## Authors' contributions

FW designed and coordinated the study. FW and DM interpreted results and drafted the manuscript. AO, MF, AB, BB and FW carried out array-based expression profiling and data analyses of neuroblastoma tumor samples and cell lines. BH was responsible for clinical data management. TB and RK performed *in silico *promoter analyses. JV and FP contributed samples and performed literature searches of MYCN/c-MYC target genes. DM performed chromatin immunoprecipitation experiments. DM, TB and FW analyzed ChIP-chip data. AB, BH and FW carried out global test and survival analyses. FW, DM, KOH, JV, FP and MS contributed to the manuscript. All authors read and approved the final manuscript.

## Additional data files

The following additional data are available. Additional data file 1 is a figure showing a Cluster map of genetic programs regulated by conditional expression of c-MYC and MYCN proteins in SH-EP^MYCN ^cells. Additional data file 2 is a document describing in more detail the methods and materials. Additional data files 3 and 4 are sets of figures showing ChIP-chip results of MYCN/c-MYC target genes in the Kelly and SJ-NB12 cell lines. Additional data files 5, 6 and 7 are tables listing MYCN/c-MYC target genes overexpressed in stage 4s-NA, stage 4-NA and *MYCN *amplified tumors, respectively, compared to localized-NA tumors. Additional data file 8 is a table of genes and primers selected to confirm ChIP-chip results. Additional data files 9, 10 and 11 are figures showing the association of MYCN/c-MYC induced genes with neuroblastoma subtypes using the Global test. Additional data file 12 is a table providing patient data.

## Supplementary Material

Additional data file 1 Cluster map of genetic programs regulated by conditional expression of c-MYC and MYCN proteins in SH-EP^MYCN ^cells. Click here for file

Additional data file 2 Detailed methods and materials. Click here for file

Additional data file 3 ChIP-chip results of MYCN/c-MYC target genes in the Kelly cell line. Click here for file

Additional data file 4 ChIP-chip results of MYCN/c-MYC target genes in the SJ-NB12 cell line. Click here for file

Additional data file 5 MYCN/c-MYC target genes, which grouped in class 1 and 2. Click here for file

Additional data file 6 MYCN/c-MYC target genes, which grouped in class 4. Click here for file

Additional data file 7 MYCN/c-MYC target genes, which grouped in class 3. Click here for file

Additional data file 8 Genes and primers selected to confirm ChIP-chip results. Click here for file

Additional data file 9 Association of MYCN/c-MYC induced genes with stage 4s-NA neuroblastomas using the Global test. Click here for file

Additional data file 10 Association of MYCN/c-MYC induced genes with stage 4-NA neuroblastomas using the Global test. Click here for file

Additional data file 11 Association of MYCN/c-MYC induced genes with *MYCN *amplified neuroblastomas using the Global test. Click here for file

Additional data file 12 Patient data. Click here for file
