# Supplementary figures and images for: Distinct transcriptional MYCN/c-MYC activities are associated with spontaneous regression or malignant progression in neuroblastomas
Source: Genome Biol. 2008 Oct 13;9(10):R150. doi: 10.1186/gb-2008-9-10-r150 (PMC2760877; doi:10.1186/gb-2008-9-10-r150)

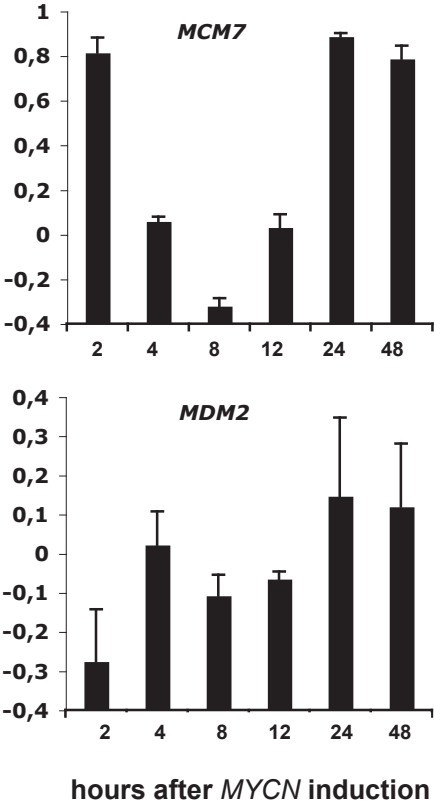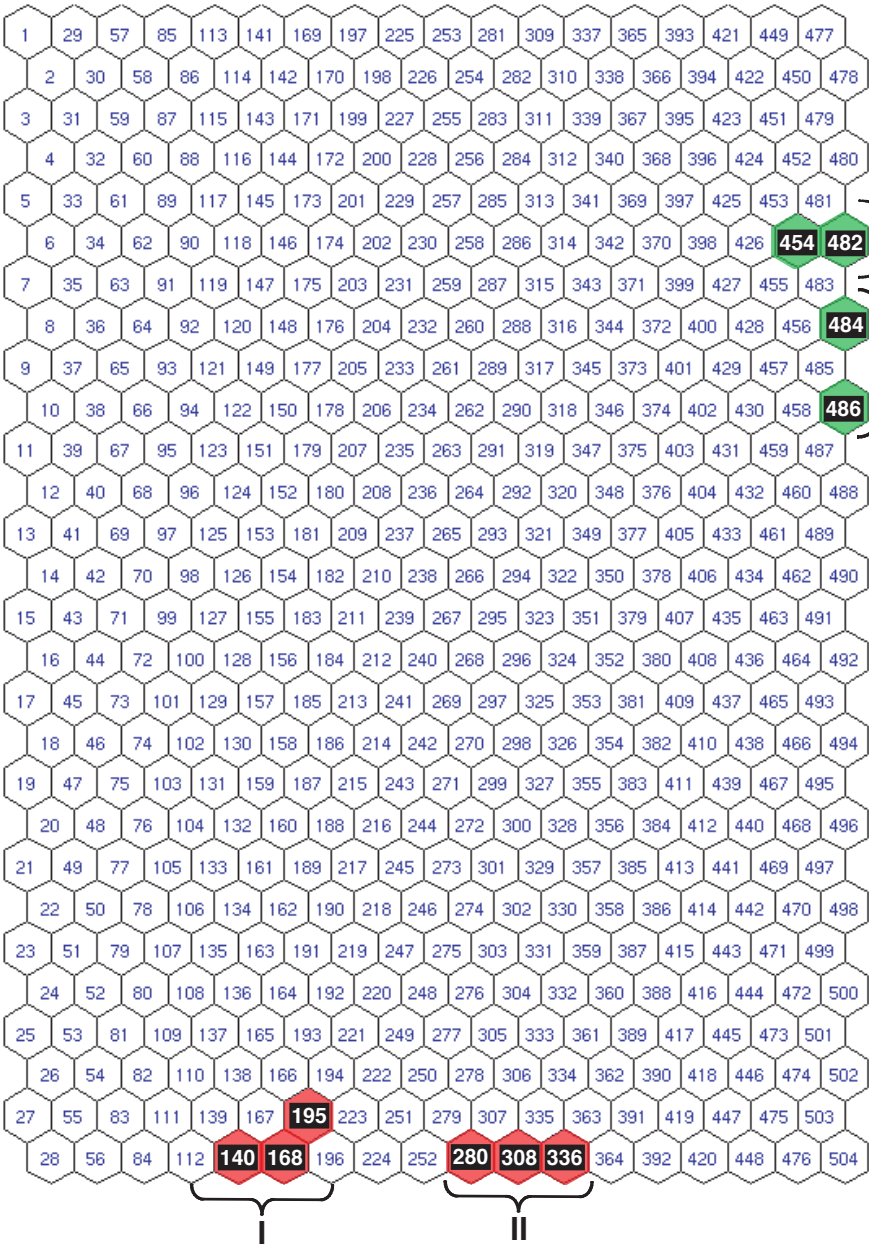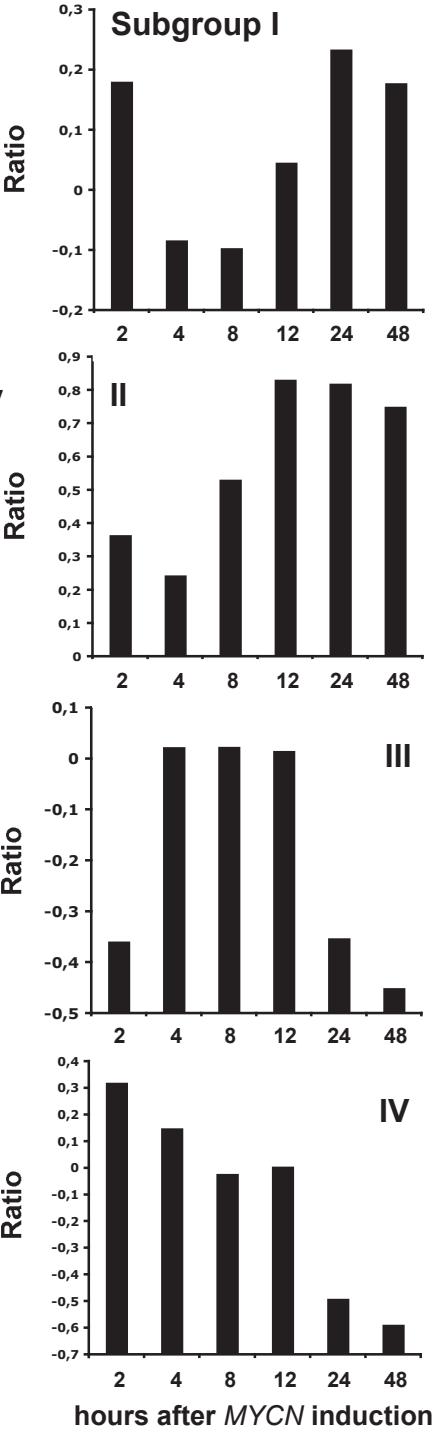

Supplement: Additional data file 1 — Cluster map of genetic programs regulated by conditional expression of c-MYC and MYCN proteins in SH-EPMYCN cells. [file gb-2008-9-10-r150-S1.pdf]

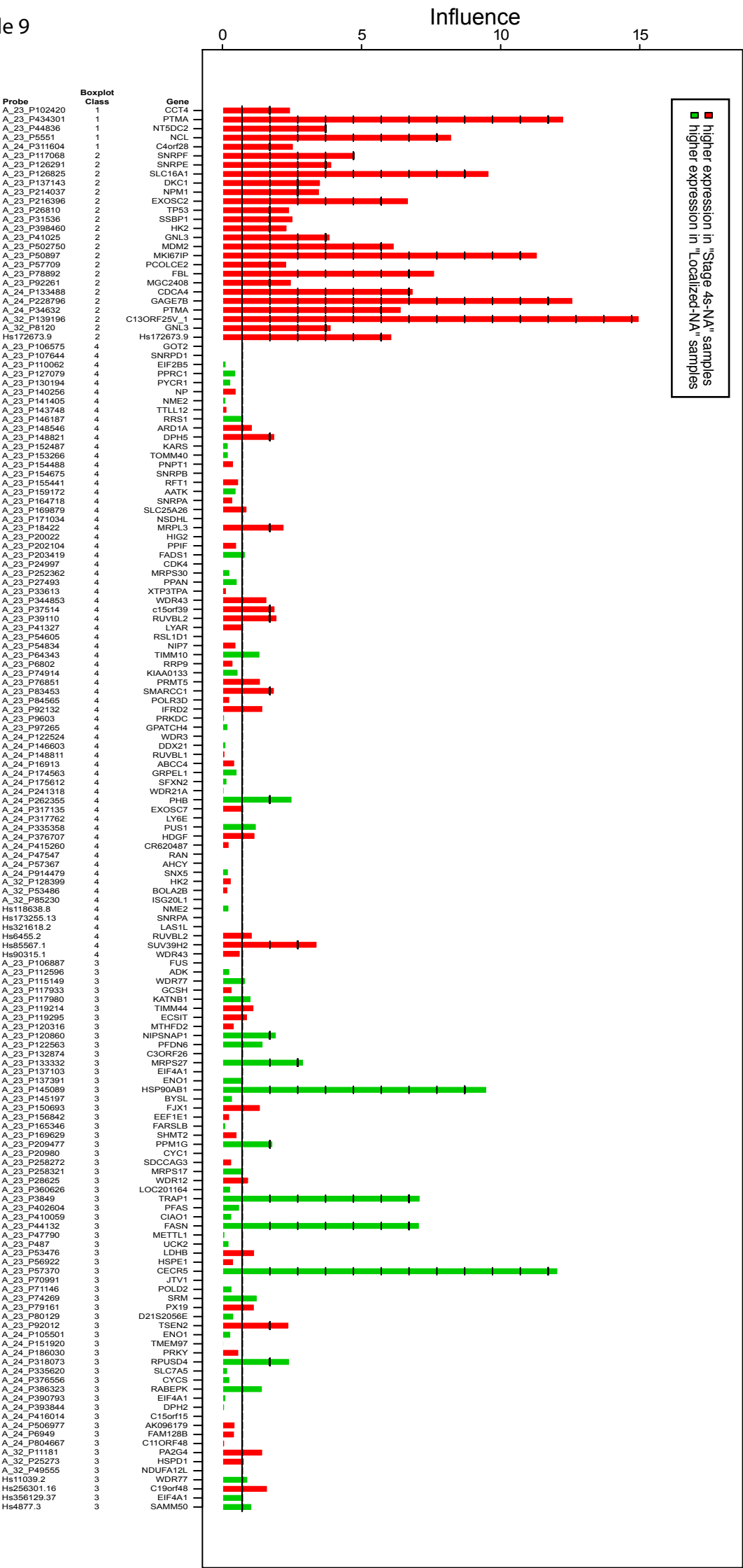

Supplement: Additional data file 9 — Association of MYCN/c-MYC induced genes with stage 4s-NA neuroblastomas using the Global test. [file gb-2008-9-10-r150-S9.pdf]

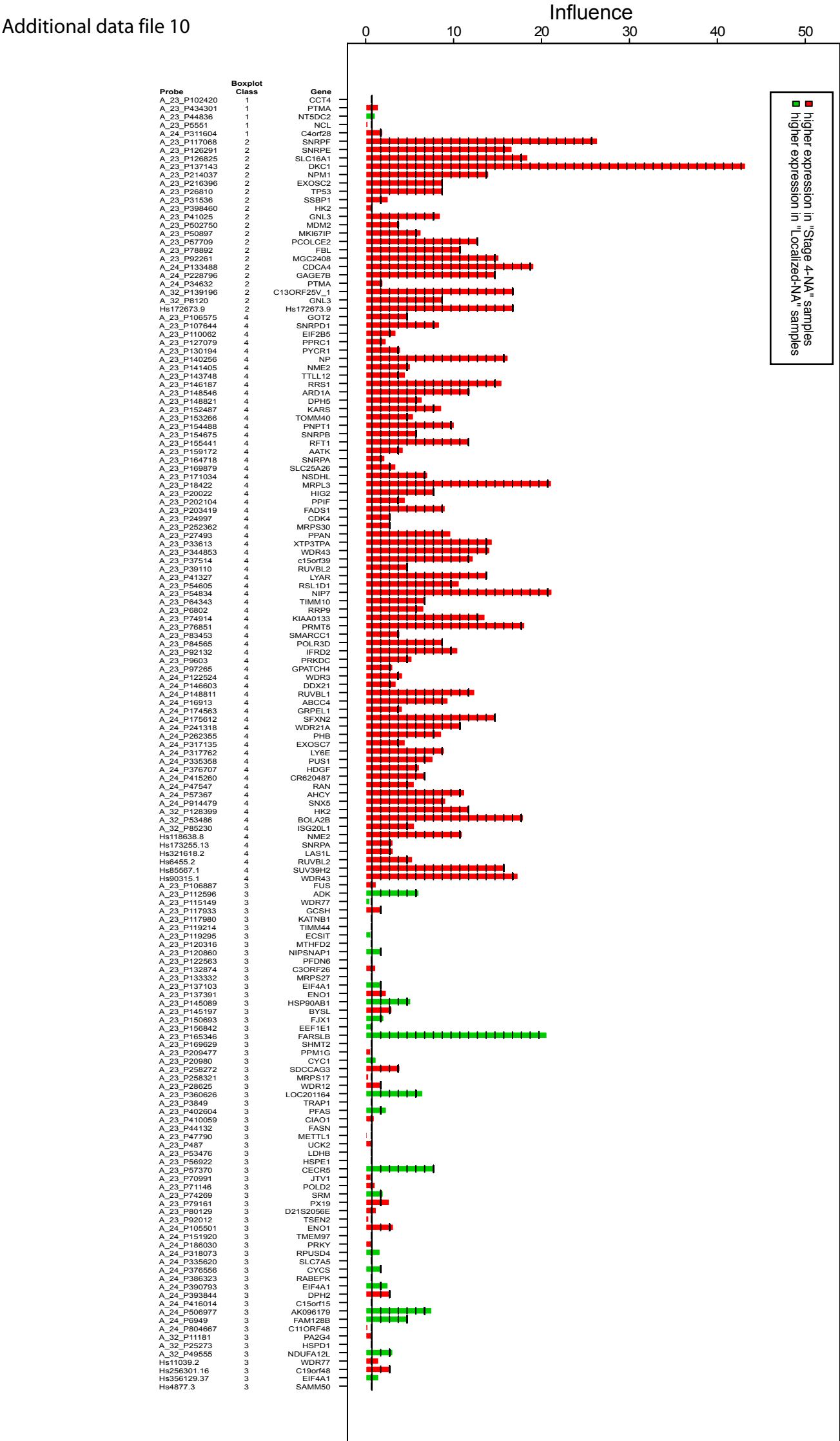

Supplement: Additional data file 10 — Association of MYCN/c-MYC induced genes with stage 4-NA neuroblastomas using the Global test. [file gb-2008-9-10-r150-S10.pdf]

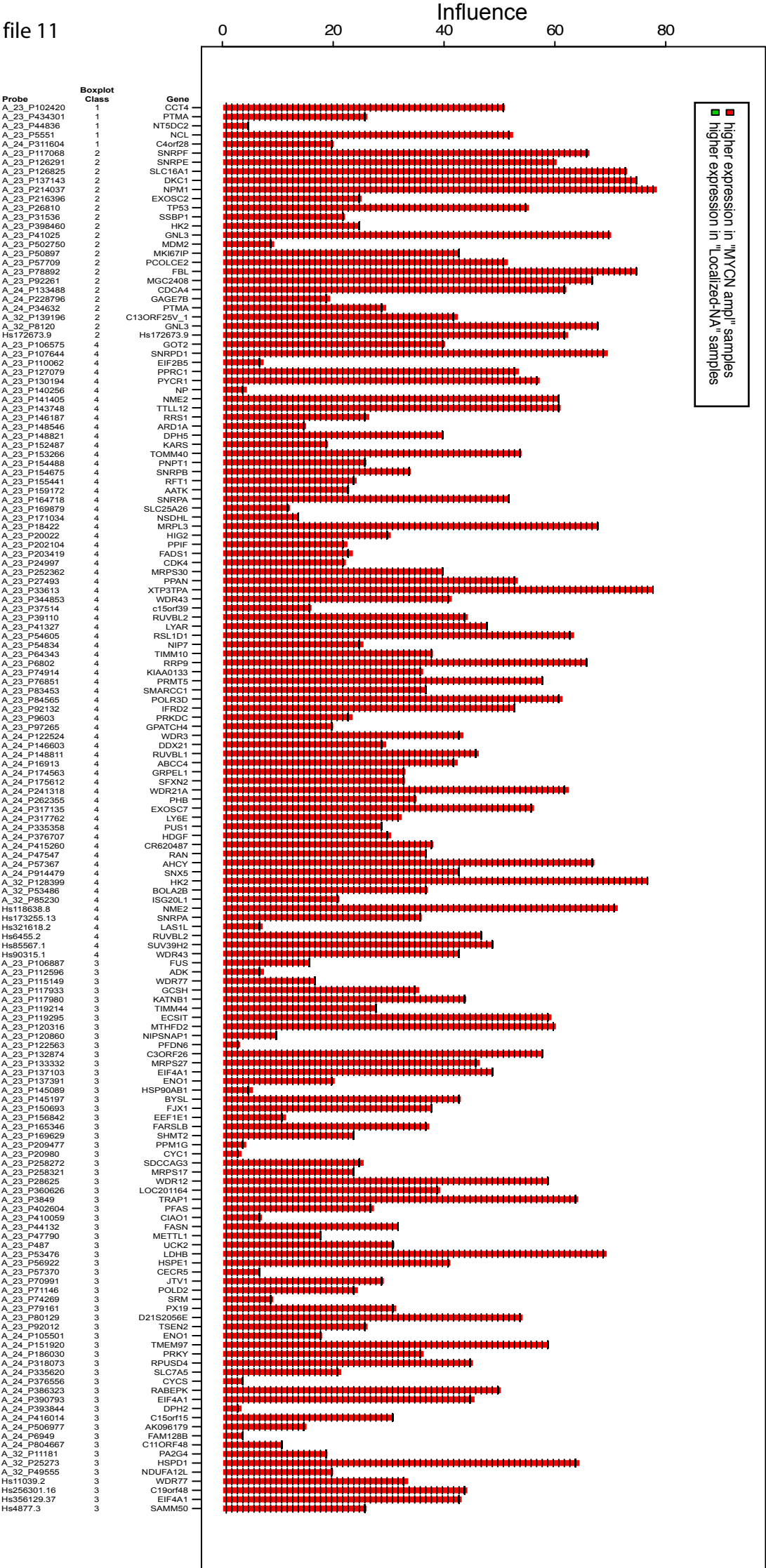

Supplement: Additional data file 11 — Association of MYCN/c-MYC induced genes with MYCN amplified neuroblastomas using the Global test. [file gb-2008-9-10-r150-S11.pdf]
