# Supplementary material for: Distinct transcriptional MYCN/c-MYC activities are associated with spontaneous regression or malignant progression in neuroblastomas
Source: Genome Biol. 2008 Oct 13;9(10):R150. doi: 10.1186/gb-2008-9-10-r150 (PMC2760877; doi:10.1186/gb-2008-9-10-r150)
Supplement: Additional data file 2 — Detailed methods and materials. [file gb-2008-9-10-r150-S2.doc]

**Additional data file 2**

**Patients**

All patients from this study (n=251) were enrolled in the German Neuroblastoma Trials NB90-NB2004 with informed consent and diagnosed between 1989 and 2004 (see Additional data file 12). Tumor samples were collected prior to any cytoreductive treatment. The only criterion for patient selection was availability of sufficient amounts of tumor material. Patients’ age at diagnosis ranged from 0 to 296 month (median age, 15 months). Median follow-up for patients without fatal events was 4.5 years (range, 0.8 to 15.6 years). Stage was classified according to the International Neuroblastoma Staging System (INSS) [44]; response to treatment was defined according to the revised criteria of the International Neuroblastoma Response Criteria (INRC) [44]. Standard molecular markers were assessed in the reference laboratories of the German neuroblastoma trial in Cologne, Heidelberg, Marburg, Stuttgart, and Zurich. Chromosomal aberrations were defined according to the guidelines of the European Neuroblastoma Quality Assessment Group [45]. Tumor specimens were checked for at least 60% tumor content.

**Neuroblastoma sample preparation and gene expression analysis**

Total RNA was isolated from 30 to 60 mg of snap-frozen tissue using the FastPrep FP120 cell disruptor (Qbiogene-Inc) and the TRIzol reagent (Invitrogen). RNA integrity was assessed using the 2100 Bioanalyzer (Agilent Technologies) considering only samples with an RNA Integrity Number of at least 7.5. Gene expression profiles from the tumors were generated as dye-flipped dual-color replicates using customized 11K olignucleotide microarrays as previously described [24]. The reference for each tumor RNA was an RNA pool of 100 neuroblastoma tumor samples. Raw microarray data were preprocessed using software packages from the *R*-project [46] and Bioconductor [47]. Quality control was performed utilizing the package ArrayMagic [48]. Samples were normalized using the variance stabilization algorithm (R package vsn version 1.12.0) [49] and data from dye-flipped chip pairs were averaged. All raw and normalized microarray data are available at the ArrayExpress database (Accession: E-TABM-38) [36].

***In silico* promoter analysis**

All available promoters of genes represented on the customized neuroblastoma oligo array were extracted from Ensembl [50] database (release 41, October 2006). We conducted two different scans for putative MYCN/c-MYC binding sites within +2kb and -2kb from the transcriptional start: (1) a scan for canonical E-box sequences (5’-CACGTG-3’) using a Perl script and (2) a scan with a position-weight matrix (PWM) using the cureos package v0.2 for the R open-source software [46]. The 12 bp MYCN PWM (V$NMYC_01) was taken from the TRANSFAC [26] database (release 10.3, September 2006). P-values for each PWM hit in each of the 4kb promoter sequence were calculated by computing the PWM score based on the likelihoods for each position of the PWM and comparing it to the score distribution of 1,000 random permutations of the respective 4kb promoter sequence. CpG island and mammalian conservation information was retrieved from the UCSC genome browser (Hg 18, release March 2006). For the visualization of ChIP-chip results, the cureos package v0.2 for R was used (available upon request).

**Literature search for MYCN regulated genes**

We used PubMed for the literature search and analysed all abstracts of papers retrieved with the following query: (("MYCN" or "n-myc" or "Nmyc") or ((MYCN or n-myc or Nmyc) and ((amplifi*) or (neuroblastom* or glioblastom* or medulloblastom* or PNET or "melanotic neuroectodermal tumor" or "small cell lung cancer" or retinoblastom* or astrocytom* or rhabdomyosarcom* or "medullary thyroid carcinoma")))) and (express* or transcri* or translat* or downstream or target or overexpress* or repress* or knock-out or upregulat* or up-regulat* or downregulat* or down-regulat*).

If the title or the abstract text was indicative that informative gene expression data or transcription factor-target gene interaction data of MYCN and downstream genes was present in the paper, we carefully analysed the full text. We did the same analysis on the abstracts of all the mentioned references where applicable in the full text. We noticed that we could pick up some additional papers with this additional step which were missed by the initial search query. They were mostly papers where the emphasis of the study was not on finding MYCN target genes. We grouped all the information in an Access database, which we use for data management. We kept record of mRNA and protein expression analysis, promoter analysis (presence of E-boxes) and the different DNA-protein interaction analysis methods (ChIP, EMSA, promoter reporter assays). At regular moments we publish the information on the MYCNot web site which will become publicly available in the nearby future [51]. Reviewers can log in using the password 'MYCNotyes'. After logging in, you can click on the 'Search by gene link' to see more information. For the moment, we have approximately 1100 records holding information about 880 protein coding genes and 25 miRNAs from 114 published studies.

**SOM analysis**

Gene expression profiles from SH-EP*^MYCN^* cells after *MYCN* induction were taken for a self-organizing maps (SOM) analysis. Unsupervised learning with the self-organizing maps technique [52] was performed to yield a sufficient number of discrete clusters, also called best matching units (BMU). We applied the Matlab implementation of the SOM Helsinki package, version 2.0 beta [53]. Given our data, the algorithm chose a Kohonen map of 28 x 18 = 504 distinct hexagonal BMUs. Gene expression profiles were clustered within an intensive learning procedure (overnight runs on a 2.6 GHz PC, 5000 iterations for fine and coarse tuning, respectively). In addition, we identified all genes that are known MYCN target genes from a literature search using PubMed and/or that are deposited in the c-MYC target gene database (Version 2006/12/07) [25] and that are represented on the neuroblastoma oligo array. 380 of 501 c-MYC-induced and 161 of 231 c-MYC-repressed genes from the c-MYC target gene database and 23 of 33 MYCN-induced and 6 of 12 MYCN-repressed genes from the literature are represented on the customized array. We defined an enrichment of genes listed as up and down-regulated in the c-MYC target gene database for each cluster using Fisher’s exact test. p-values were adjusted according to Benjamini and Yekutieli to control the false discovery rate of 5% [43] and the clusters ranked subsequently. The significant ranking clusters were again tested if they showed enrichment of genes with at least one canonical E-box against the Null-hypothesis that they are randomly distributed using Fisher’s exact test.

**Hierarchical cluster analysis**

To visualize expression of the previously described classifier genes [24] using the entire neuroblastoma cohort, a two-way hierarchical cluster analysis was done using the complete linkage method and the Euclidean distance measure.

**Differential gene expression and survival analysis**

Differential gene expression of MYCN/c-MYC-regulated genes in NB tumors was evaluated for stage 4s-NA, stage 4-NA and *MYCN* amplified using localized-NA tumors (1, 2, 3) as reference and the Wilcoxon rank sum test. A result was judged as ‘statistically significant’ at a p-value of 0.05 or smaller. To test the association of *MYCN* *in vitro* clusters with overall survival (death due to neuroblastoma disease), Goeman’s global test was used [27]. To evaluate the influence of genes’ expression on outcome independent of established markers, the global test was adjusted for the following co-variables: genomic *MYCN* status, stage of the disease (stage 4 versus stage 1, 2, 3, 4s), and age at diagnosis (>=1.5 years versus <1.5 years). Because of multiple testing of probably dependent gene clusters, p-values were adjusted according to Benjamini and Yekutieli to control the false discovery rate of 5% [43]. For computation of the global test we used the R package ‘globaltest’, version 4.4.0. Univariate survival analysis for established prognostic variables was done by means of the log-rank test (R packages ‘Design’ version 2.0-12 and ‘survival’ version 2.29).
