# Supplementary material for: Distinct transcriptional MYCN/c-MYC activities are associated with spontaneous regression or malignant progression in neuroblastomas
Source: Genome Biol. 2008 Oct 13;9(10):R150. doi: 10.1186/gb-2008-9-10-r150 (PMC2760877; doi:10.1186/gb-2008-9-10-r150)
Supplement: Additional data file 3 — ChIP-chip results of MYCN/c-MYC target genes in the Kelly cell line. [file gb-2008-9-10-r150-S3.pdf]

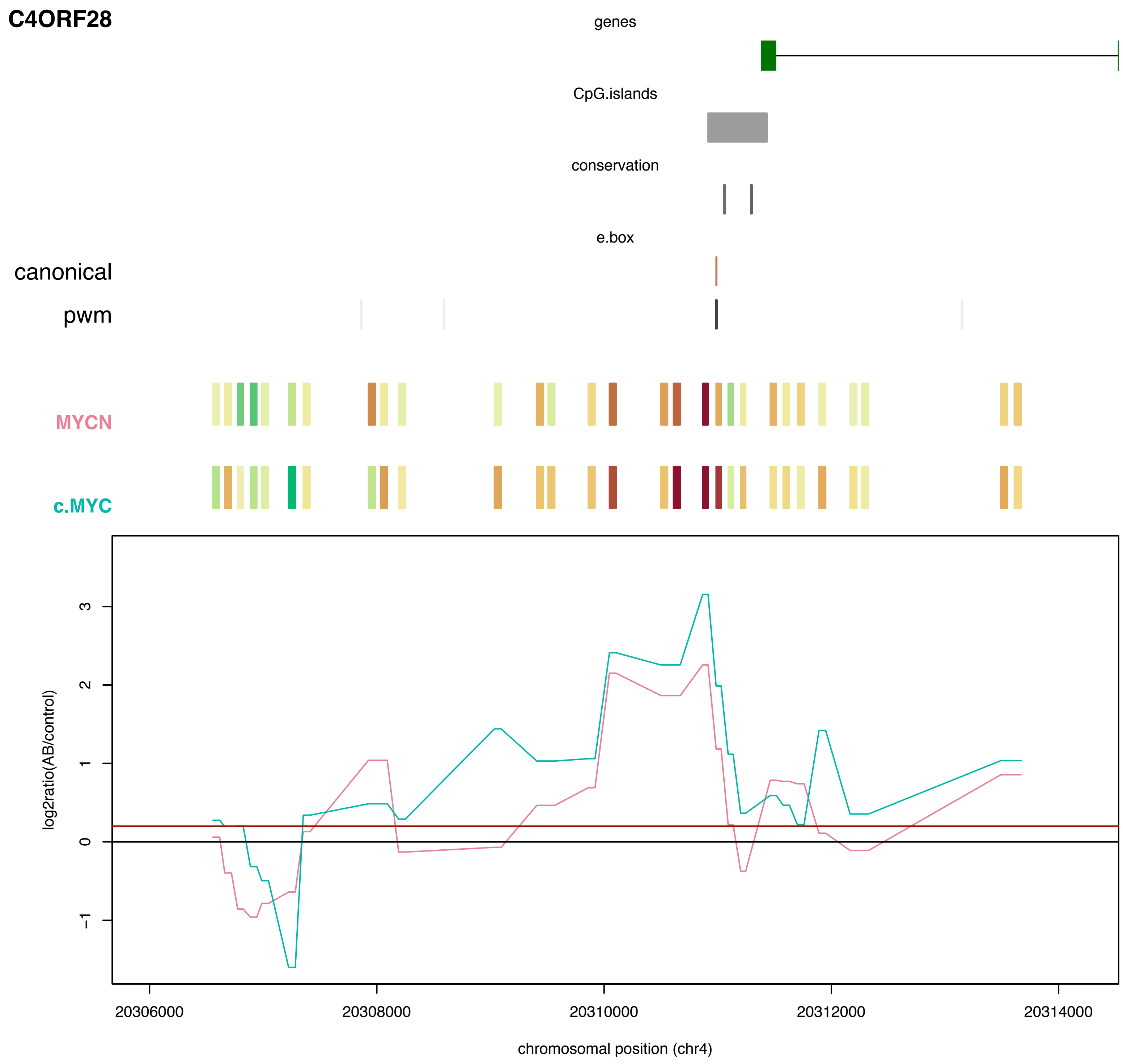
C4ORF28
genes
CpG.islands
conservation
e.box
canonical
pwm
MYCN
c.MYC
log2ratio(AB/control)
3
2
1
0
−1
20306000
20308000
20310000
20312000
20314000
chromosomal position (chr4)

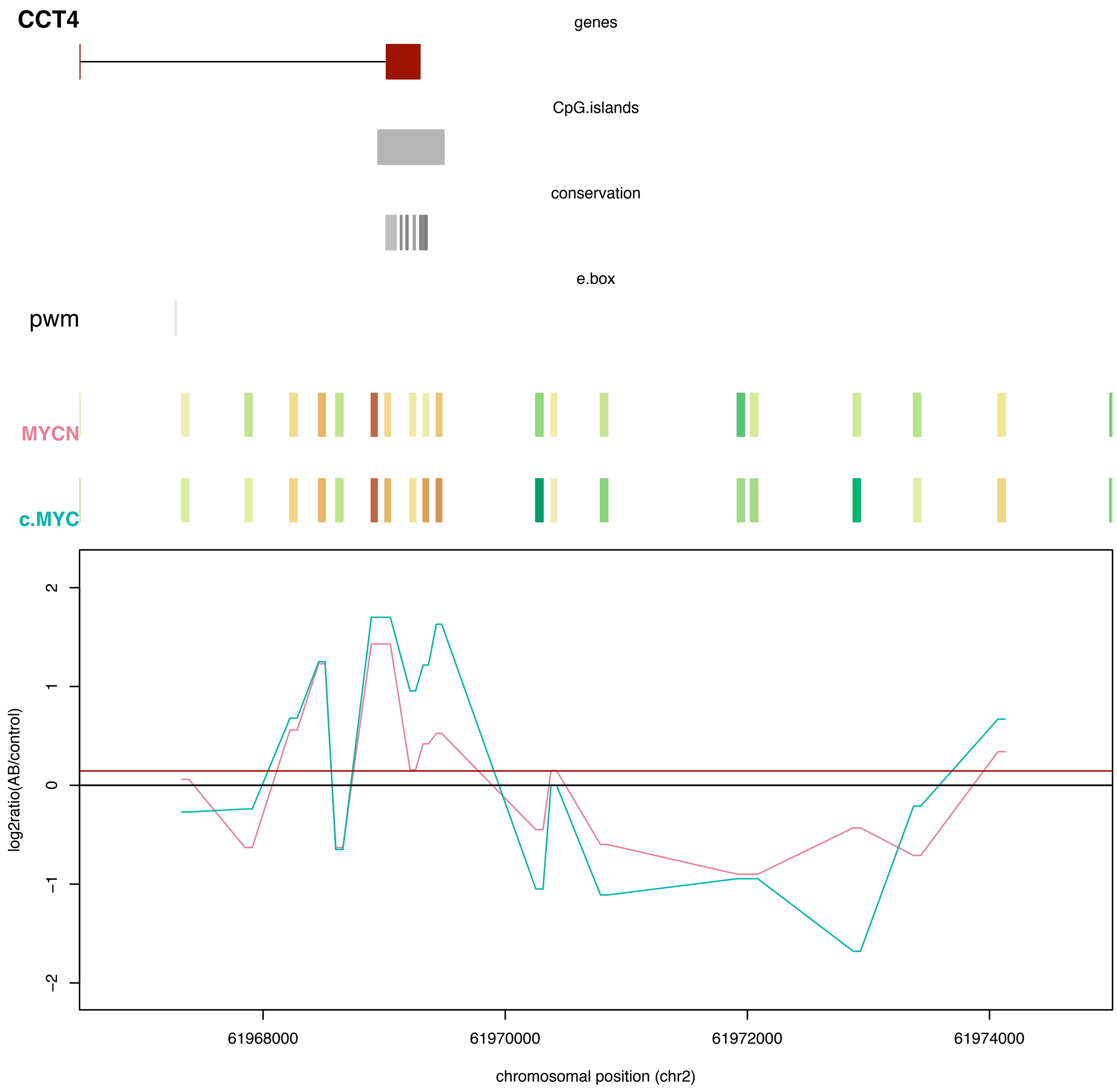

CCT4
genes
CpG.islands
conservation
e.box
pwm
MYCN
c.MYC
2
1
0
−1
−2
log2ratio(AB/control)
61968000
61970000
61972000
61974000
chromosomal position (chr2)

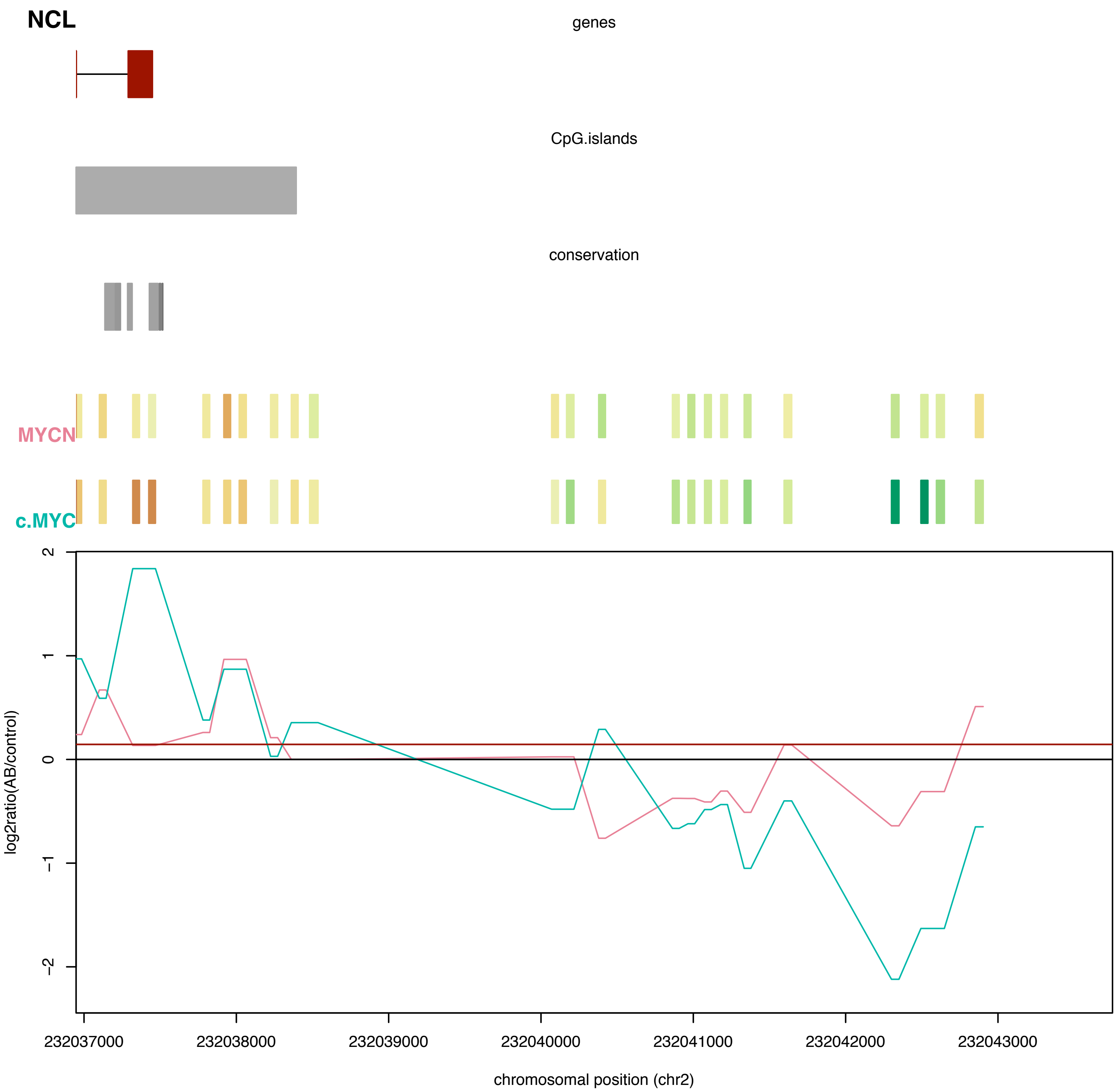

NCL
genes
CpG.islands
conservation
MYCN
c.MYC
log2ratio(AB/control)
2
1
0
−1
−2
232037000
232038000
232039000
232040000
232041000
232042000
232043000
chromosomal position (chr2)

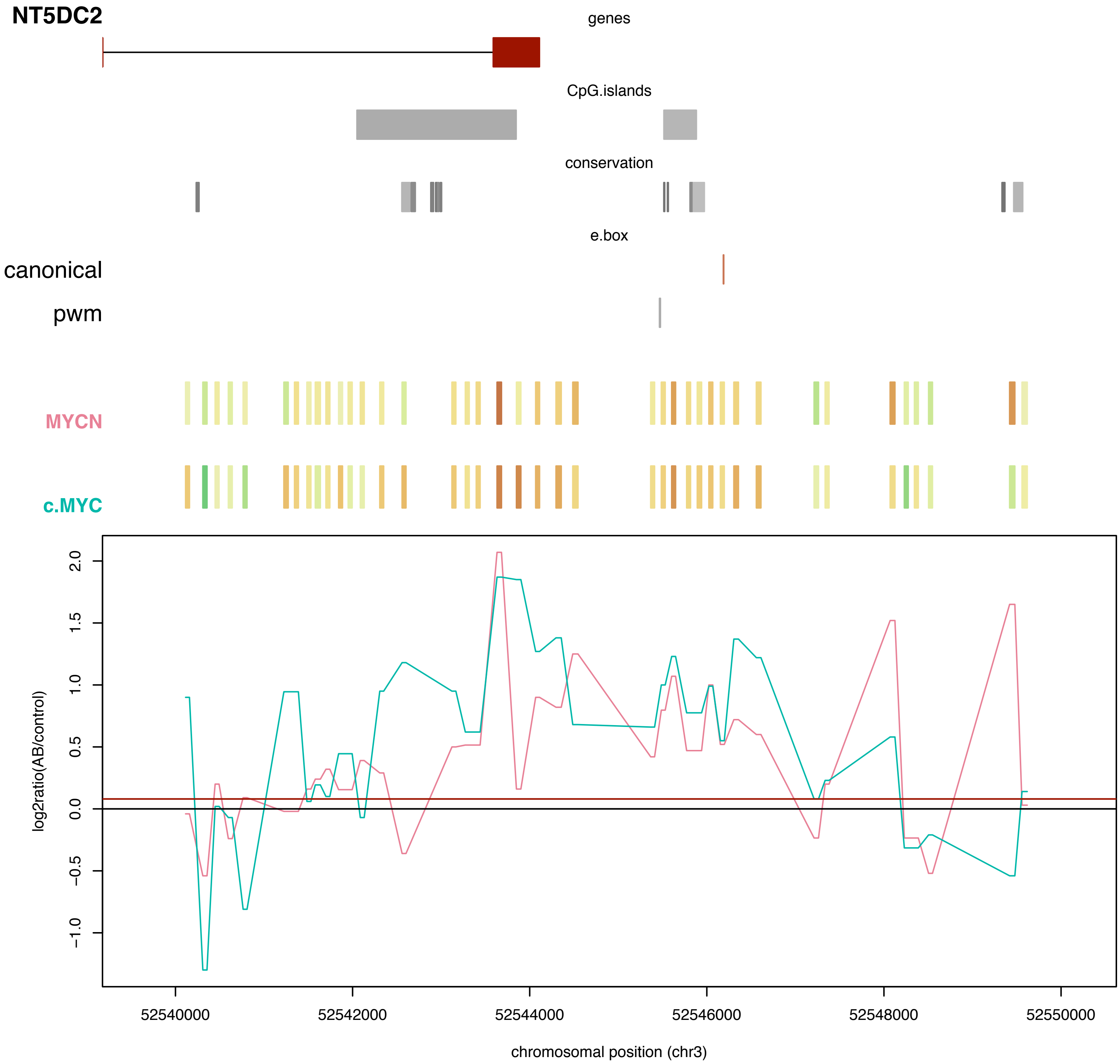

NT5DC2
genes
CpG.islands
conservation
e.box
canonical
pwm
MYCN
c.MYC
log2ratio(AB/control)
2.0
1.5
1.0
0.5
0.0
−0.5
−1.0
52540000
52542000
52544000
52546000
52548000
52550000
chromosomal position (chr3)

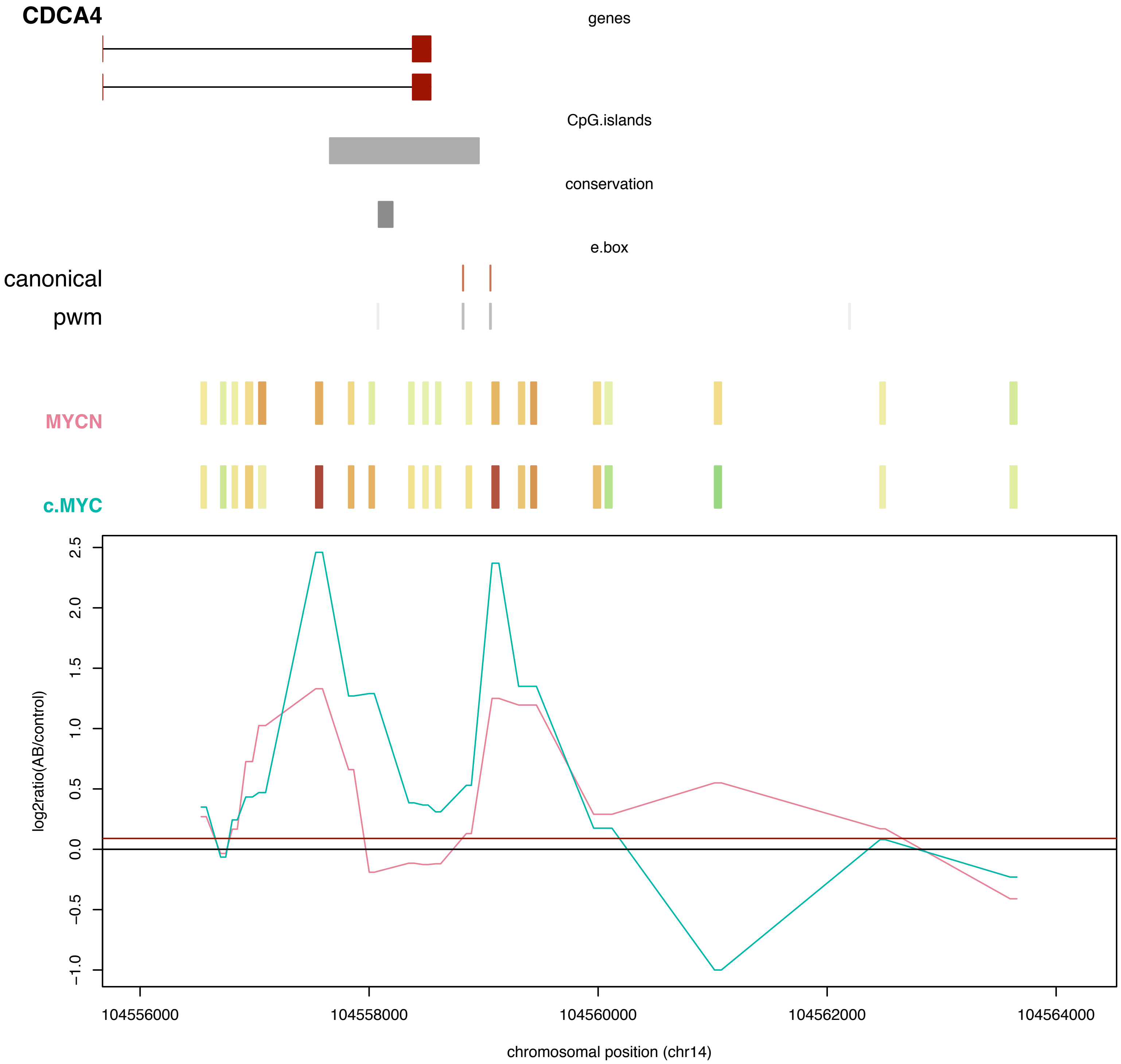

CDCA4
genes
CpG.islands
conservation
e.box
canonical
pwm
MYCN
c.MYC
2.5
2.0
1.5
1.0
0.5
0.0
−0.5
−1.0
log2ratio(AB/control)
104556000
104558000
104560000
104562000
104564000
chromosomal position (chr14)

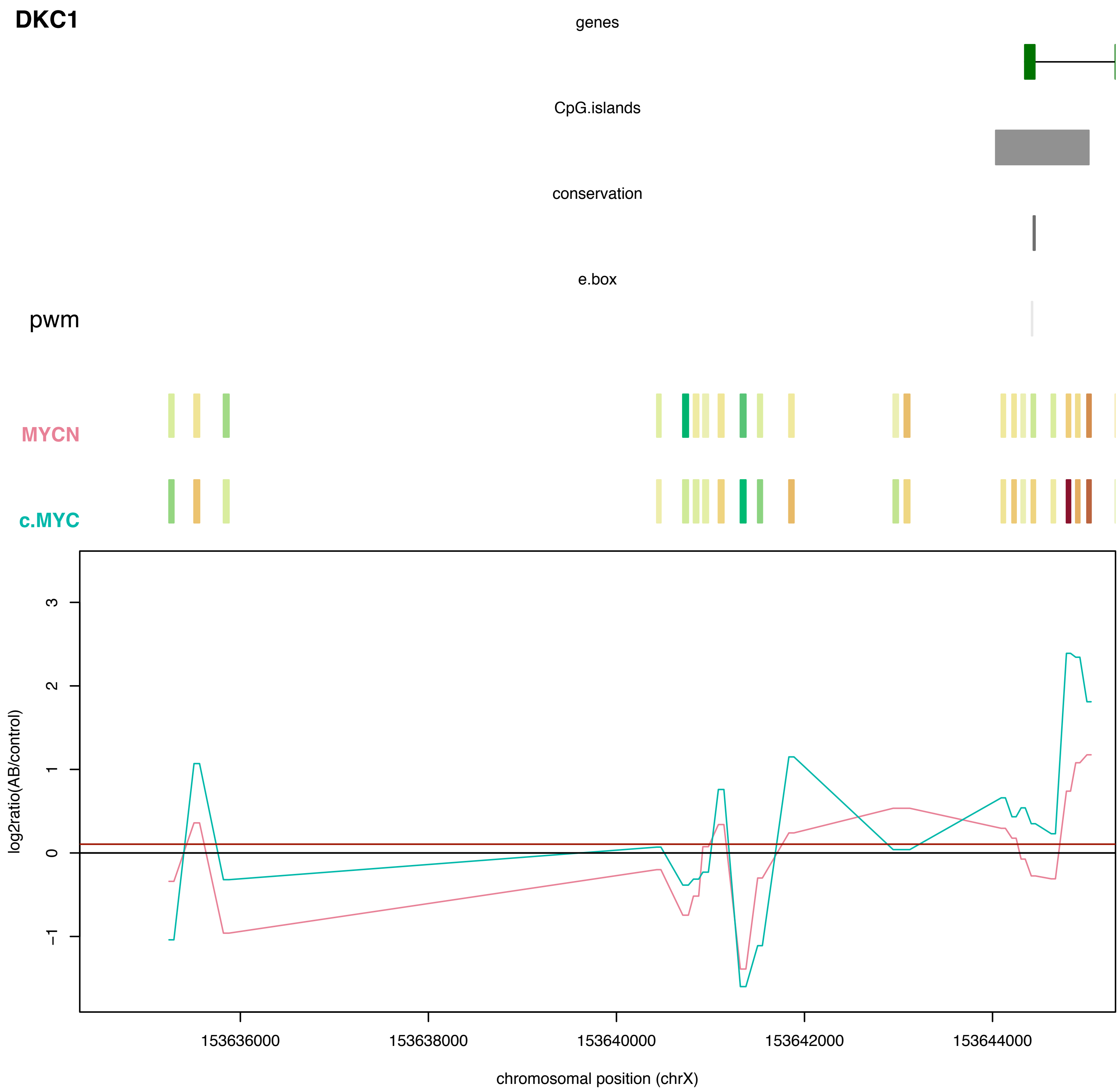

DKC1
genes
CpG.islands
conservation
e.box
pwm
MYCN
c.MYC
log2ratio(AB/control)
3
2
1
0
−1
153636000
153638000
153640000
153642000
153644000
chromosomal position (chrX)

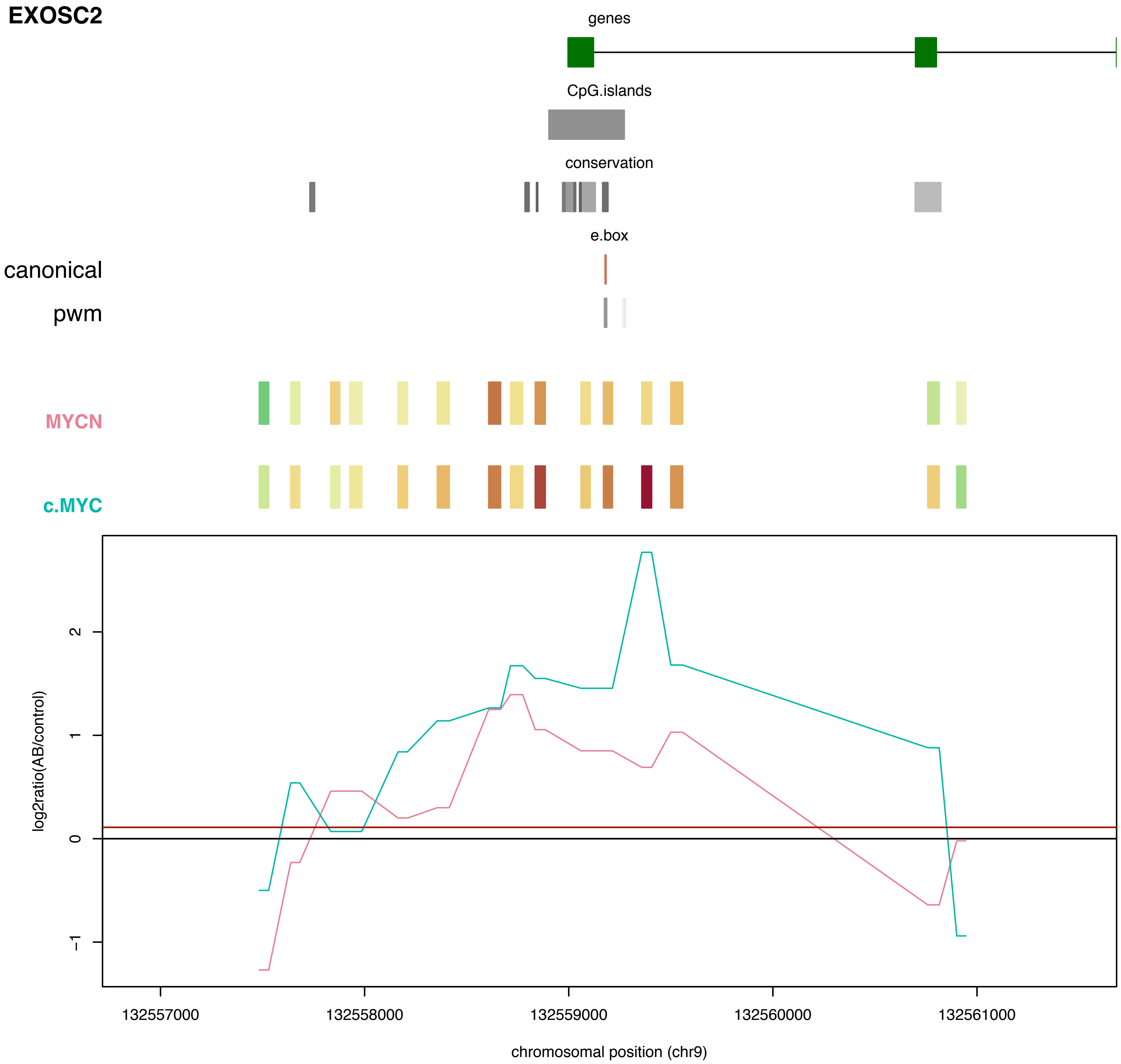

EXOSC2
genes
CpG.islands
conservation
e.box
canonical
pwm
MYCN
c.MYC
log2ratio(AB/control)
2
1
0
−1
132557000
132558000
132559000
132560000
132561000
chromosomal position (chr9)

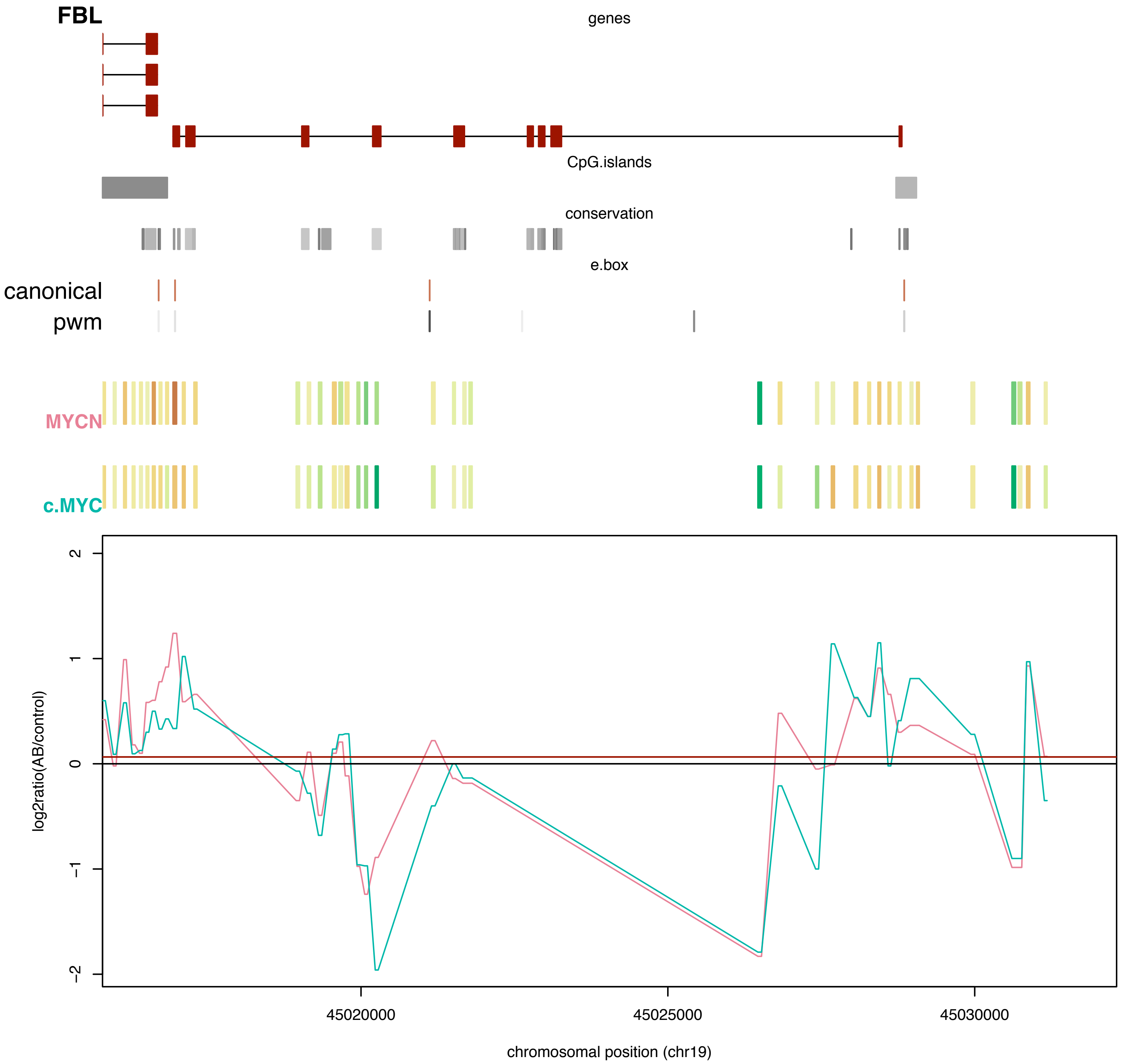
FBL
genes
CpG.islands
conservation
e.box
canonical
pwm
MYCN
c.MYC
log2ratio(AB/control)
2
1
0
−1
−2
45020000
45025000
45030000
chromosomal position (chr19)

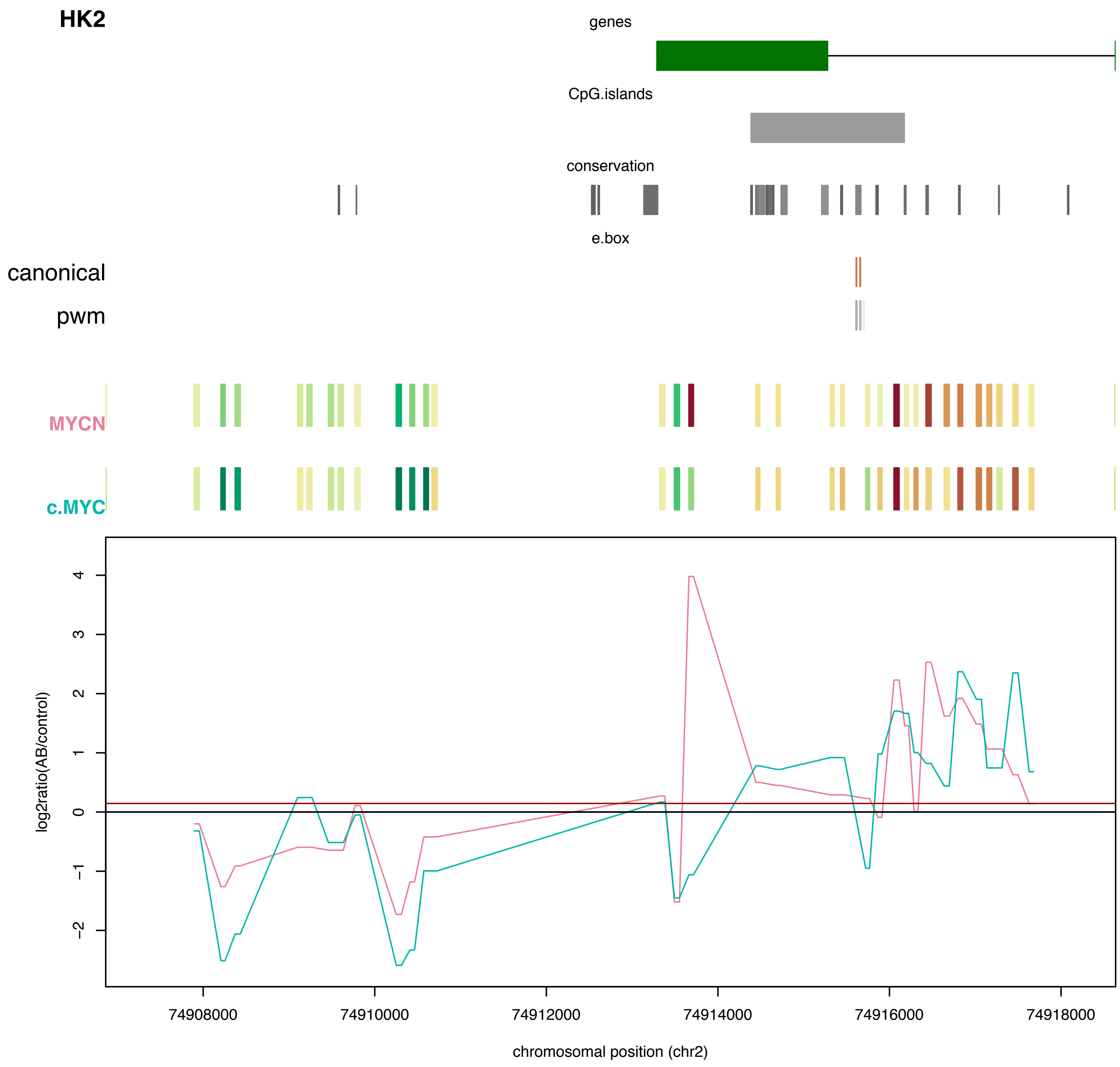
HK2
genes
CpG.islands
conservation
e.box
canonical
pwm
MYCN
c.MYC
log2ratio(AB/control)
4
3
2
1
0
−1
−2
74908000
74910000
74912000
74914000
74916000
74918000
chromosomal position (chr2)

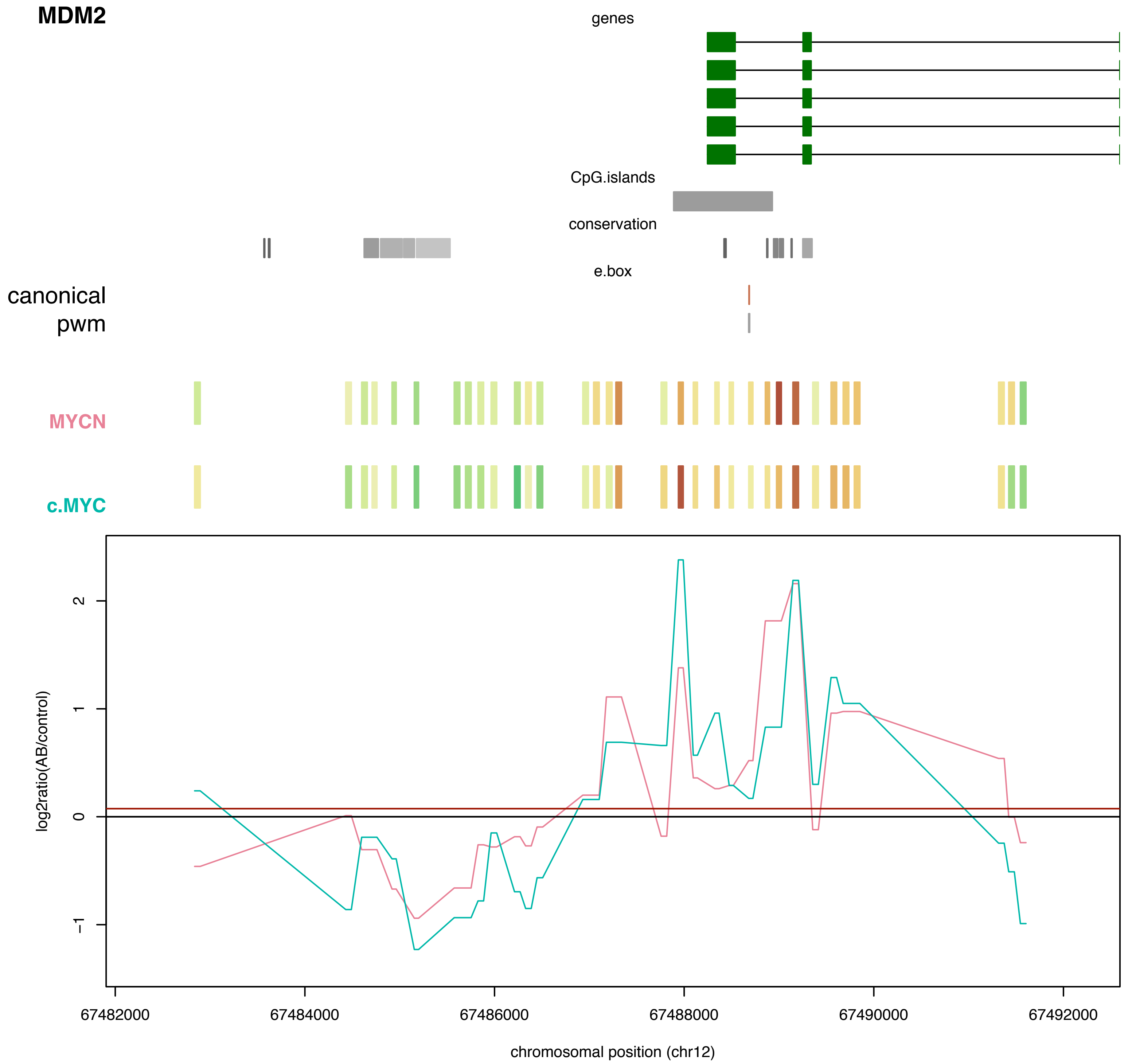
MDM2
genes
CpG.islands
conservation
e.box
canonical
pwm
MYCN
c.MYC
log2ratio(AB/control)
2
1
0
−1
67482000
67484000
67486000
67488000
67490000
67492000
chromosomal position (chr12)

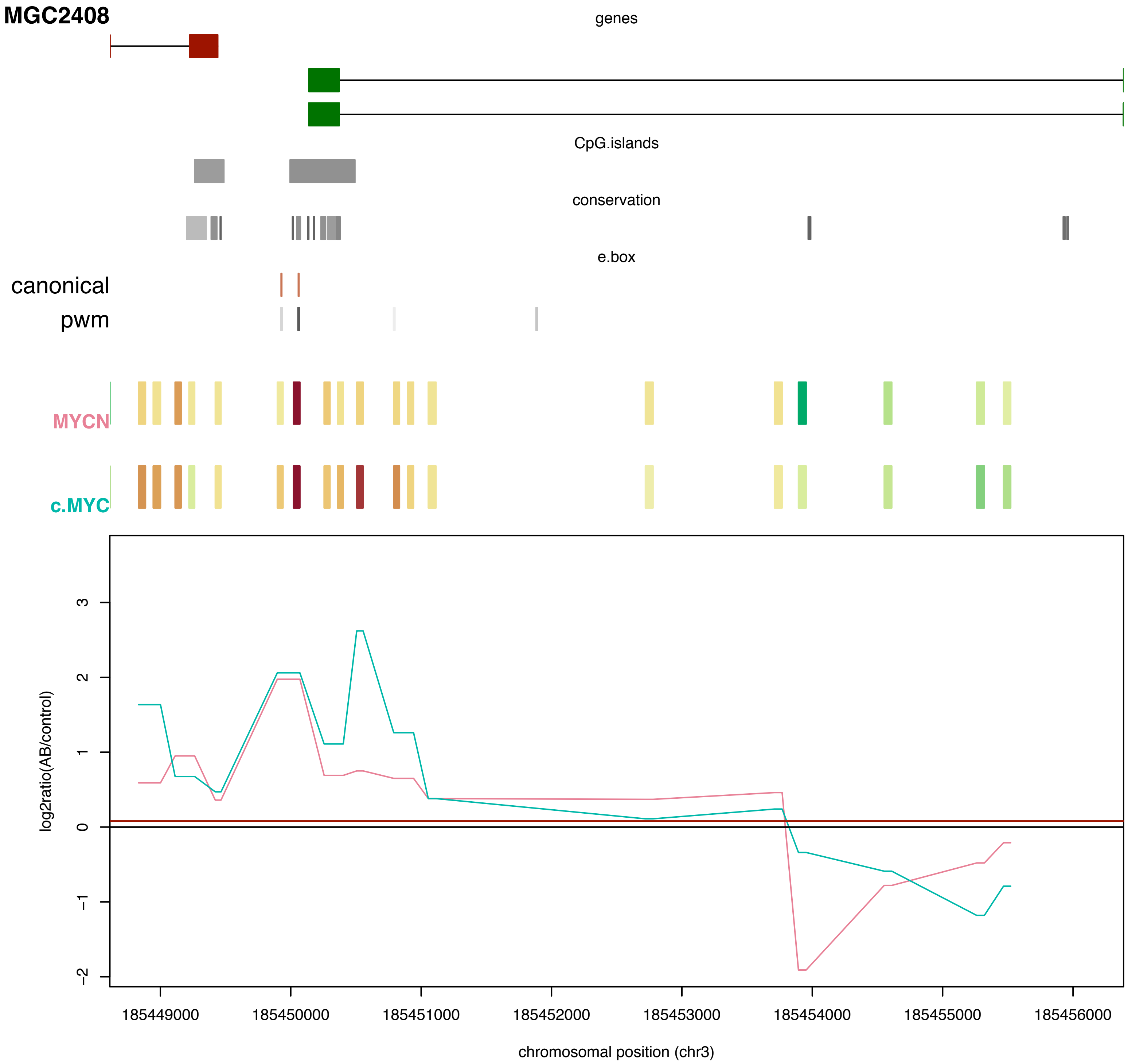

MGC2408
genes
CpG.islands
conservation
e.box
canonical
pwm
MYCN
c.MYC
log2ratio(AB/control)
3
2
1
0
−1
−2
185449000
185450000
185451000
185452000
185453000
185454000
185455000
185456000
chromosomal position (chr3)

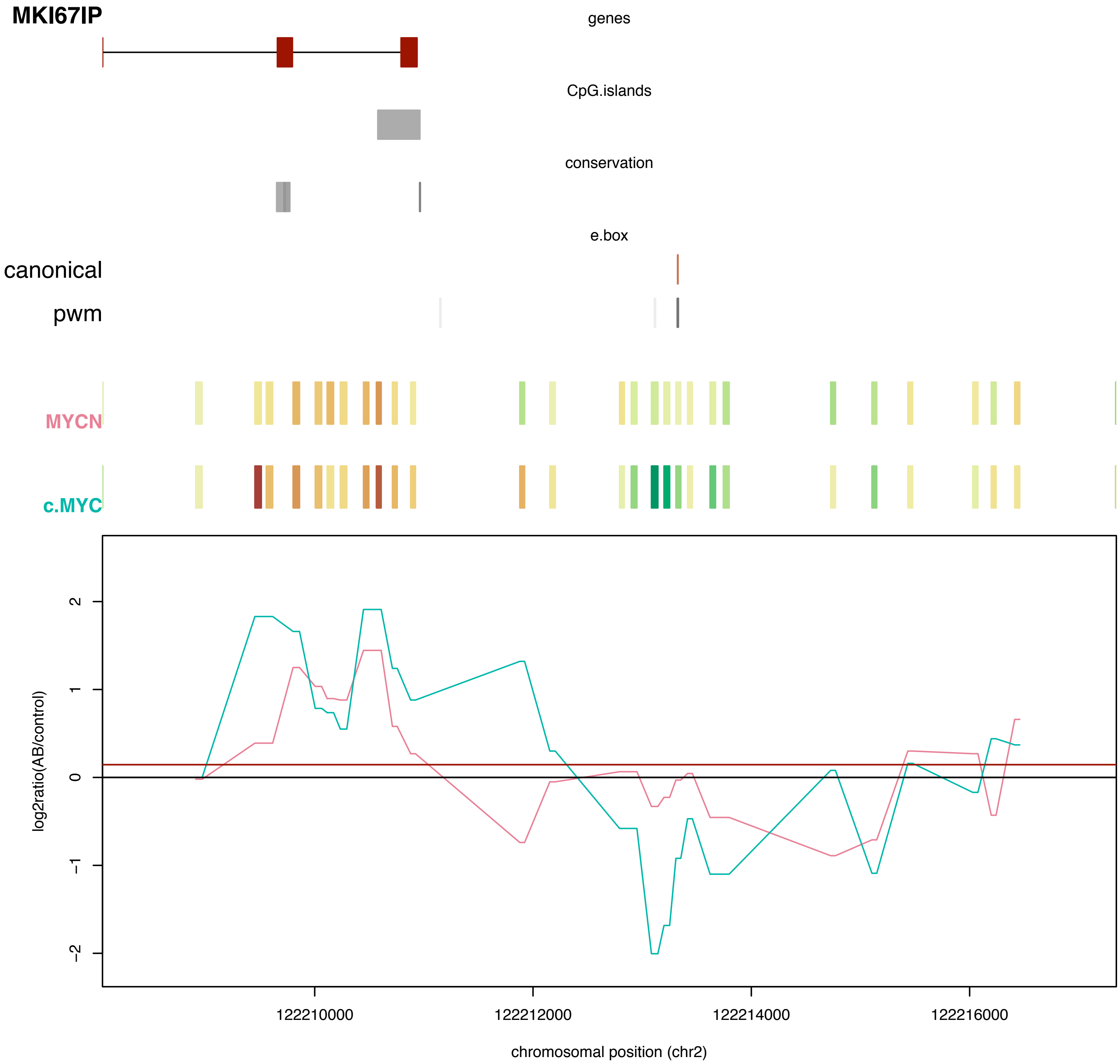

MKI67IP
genes
CpG.islands
conservation
e.box
canonical
pwm
MYCN
c.MYC
log2ratio(AB/control)
2
1
0
−1
−2
122210000
122212000
122214000
122216000
chromosomal position (chr2)

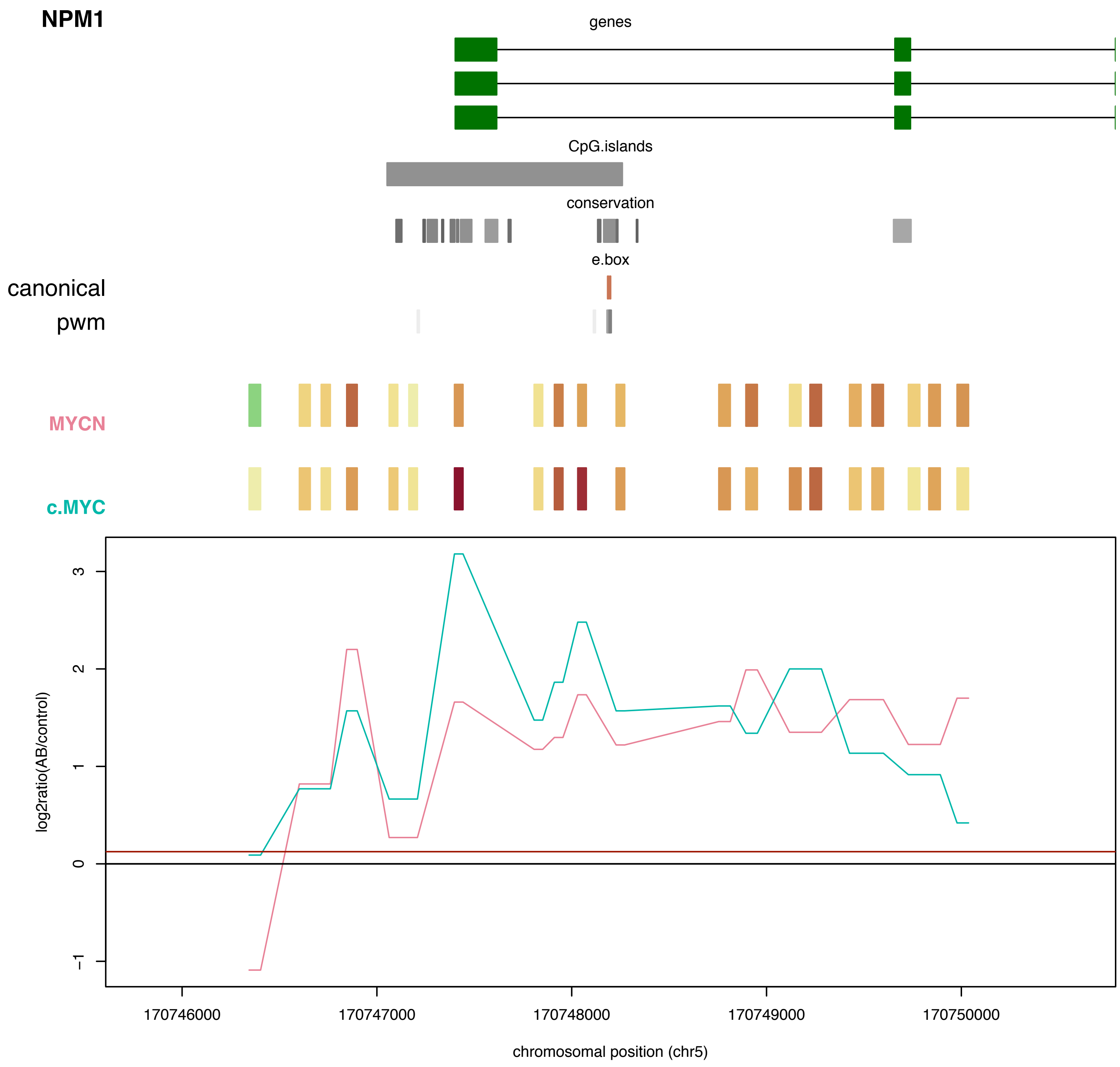

NPM1
genes
CpG.islands
conservation
e.box
canonical
pwm
MYCN
c.MYC
log2ratio(AB/control)
3
2
1
0
−1
170746000
170747000
170748000
170749000
170750000
chromosomal position (chr5)

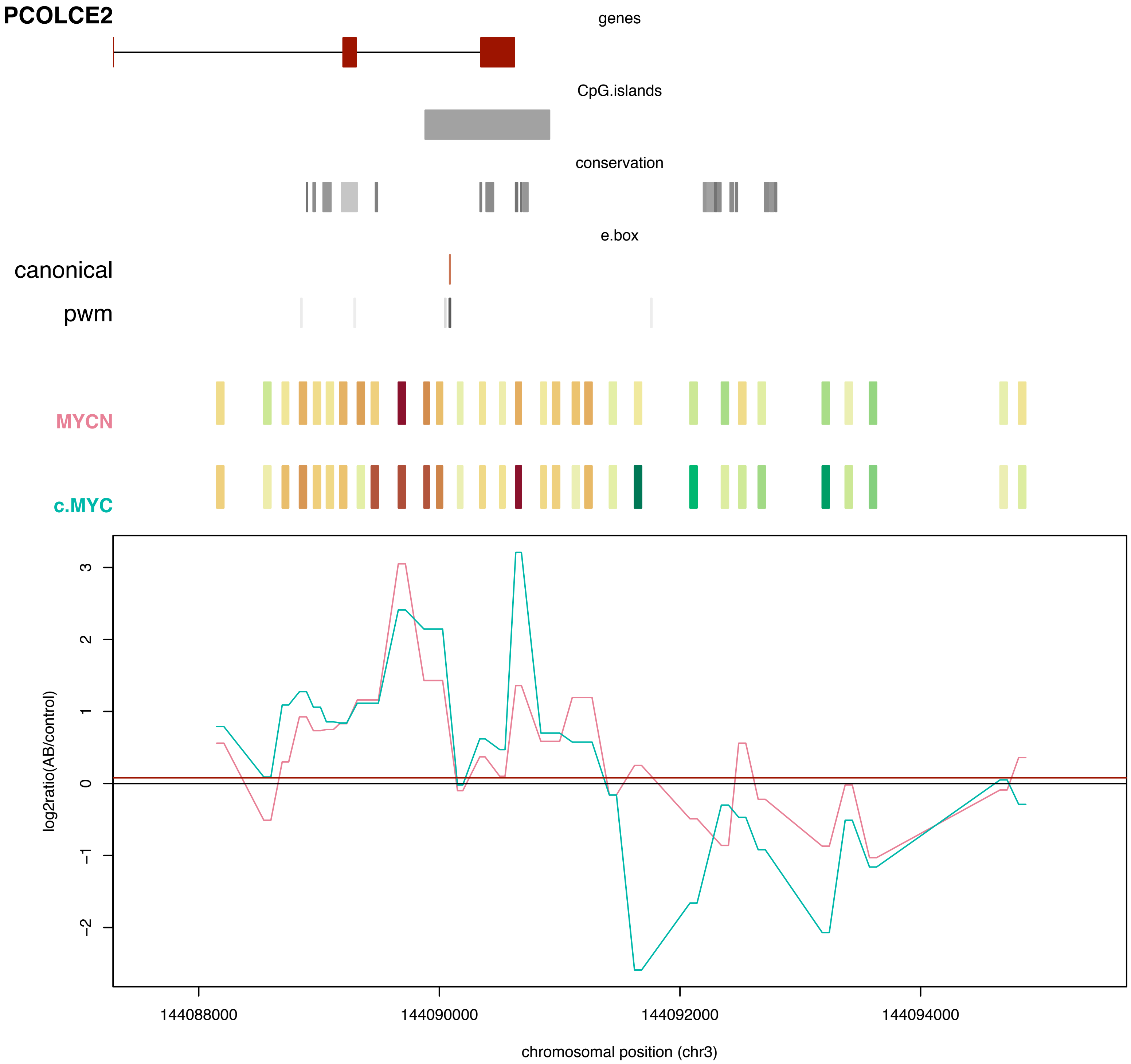
PCOLCE2
genes
CpG.islands
conservation
e.box
canonical
pwm
MYCN
c.MYC
log2ratio(AB/control)
3
2
1
0
−1
−2
144088000
144090000
144092000
144094000
chromosomal position (chr3)

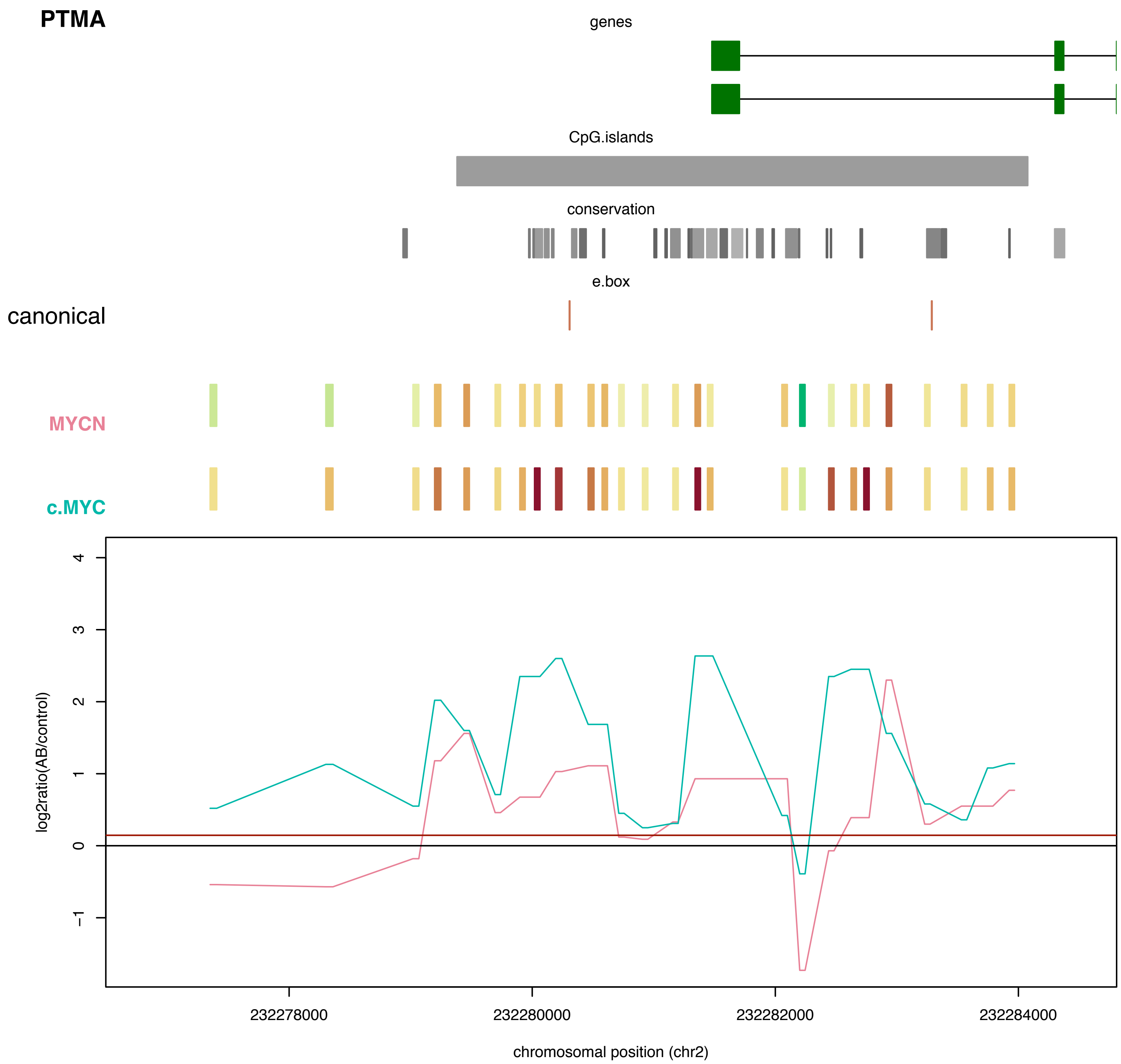

PTMA
genes
CpG.islands
conservation
e.box
canonical
MYCN
c.MYC
log2ratio(AB/control)
4
3
2
1
0
−1
232278000
232280000
232282000
232284000
chromosomal position (chr2)

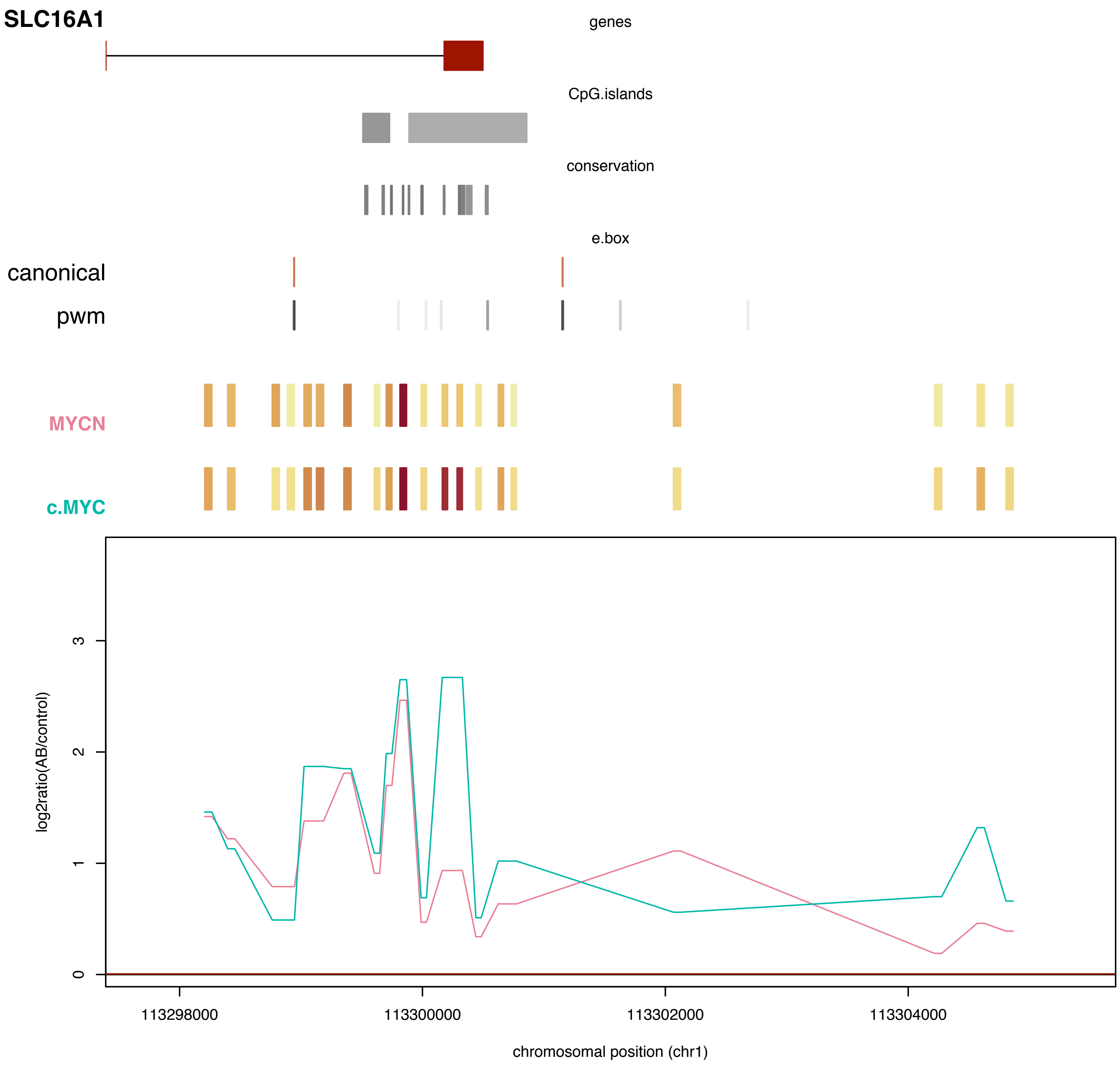
SLC16A1
genes
CpG.islands
conservation
e.box
canonical
pwm
MYCN
c.MYC
log2ratio(AB/control)
0
1
2
3
113298000
113300000
113302000
113304000
chromosomal position (chr1)

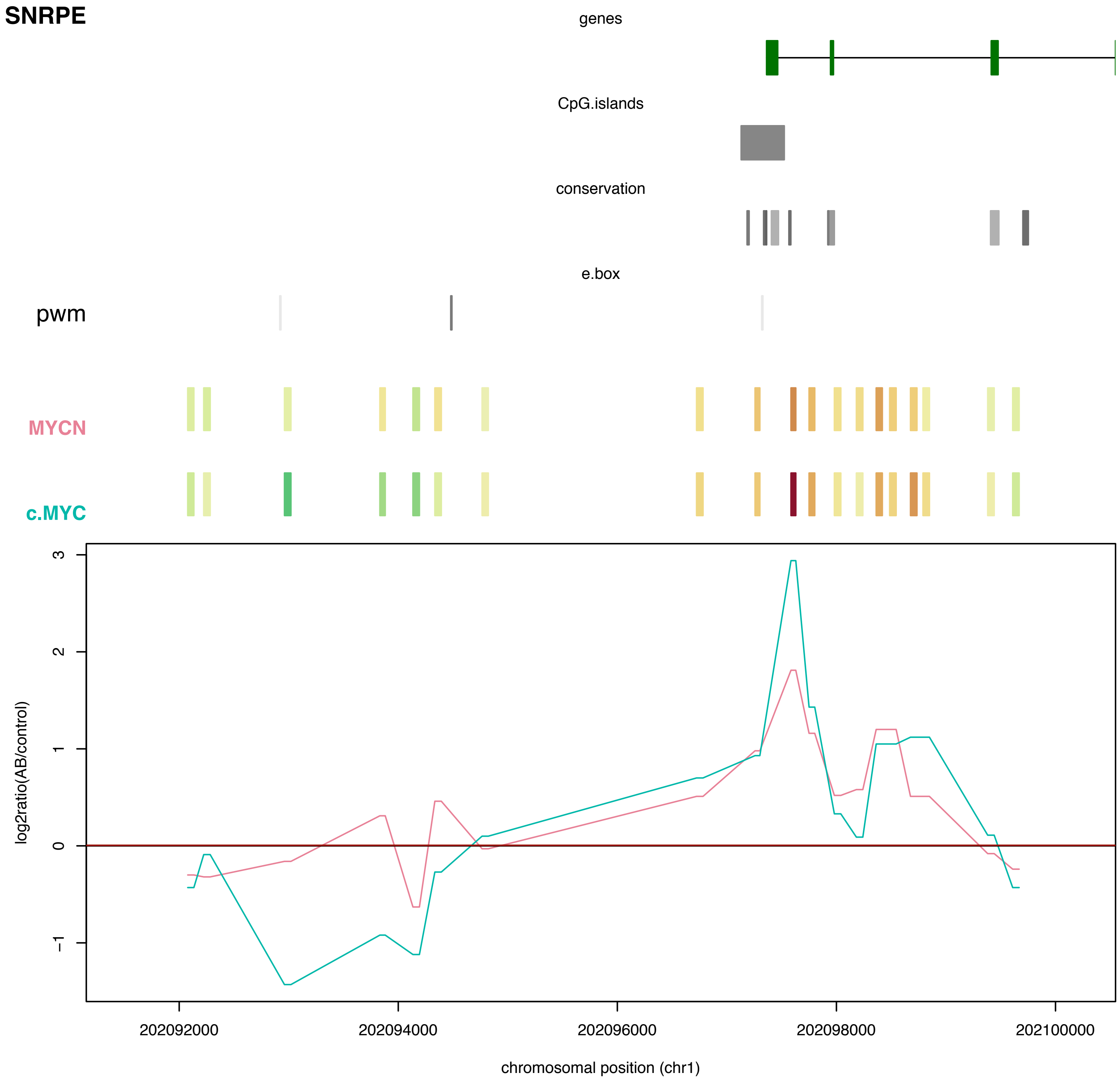

SNRPE
genes
CpG.islands
conservation
e.box
pwm
MYCN
c.MYC
log2ratio(AB/control)
3
2
1
0
−1
202092000
202094000
202096000
202098000
202100000
chromosomal position (chr1)

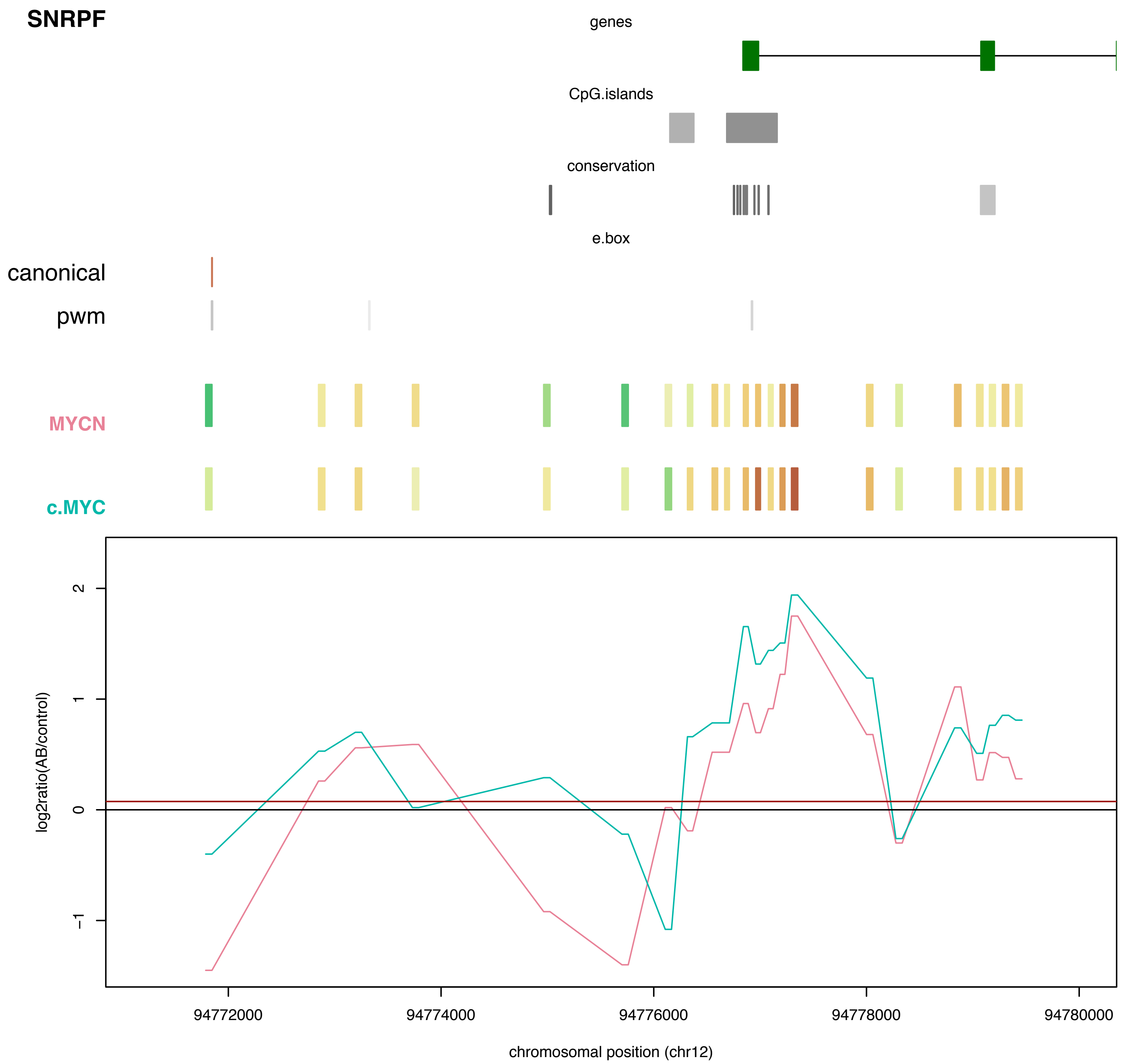
SNRPF
genes
CpG.islands
conservation
e.box
canonical
pwm
MYCN
c.MYC
log2ratio(AB/control)
2
1
0
−1
94772000
94774000
94776000
94778000
94780000
chromosomal position (chr12)

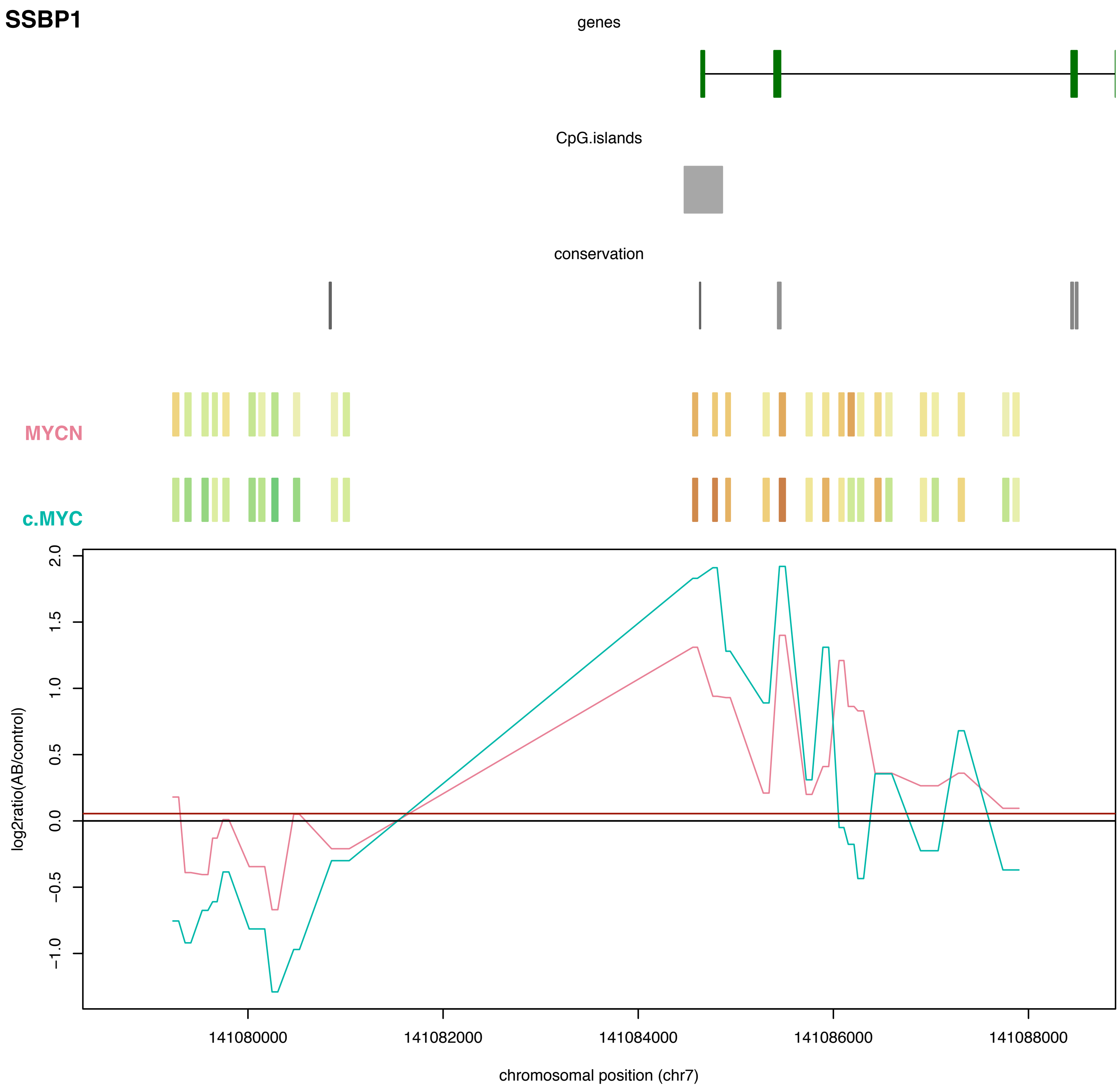

SSBP1
genes
CpG.islands
conservation
MYCN
c.MYC
log2ratio(AB/control)
2.0
1.5
1.0
0.5
0.0
−0.5
−1.0
141080000
141082000
141084000
141086000
141088000
chromosomal position (chr7)

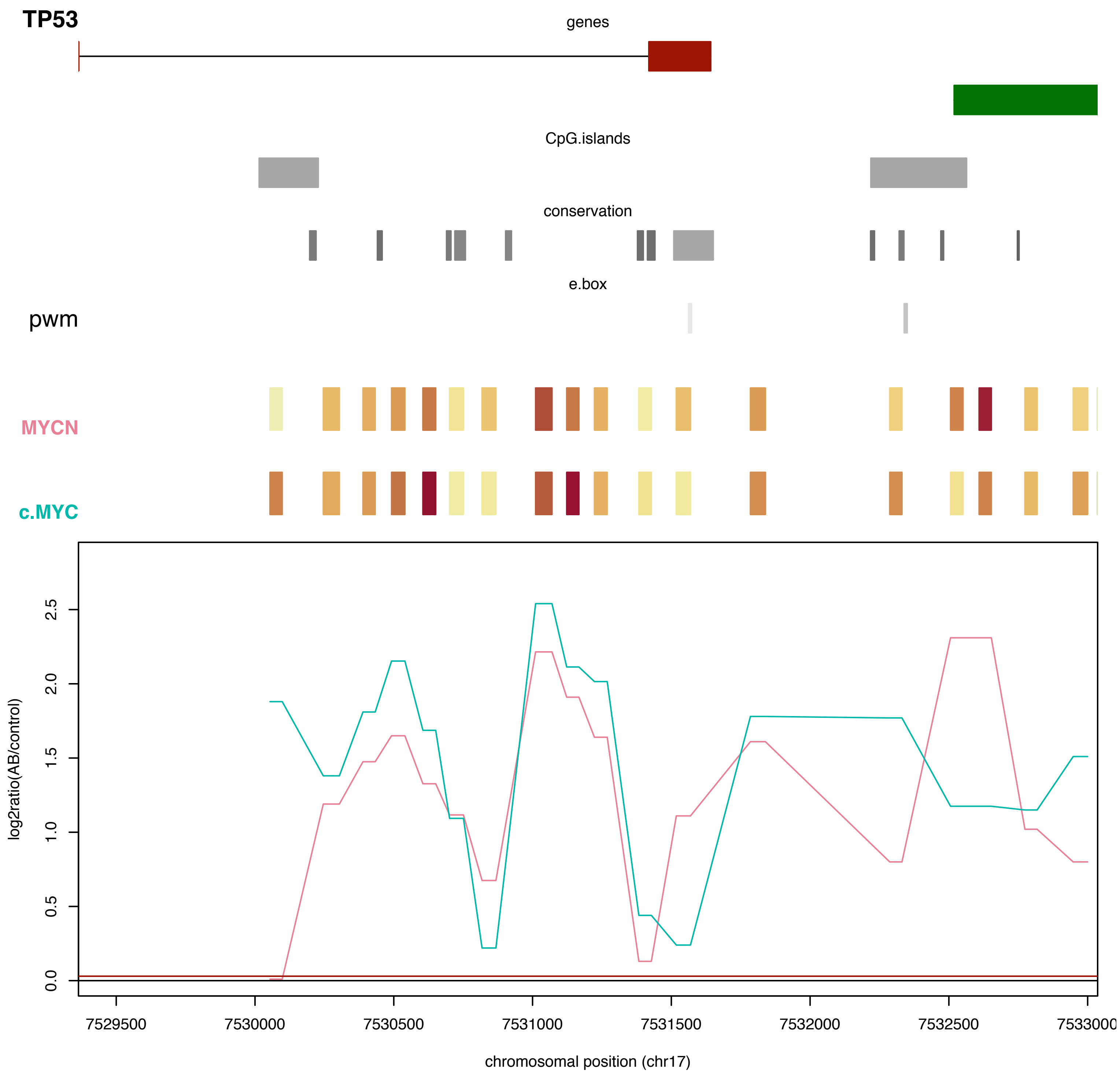
TP53
genes
CpG.islands
conservation
e.box
pwm
MYCN
c.MYC
log2ratio(AB/control)
0.0
0.5
1.0
1.5
2.0
2.5
7529500
7530000
7530500
7531000
7531500
7532000
7532500
7533000
chromosomal position (chr17)

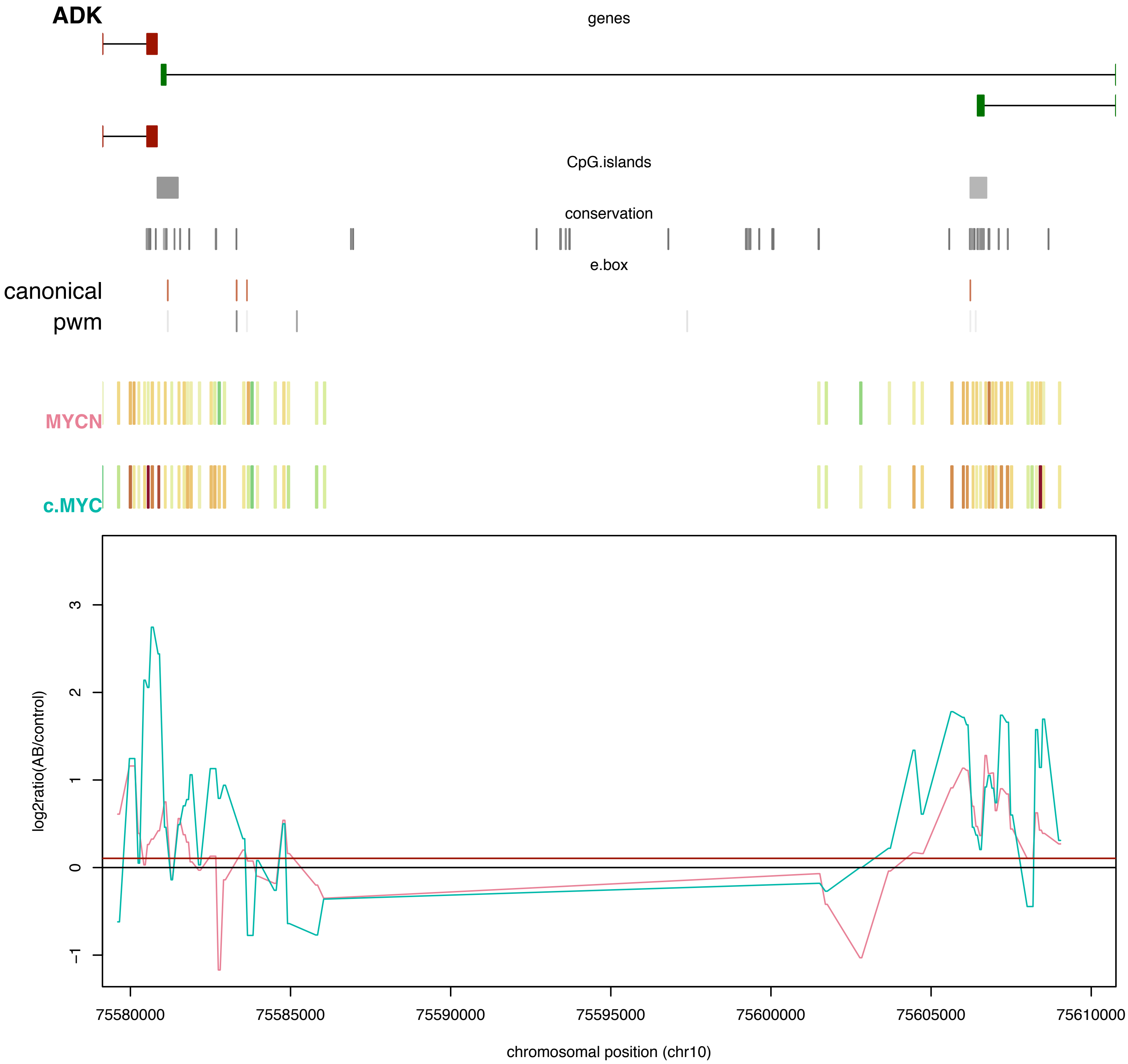

ADK
genes
CpG.islands
conservation
e.box
canonical
pwm
MYCN
c.MYC
log2ratio(AB/control)
−1
0
1
2
3
75580000
75585000
75590000
75595000
75600000
75605000
75610000
chromosomal position (chr10)

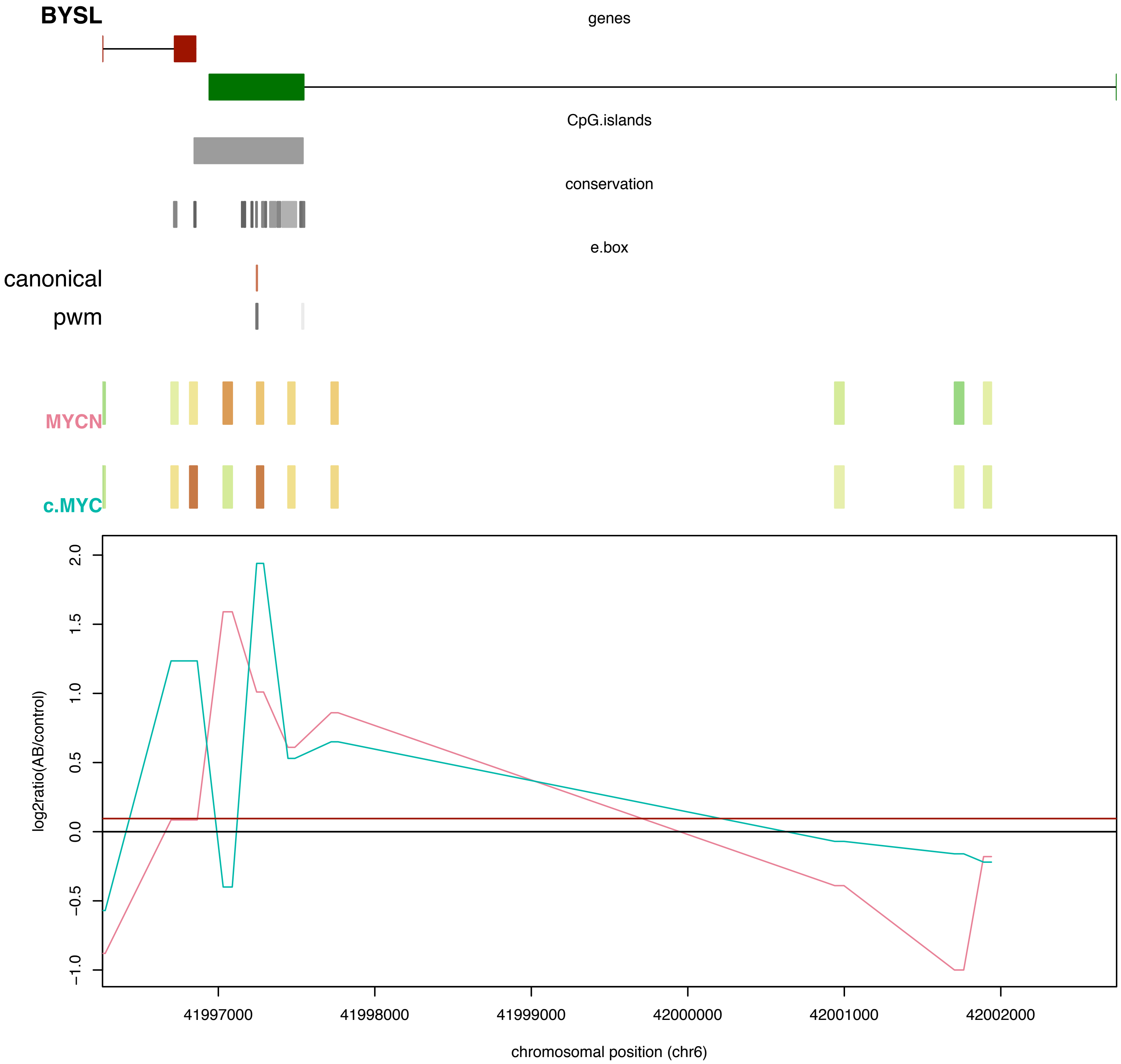
BYSL
genes
CpG.islands
conservation
e.box
canonical
pwm
MYCN
c.MYC
log2ratio(AB/control)
2.0
1.5
1.0
0.5
0.0
−0.5
−1.0
41997000
41998000
41999000
42000000
42001000
42002000
chromosomal position (chr6)

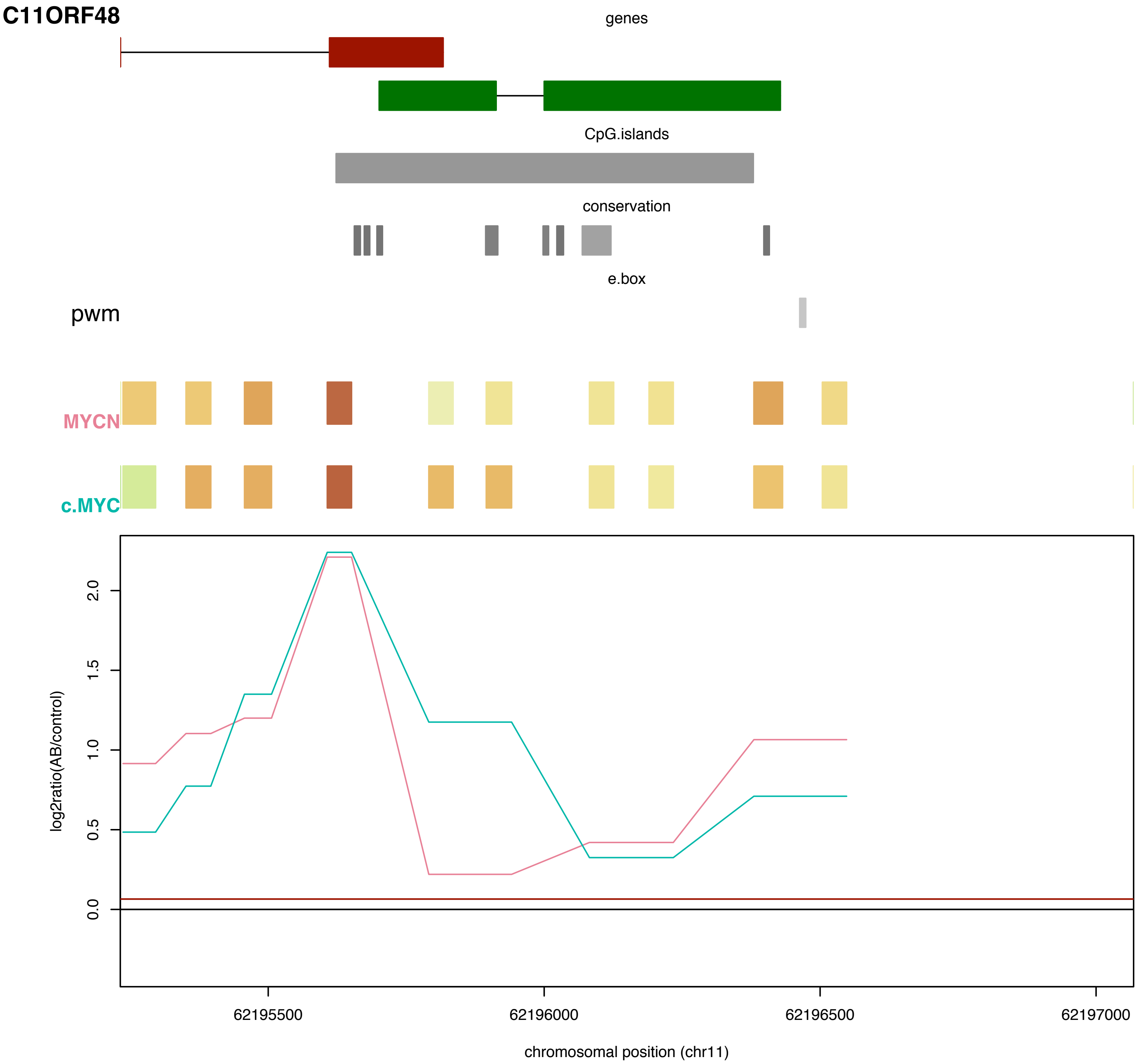

C11ORF48
genes
CpG.islands
conservation
e.box
pwm
MYCN
c.MYC
log2ratio(AB/control)
0.0
0.5
1.0
1.5
2.0
62195500
62196000
62196500
62197000
chromosomal position (chr11)

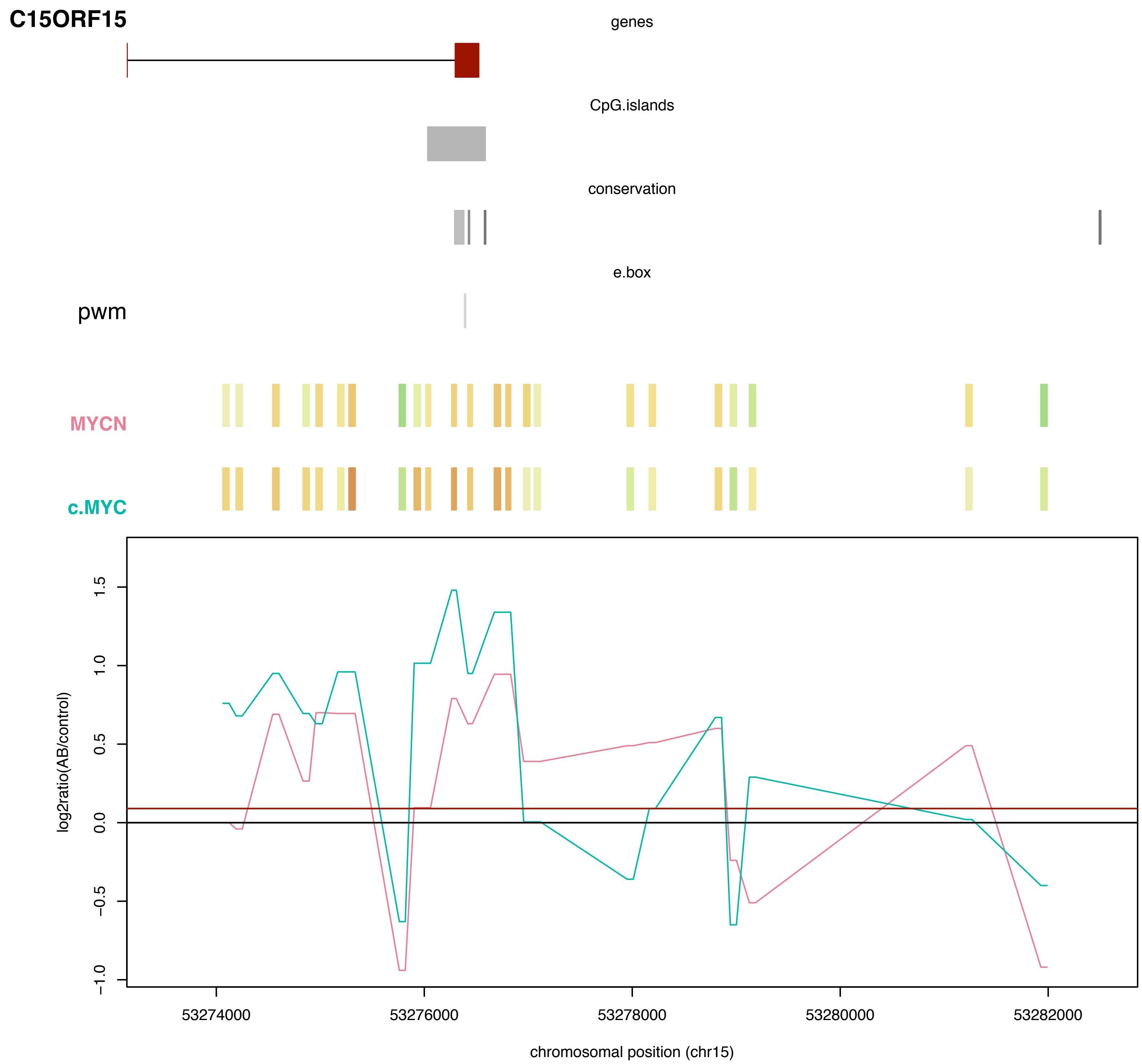

C15ORF15
genes
CpG.islands
conservation
e.box
pwm
MYCN
c.MYC
log2ratio(AB/control)
1.5
1.0
0.5
0.0
−0.5
−1.0
53274000
53276000
53278000
53280000
53282000
chromosomal position (chr15)

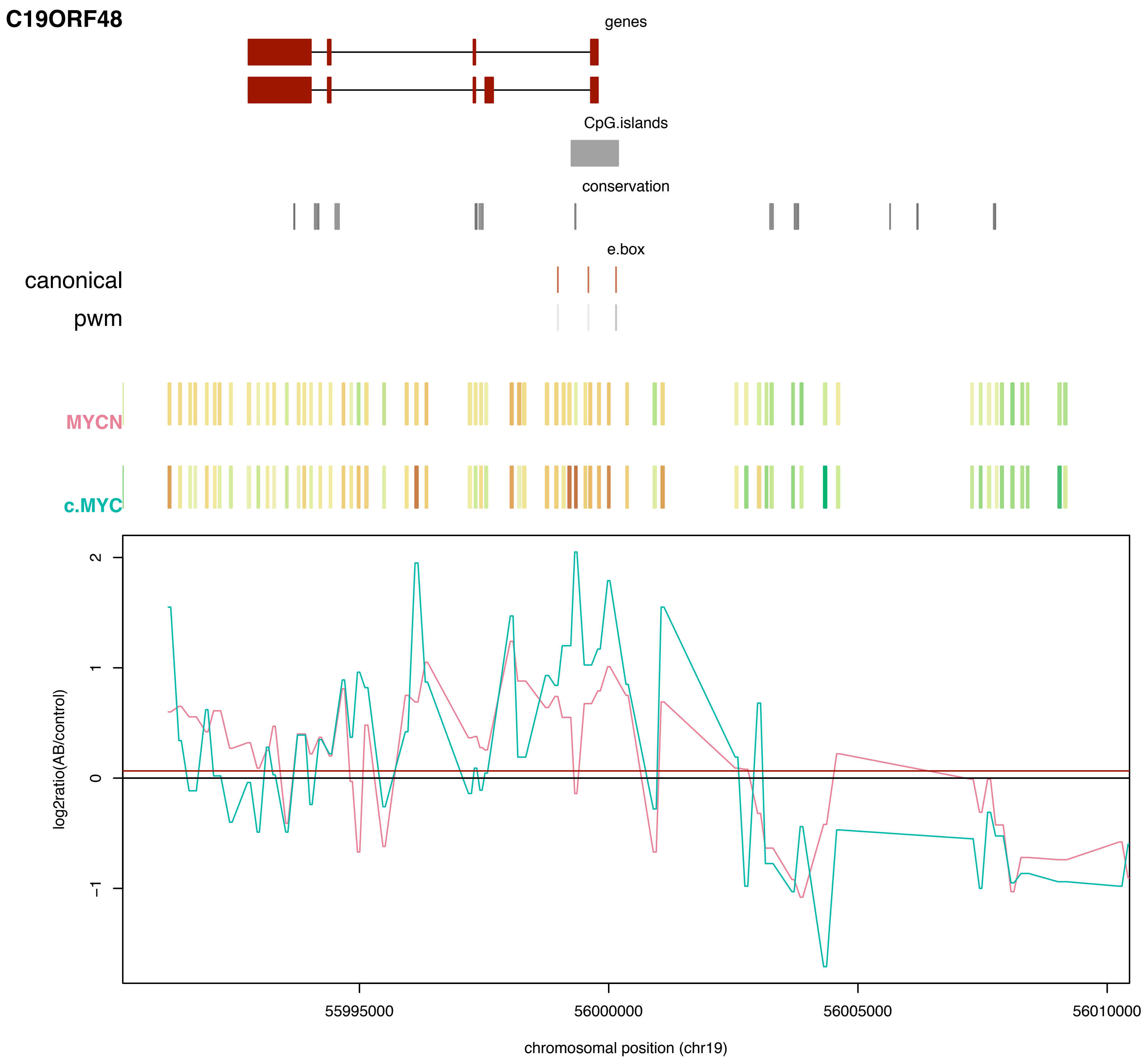

C19ORF48
genes
CpG.islands
conservation
e.box
canonical
pwm
MYCN
c.MYC
log2ratio(AB/control)
2
1
0
−1
55995000
56000000
56005000
56010000
chromosomal position (chr19)

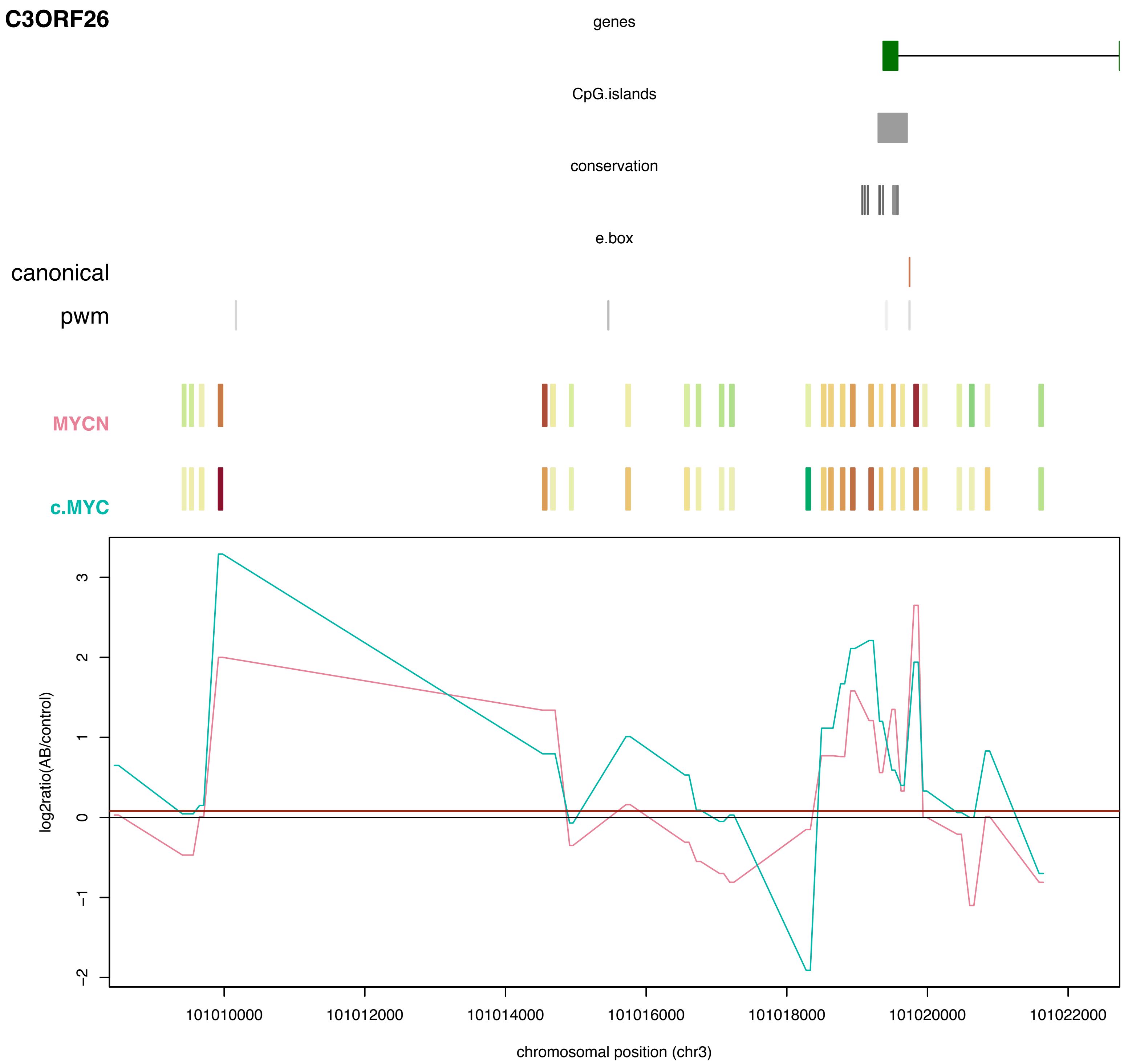

C3ORF26
genes
CpG.islands
conservation
e.box
canonical
pwm
MYCN
c.MYC
log2ratio(AB/control)
3
2
1
0
−1
−2
101010000
101012000
101014000
101016000
101018000
101020000
101022000
chromosomal position (chr3)

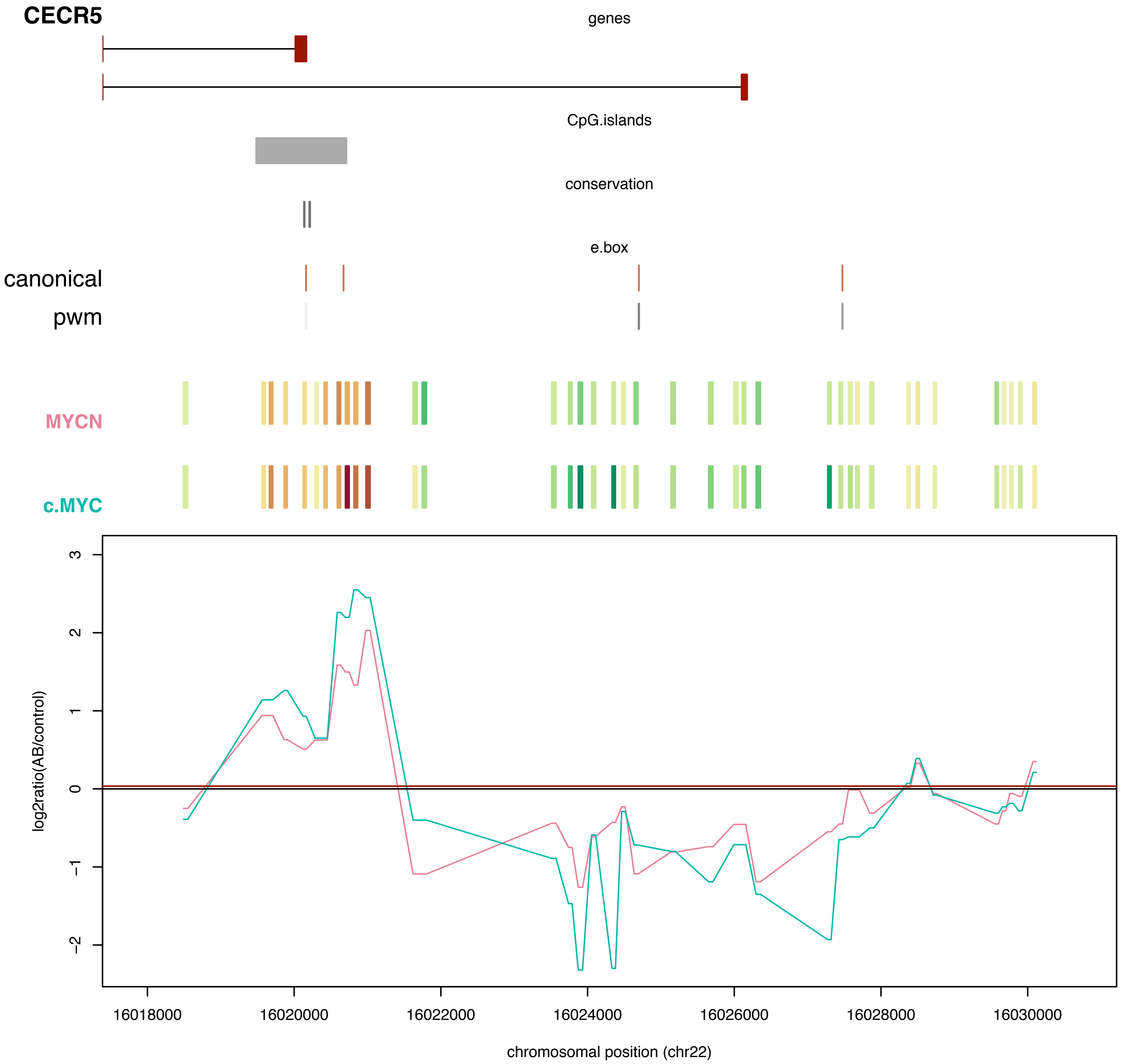
CECR5
genes
CpG.islands
conservation
e.box
canonical
pwm
MYCN
c.MYC
log2ratio(AB/control)
3
2
1
0
−1
−2
16018000
16020000
16022000
16024000
16026000
16028000
16030000
chromosomal position (chr22)

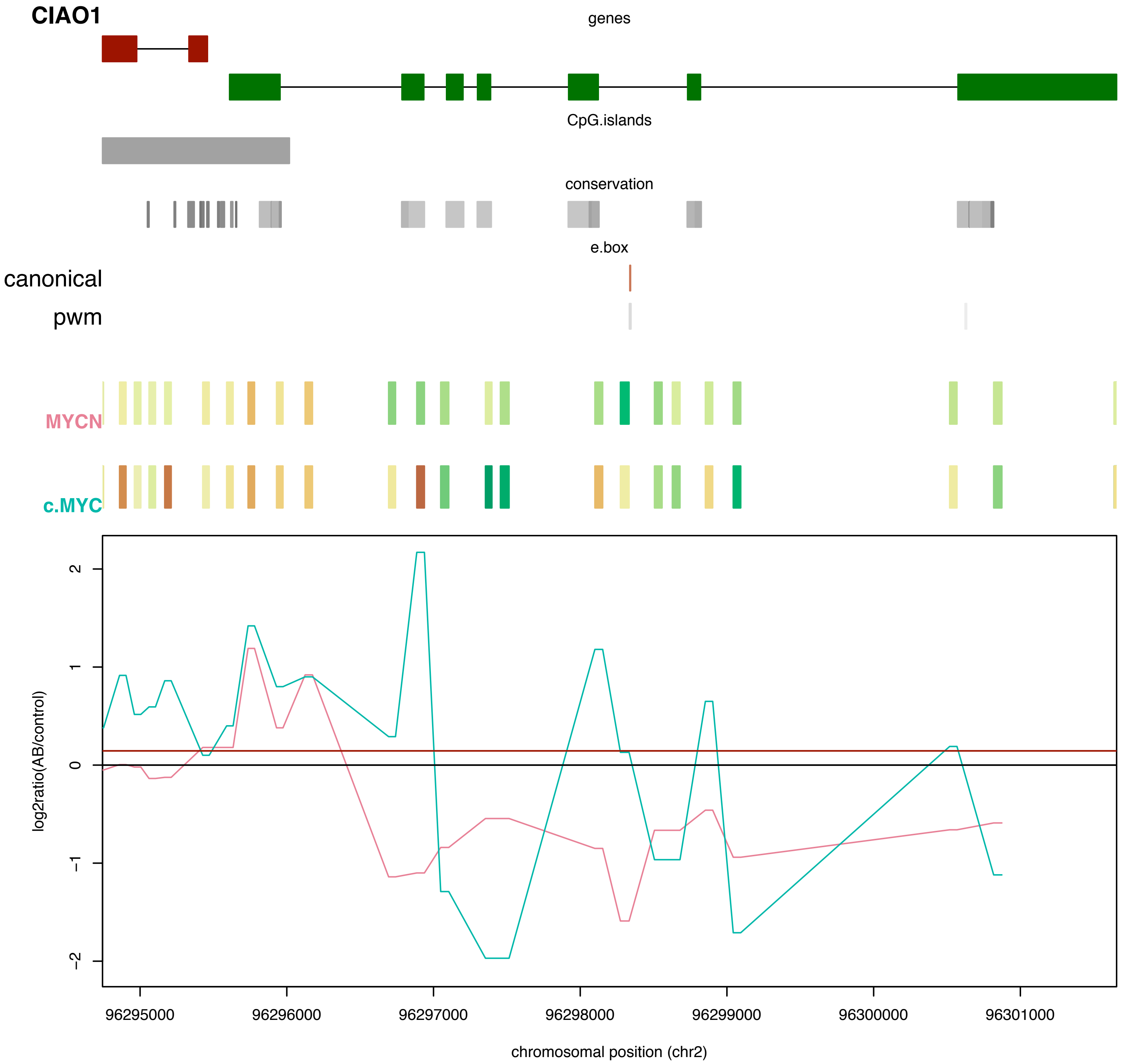

CIAO1
genes
CpG.islands
conservation
e.box
canonical
pwm
MYCN
c.MYC
log2ratio(AB/control)
2
1
0
−1
−2
96295000
96296000
96297000
96298000
96299000
96300000
96301000
chromosomal position (chr2)

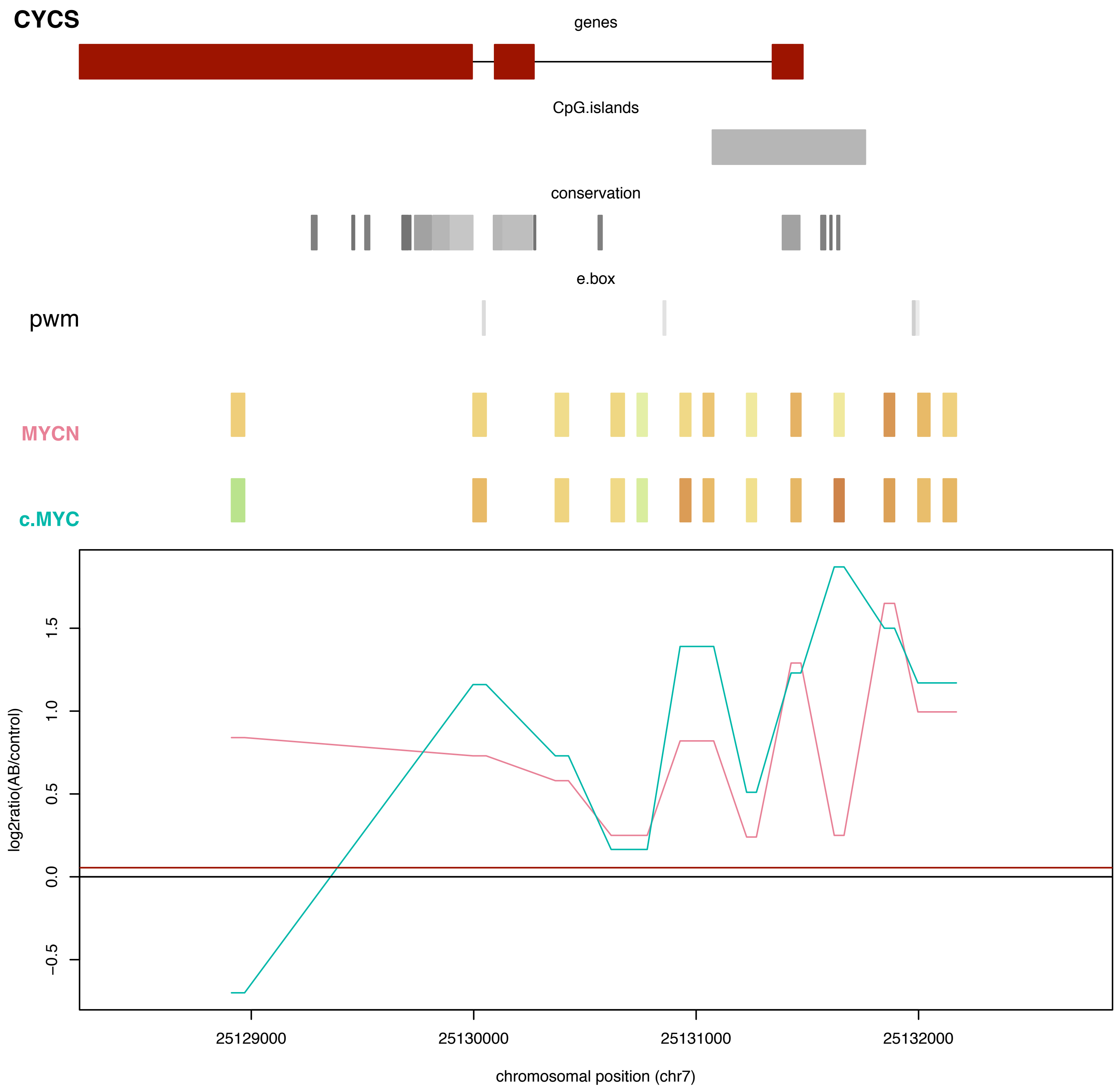
CYCS
genes
CpG.islands
conservation
e.box
pwm
MYCN
c.MYC
log2ratio(AB/control)
−0.5
0.0
0.5
1.0
1.5
25129000
25130000
25131000
25132000
chromosomal position (chr7)

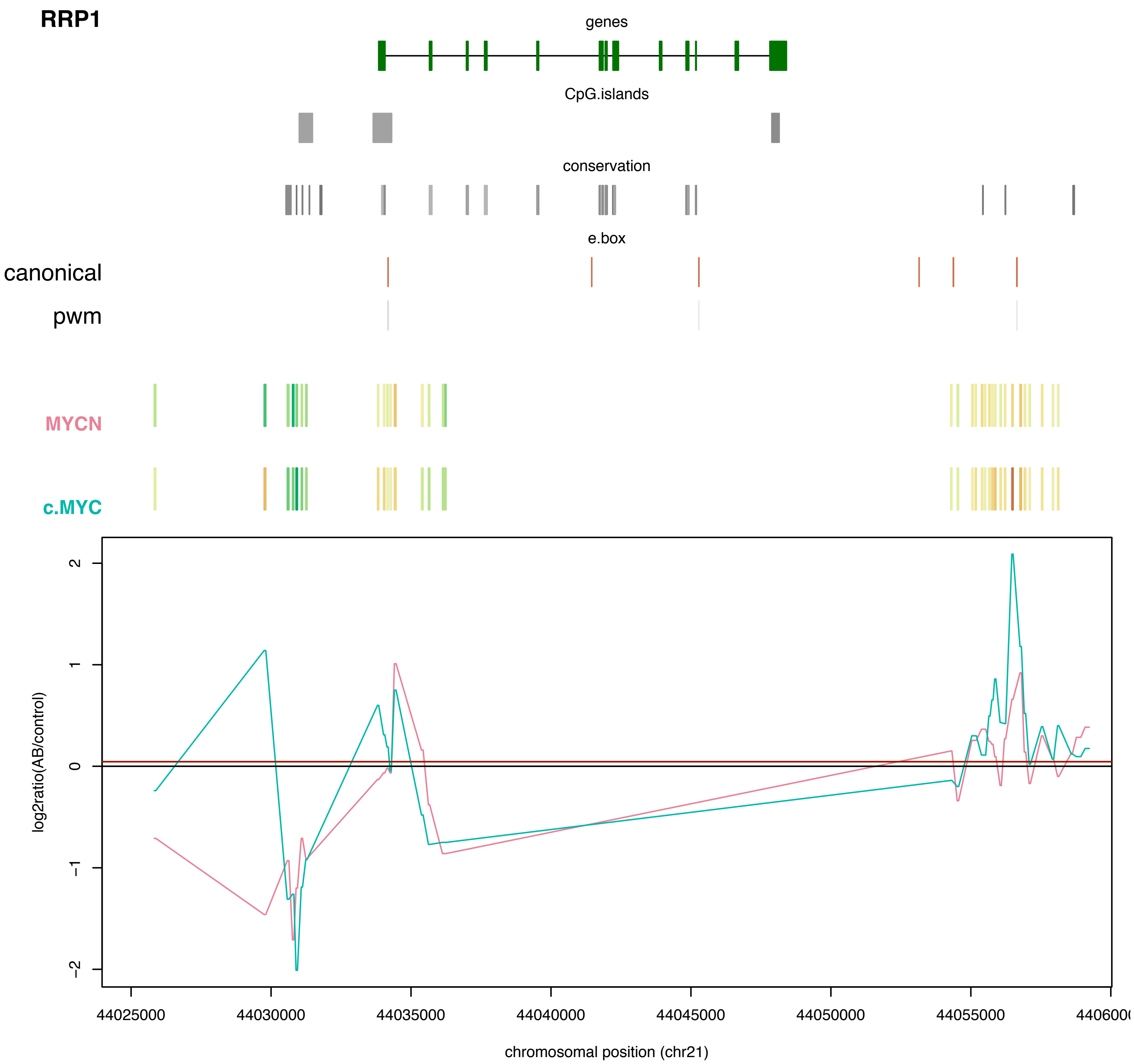

RRP1
genes
CpG.islands
conservation
e.box
canonical
pwm
MYCN
c.MYC
log2ratio(AB/control)
2
1
0
−1
−2
44025000
44030000
44035000
44040000
44045000
44050000
44055000
chromosomal position (chr21)

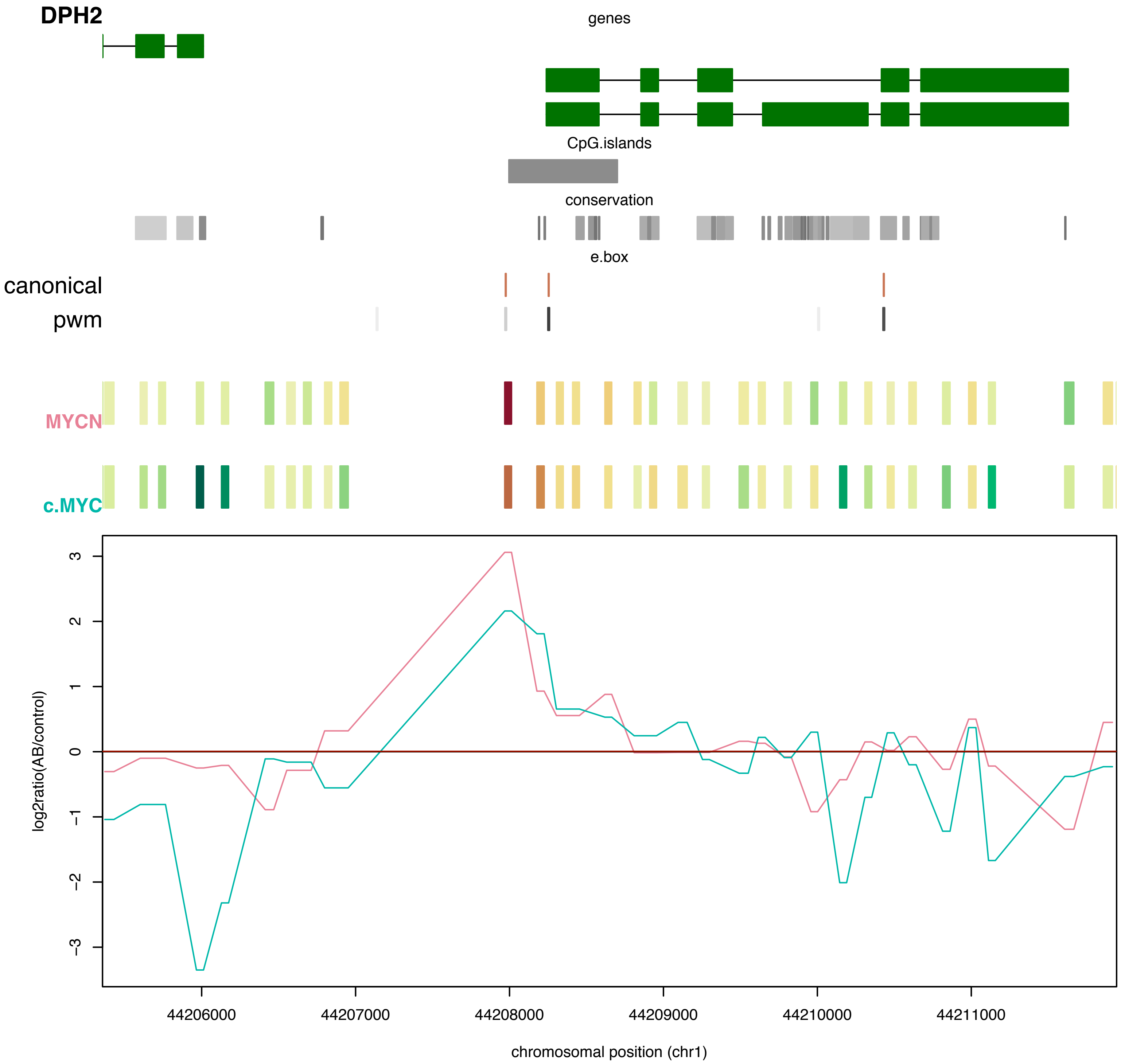

DPH2
genes
CpG.islands
conservation
e.box
canonical
pwm
MYCN
c.MYC
log2ratio(AB/control)
3
2
1
0
−1
−2
−3
44206000
44207000
44208000
44209000
44210000
44211000
chromosomal position (chr1)

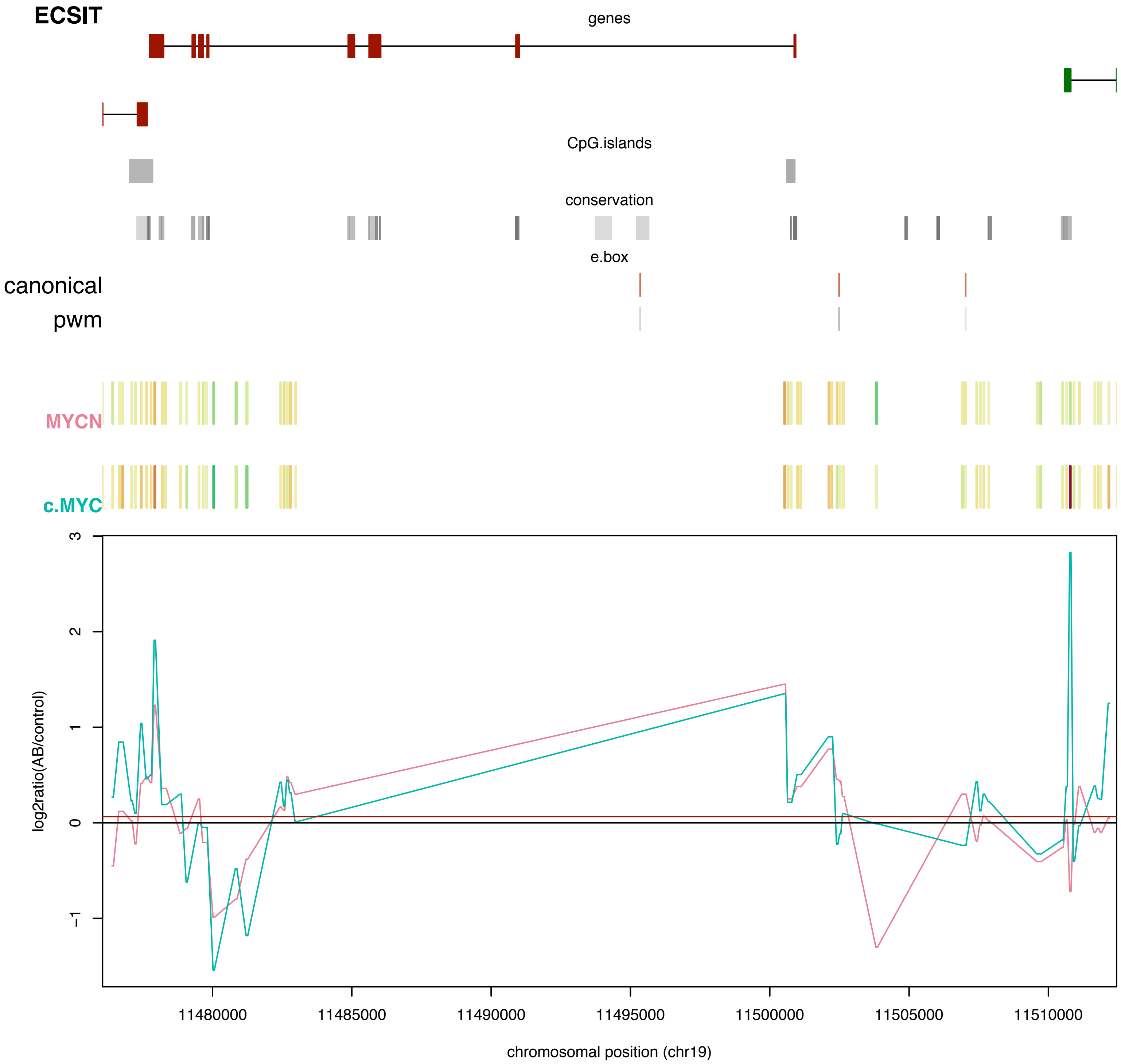

ECSIT
genes
CpG.islands
conservation
e.box
canonical
pwm
MYCN
c.MYC
3
2
1
0
−1
log2ratio(AB/control)
11480000
11485000
11490000
11495000
11500000
11505000
11510000
chromosomal position (chr19)

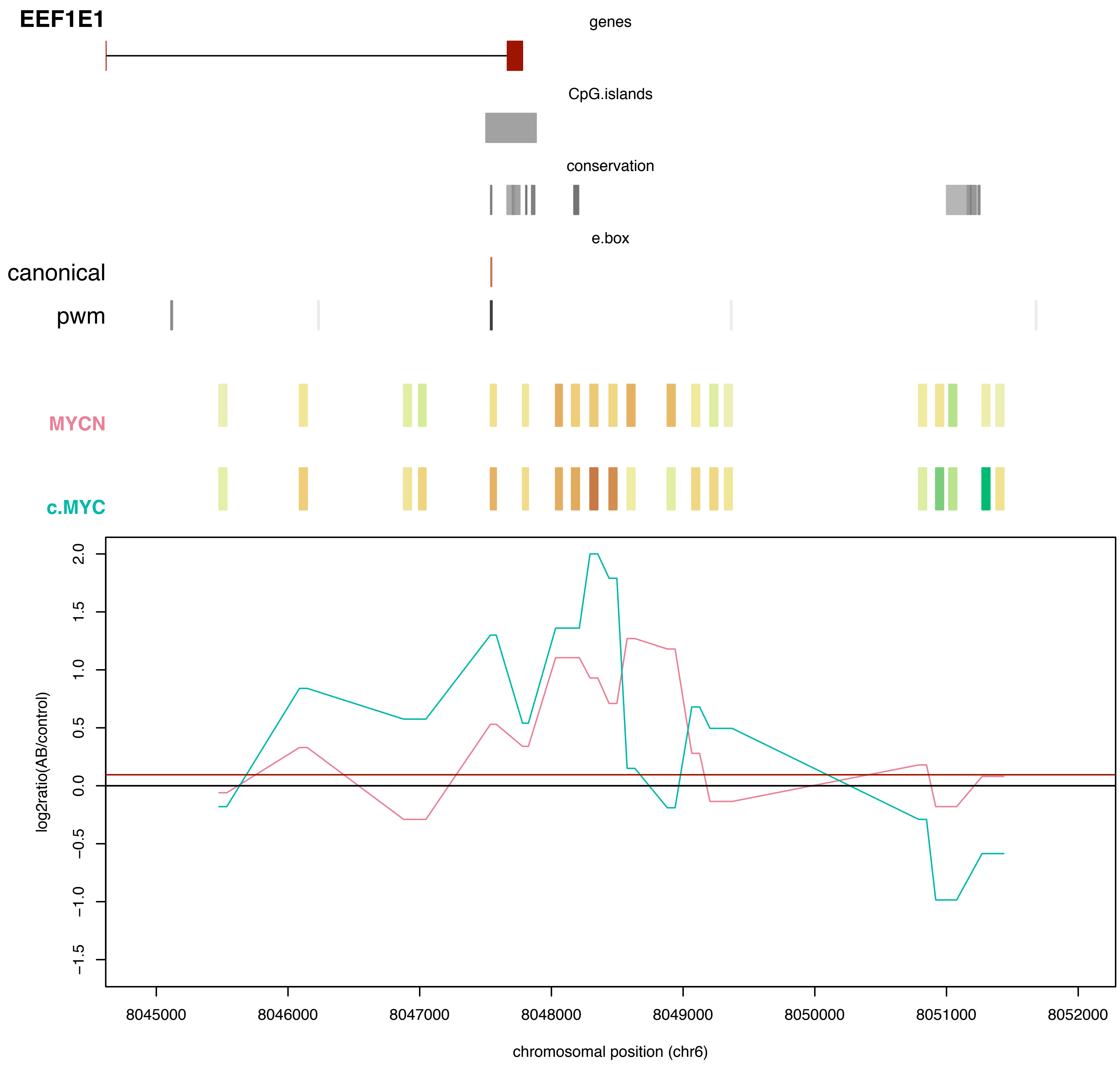

EEF1E1
genes
CpG.islands
conservation
e.box
canonical
pwm
MYCN
c.MYC
log2ratio(AB/control)
2.0
1.5
1.0
0.5
0.0
−0.5
−1.0
−1.5
8045000
8046000
8047000
8048000
8049000
8050000
8051000
8052000
chromosomal position (chr6)

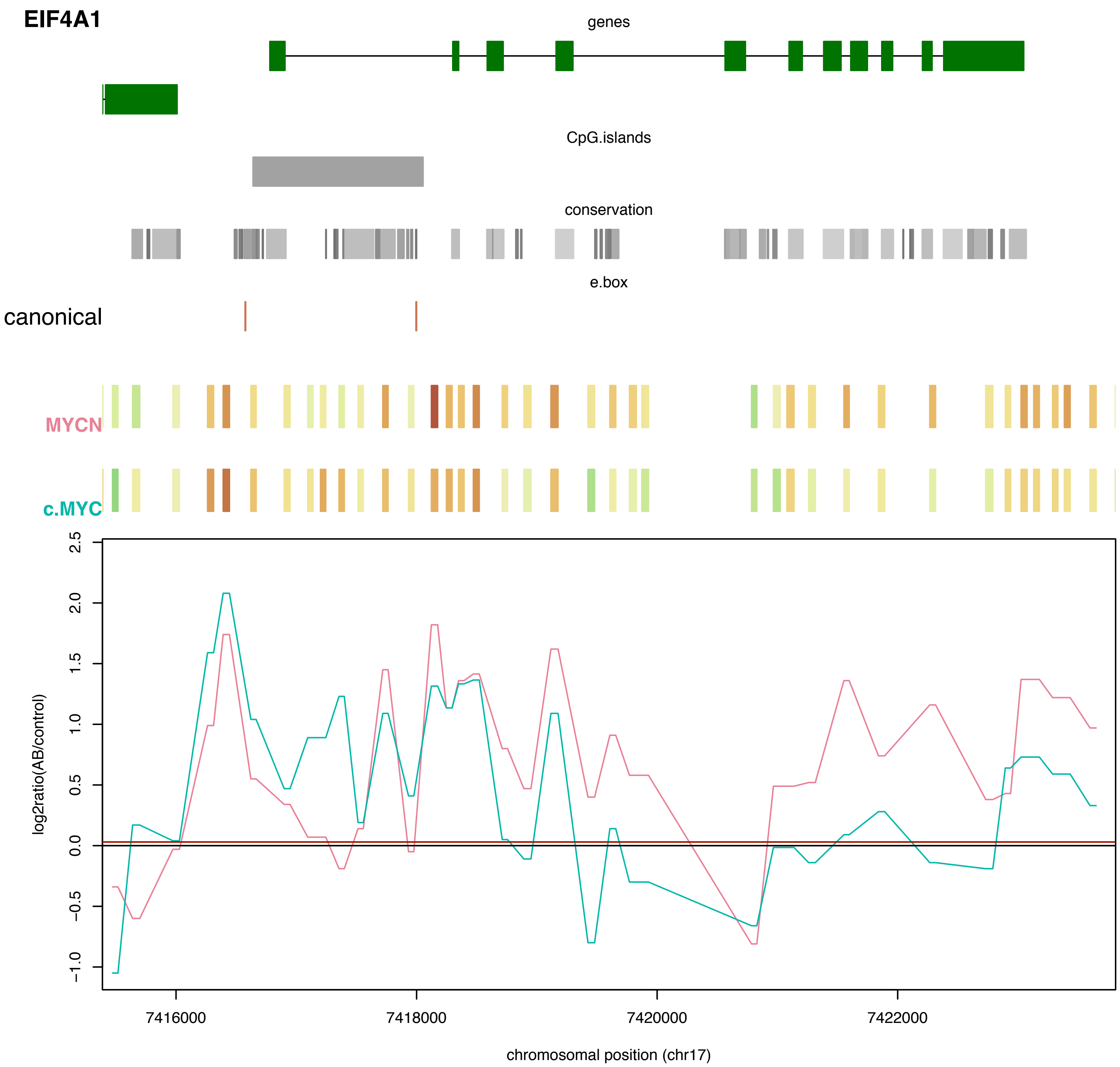
EIF4A1
genes
CpG.islands
conservation
e.box
canonical
MYCN
c.MYC
log2ratio(AB/control)
2.5
2.0
1.5
1.0
0.5
0.0
−0.5
−1.0
7416000
7418000
7420000
7422000
chromosomal position (chr17)

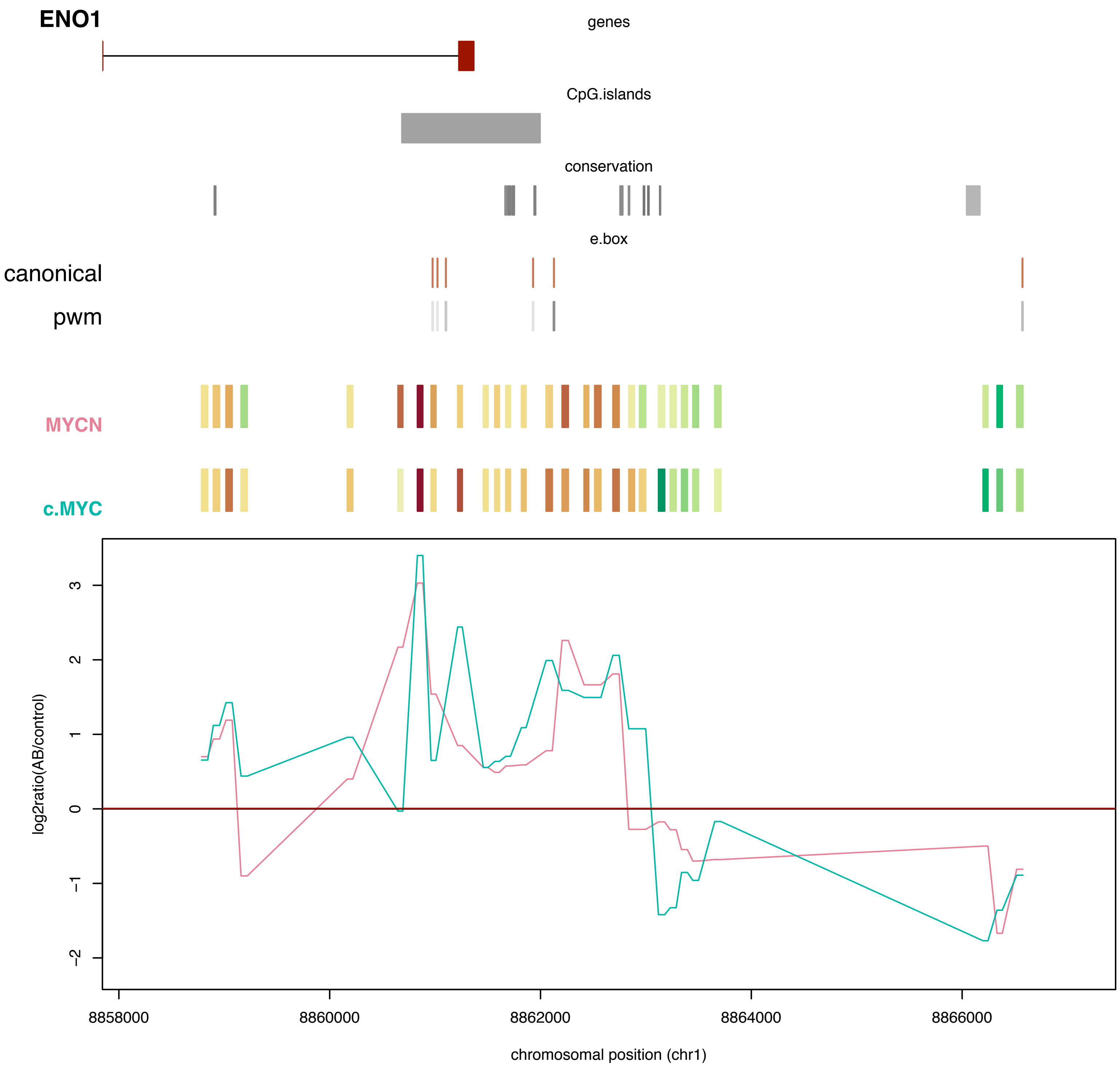
ENO1
genes
CpG.islands
conservation
e.box
canonical
pwm
MYCN
c.MYC
log2ratio(AB/control)
3
2
1
0
−1
−2
8858000
8860000
8862000
8864000
8866000
chromosomal position (chr1)

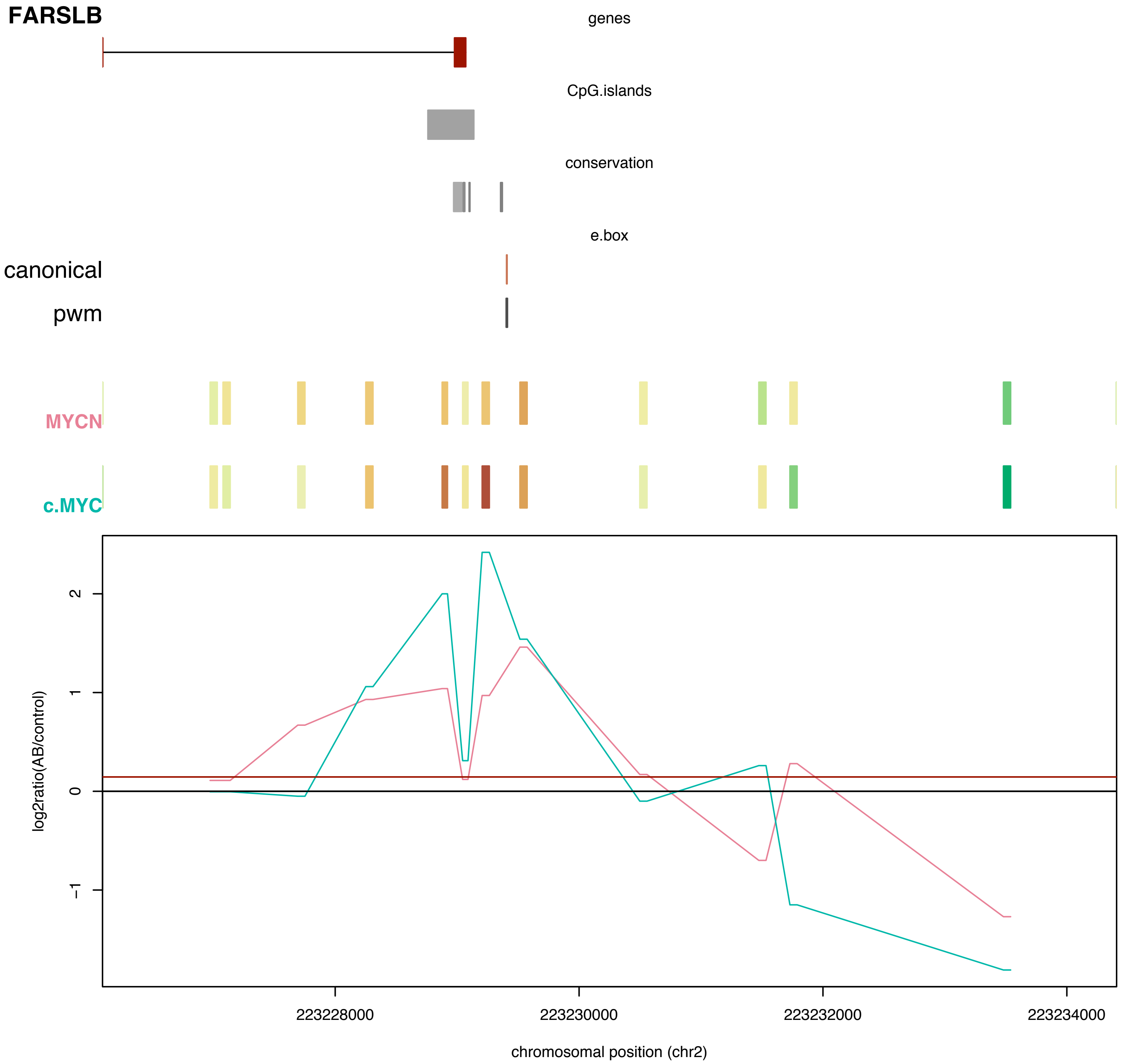

FARSLB
genes
CpG.islands
conservation
e.box
canonical
pwm
MYCN
c.MYC
log2ratio(AB/control)
2
1
0
−1
223228000
223230000
223232000
223234000
chromosomal position (chr2)

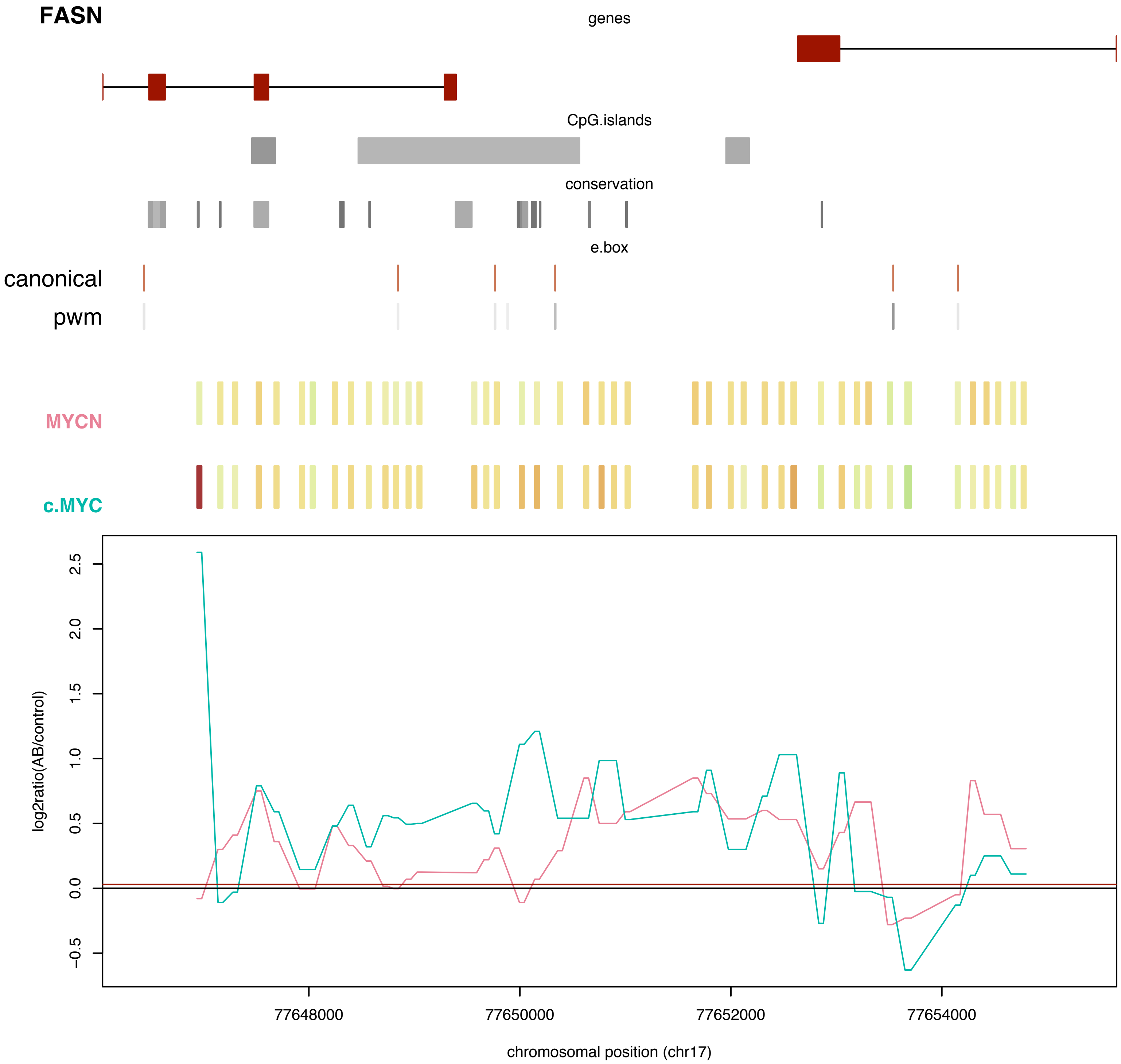

FASN
genes
CpG.islands
conservation
e.box
canonical
pwm
MYCN
c.MYC
log2ratio(AB/control)
2.5
2.0
1.5
1.0
0.5
0.0
−0.5
77648000
77650000
77652000
77654000
chromosomal position (chr17)

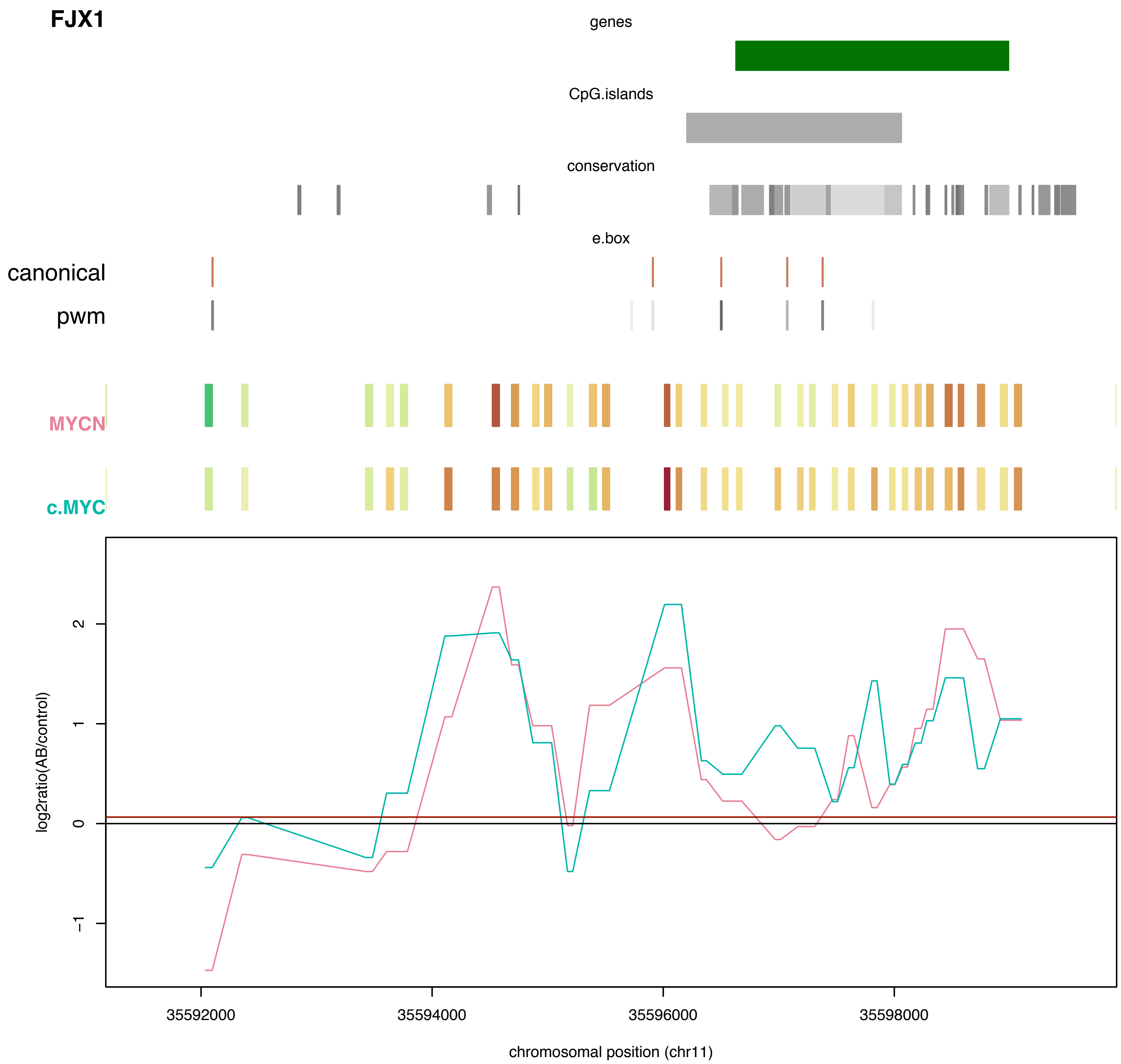

FJX1
genes
CpG.islands
conservation
e.box
canonical
pwm
MYCN
c.MYC
log2ratio(AB/control)
2
1
0
−1
35592000
35594000
35596000
35598000
chromosomal position (chr11)

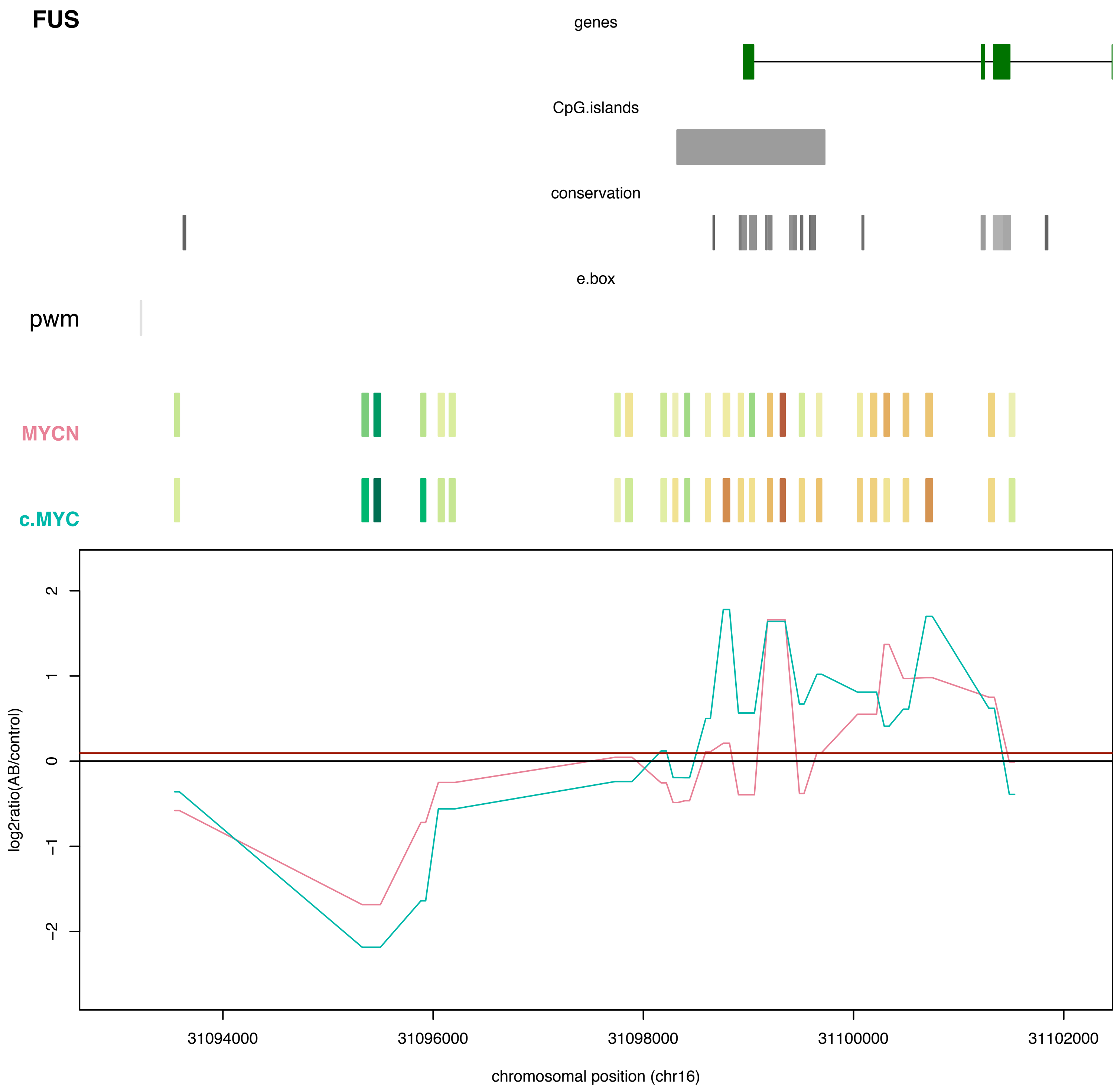
FUS
genes
CpG.islands
conservation
e.box
pwm
MYCN
c.MYC
log2ratio(AB/control)
2
1
0
−1
−2
31094000
31096000
31098000
31100000
31102000
chromosomal position (chr16)

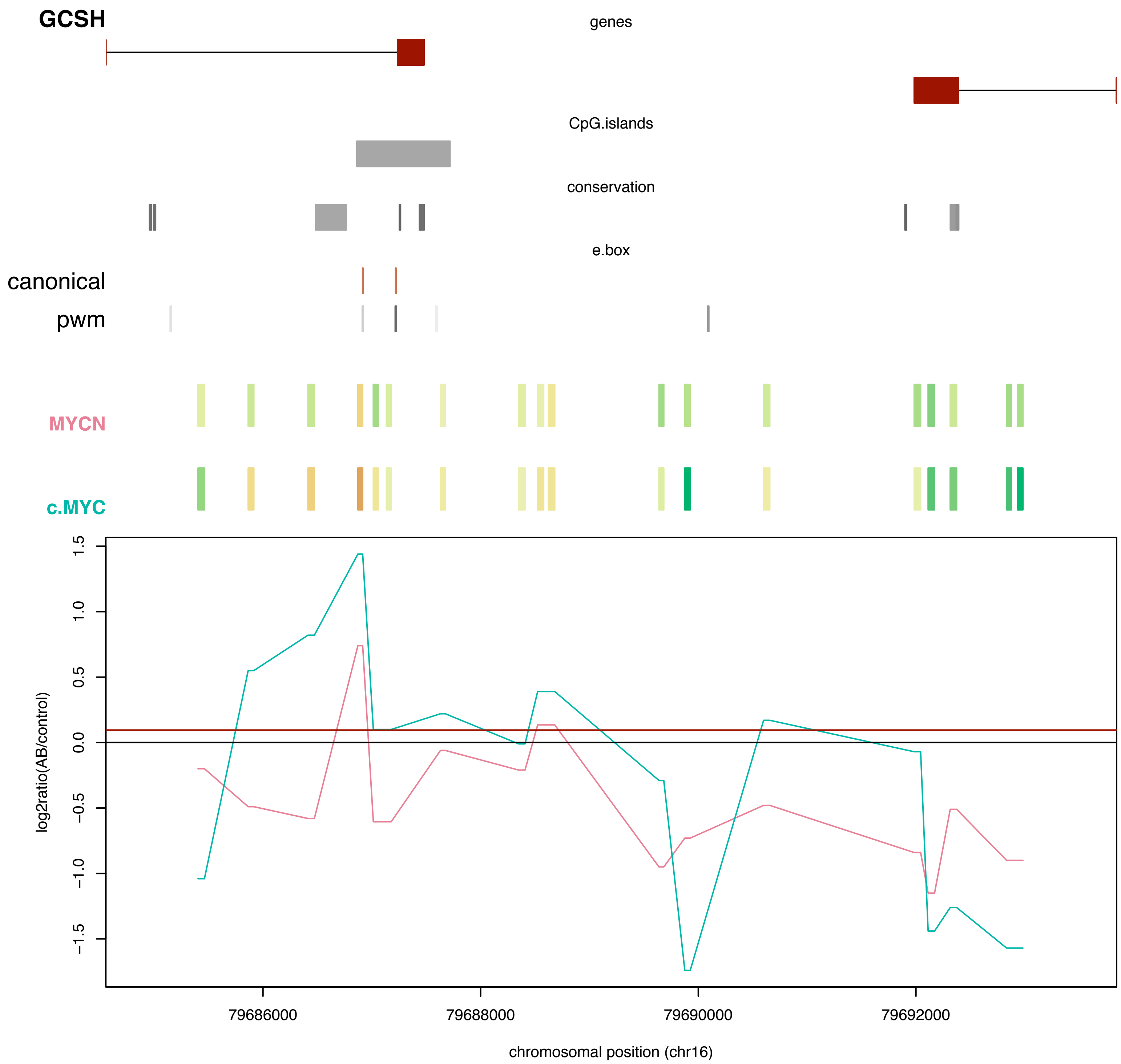

GCSH
genes
CpG.islands
conservation
e.box
canonical
pwm
MYCN
c.MYC
log2ratio(AB/control)
1.5
1.0
0.5
0.0
−0.5
−1.0
−1.5
79686000
79688000
79690000
79692000
chromosomal position (chr16)

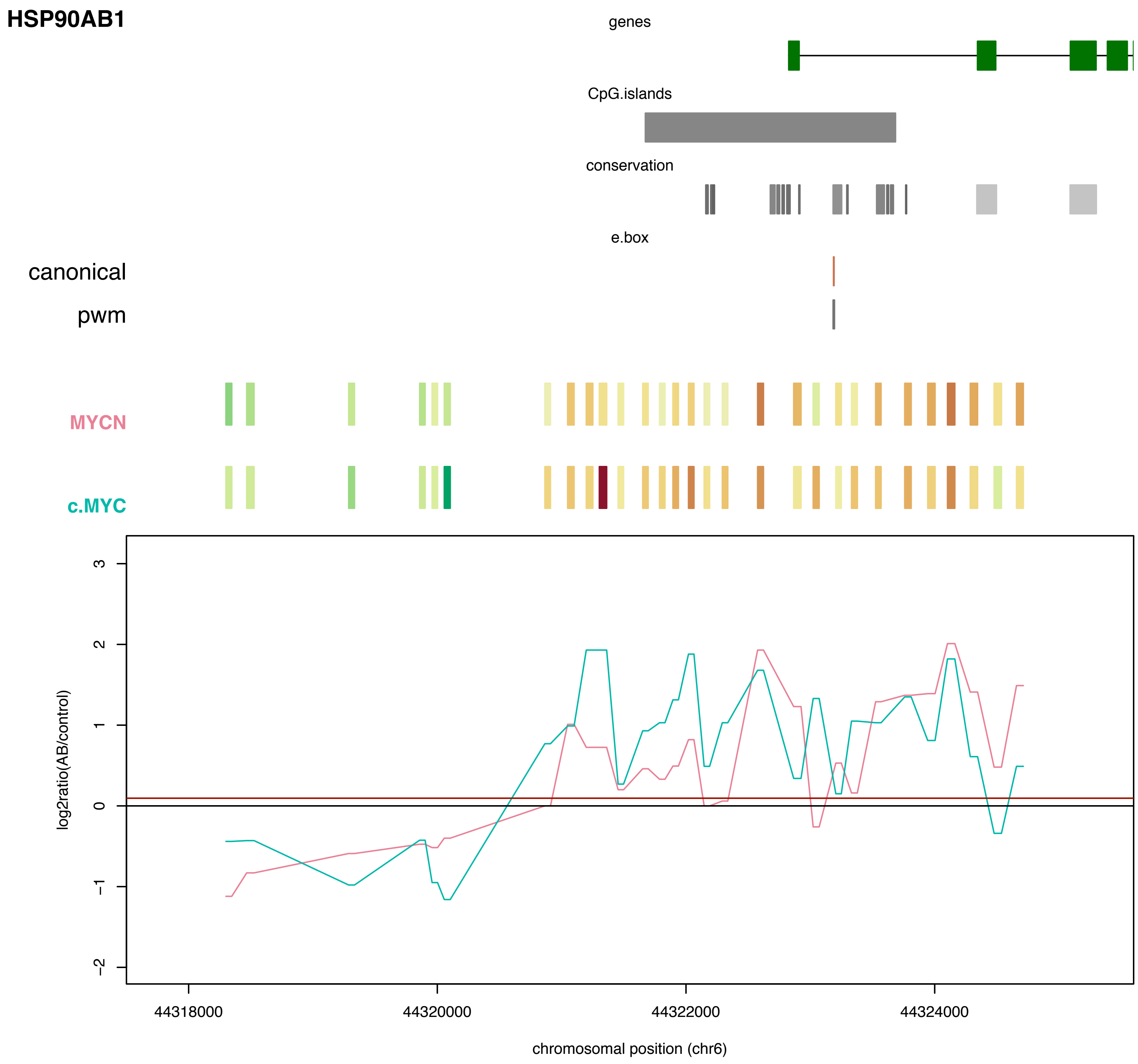

HSP90AB1
genes
CpG.islands
conservation
e.box
canonical
pwm
MYCN
c.MYC
log2ratio(AB/control)
3
2
1
0
−1
−2
44318000
44320000
44322000
44324000
chromosomal position (chr6)

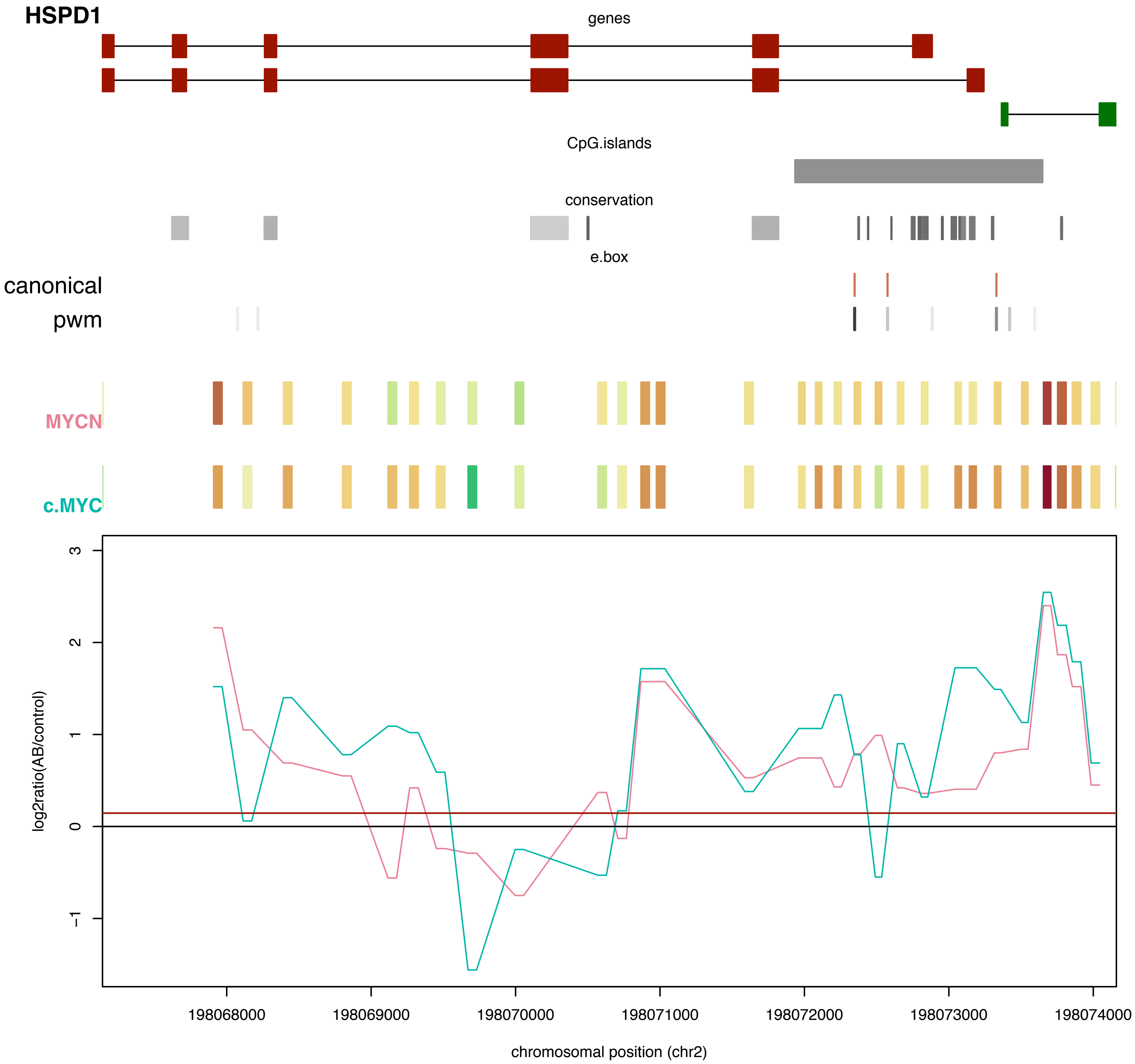

HSPD1
genes
CpG.islands
conservation
e.box
canonical
pwm
MYCN
c.MYC
log2ratio(AB/control)
3
2
1
0
−1
198068000
198069000
198070000
198071000
198072000
198073000
198074000
chromosomal position (chr2)

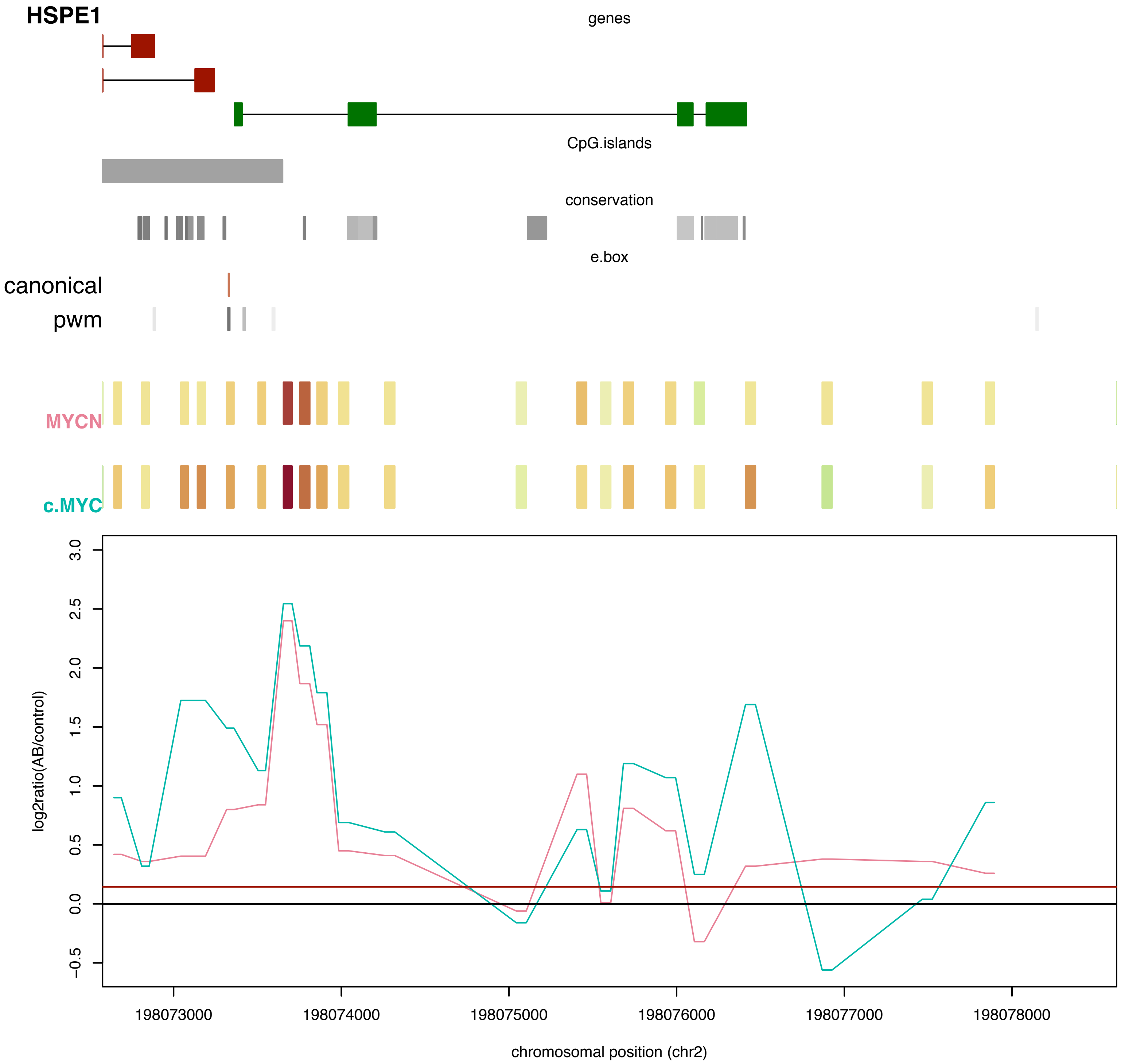
HSPE1
genes
CpG.islands
conservation
e.box
canonical
pwm
MYCN
c.MYC
log2ratio(AB/control)
3.0
2.5
2.0
1.5
1.0
0.5
0.0
−0.5
198073000
198074000
198075000
198076000
198077000
198078000
chromosomal position (chr2)

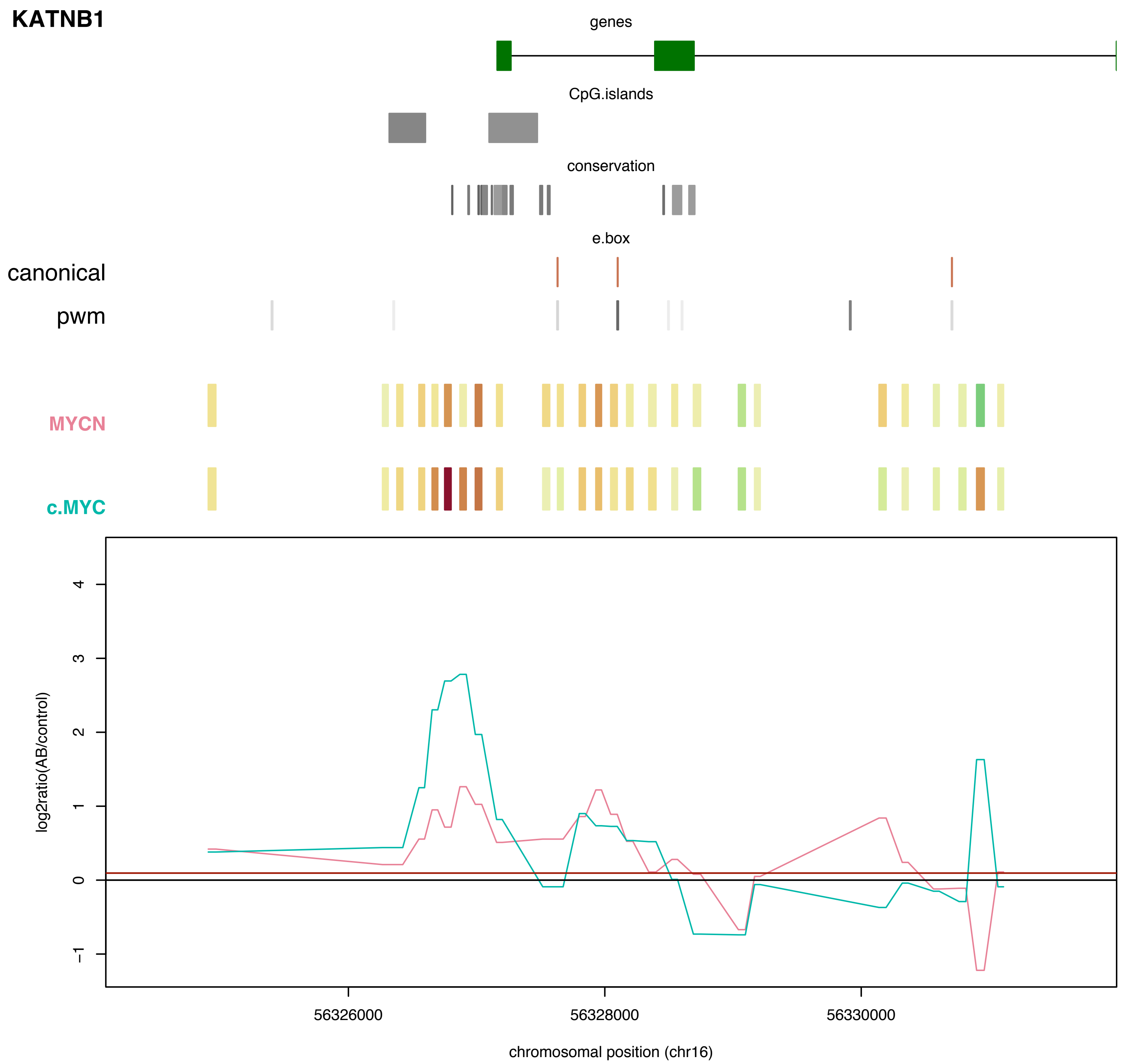

KATNB1
genes
CpG.islands
conservation
e.box
canonical
pwm
MYCN
c.MYC
log2ratio(AB/control)
−1
0
1
2
3
4
56326000
56328000
56330000
chromosomal position (chr16)

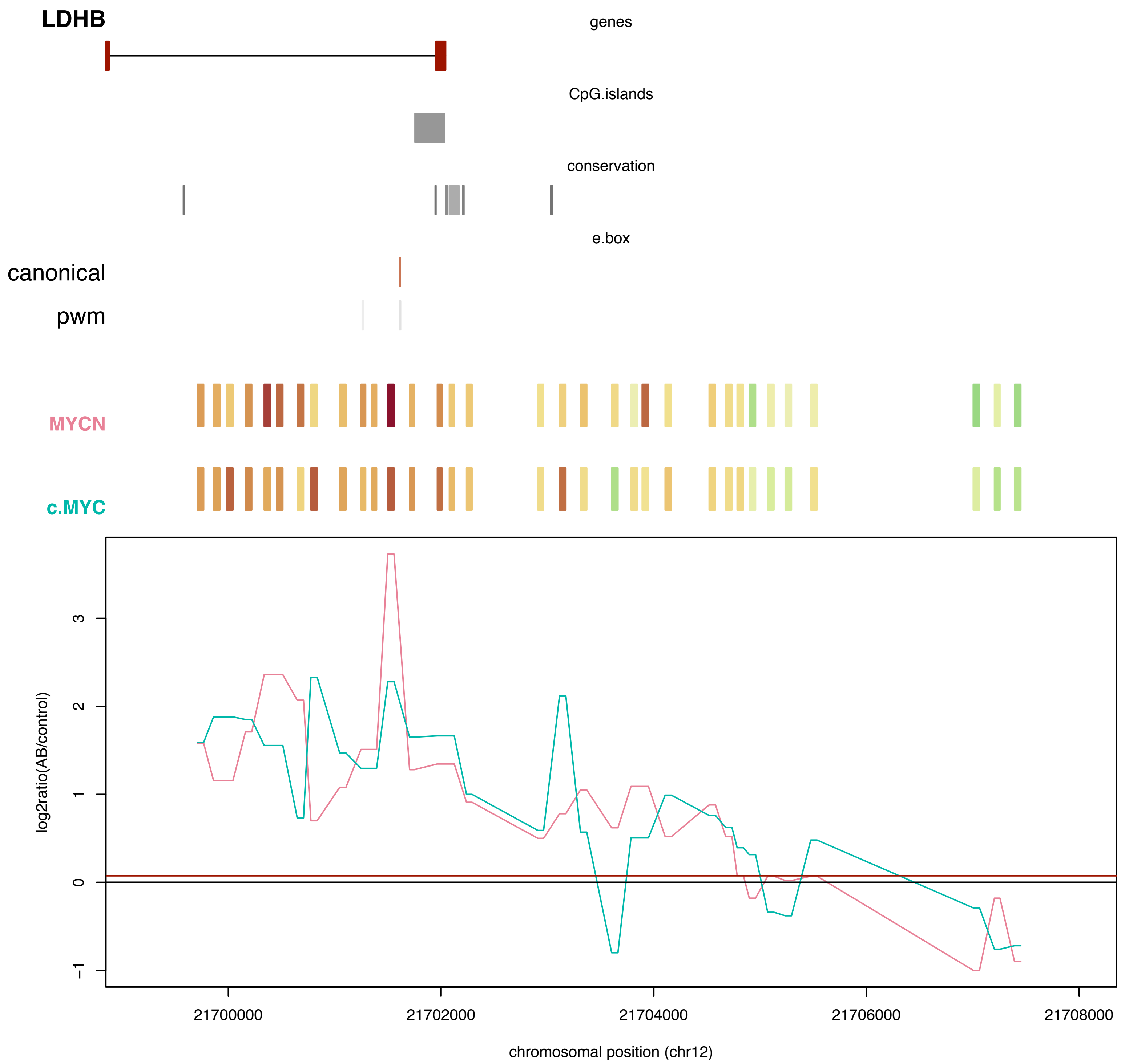

LDHB
genes
CpG.islands
conservation
e.box
canonical
pwm
MYCN
c.MYC
log2ratio(AB/control)
3
2
1
0
−1
21700000
21702000
21704000
21706000
21708000
chromosomal position (chr12)

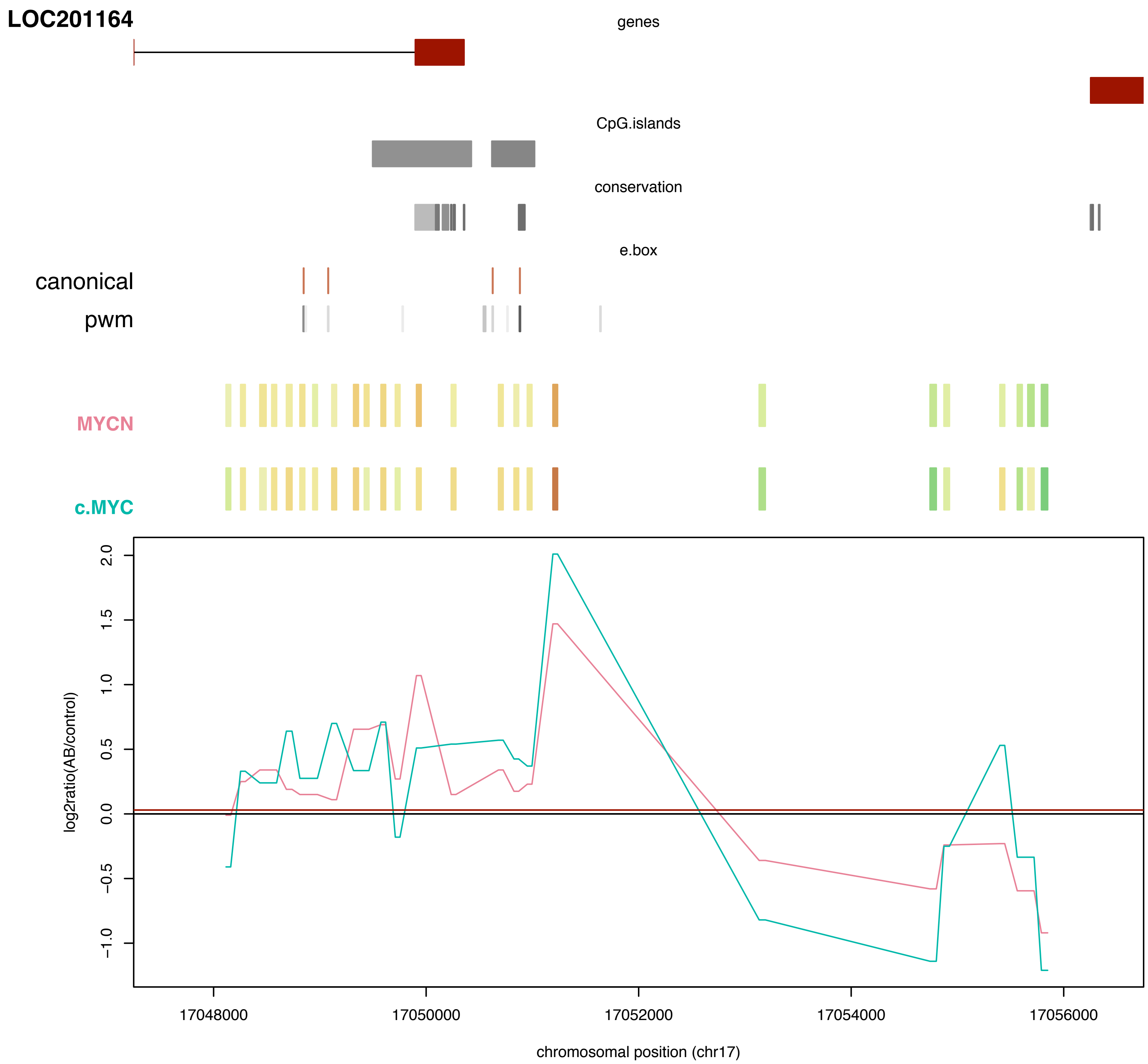

LOC201164
genes
CpG.islands
conservation
e.box
canonical
pwm
MYCN
c.MYC
log2ratio(AB/control)
2.0
1.5
1.0
0.5
0.0
−0.5
−1.0
17048000
17050000
17052000
17054000
17056000
chromosomal position (chr17)

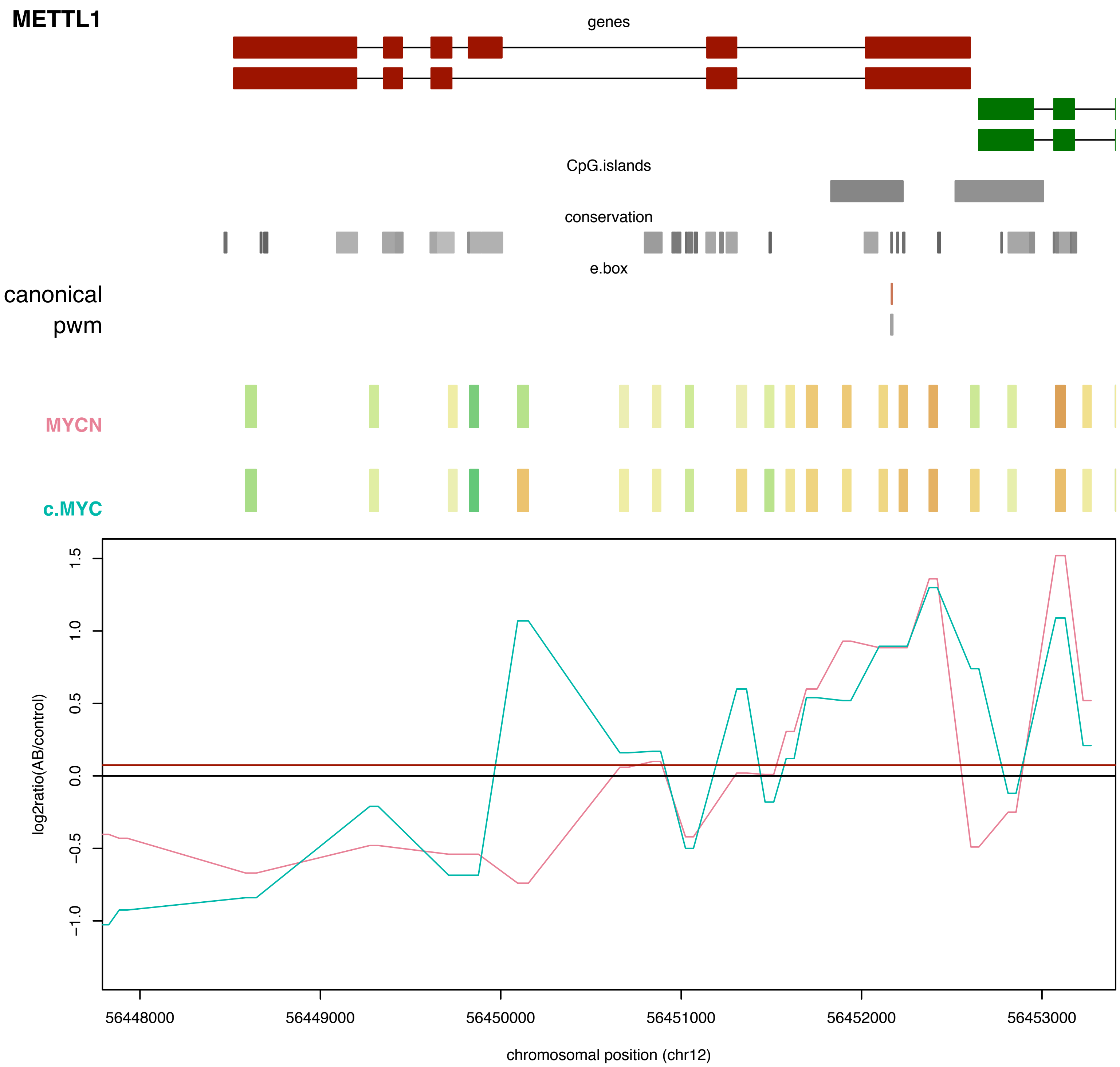
METTL1
genes
CpG.islands
conservation
e.box
canonical
pwm
MYCN
c.MYC
log2ratio(AB/control)
1.5
1.0
0.5
0.0
−0.5
−1.0
56448000
56449000
56450000
56451000
56452000
56453000
chromosomal position (chr12)

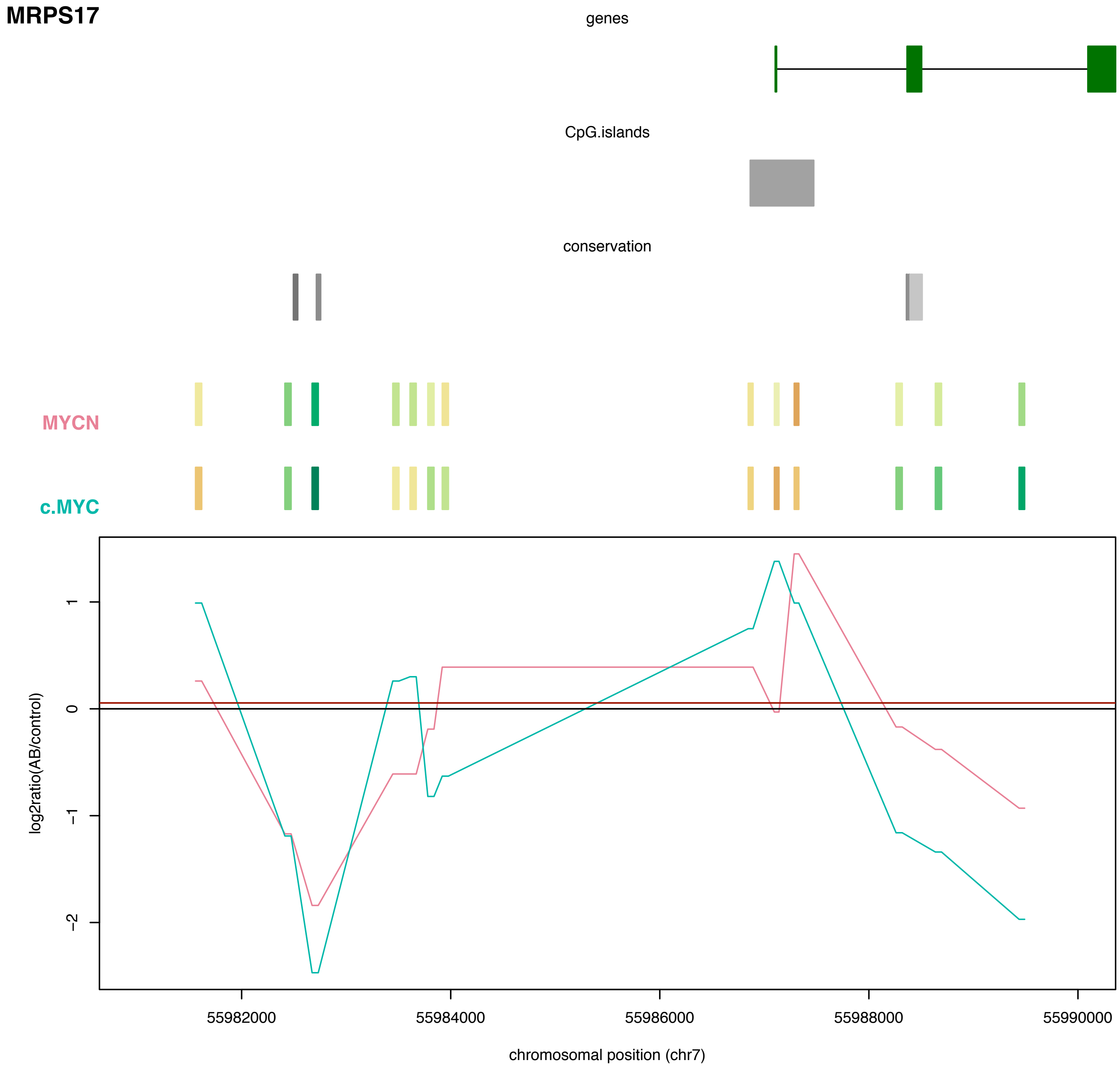

MRPS17
genes
CpG.islands
conservation
MYCN
c.MYC
log2ratio(AB/control)
1
0
−1
−2
55982000
55984000
55986000
55988000
55990000
chromosomal position (chr7)

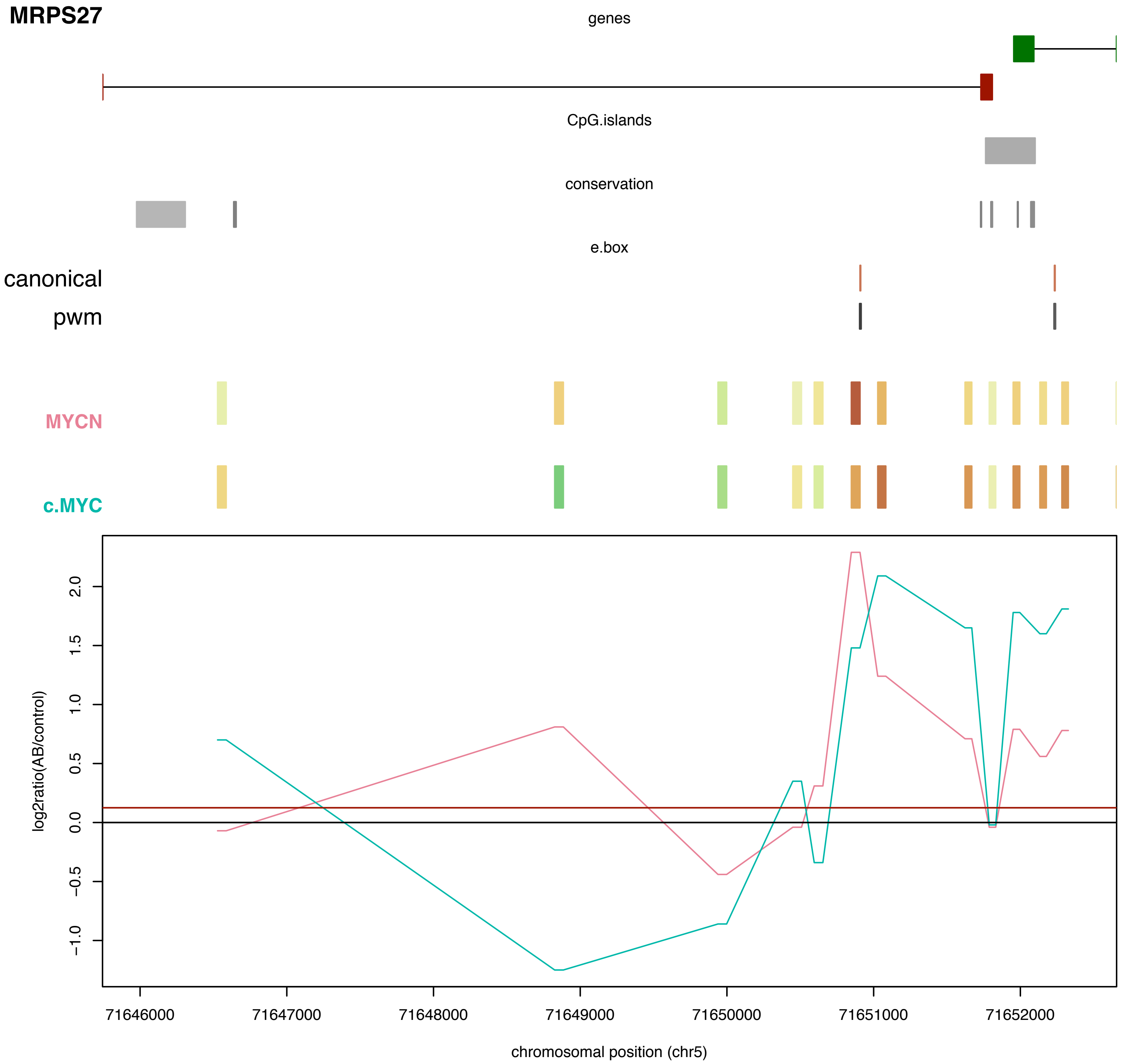
MRPS27
genes
CpG.islands
conservation
e.box
canonical
pwm
MYCN
c.MYC
log2ratio(AB/control)
−1.0
−0.5
0.0
0.5
1.0
1.5
2.0
71646000
71647000
71648000
71649000
71650000
71651000
71652000
chromosomal position (chr5)

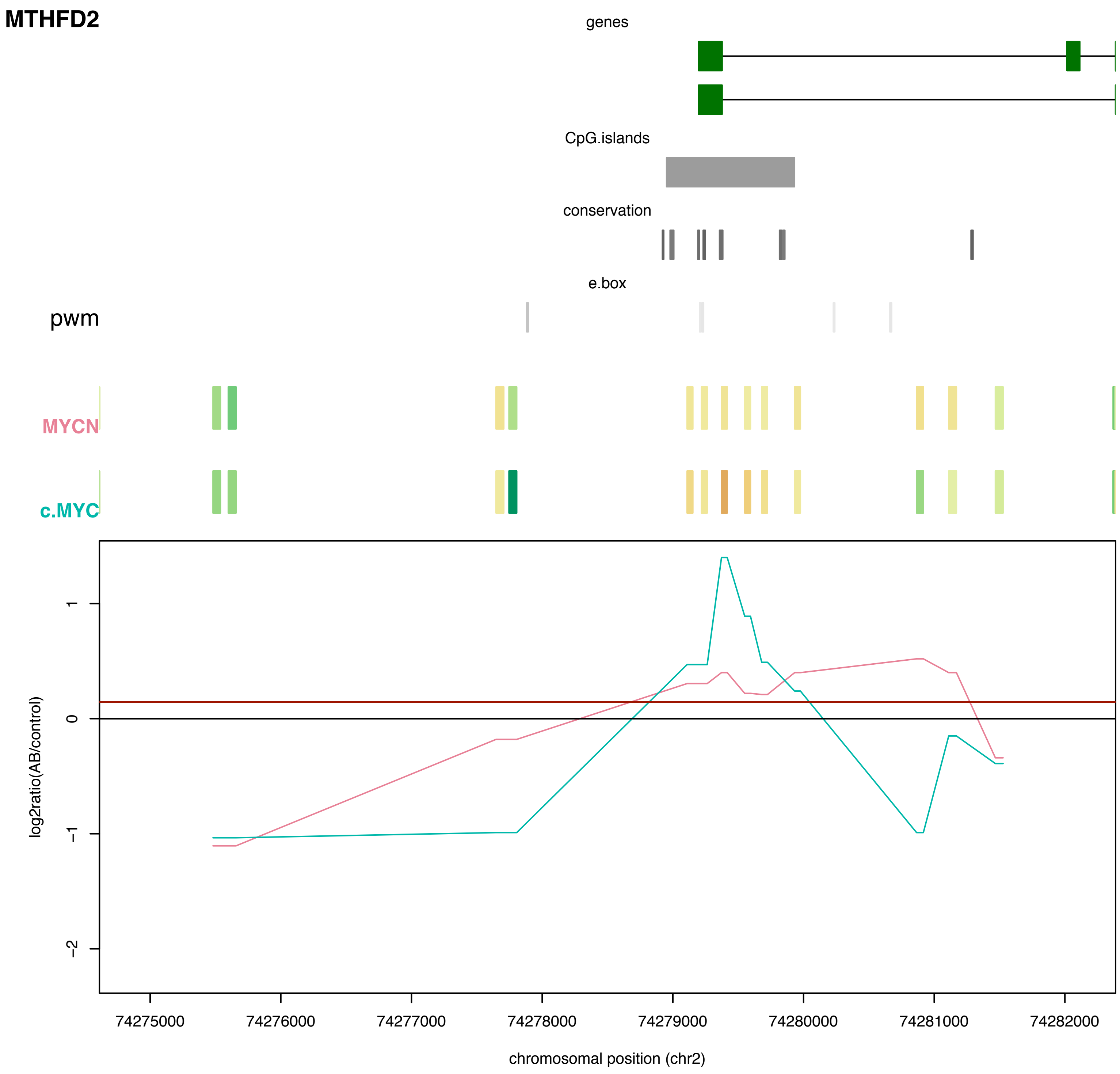

MTHFD2
genes
CpG.islands
conservation
e.box
pwm
MYCN
c.MYC
log2ratio(AB/control)
1
0
−1
−2
74275000
74276000
74277000
74278000
74279000
74280000
74281000
74282000
chromosomal position (chr2)

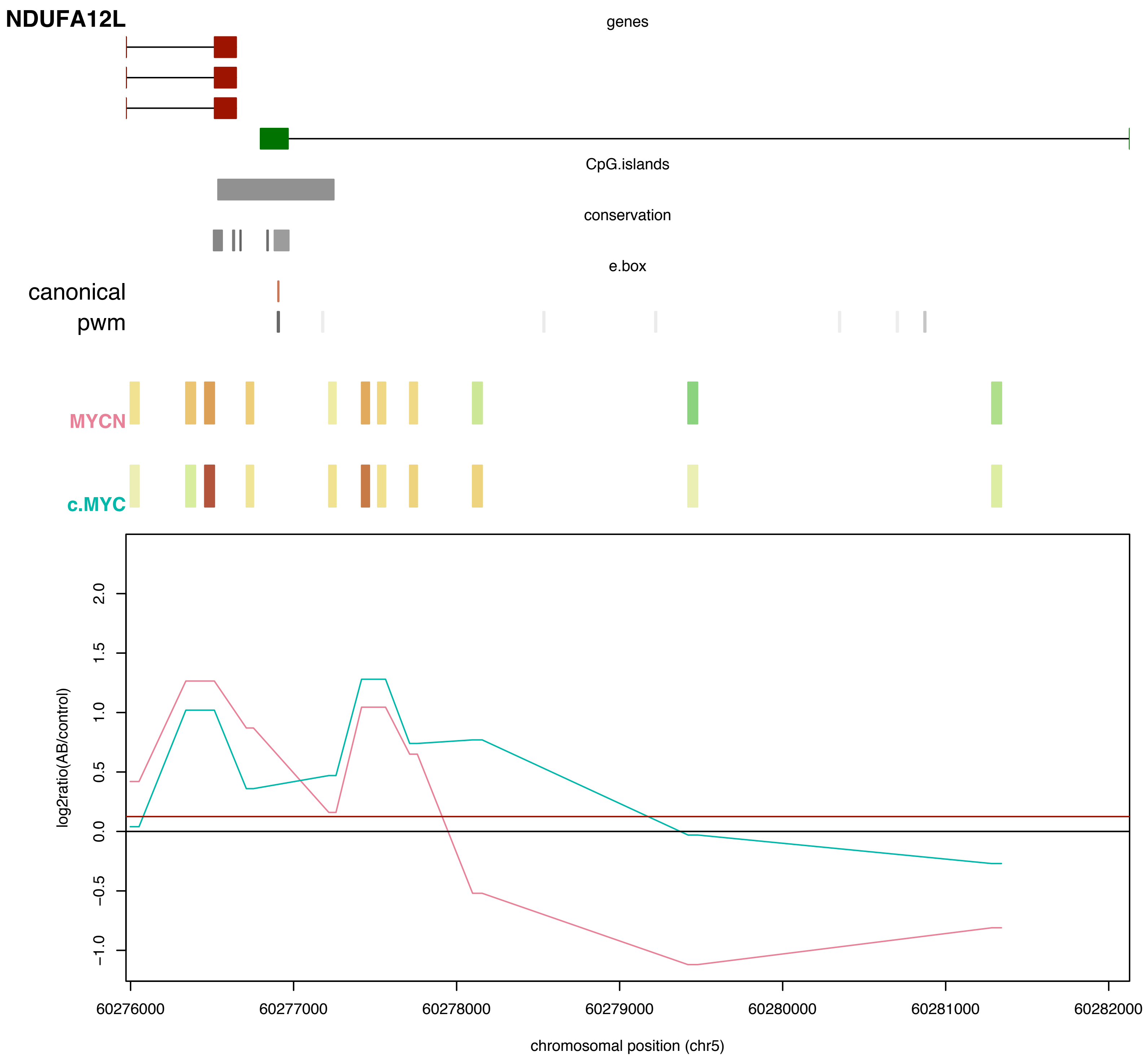
NDUFA12L
genes
CpG.islands
conservation
e.box
canonical
pwm
MYCN
c.MYC
log2ratio(AB/control)
2.0
1.5
1.0
0.5
0.0
−0.5
−1.0
60276000
60277000
60278000
60279000
60280000
60281000
60282000
chromosomal position (chr5)

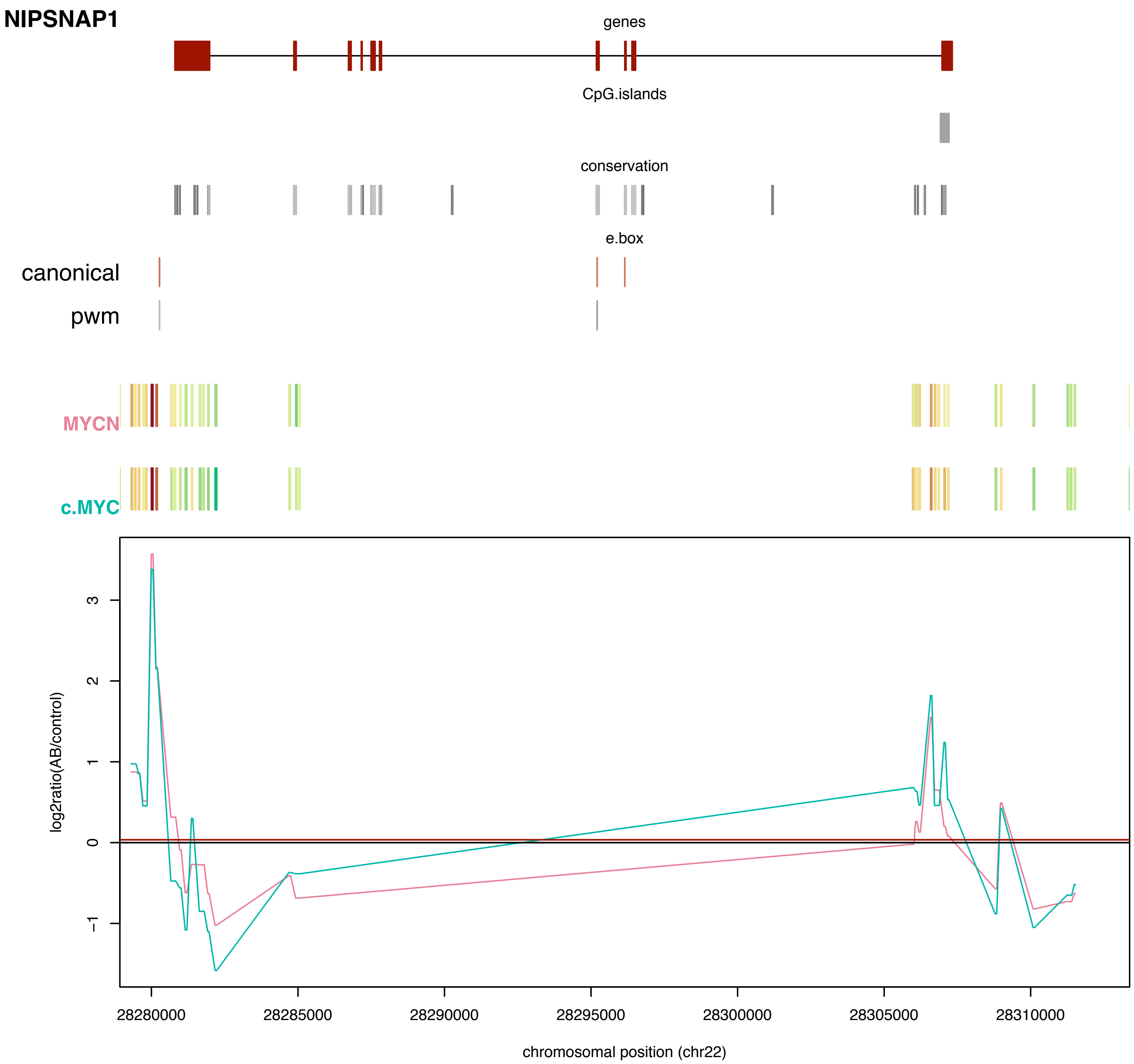

NIPSNAP1
genes
CpG.islands
conservation
e.box
canonical
pwm
MYCN
c.MYC
log2ratio(AB/control)
−1
0
1
2
3
28280000
28285000
28290000
28295000
28300000
28305000
28310000
chromosomal position (chr22)

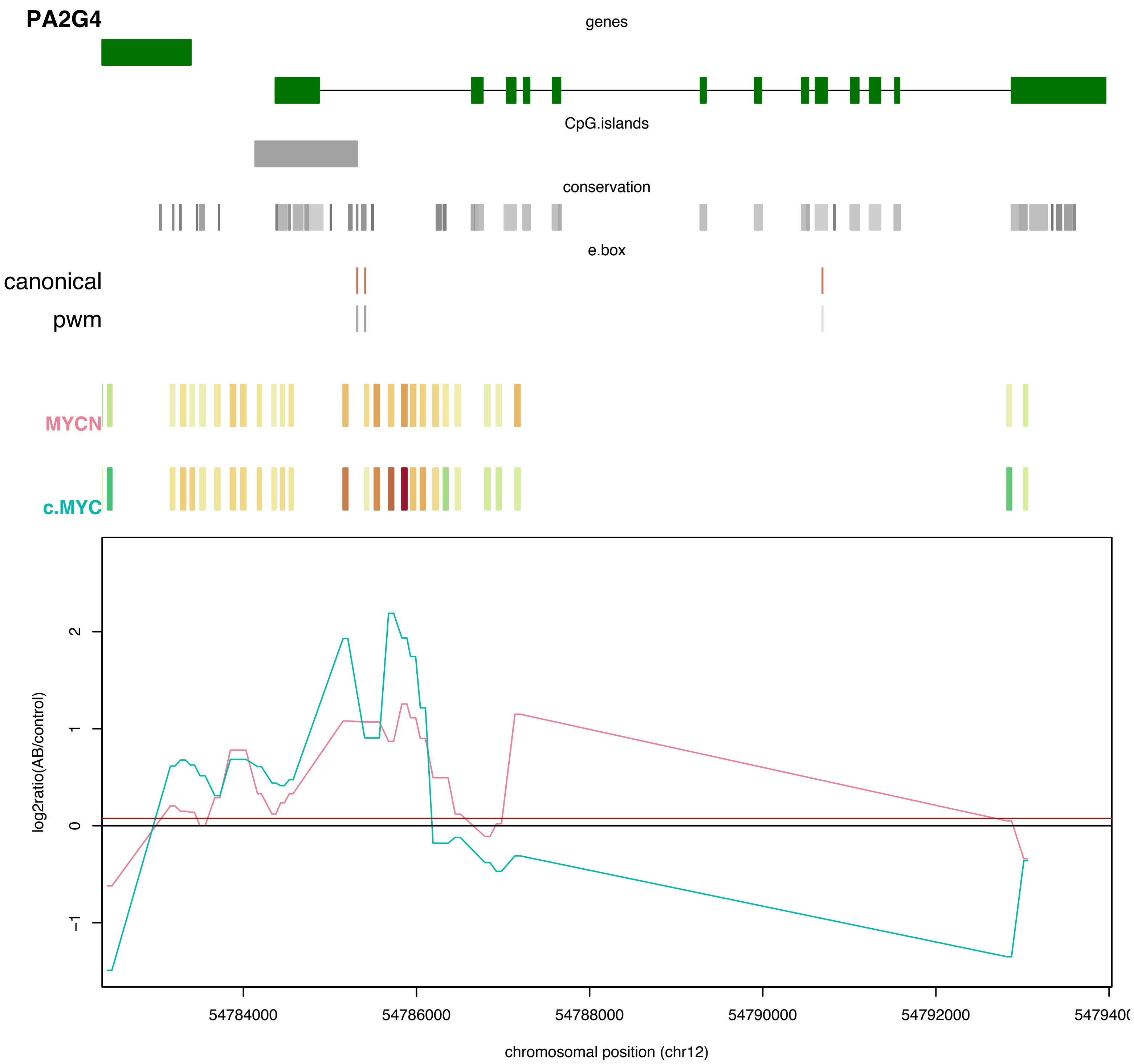

PA2G4
genes
CpG.islands
conservation
e.box
canonical
pwm
MYCN
c.MYC
log2ratio(AB/control)
2
1
0
−1
54784000
54786000
54788000
54790000
54792000
chromosomal position (chr12)

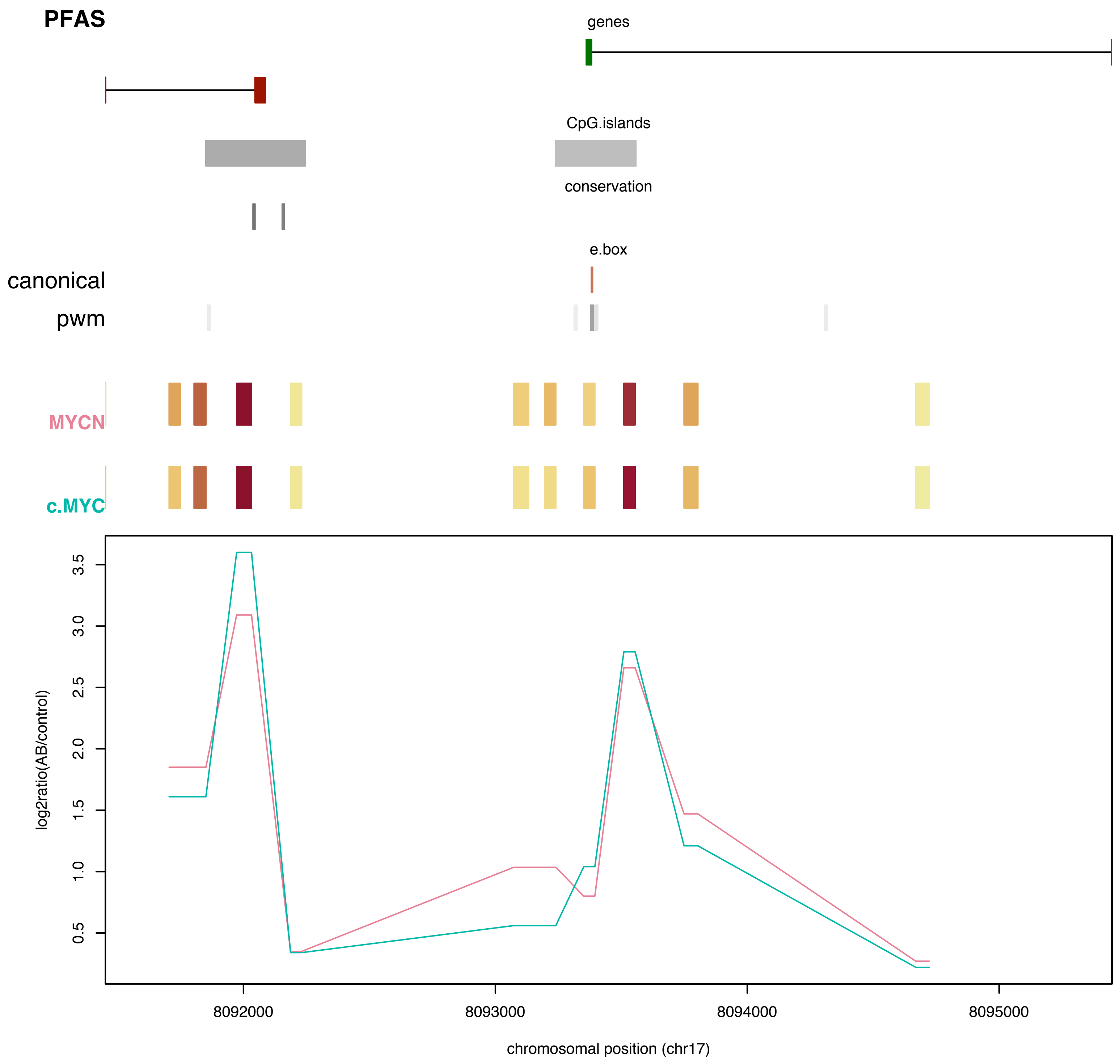

PFAS
genes
CpG.islands
conservation
e.box
canonical
pwm
MYCN
c.MYC
log2ratio(AB/control)
0.5
1.0
1.5
2.0
2.5
3.0
3.5
8092000
8093000
8094000
8095000
chromosomal position (chr17)

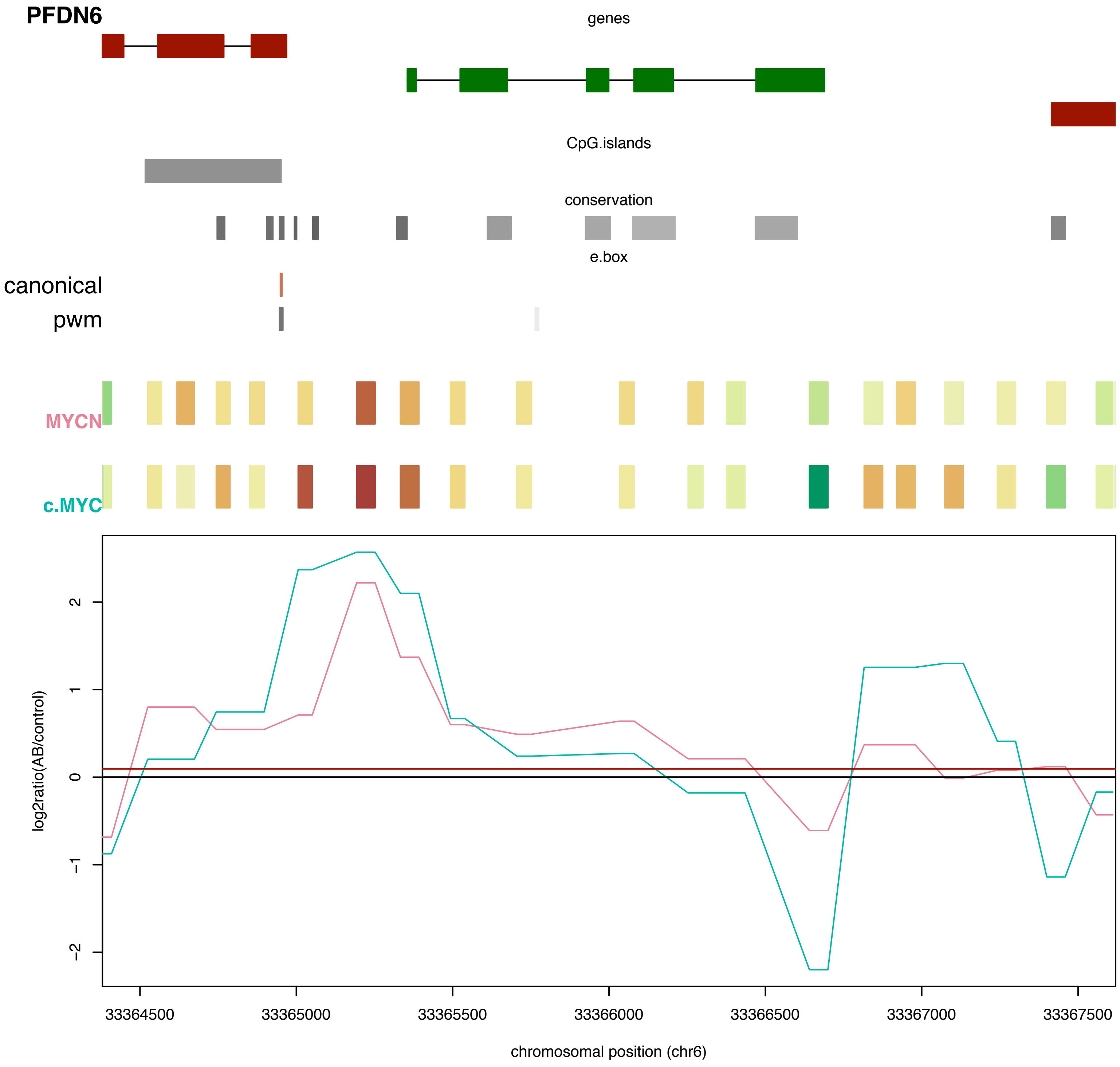
PFDN6
genes
CpG.islands
conservation
e.box
canonical
pwm
MYCN
c.MYC
log2ratio(AB/control)
2
1
0
−1
−2
33364500
33365000
33365500
33366000
33366500
33367000
33367500
chromosomal position (chr6)

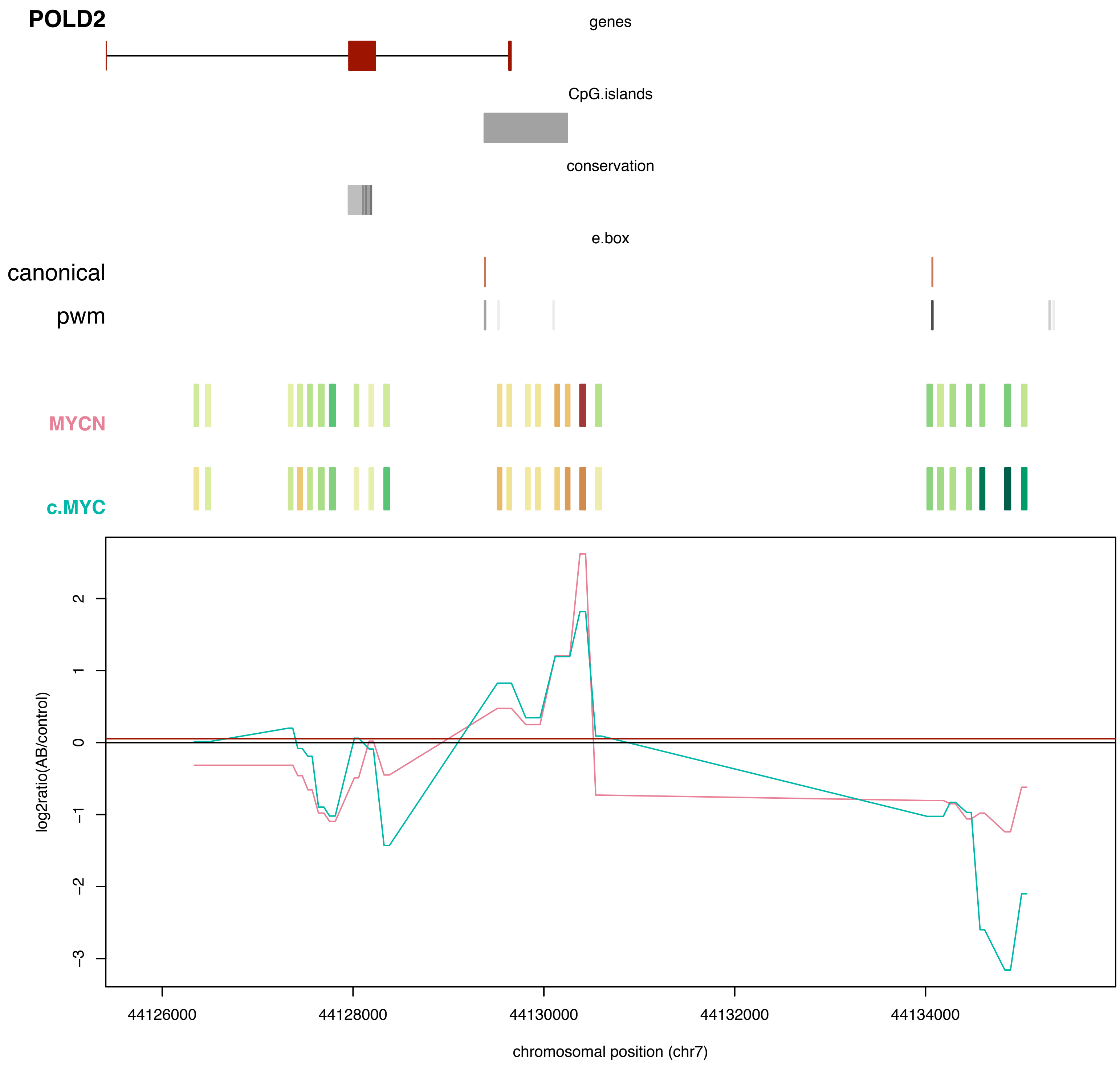

POLD2
genes
CpG.islands
conservation
e.box
canonical
pwm
MYCN
c.MYC
log2ratio(AB/control)
2
1
0
−1
−2
−3
44126000
44128000
44130000
44132000
44134000
chromosomal position (chr7)

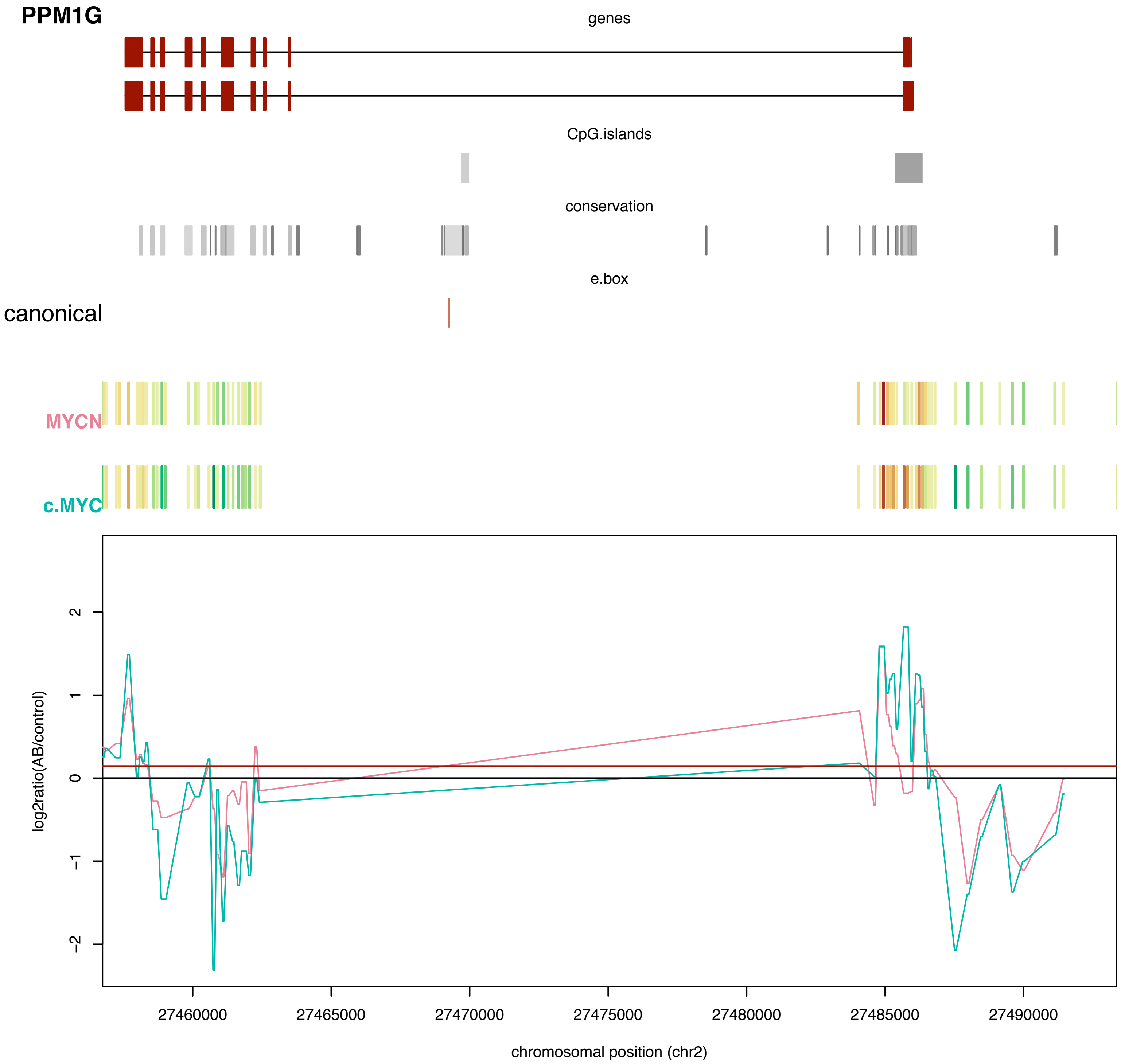

PPM1G
genes
CpG.islands
conservation
e.box
canonical
MYCN
c.MYC
log2ratio(AB/control)
2
1
0
−1
−2
27460000
27465000
27470000
27475000
27480000
27485000
27490000
chromosomal position (chr2)

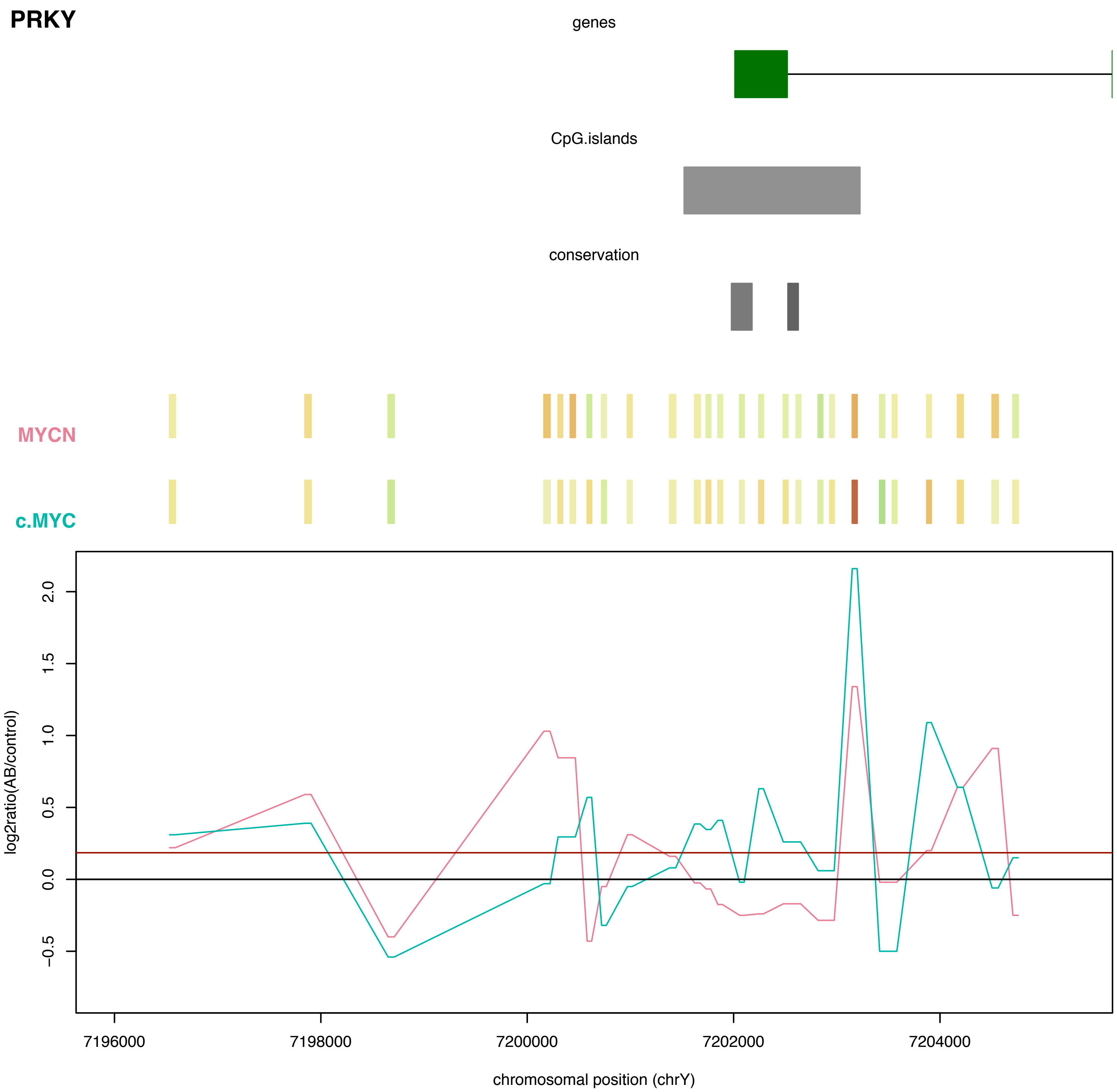

PRKY
genes
CpG.islands
conservation
MYCN
c.MYC
log2ratio(AB/control)
−0.5
0.0
0.5
1.0
1.5
2.0
7196000
7198000
7200000
7202000
7204000
chromosomal position (chrY)

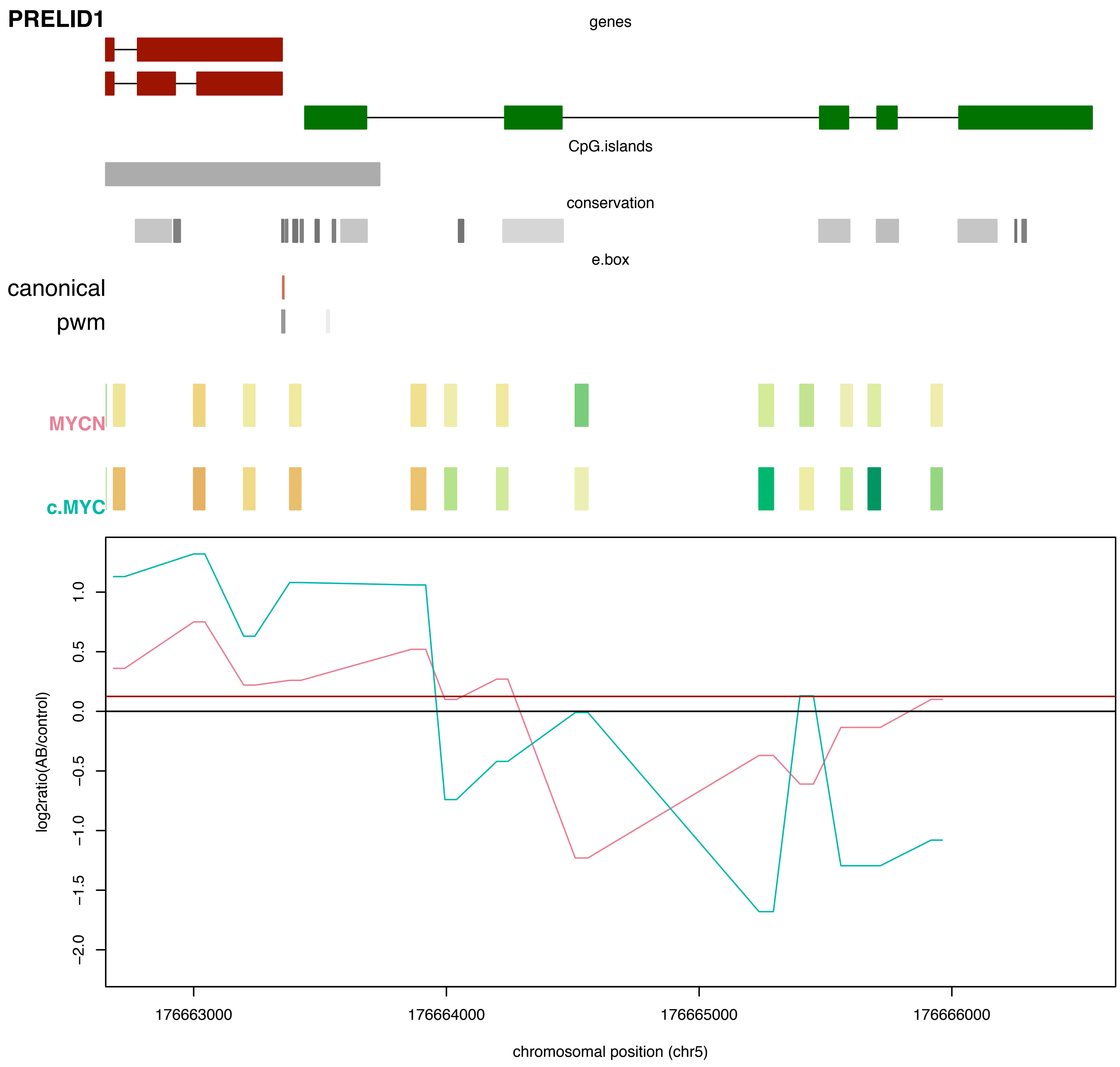

PRELID1
genes
CpG.islands
conservation
e.box
canonical
pwm
MYCN
c.MYC
log2ratio(AB/control)
1.0
0.5
0.0
−0.5
−1.0
−1.5
−2.0
176663000
176664000
176665000
176666000
chromosomal position (chr5)

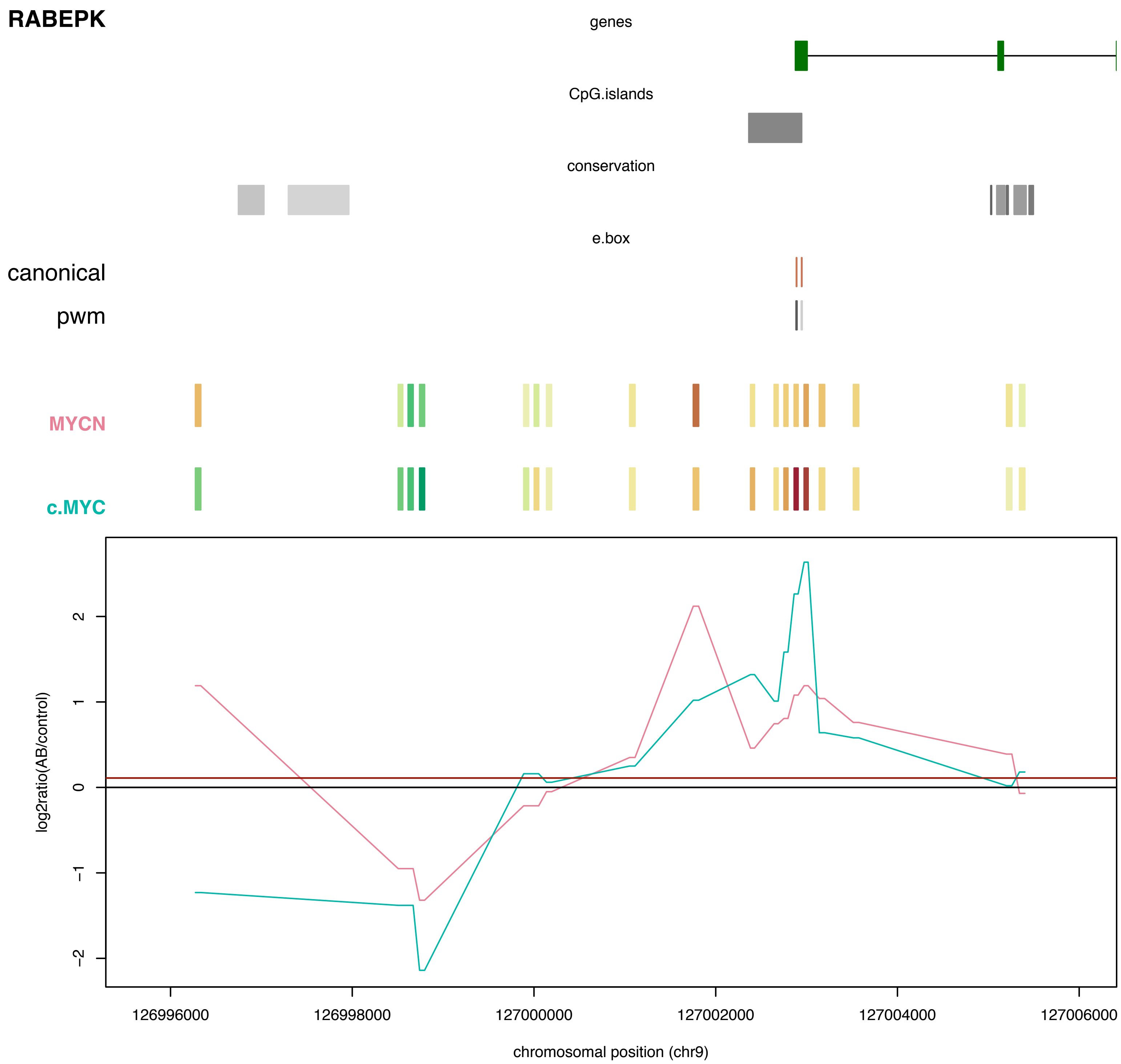

RABEPK
genes
CpG.islands
conservation
e.box
canonical
pwm
MYCN
c.MYC
log2ratio(AB/control)
2
1
0
−1
−2
126996000
126998000
127000000
127002000
127004000
127006000
chromosomal position (chr9)

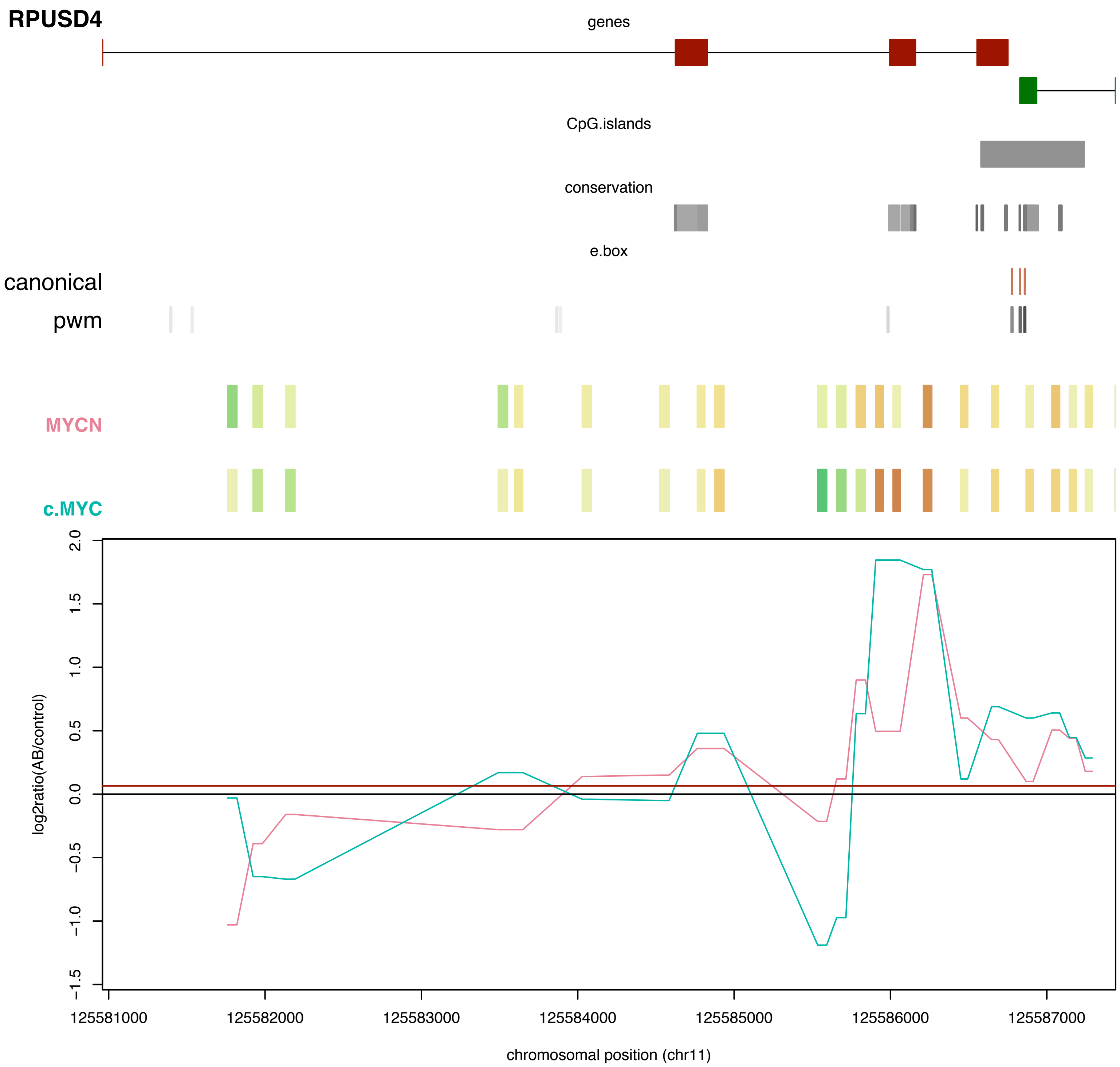
RPUSD4
genes
CpG.islands
conservation
e.box
canonical
pwm
MYCN
c.MYC
log2ratio(AB/control)
2.0
1.5
1.0
0.5
0.0
−0.5
−1.0
−1.5
125581000
125582000
125583000
125584000
125585000
125586000
125587000
chromosomal position (chr11)

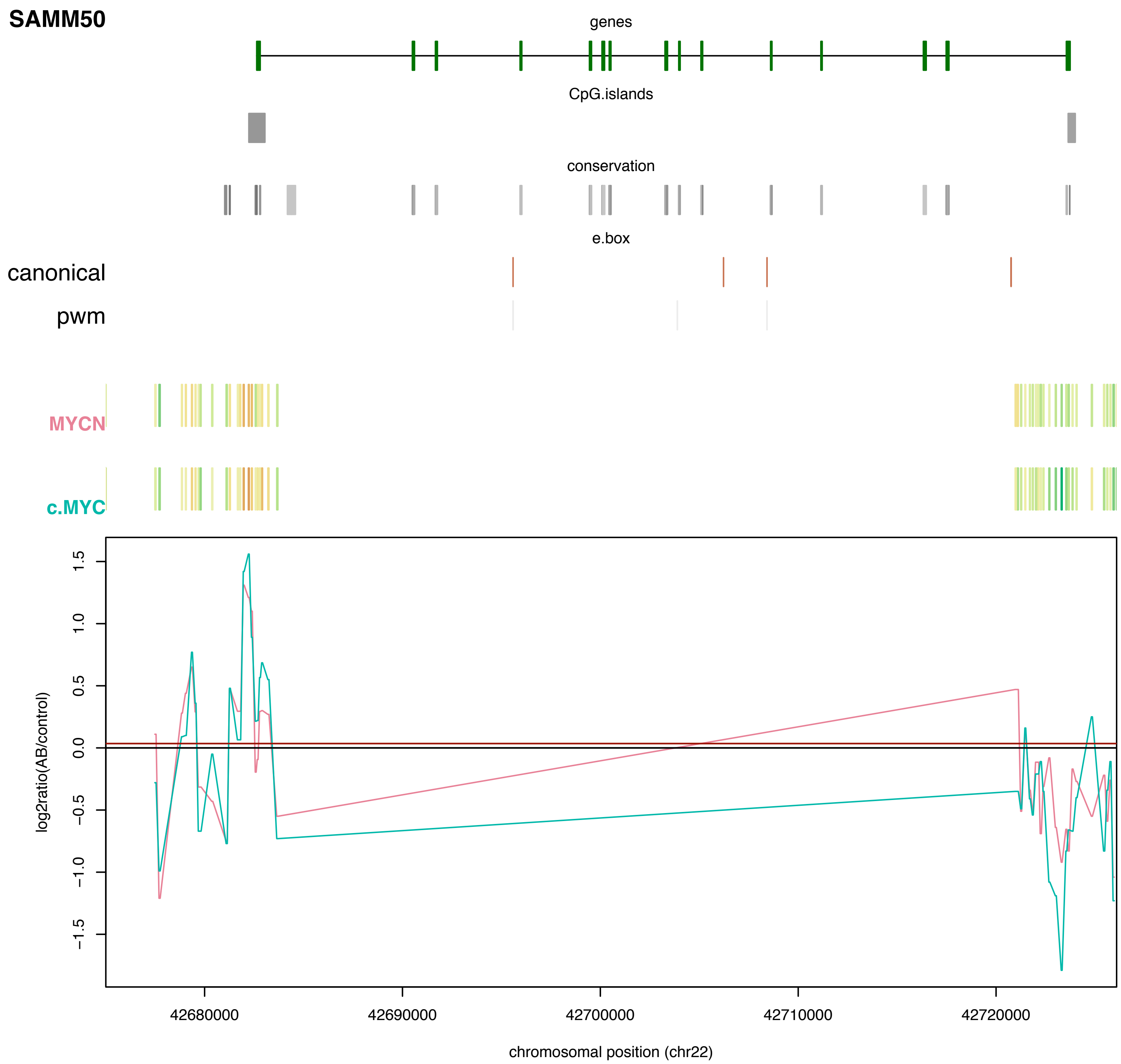
SAMM50
genes
CpG.islands
conservation
e.box
canonical
pwm
MYCN
c.MYC
log2ratio(AB/control)
1.5
1.0
0.5
0.0
−0.5
−1.0
−1.5
42680000
42690000
42700000
42710000
42720000
chromosomal position (chr22)

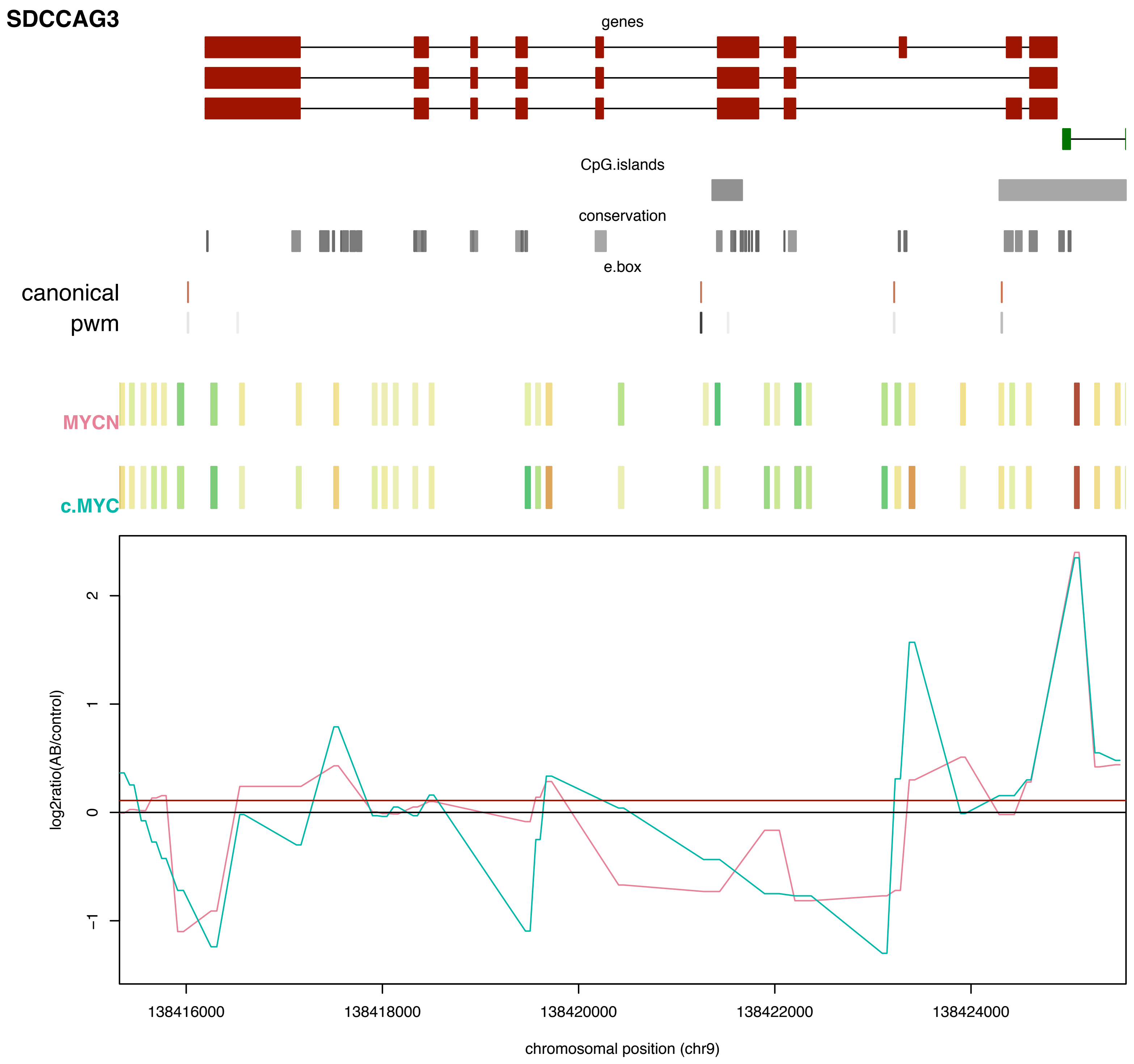

SDCCAG3
genes
CpG.islands
conservation
e.box
canonical
pwm
MYCN
c.MYC
log2ratio(AB/control)
2
1
0
−1
138416000
138418000
138420000
138422000
138424000
chromosomal position (chr9)

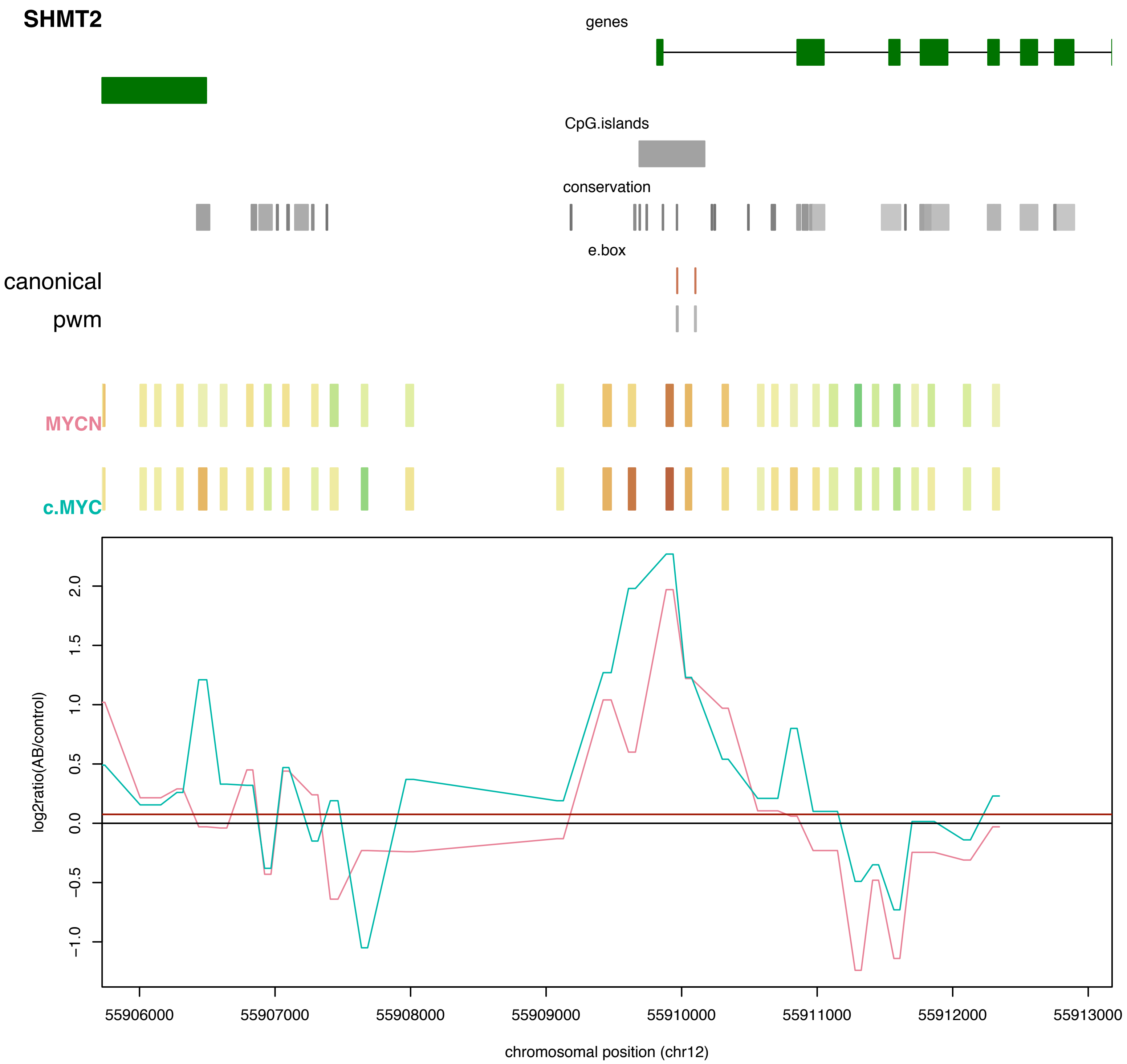

SHMT2
genes
CpG.islands
conservation
e.box
canonical
pwm
MYCN
c.MYC
log2ratio(AB/control)
2.0
1.5
1.0
0.5
0.0
−0.5
−1.0
55906000
55907000
55908000
55909000
55910000
55911000
55912000
55913000
chromosomal position (chr12)

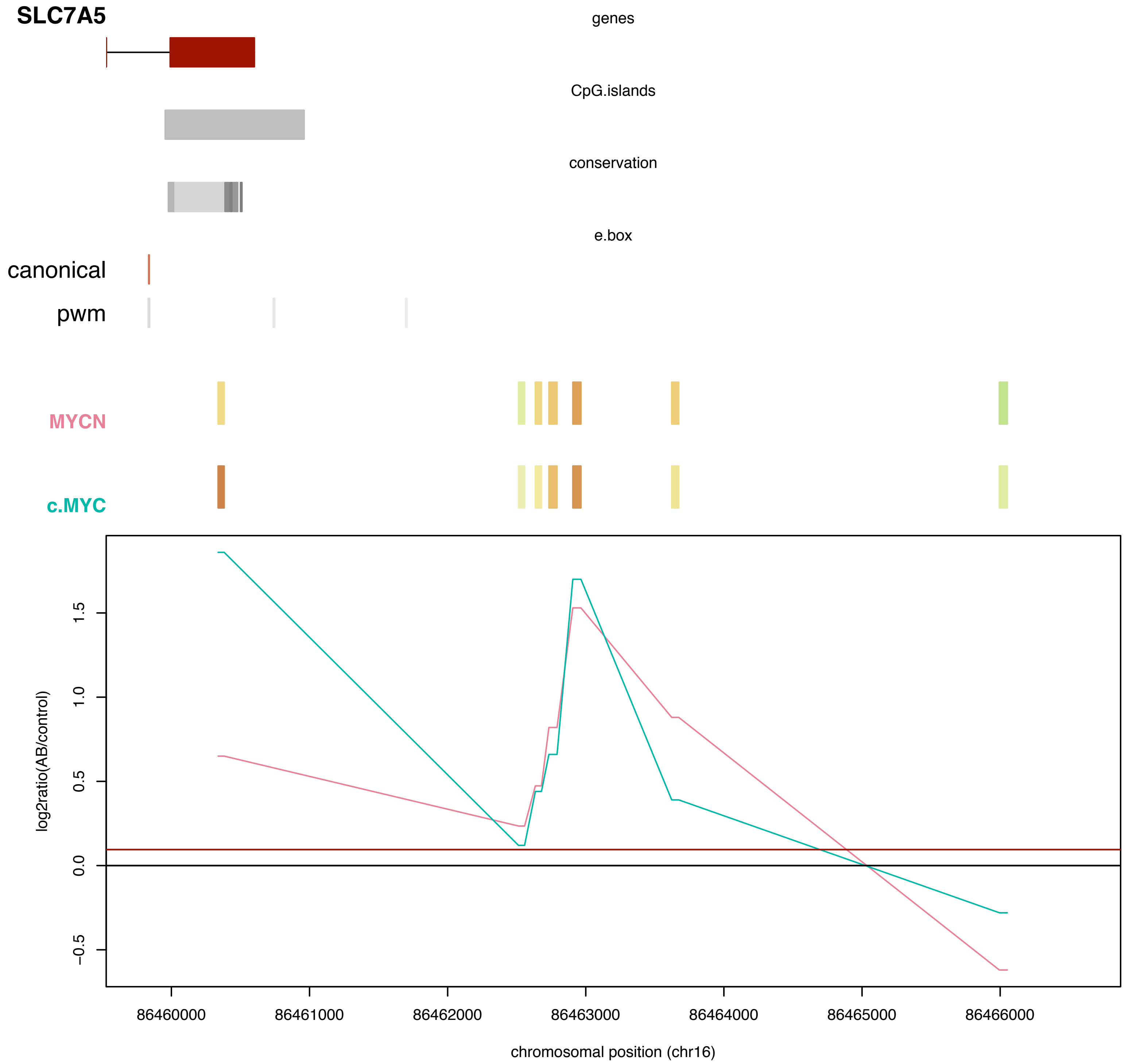

SLC7A5
genes
CpG.islands
conservation
e.box
canonical
pwm
MYCN
c.MYC
log2ratio(AB/control)
−0.5
0.0
0.5
1.0
1.5
86460000
86461000
86462000
86463000
86464000
86465000
86466000
chromosomal position (chr16)

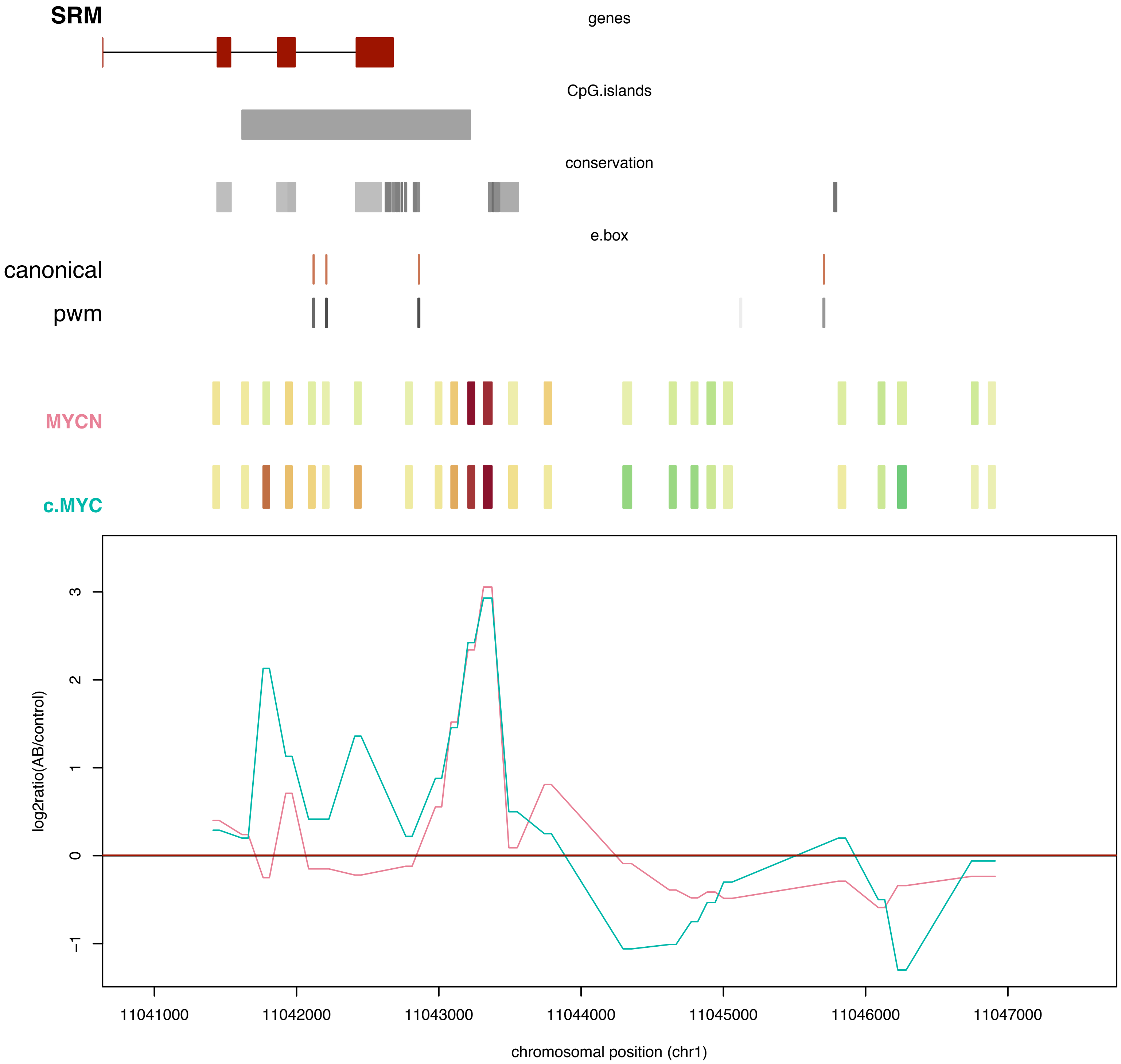

SRM
genes
CpG.islands
conservation
e.box
canonical
pwm
MYCN
c.MYC
log2ratio(AB/control)
3
2
1
0
−1
11041000
11042000
11043000
11044000
11045000
11046000
11047000
chromosomal position (chr1)

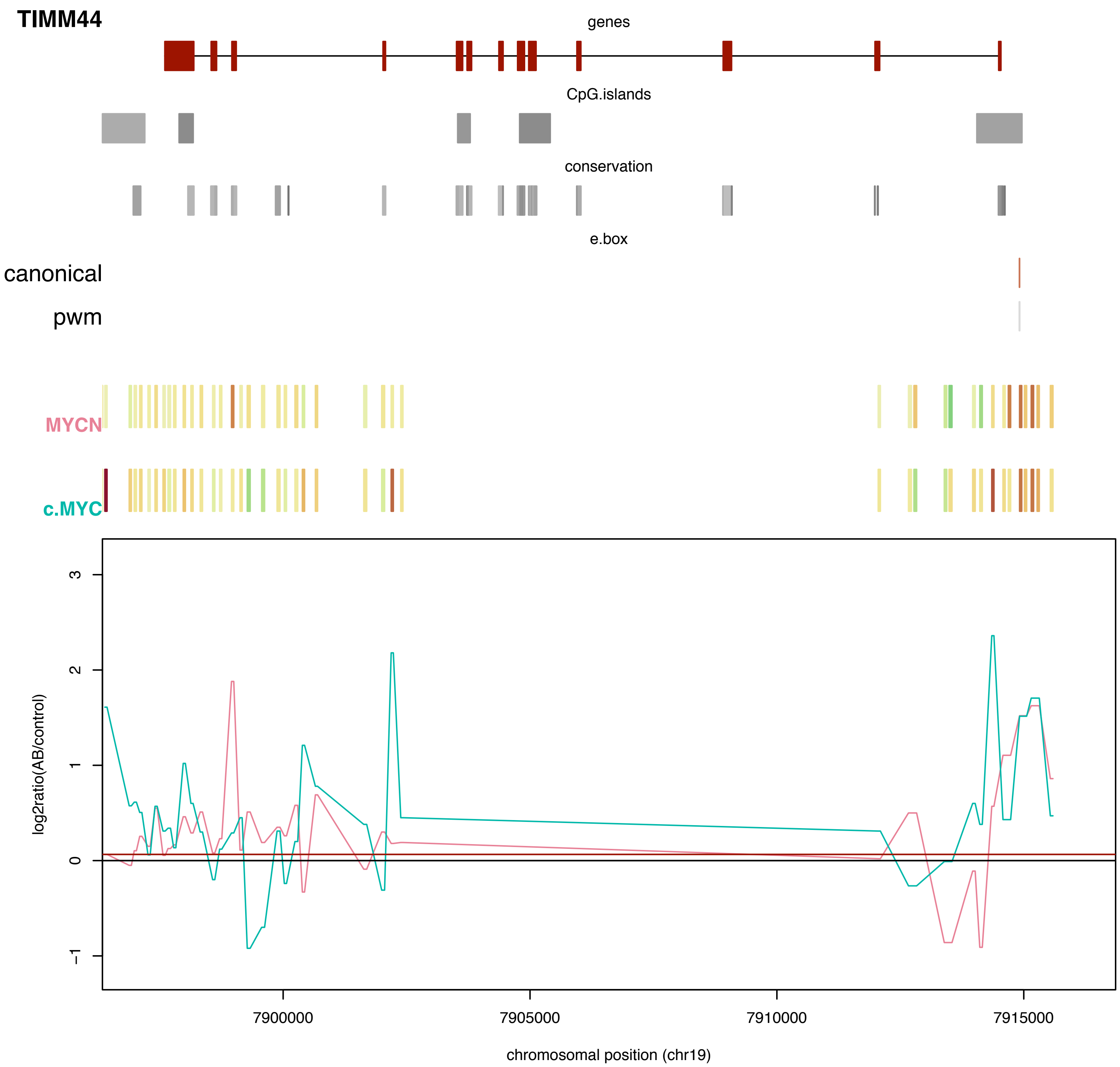
TIMM44
genes
CpG.islands
conservation
e.box
canonical
pwm
MYCN
c.MYC
log2ratio(AB/control)
3
2
1
0
−1
7900000
7905000
7910000
7915000
chromosomal position (chr19)

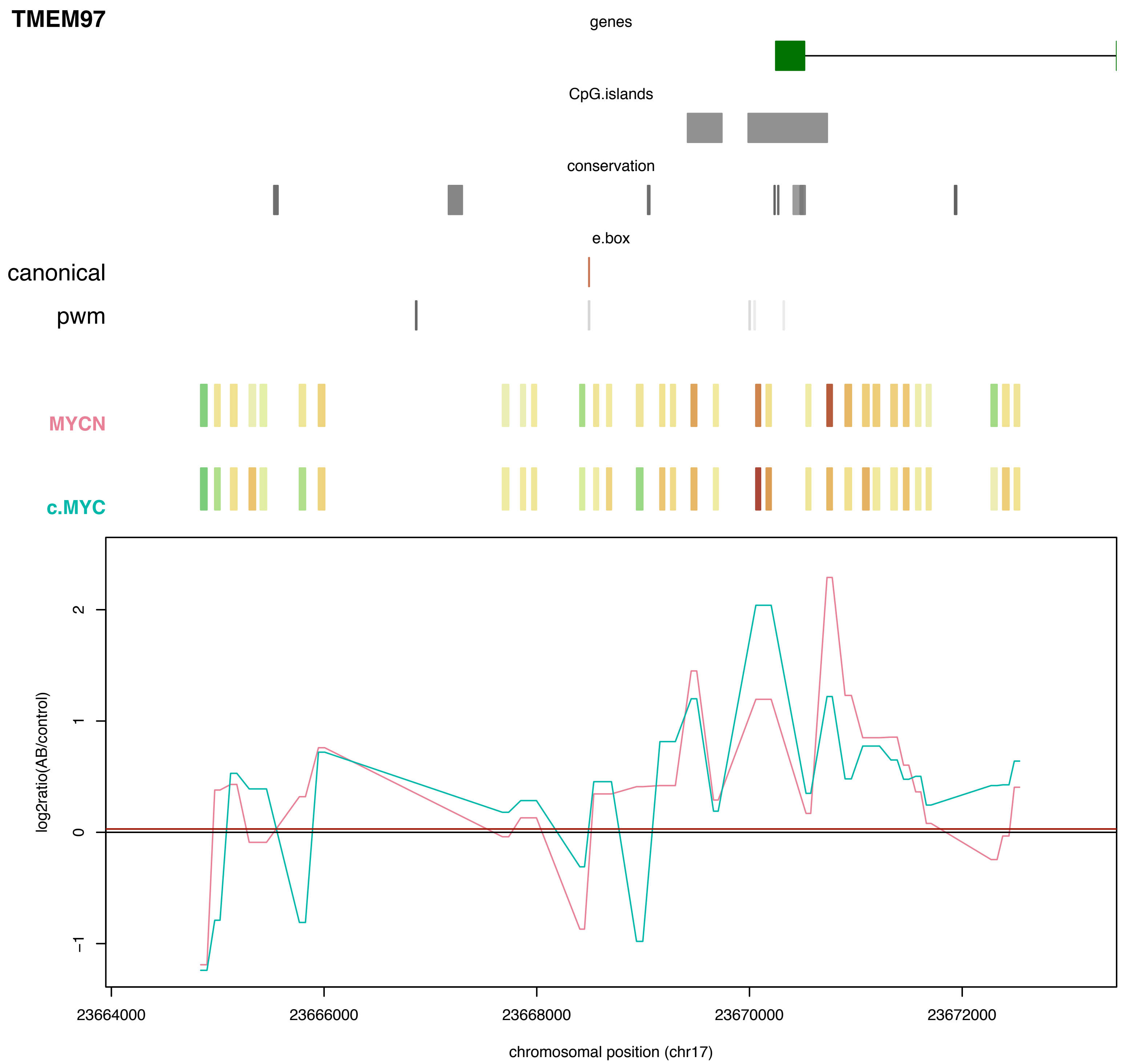

TMEM97
genes
CpG.islands
conservation
e.box
canonical
pwm
MYCN
c.MYC
log2ratio(AB/control)
2
1
0
−1
23664000
23666000
23668000
23670000
23672000
chromosomal position (chr17)

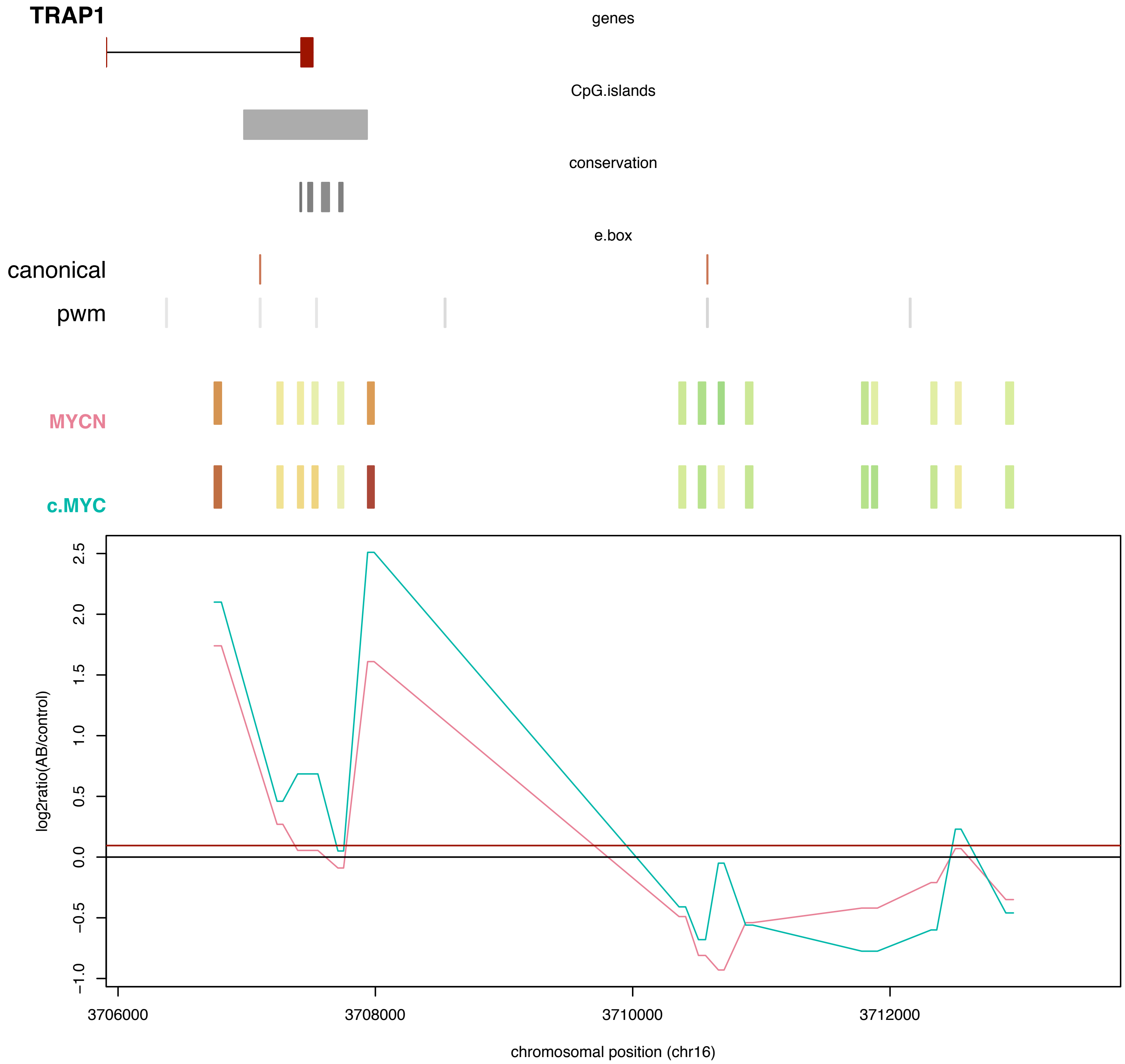
TRAP1
genes
CpG.islands
conservation
e.box
canonical
pwm
MYCN
c.MYC
log2ratio(AB/control)
2.5
2.0
1.5
1.0
0.5
0.0
−0.5
−1.0
3706000
3708000
3710000
3712000
chromosomal position (chr16)

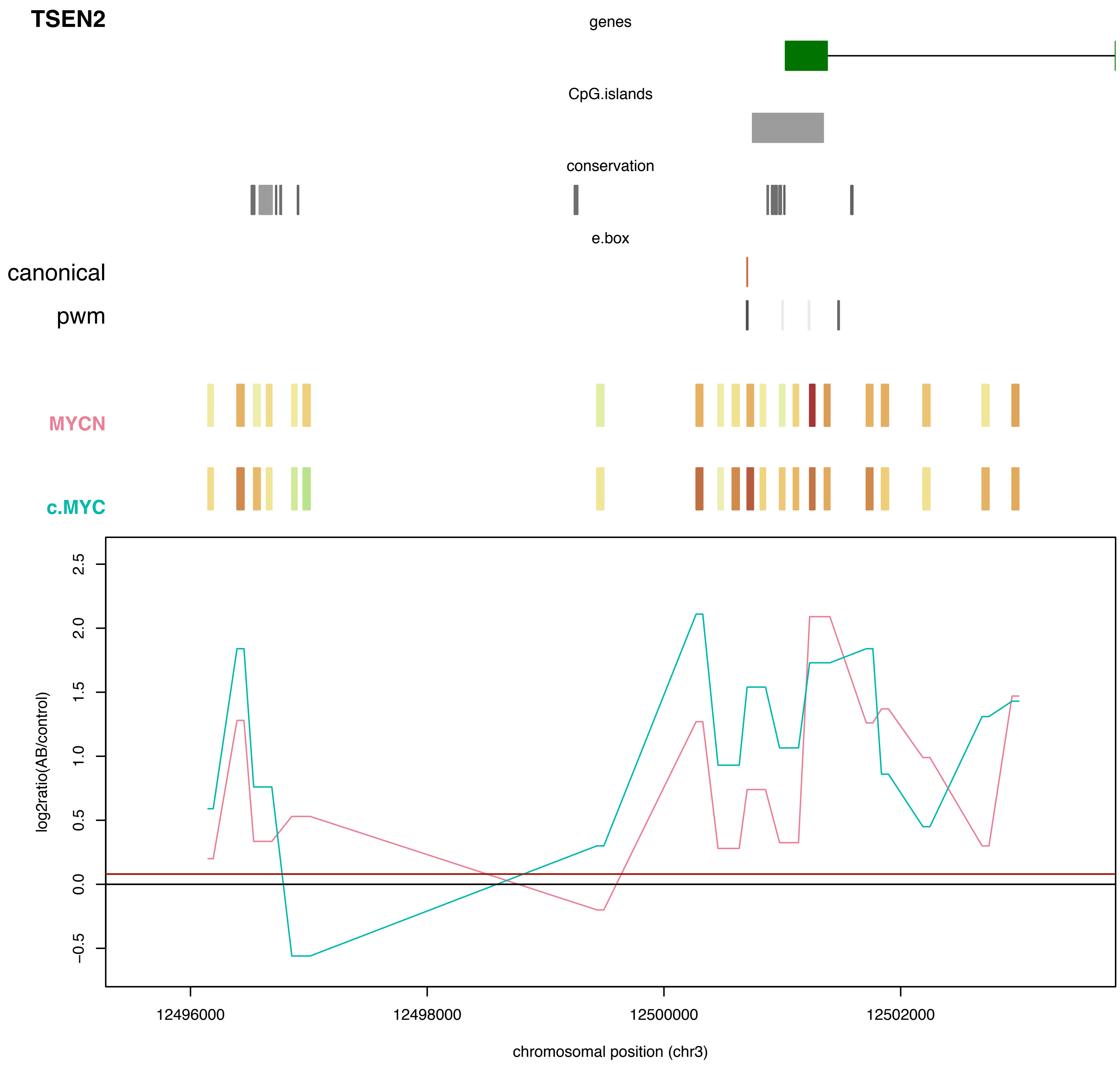

TSEN2
genes
CpG.islands
conservation
e.box
canonical
pwm
MYCN
c.MYC
log2ratio(AB/control)
2.5
2.0
1.5
1.0
0.5
0.0
−0.5
12496000
12498000
12500000
12502000
chromosomal position (chr3)

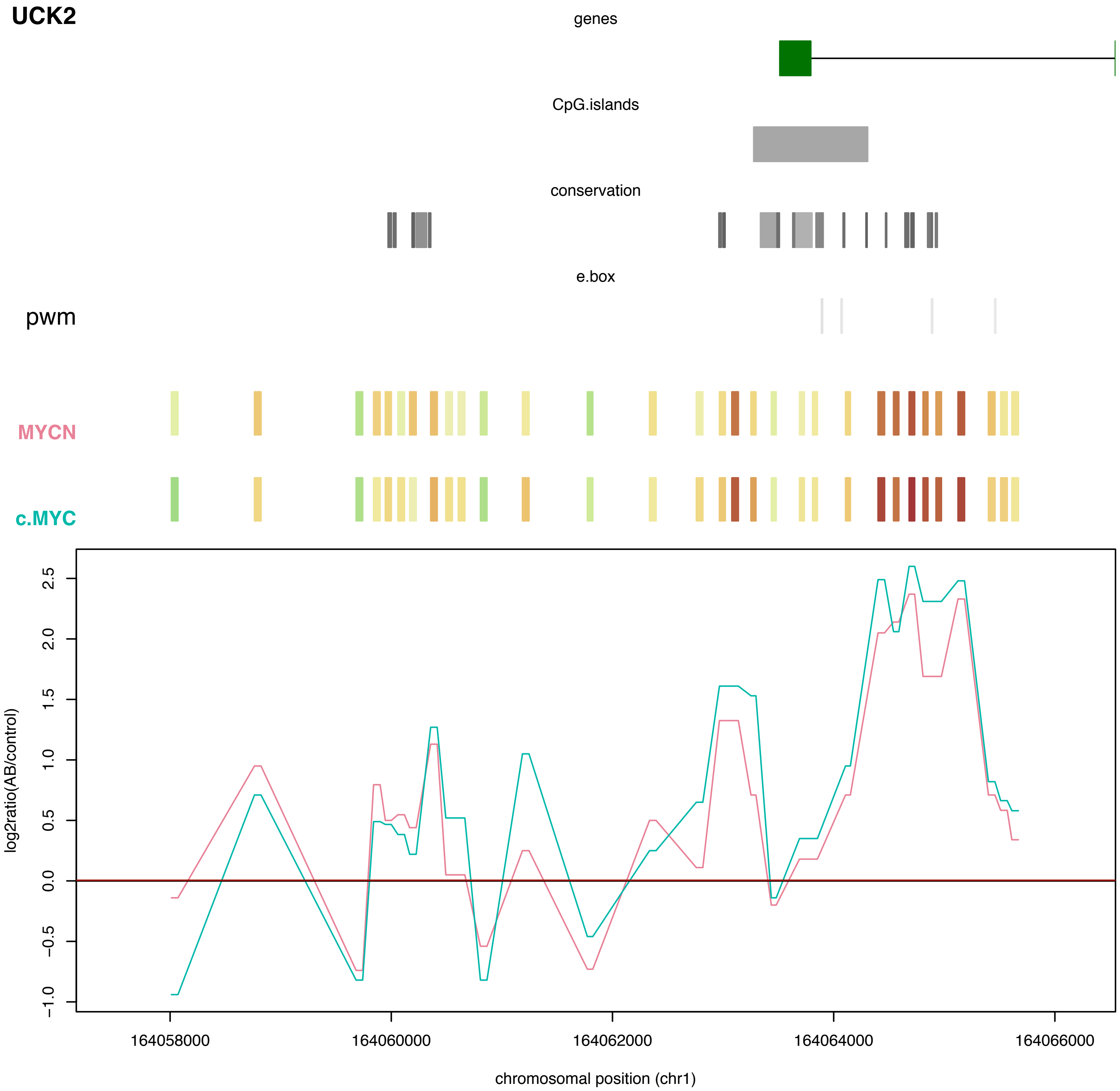

UCK2
genes
CpG.islands
conservation
e.box
pwm
MYCN
c.MYC
log2ratio(AB/control)
−1.0
−0.5
0.0
0.5
1.0
1.5
2.0
2.5
164058000
164060000
164062000
164064000
164066000
chromosomal position (chr1)

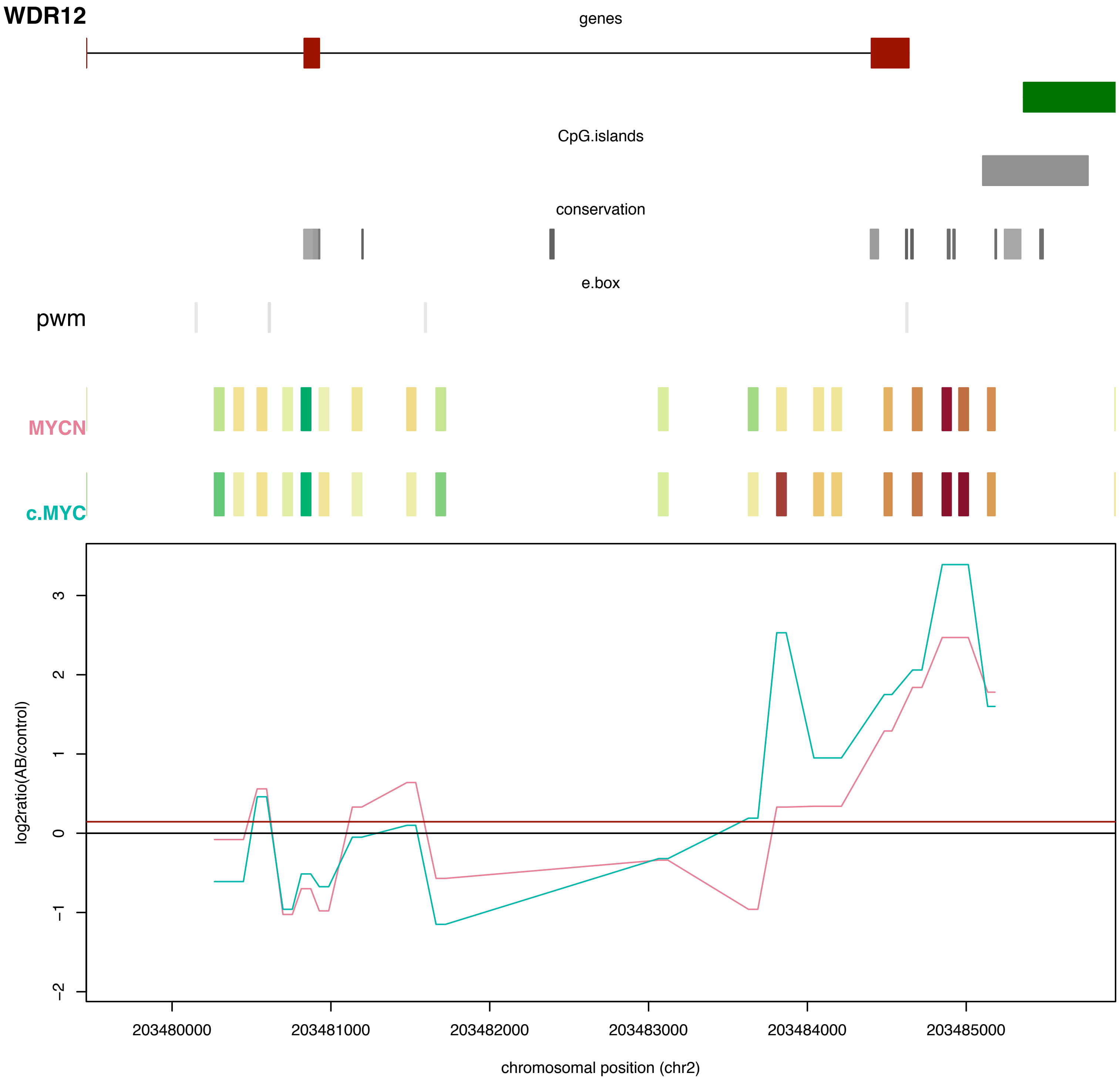

WDR12
genes
CpG.islands
conservation
e.box
pwm
MYCN
c.MYC
log2ratio(AB/control)
3
2
1
0
-1
-2
203480000
203481000
203482000
203483000
203484000
203485000
chromosomal position (chr2)

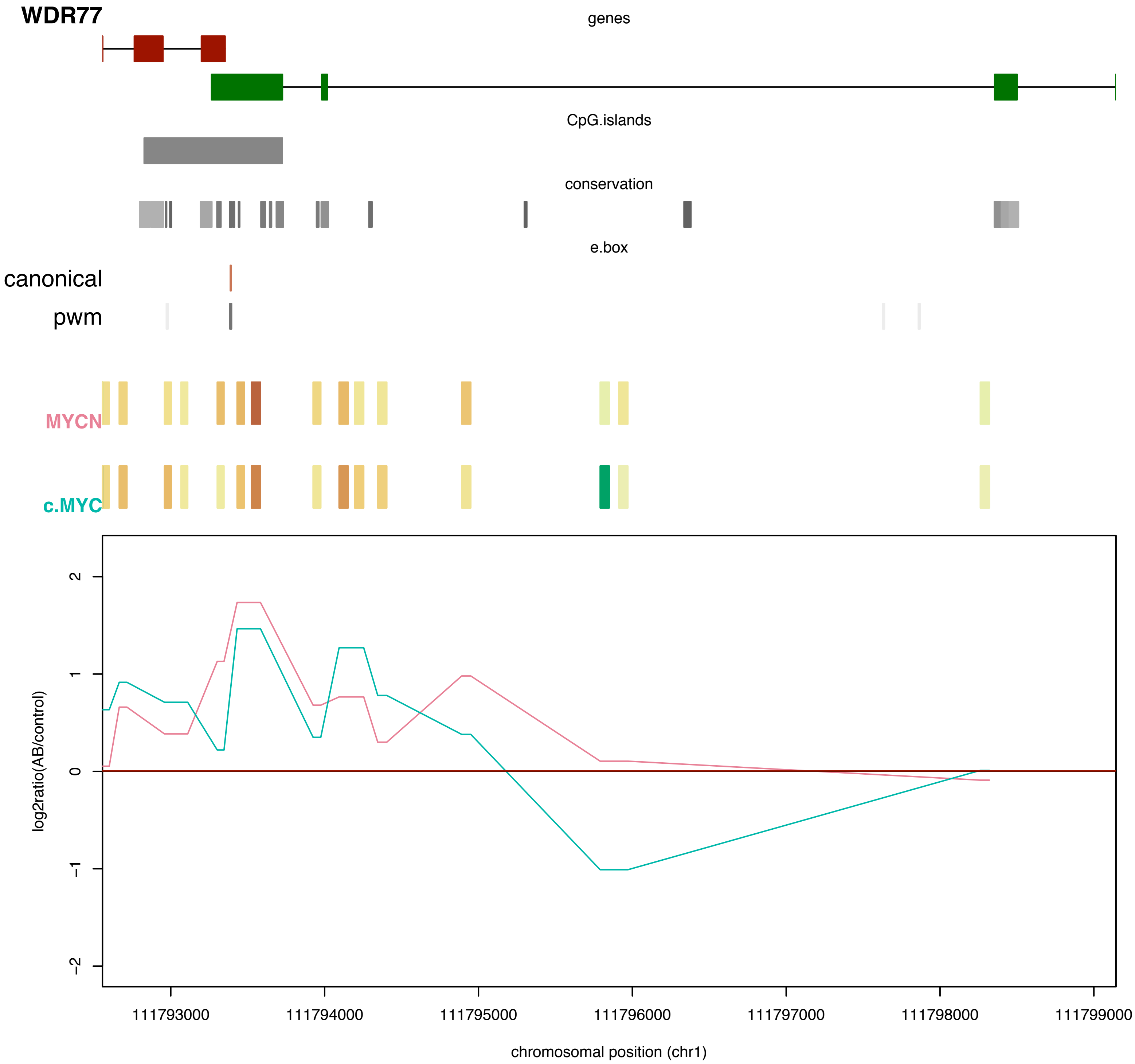

WDR77
genes
CpG.islands
conservation
e.box
canonical
pwm
MYCN
c.MYC
log2ratio(AB/control)
2
1
0
−1
−2
111793000
111794000
111795000
111796000
111797000
111798000
111799000
chromosomal position (chr1)

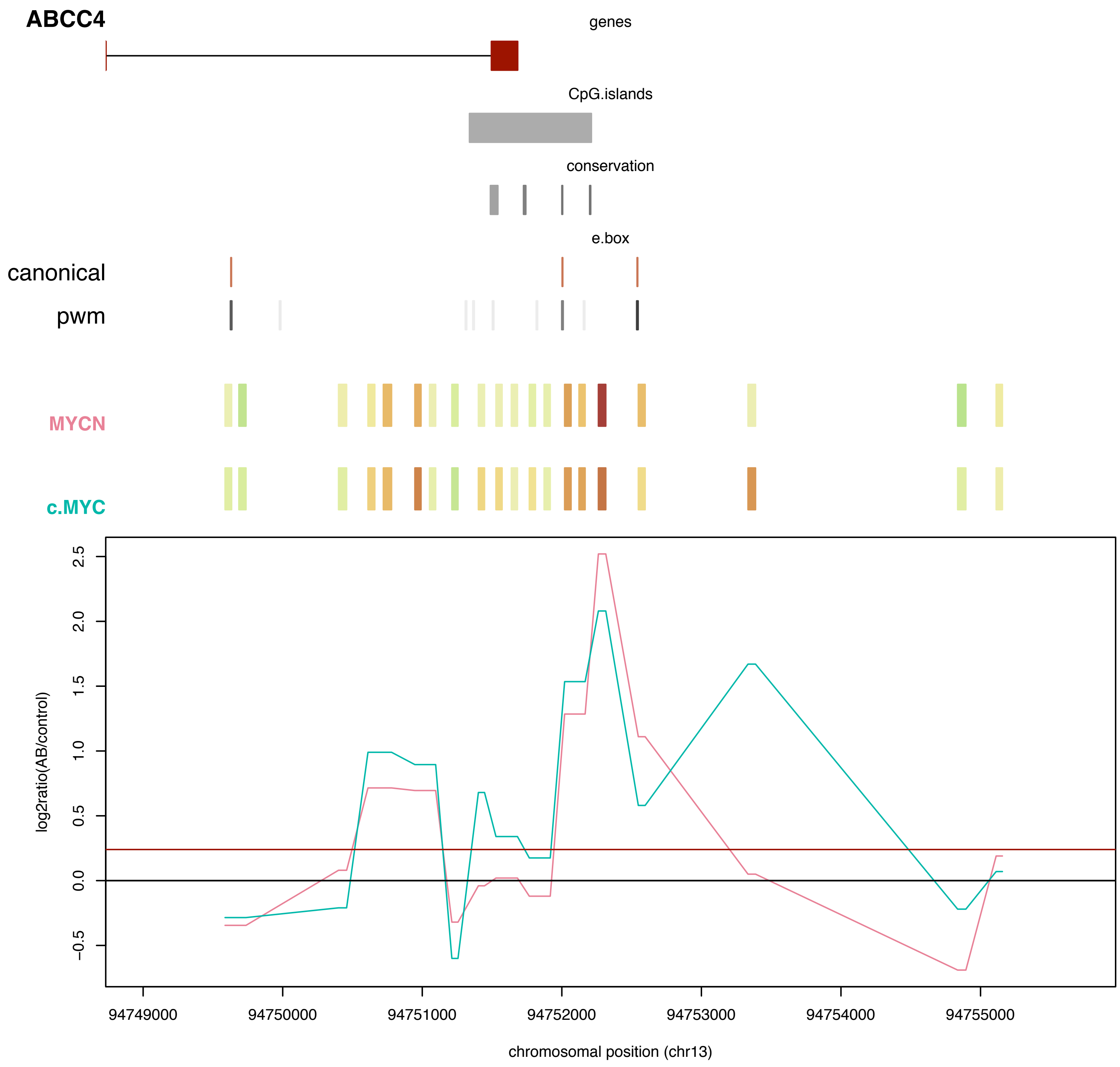

ABCC4
genes
CpG.islands
conservation
e.box
canonical
pwm
MYCN
c.MYC
log2ratio(AB/control)
−0.5
0.0
0.5
1.0
1.5
2.0
2.5
94749000
94750000
94751000
94752000
94753000
94754000
94755000
chromosomal position (chr13)

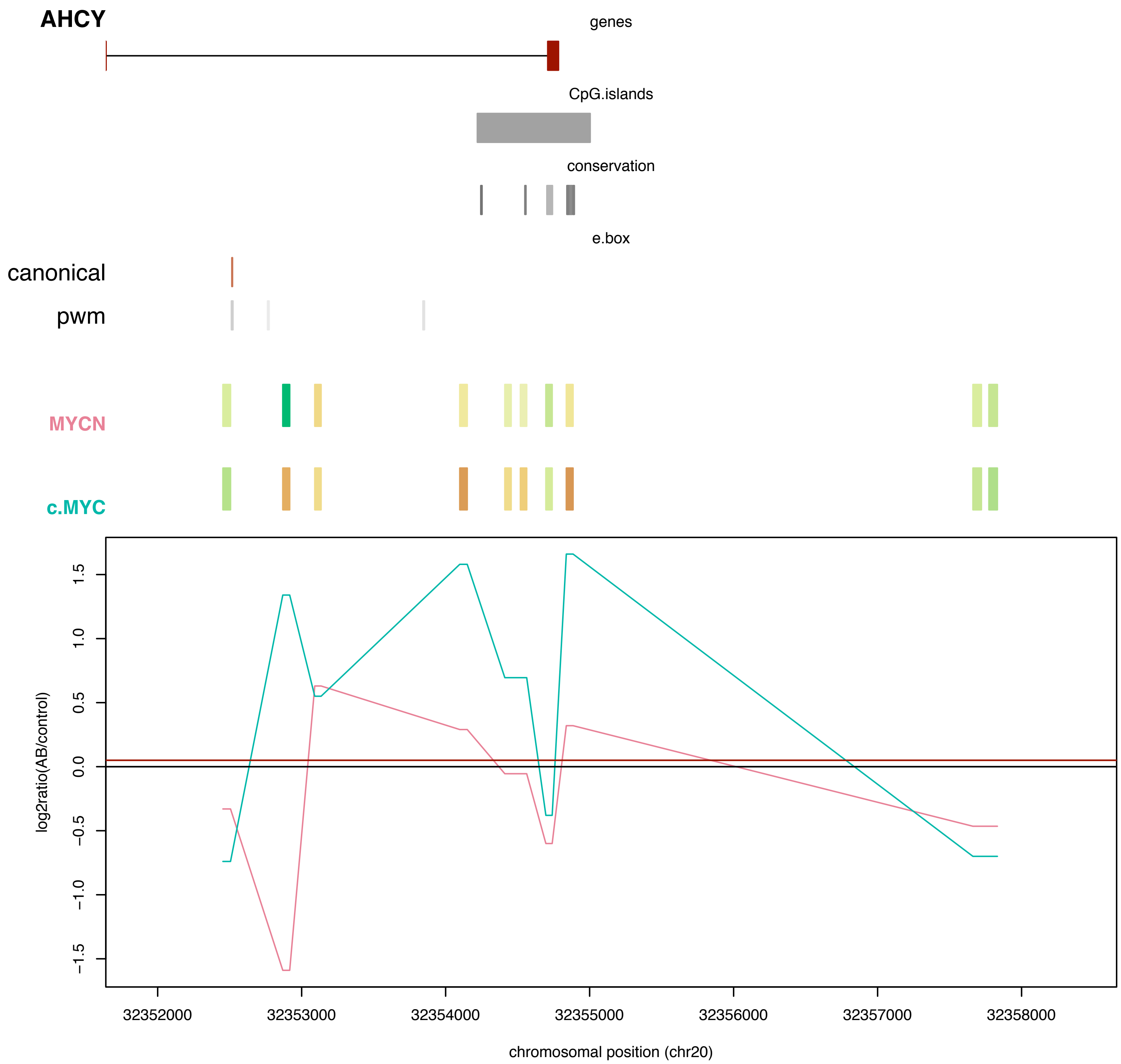
AHCY
genes
CpG.islands
conservation
e.box
canonical
pwm
MYCN
c.MYC
log2ratio(AB/control)
1.5
1.0
0.5
0.0
−0.5
−1.0
−1.5
32352000
32353000
32354000
32355000
32356000
32357000
32358000
chromosomal position (chr20)

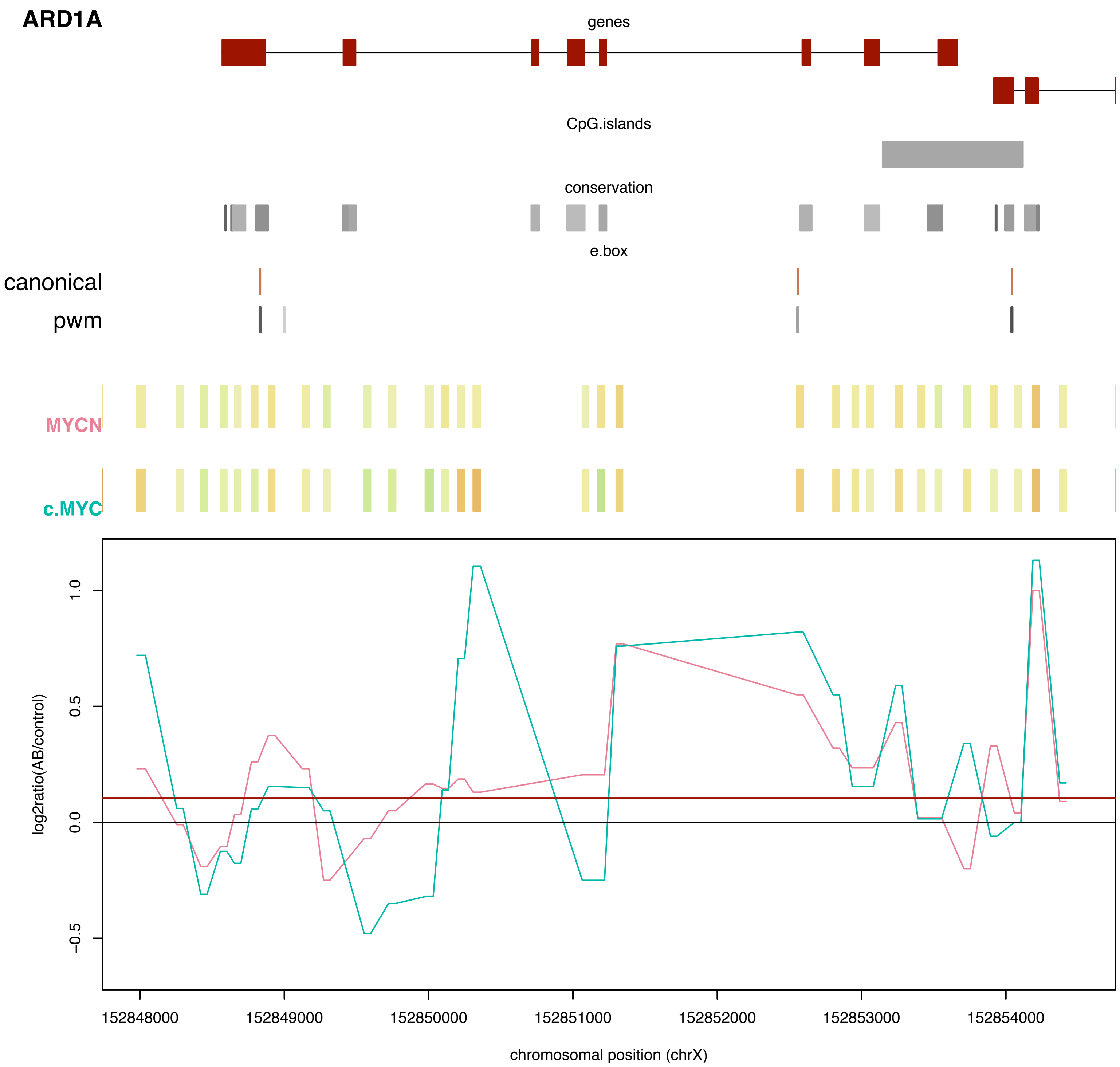
ARD1A
genes
CpG.islands
conservation
e.box
canonical
pwm
MYCN
c.MYC
log2ratio(AB/control)
1.0
0.5
0.0
−0.5
152848000
152849000
152850000
152851000
152852000
152853000
152854000
chromosomal position (chrX)

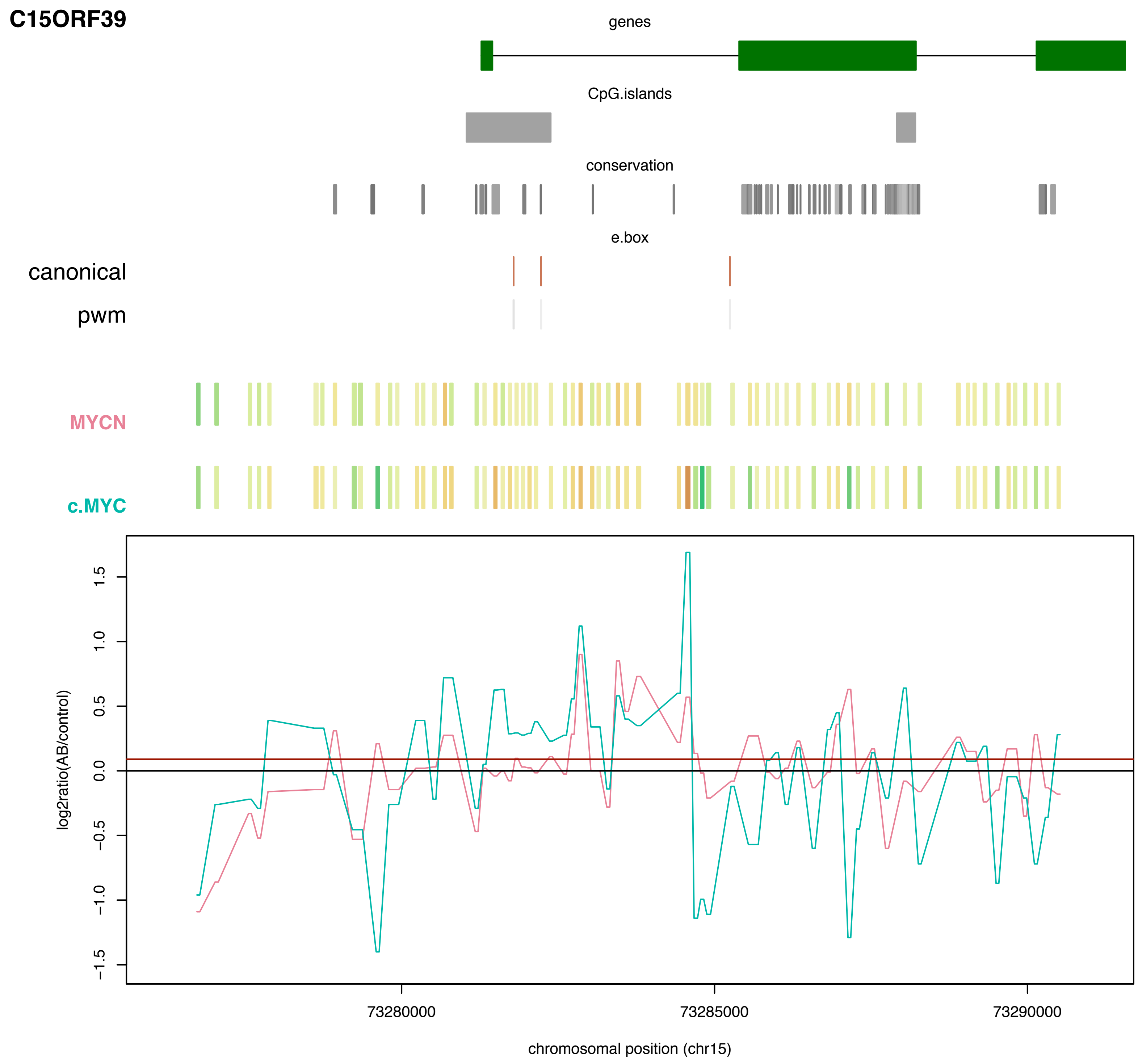
C15ORF39
genes
CpG.islands
conservation
e.box
canonical
pwm
MYCN
c.MYC
log2ratio(AB/control)
1.5
1.0
0.5
0.0
−0.5
−1.0
−1.5
73280000
73285000
73290000
chromosomal position (chr15)

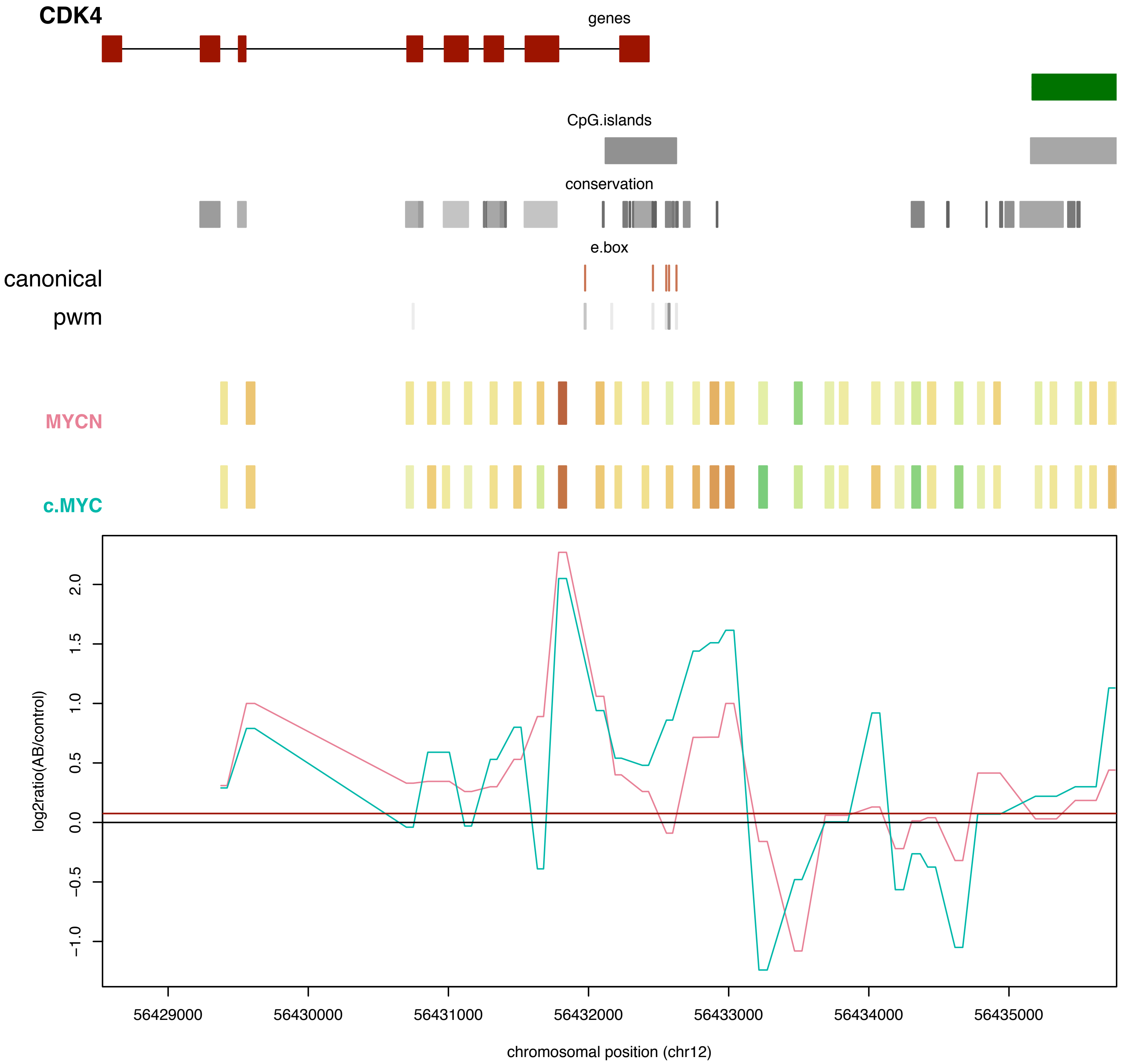
CDK4
genes
CpG.islands
conservation
e.box
canonical
pwm
MYCN
c.MYC
log2ratio(AB/control)
2.0
1.5
1.0
0.5
0.0
−0.5
−1.0
56429000
56430000
56431000
56432000
56433000
56434000
56435000
chromosomal position (chr12)

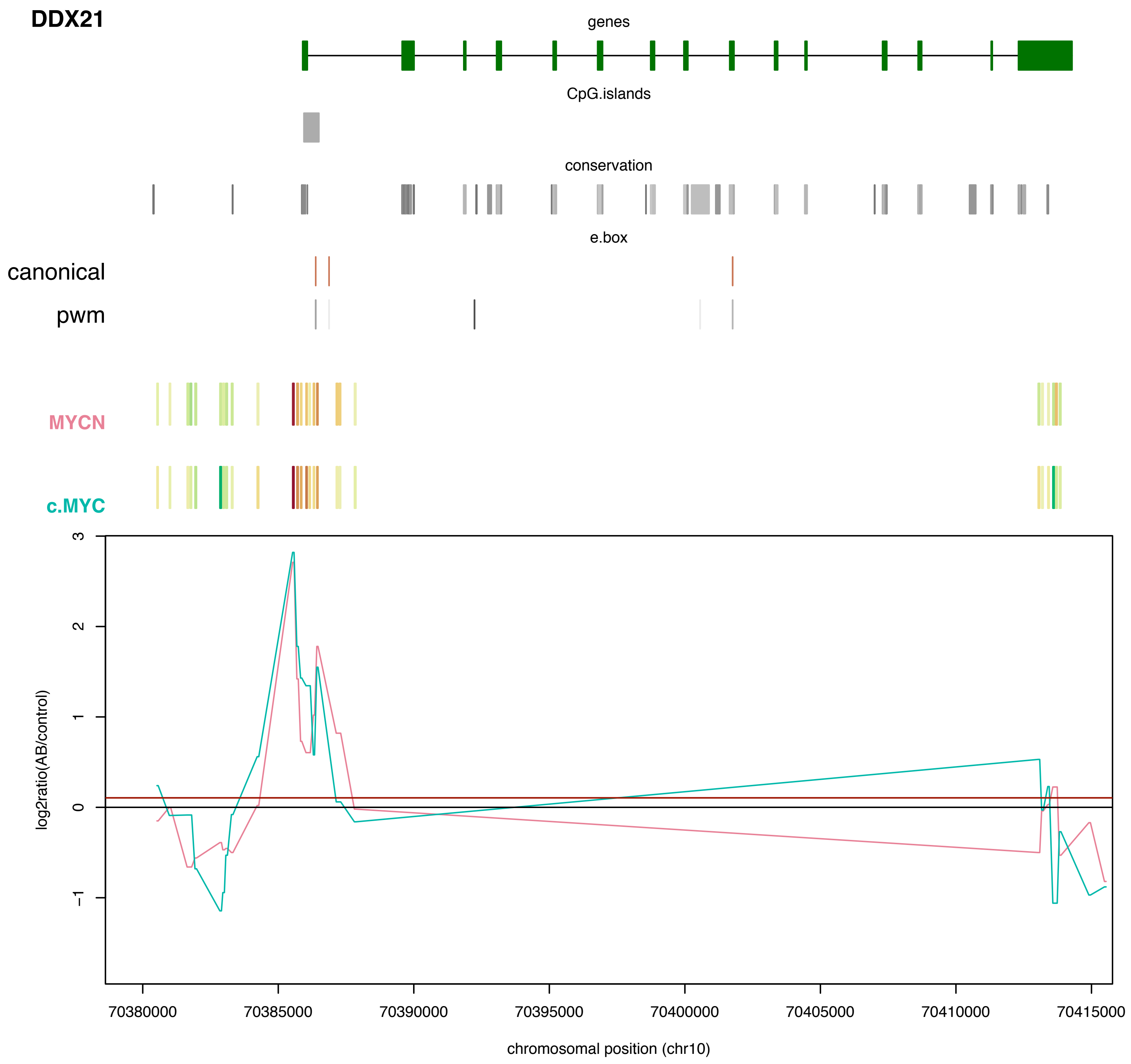

DDX21
genes
CpG.islands
conservation
e.box
canonical
pwm
MYCN
c.MYC
log2ratio(AB/control)
3
2
1
0
−1
70380000
70385000
70390000
70395000
70400000
70405000
70410000
70415000
chromosomal position (chr10)

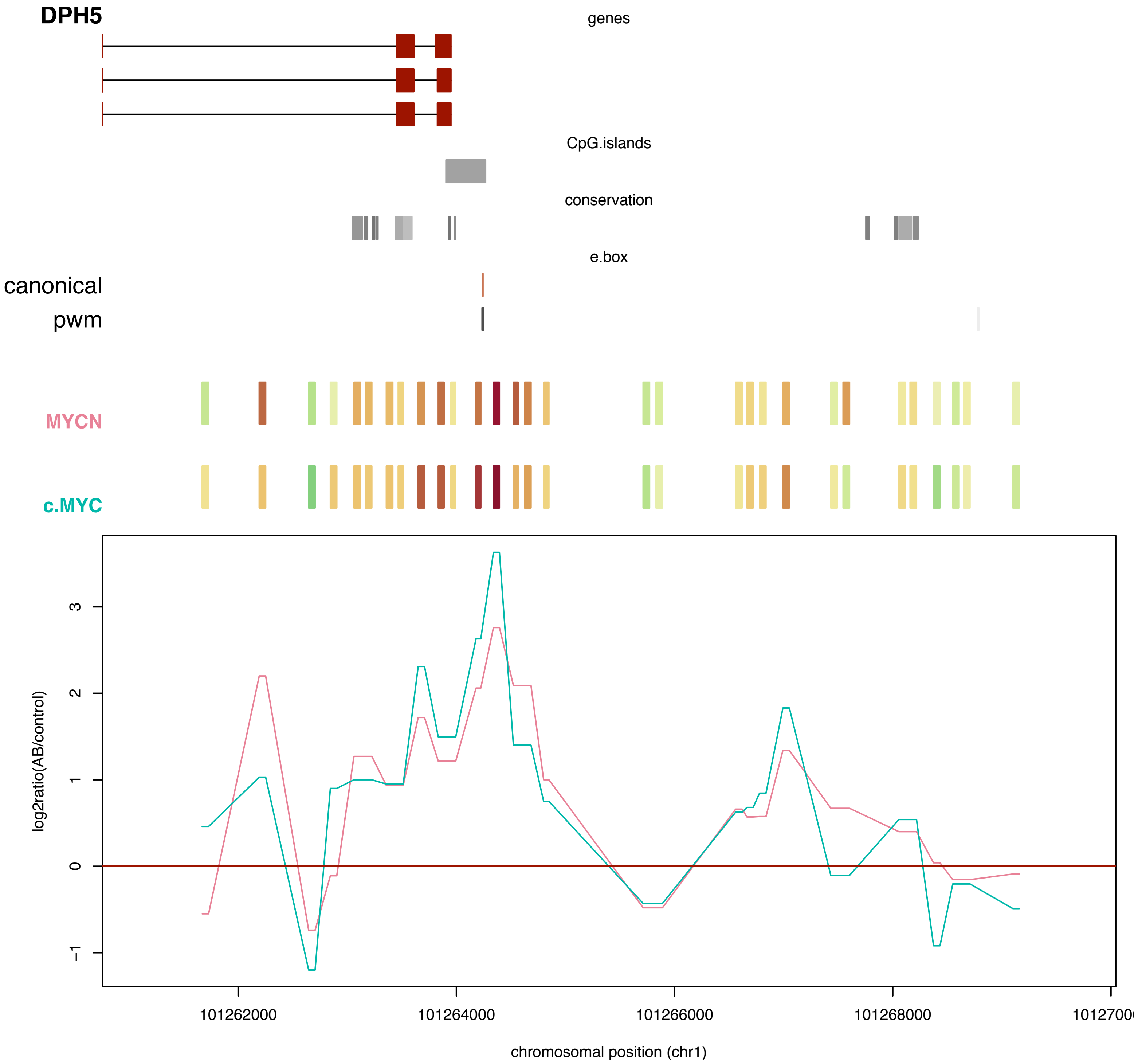

DPH5
genes
CpG.islands
conservation
e.box
canonical
pwm
MYCN
c.MYC
log2ratio(AB/control)
3
2
1
0
−1
101262000
101264000
101266000
101268000
chromosomal position (chr1)

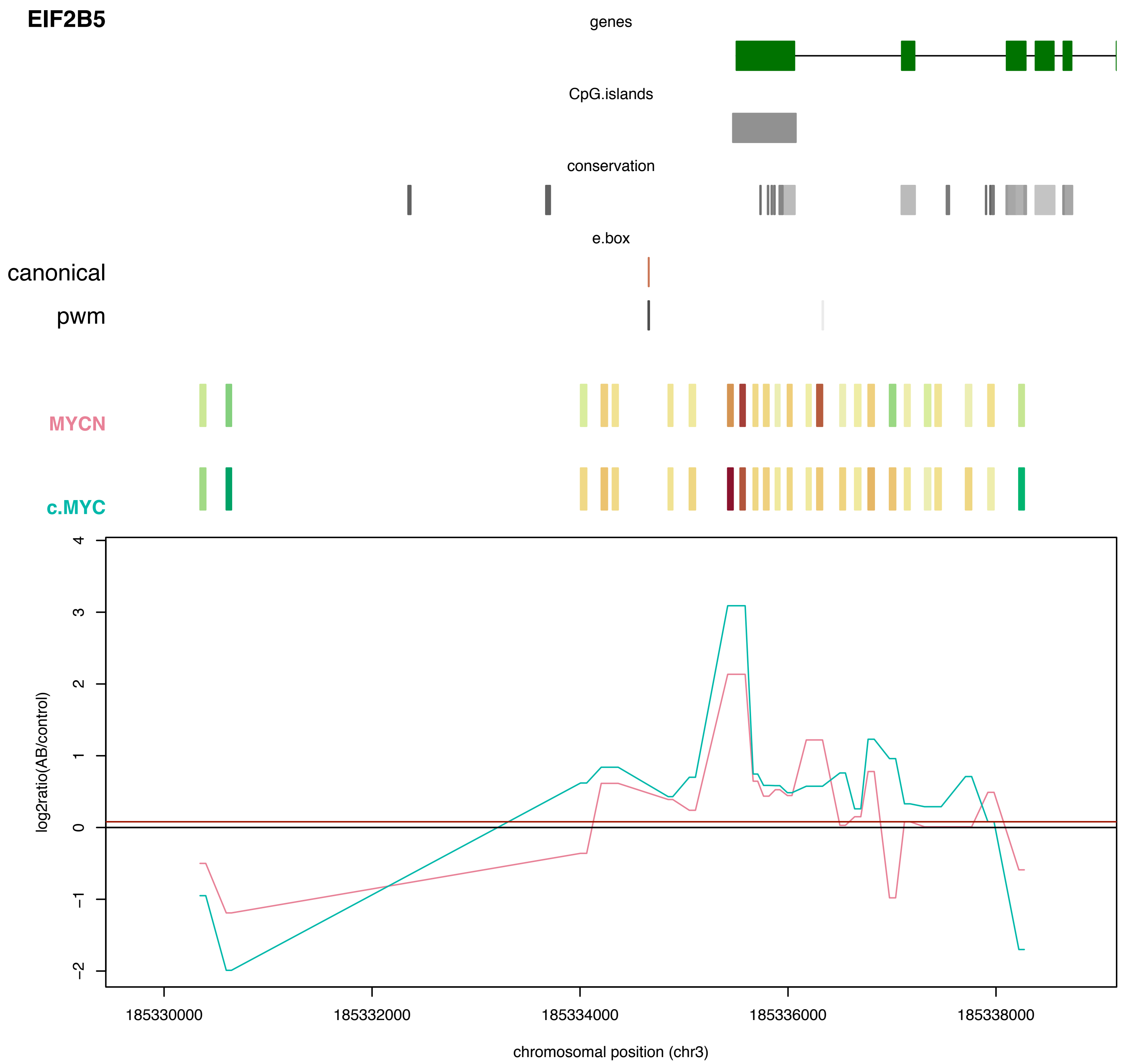

EIF2B5
genes
CpG.islands
conservation
e.box
canonical
pwm
MYCN
c.MYC
log2ratio(AB/control)
4
3
2
1
0
−1
−2
185330000
185332000
185334000
185336000
185338000
chromosomal position (chr3)

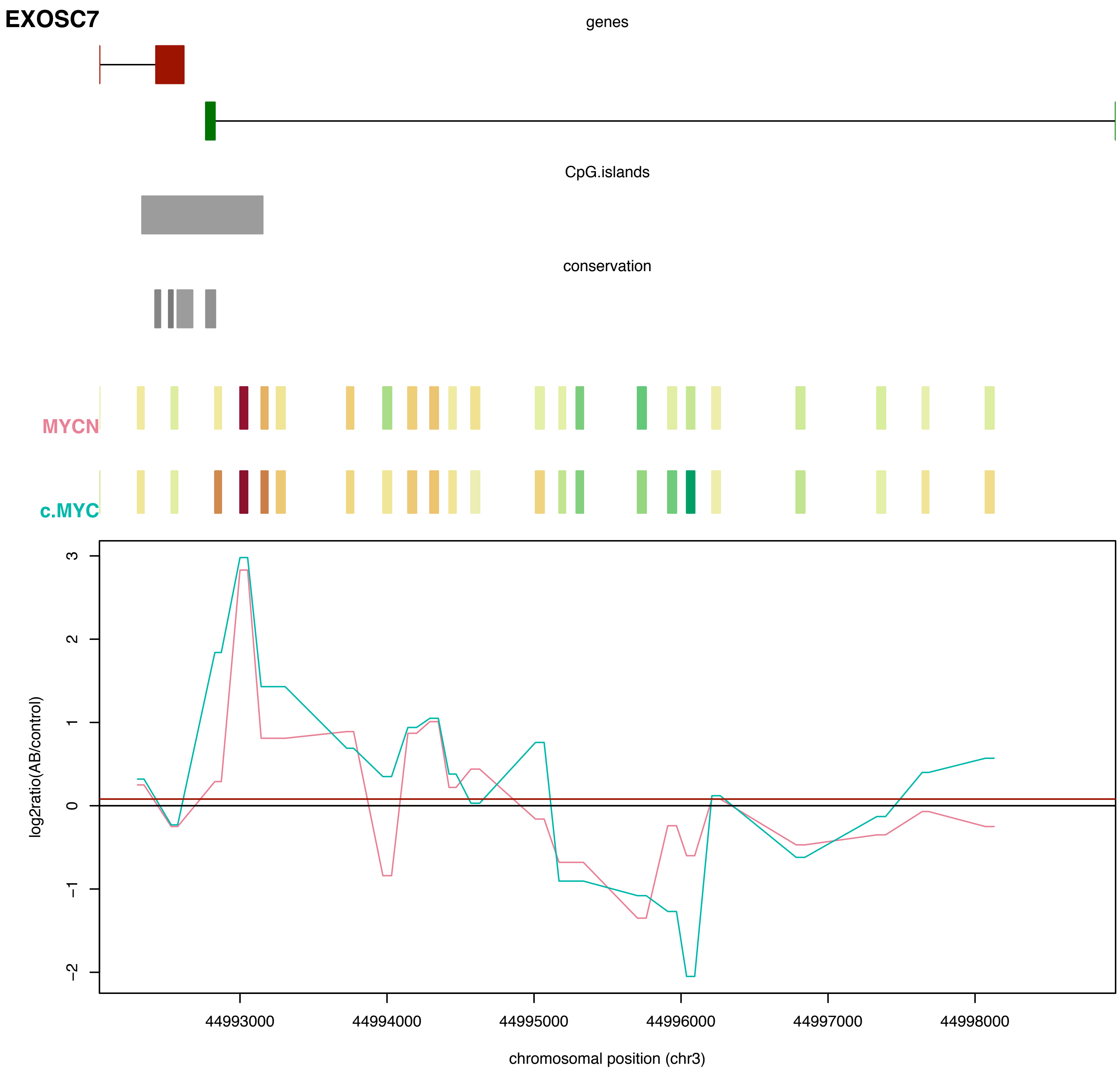

EXOSC7
genes
CpG.islands
conservation
MYCN
c.MYC
log2ratio(AB/control)
3
2
1
0
−1
−2
44993000
44994000
44995000
44996000
44997000
44998000
chromosomal position (chr3)

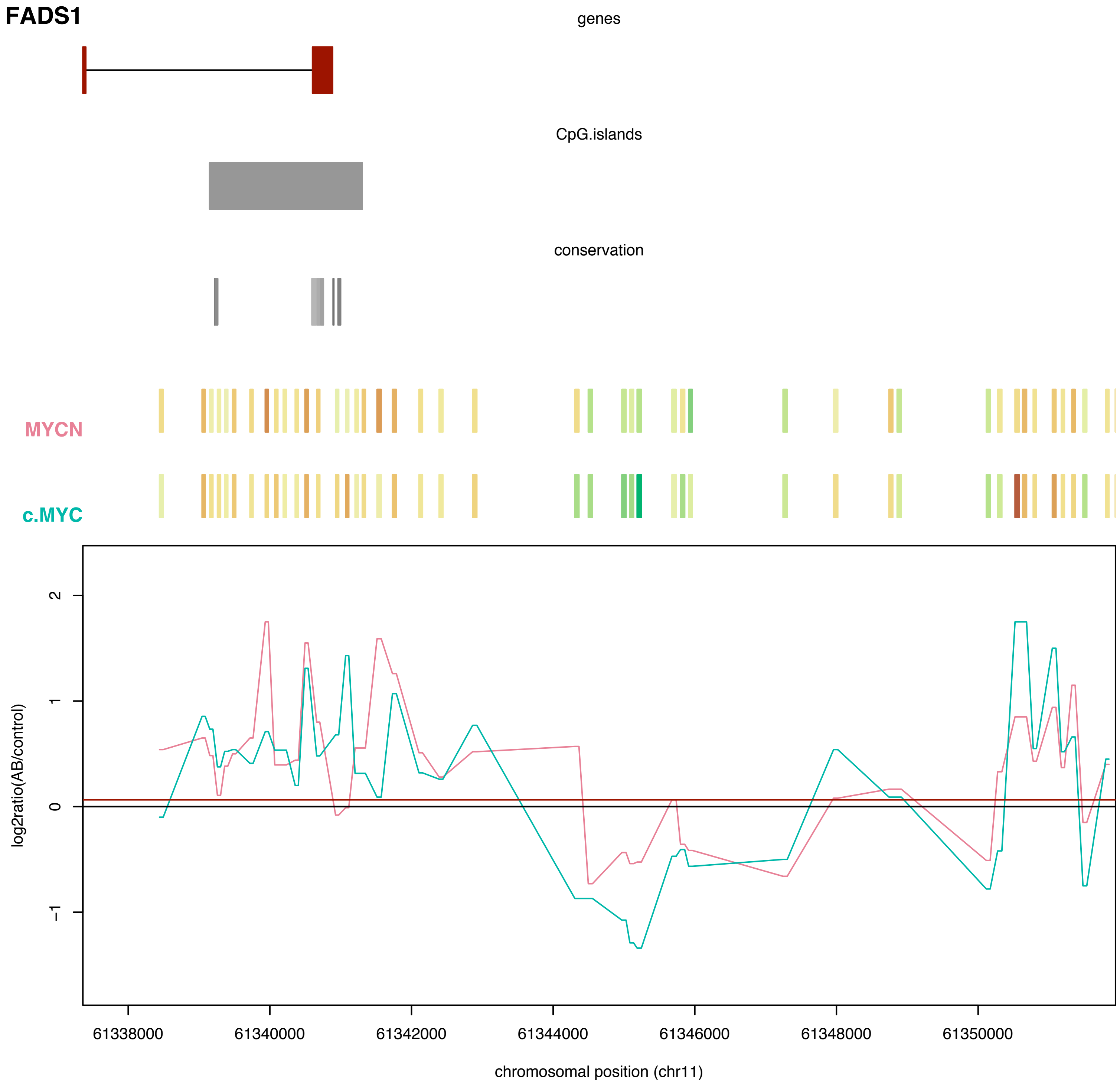

FADS1
genes
CpG.islands
conservation
MYCN
c.MYC
log2ratio(AB/control)
2
1
0
−1
61338000
61340000
61342000
61344000
61346000
61348000
61350000
chromosomal position (chr11)

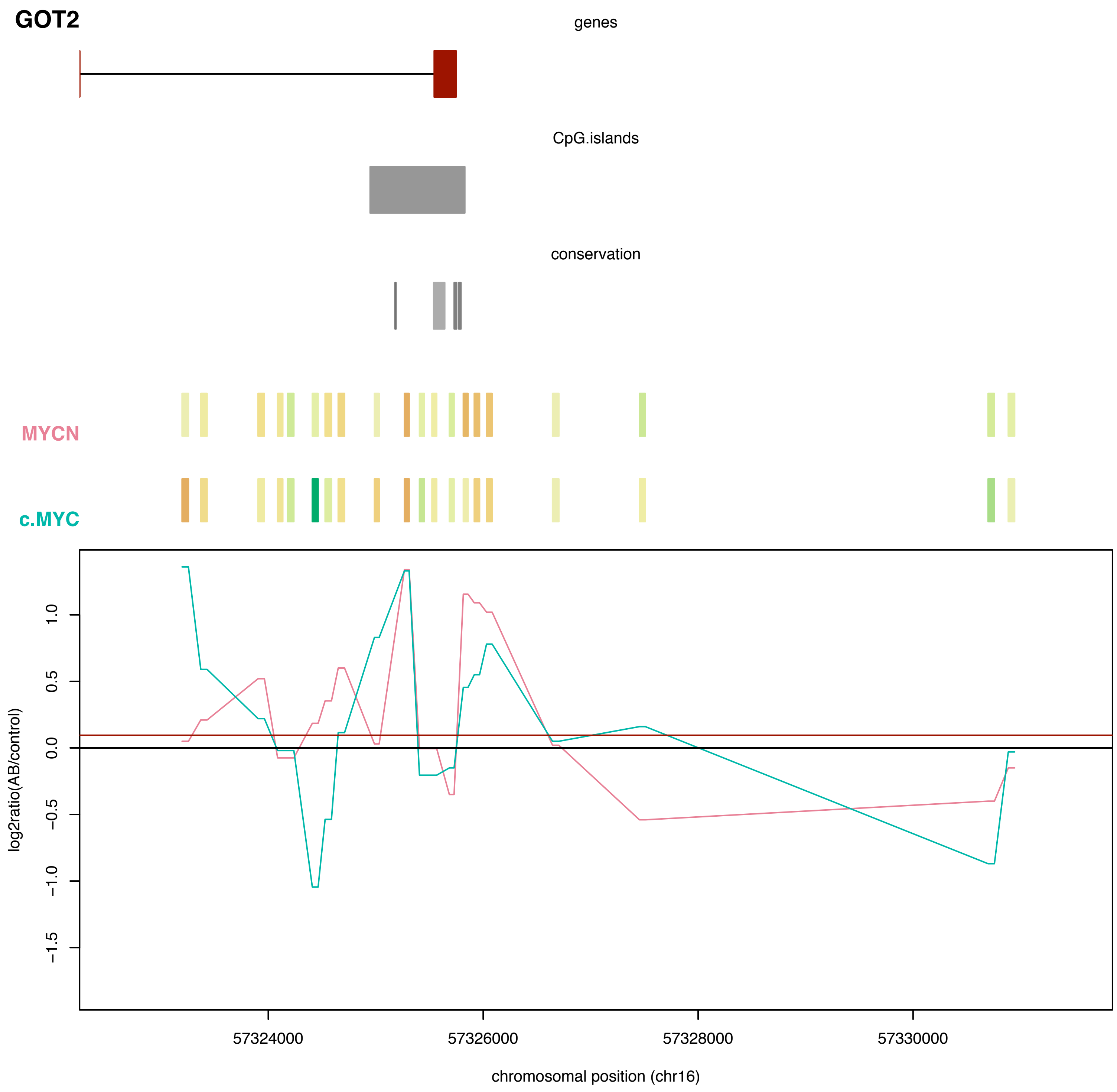
GOT2
genes
CpG.islands
conservation
MYCN
c.MYC
log2ratio(AB/control)
1.0
0.5
0.0
−0.5
−1.0
−1.5
57324000
57326000
57328000
57330000
chromosomal position (chr16)

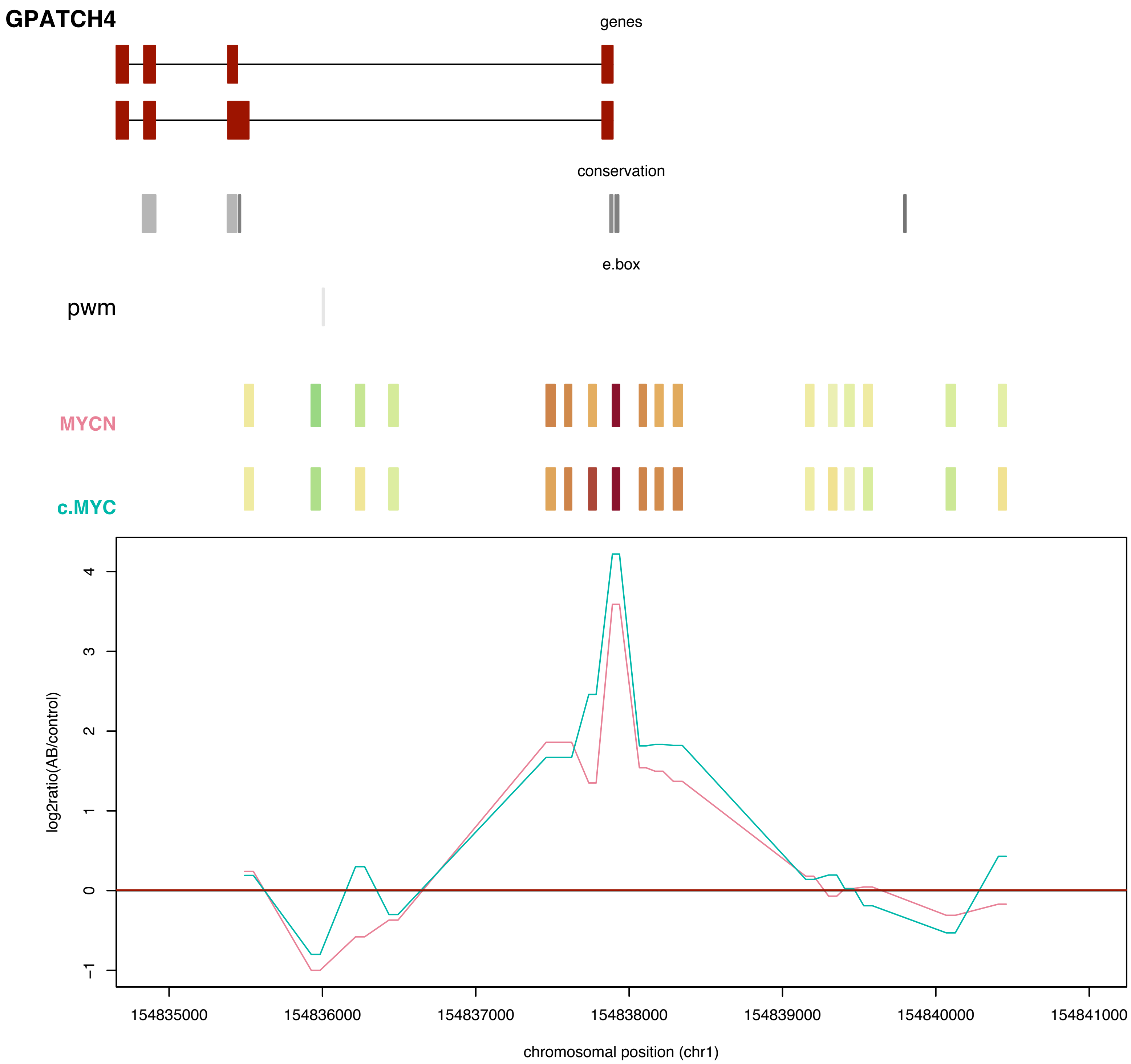

GPATCH4
genes
conservation
e.box
pwm
MYCN
c.MYC
log2ratio(AB/control)
4
3
2
1
0
−1
154835000
154836000
154837000
154838000
154839000
154840000
154841000
chromosomal position (chr1)

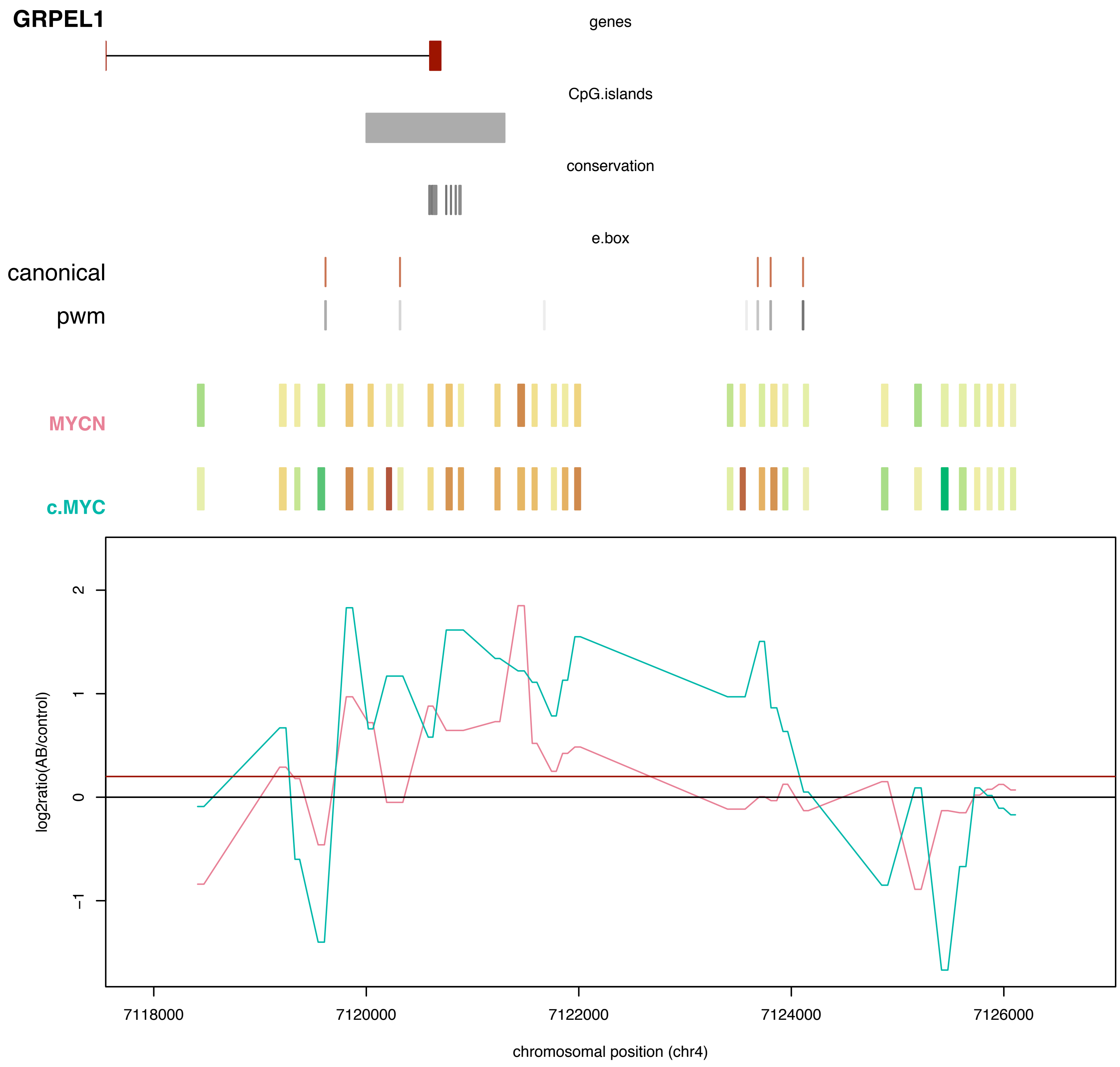

GRPEL1
genes
CpG.islands
conservation
e.box
canonical
pwm
MYCN
c.MYC
log2ratio(AB/control)
2
1
0
−1
7118000
7120000
7122000
7124000
7126000
chromosomal position (chr4)

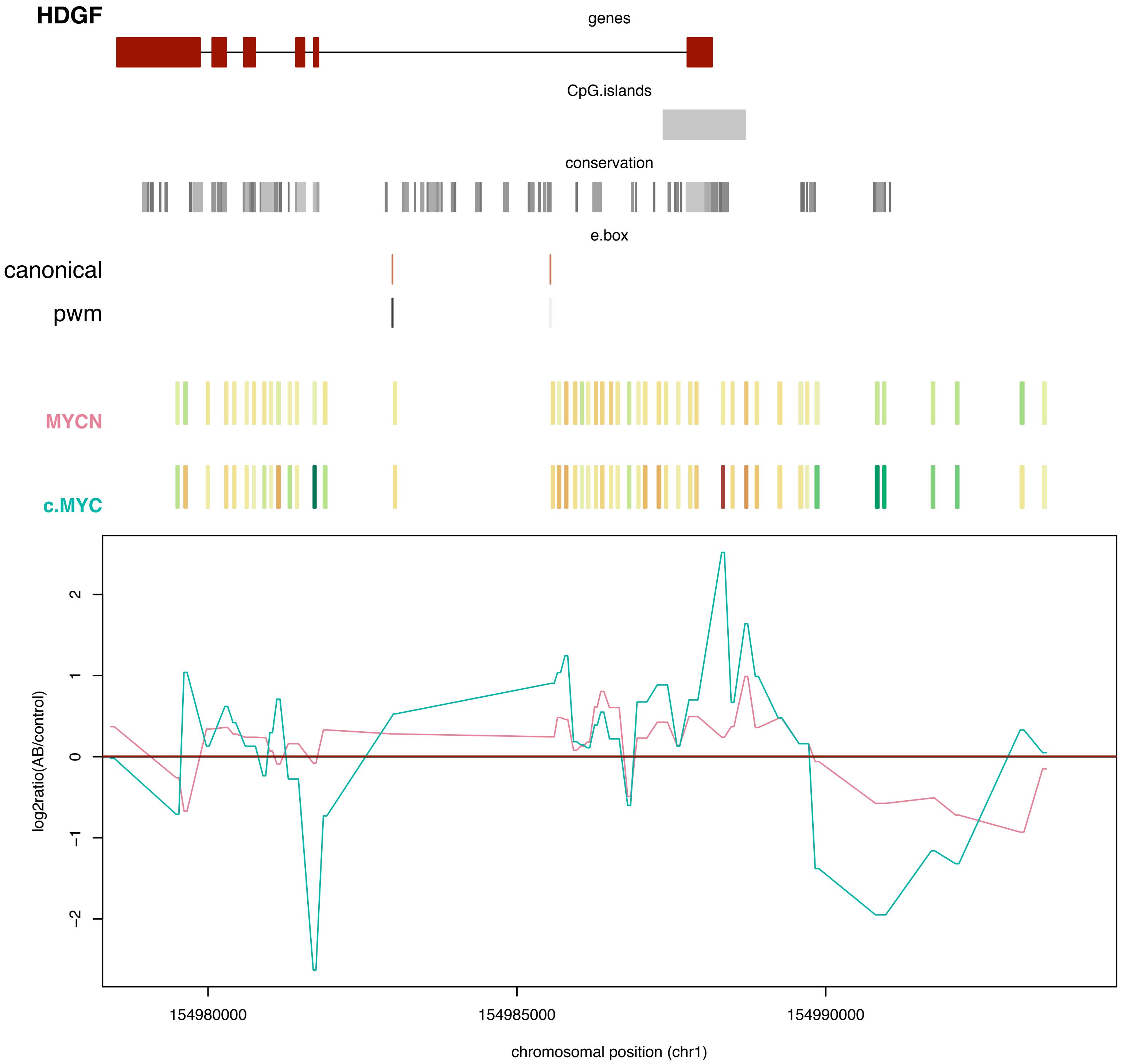

HDGF
genes
CpG.islands
conservation
e.box
canonical
pwm
MYCN
c.MYC
log2ratio(AB/control)
2
1
0
−1
−2
154980000
154985000
154990000
chromosomal position (chr1)

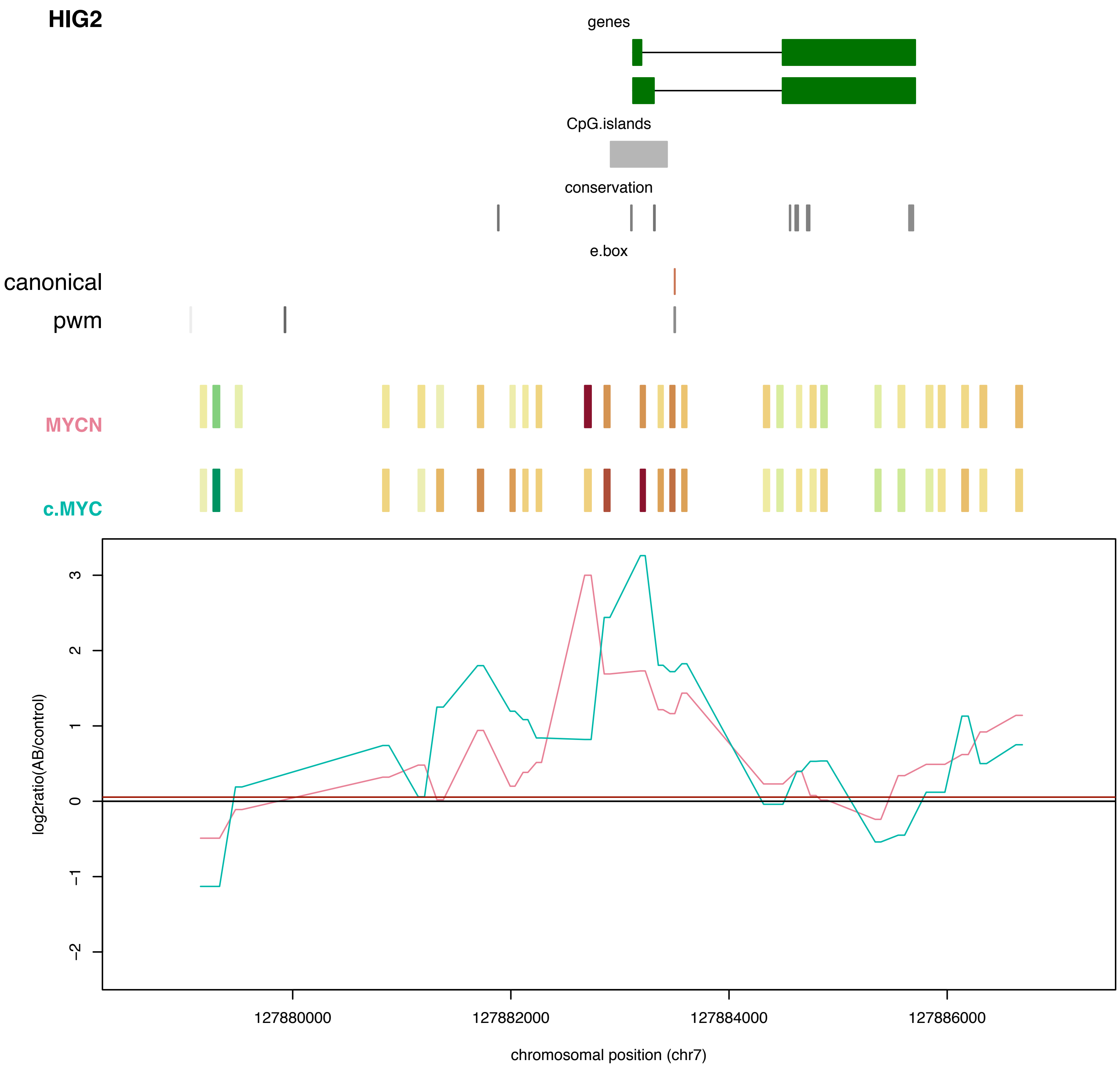
HIG2
genes
CpG.islands
conservation
e.box
canonical
pwm
MYCN
c.MYC
log2ratio(AB/control)
3
2
1
0
−1
−2
127880000
127882000
127884000
127886000
chromosomal position (chr7)

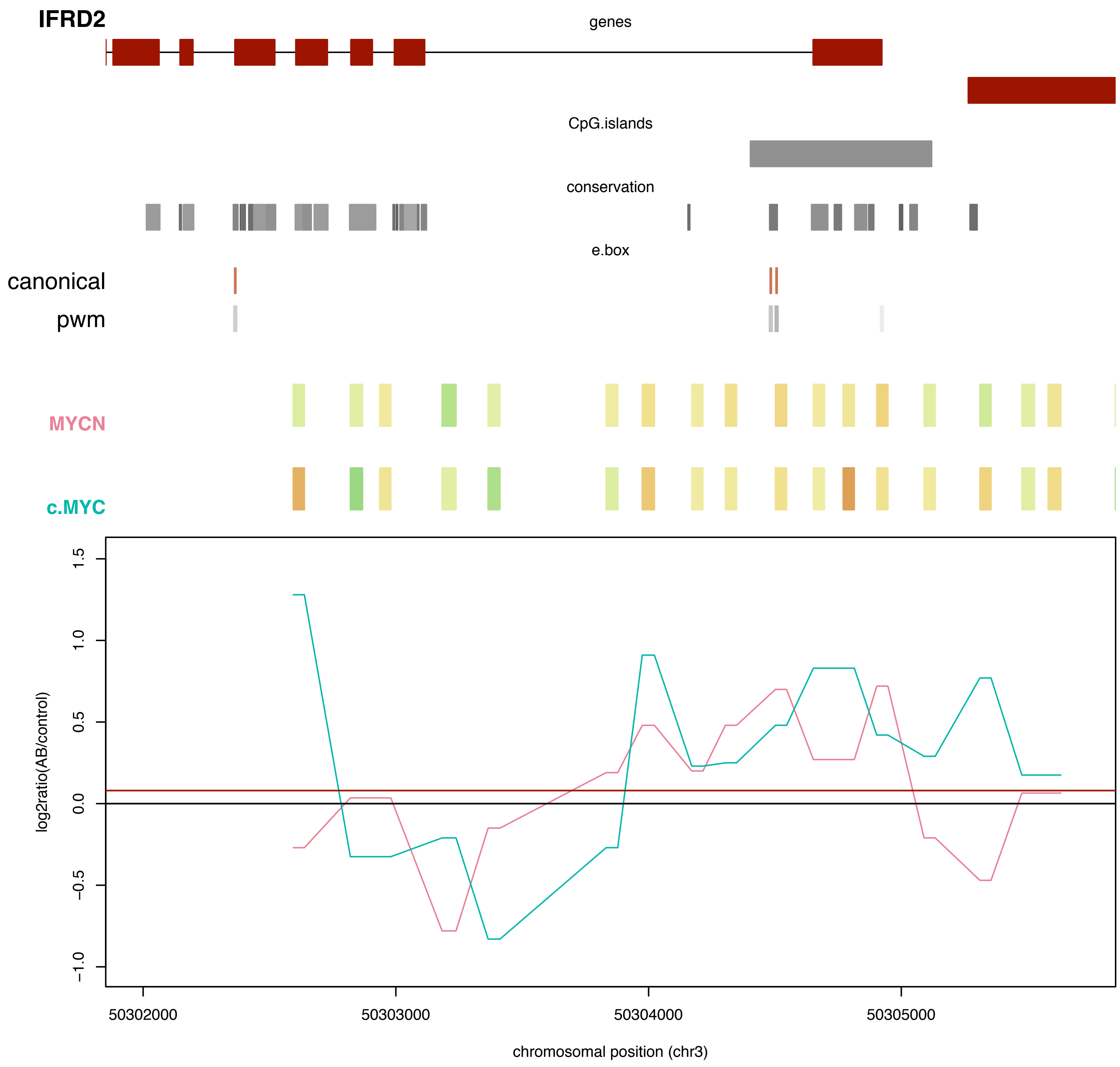

IFRD2
genes
CpG.islands
conservation
e.box
canonical
pwm
MYCN
c.MYC
log2ratio(AB/control)
1.5
1.0
0.5
0.0
−0.5
−1.0
50302000
50303000
50304000
50305000
chromosomal position (chr3)

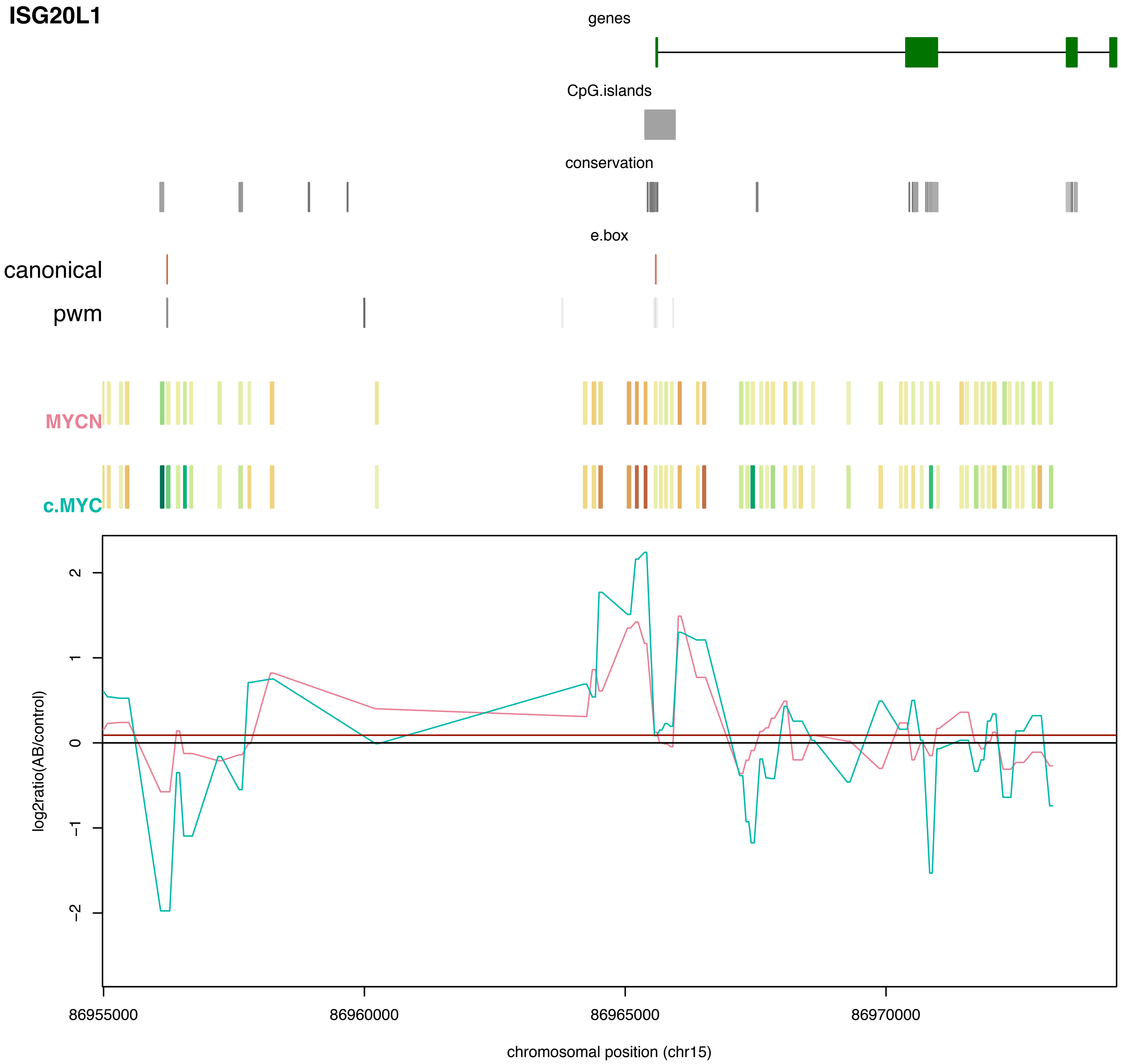

ISG20L1
genes
CpG.islands
conservation
e.box
canonical
pwm
MYCN
c.MYC
log2ratio(AB/control)
2
1
0
−1
−2
86955000
86960000
86965000
86970000
chromosomal position (chr15)

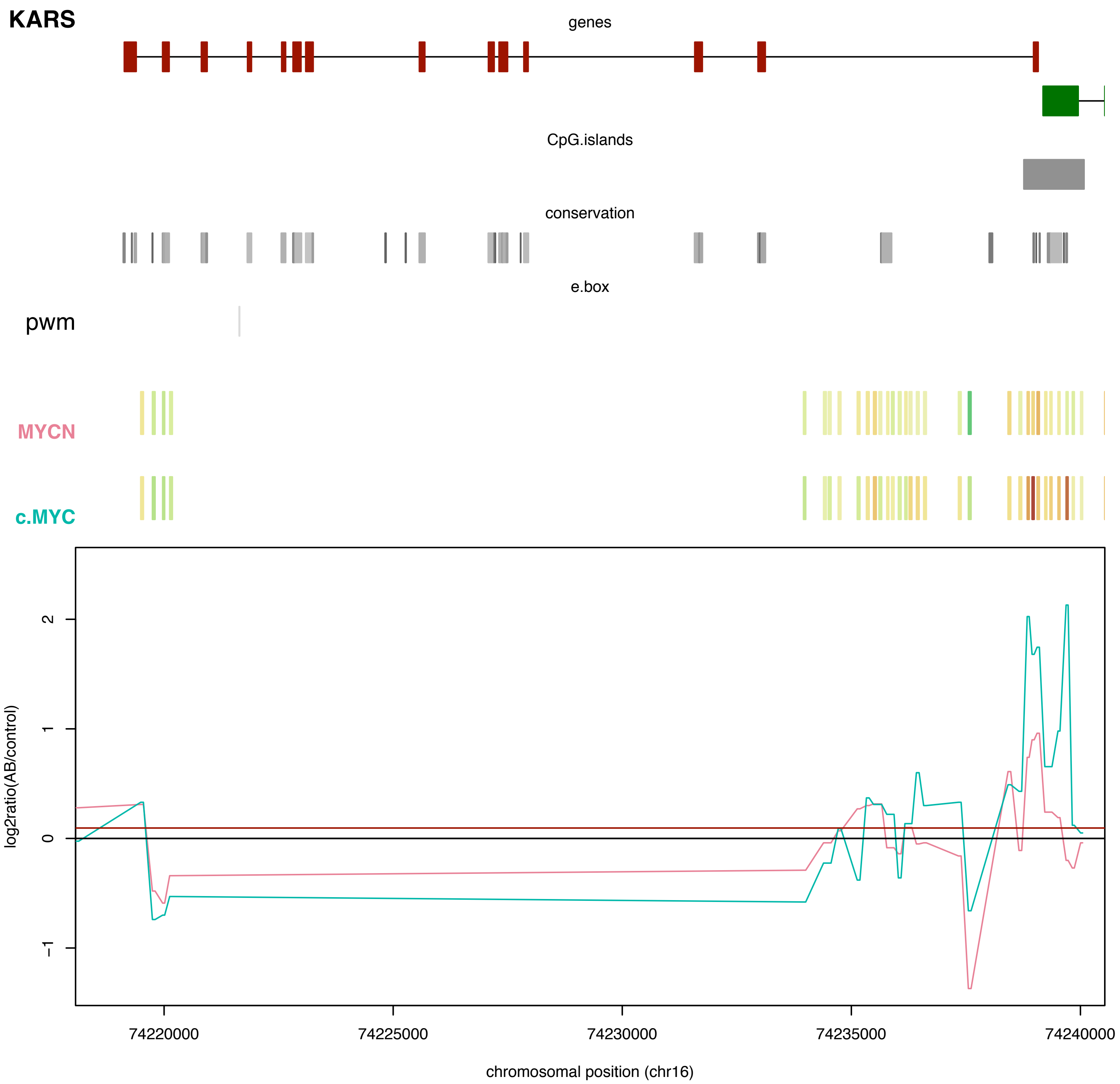
KARS
genes
CpG.islands
conservation
e.box
pwm
MYCN
c.MYC
log2ratio(AB/control)
2
1
0
−1
74220000
74225000
74230000
74235000
74240000
chromosomal position (chr16)

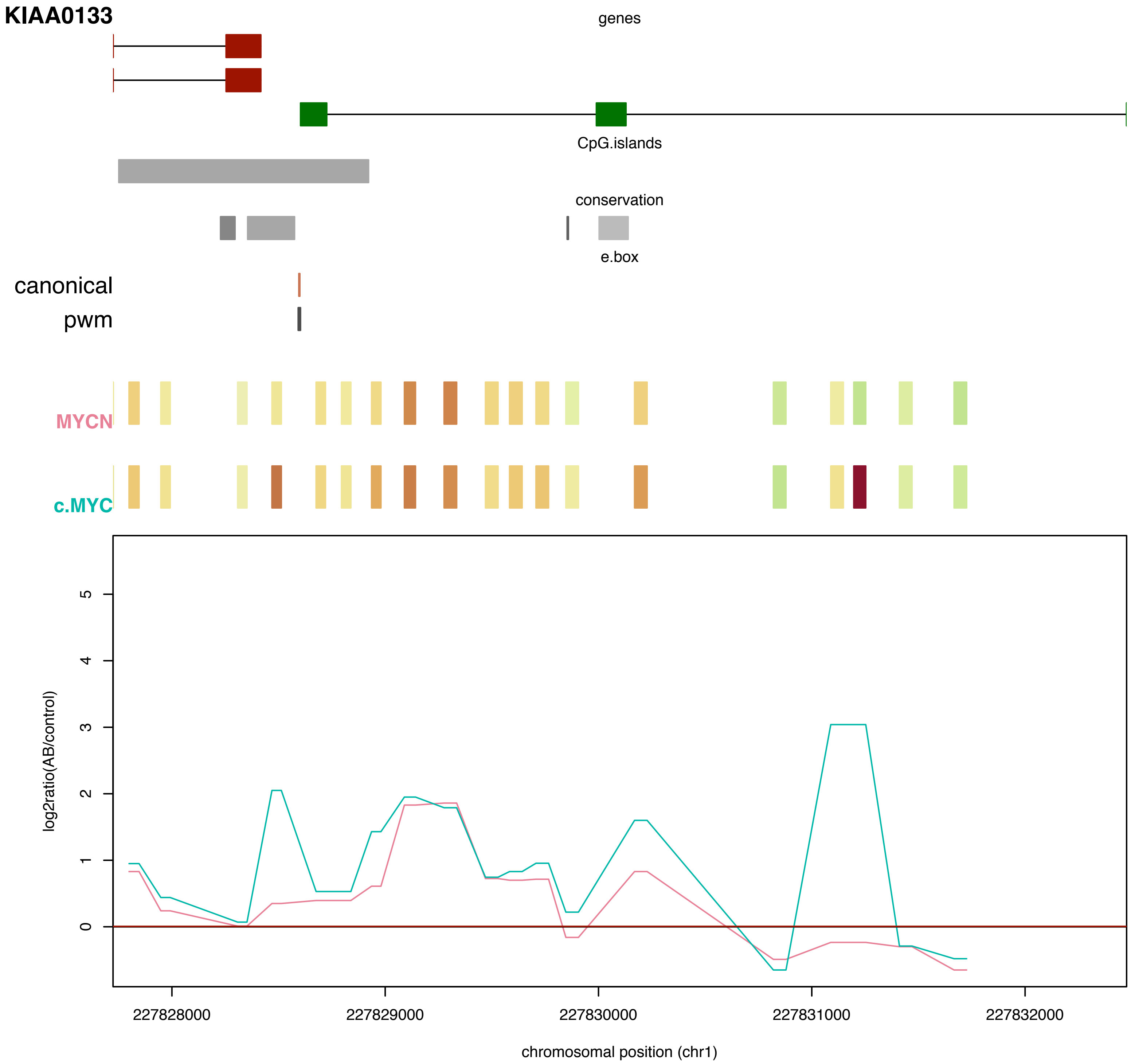
KIAA0133
genes
CpG.islands
conservation
e.box
canonical
pwm
MYCN
c.MYC
log2ratio(AB/control)
0
1
2
3
4
5
227828000
227829000
227830000
227831000
227832000
chromosomal position (chr1)

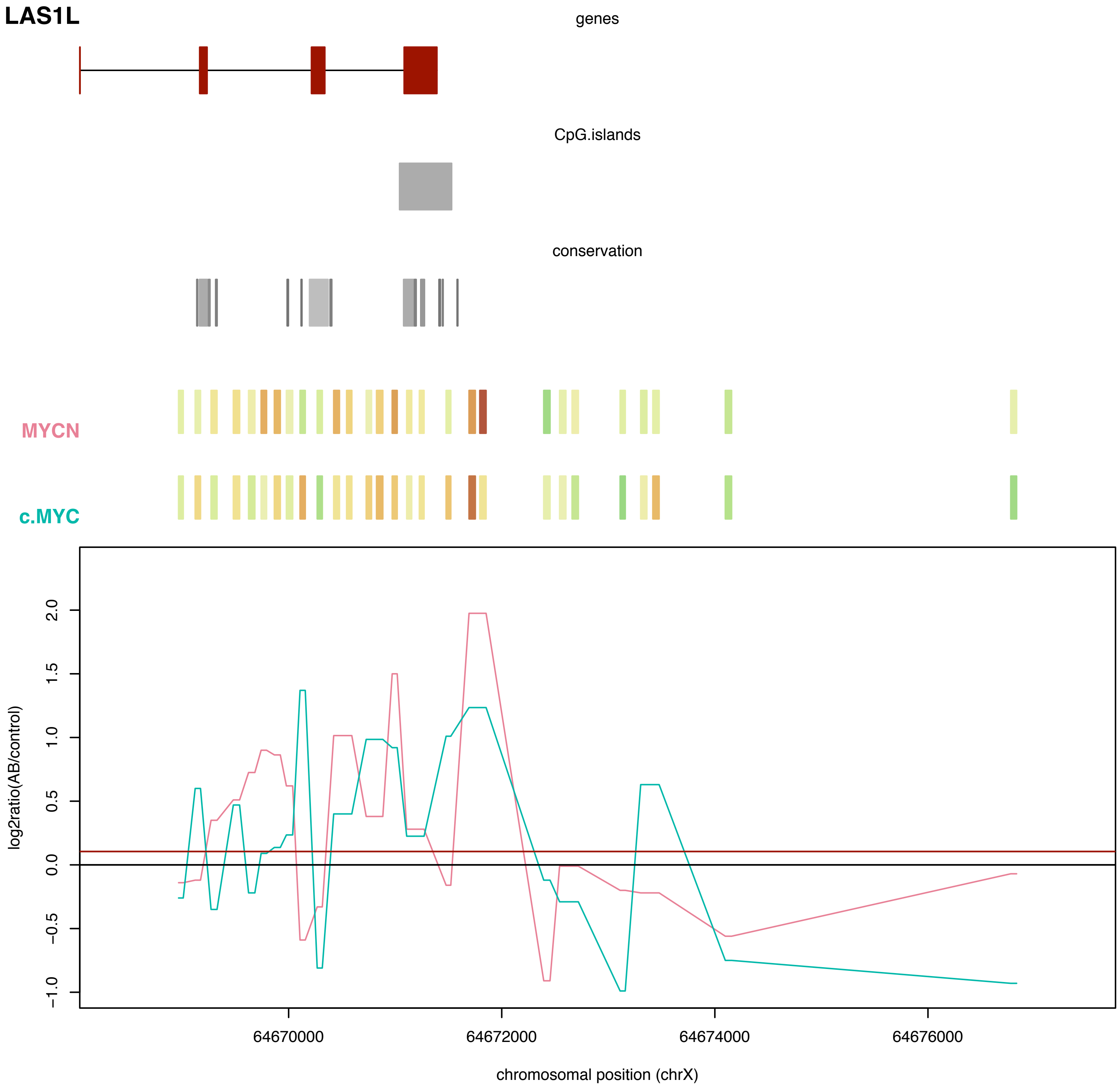

LAS1L
genes
CpG.islands
conservation
MYCN
c.MYC
log2ratio(AB/control)
2.0
1.5
1.0
0.5
0.0
−0.5
−1.0
64670000
64672000
64674000
64676000
chromosomal position (chrX)

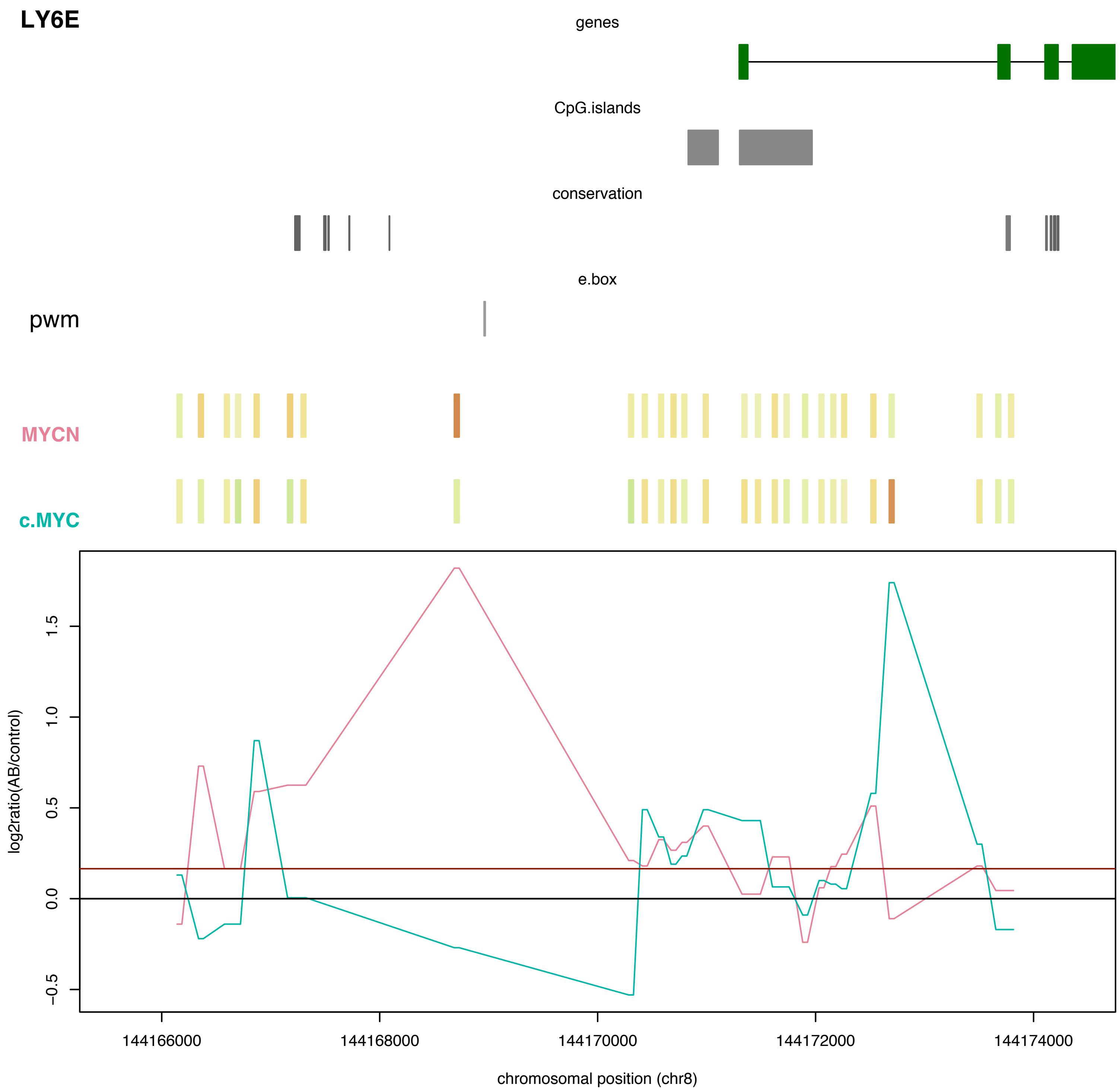

LY6E
genes
CpG.islands
conservation
e.box
pwm
MYCN
c.MYC
log2ratio(AB/control)
−0.5
0.0
0.5
1.0
1.5
144166000
144168000
144170000
144172000
144174000
chromosomal position (chr8)

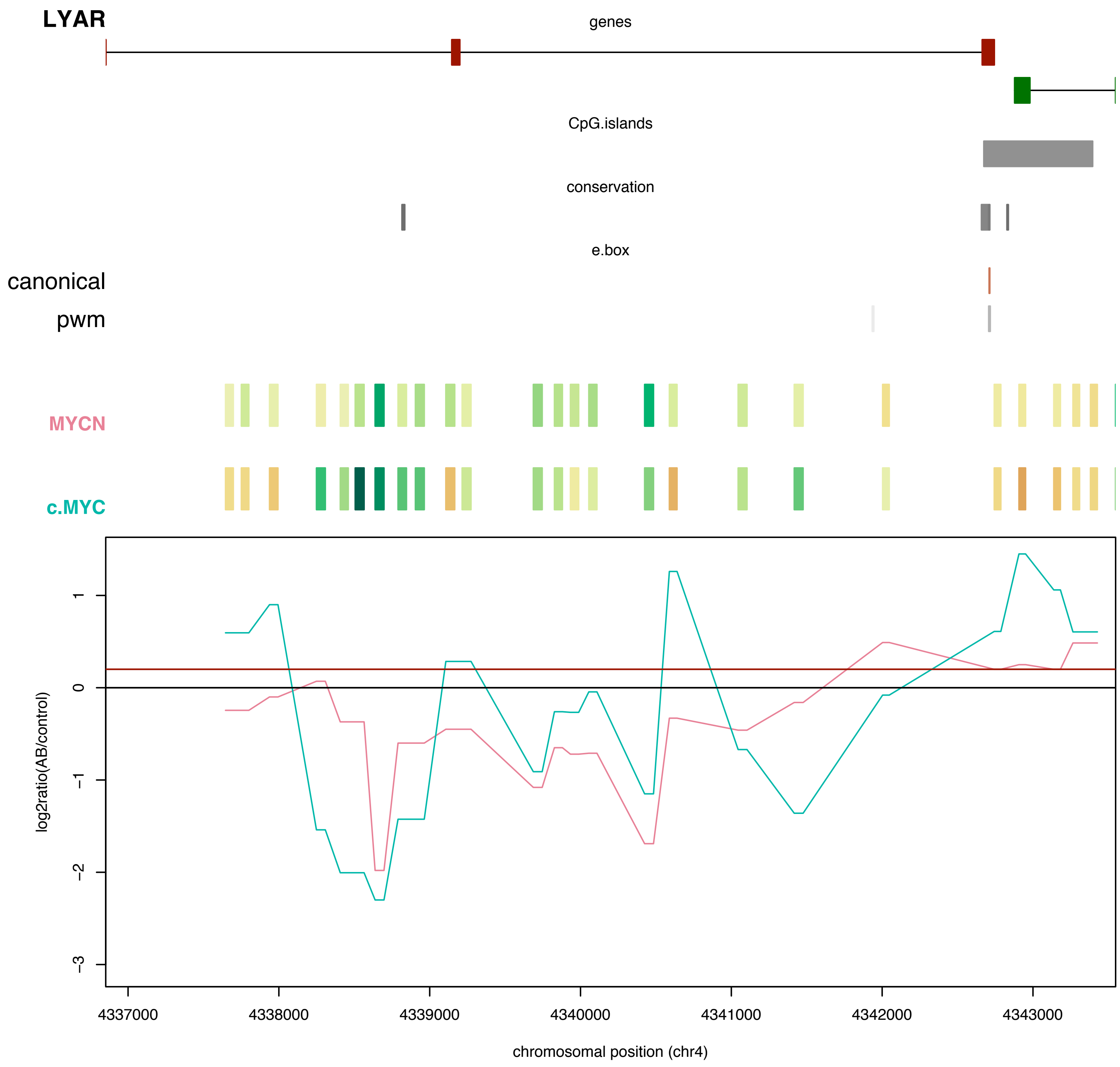

LYAR
genes
CpG.islands
conservation
e.box
canonical
pwm
MYCN
c.MYC
log2ratio(AB/control)
1
0
−1
−2
−3
4337000
4338000
4339000
4340000
4341000
4342000
4343000
chromosomal position (chr4)

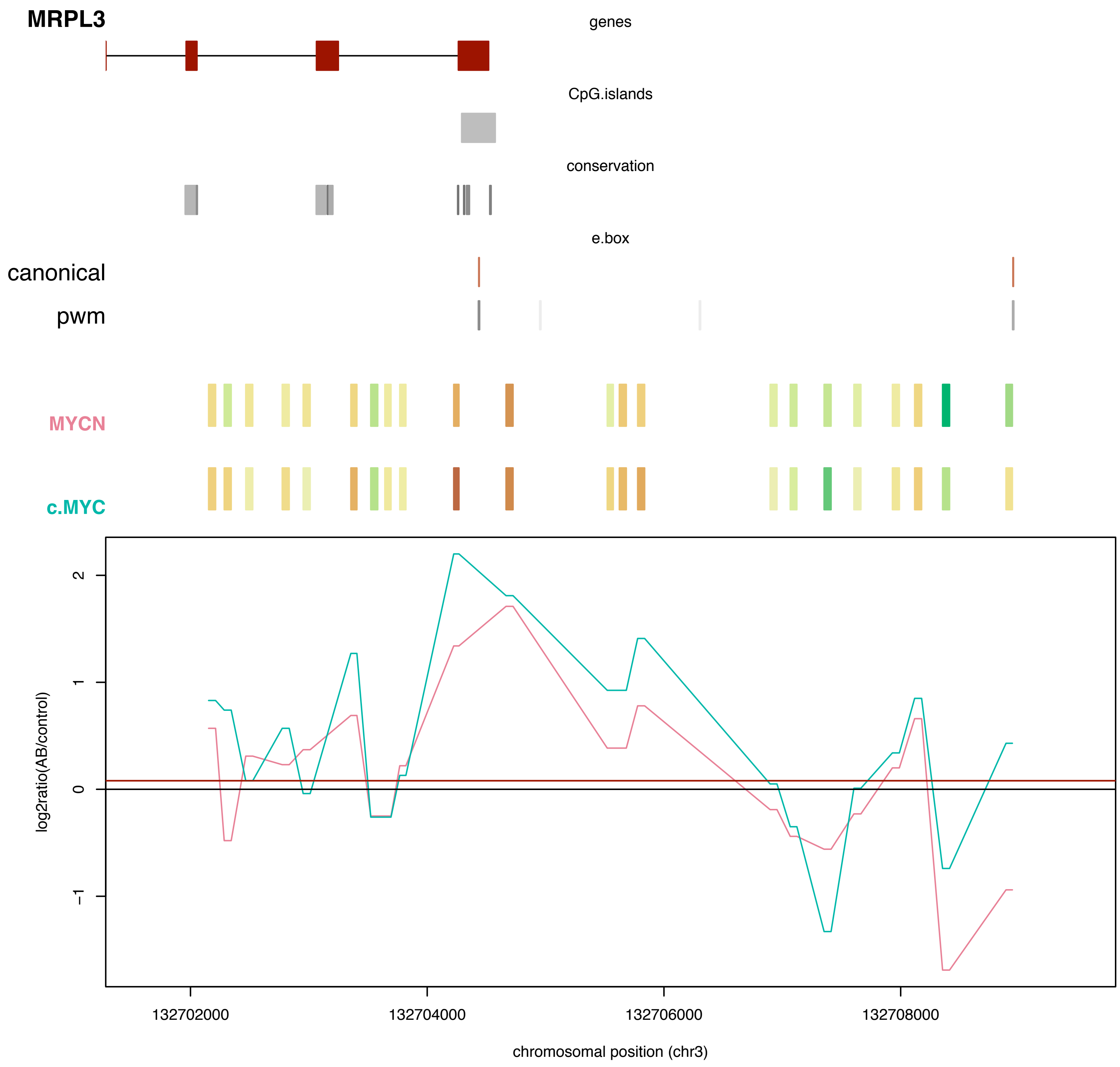

MRPL3
genes
CpG.islands
conservation
e.box
canonical
pwm
MYCN
c.MYC
log2ratio(AB/control)
2
1
0
−1
132702000
132704000
132706000
132708000
chromosomal position (chr3)

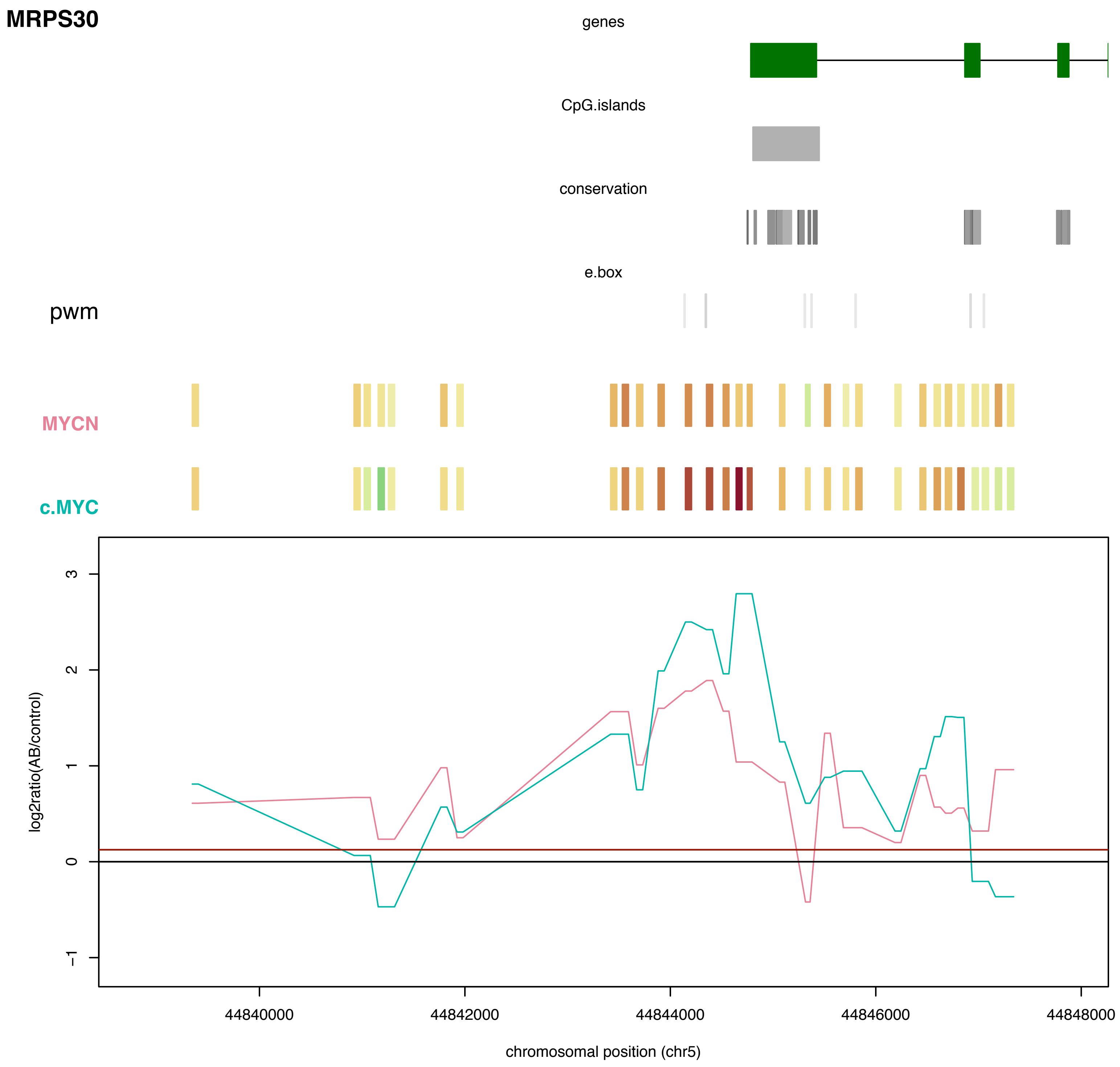

MRPS30
genes
CpG.islands
conservation
e.box
pwm
MYCN
c.MYC
log2ratio(AB/control)
3
2
1
0
−1
44840000
44842000
44844000
44846000
44848000
chromosomal position (chr5)

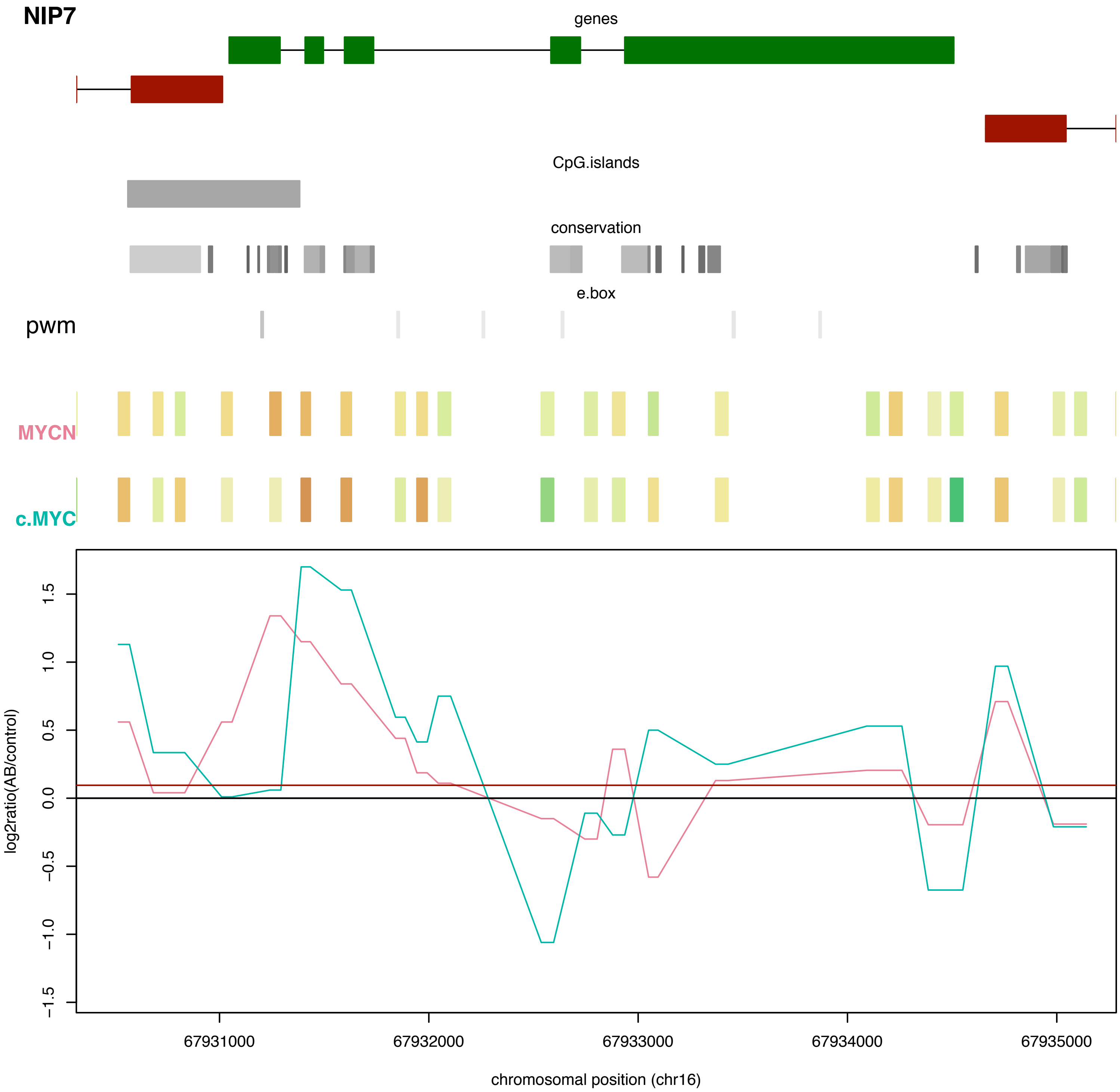
NIP7
genes
CpG.islands
conservation
e.box
pwm
MYCN
c.MYC
log2ratio(AB/control)
−1.5
−1.0
−0.5
0.0
0.5
1.0
1.5
67931000
67932000
67933000
67934000
67935000
chromosomal position (chr16)

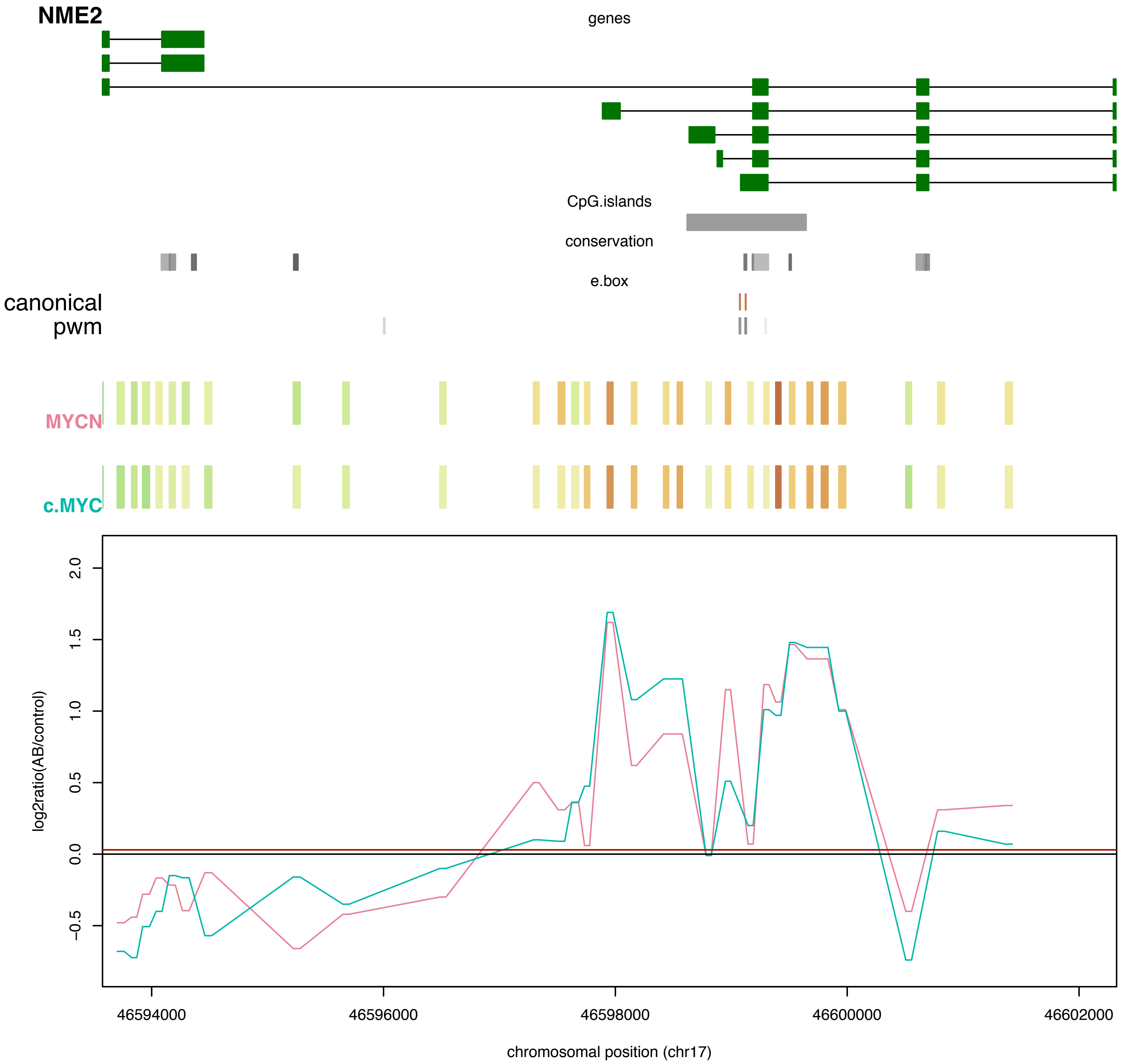

NME2
genes
CpG.islands
conservation
e.box
canonical
pwm
MYCN
c.MYC
log2ratio(AB/control)
−0.5
0.0
0.5
1.0
1.5
2.0
46594000
46596000
46598000
46600000
46602000
chromosomal position (chr17)

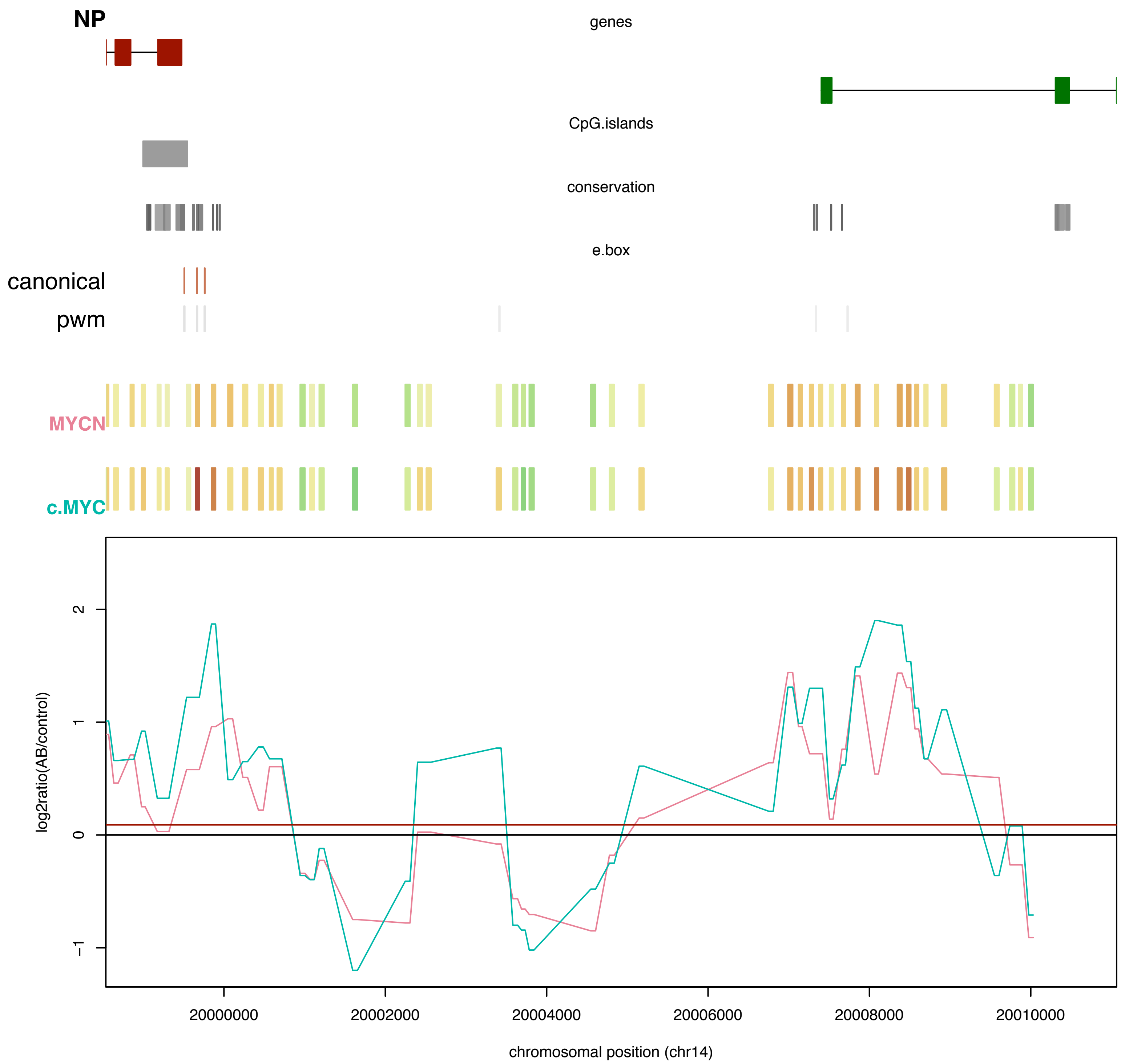
NP
genes
CpG.islands
conservation
e.box
canonical
pwm
MYCN
c.MYC
log2ratio(AB/control)
2
1
0
−1
20000000
20002000
20004000
20006000
20008000
20010000
chromosomal position (chr14)

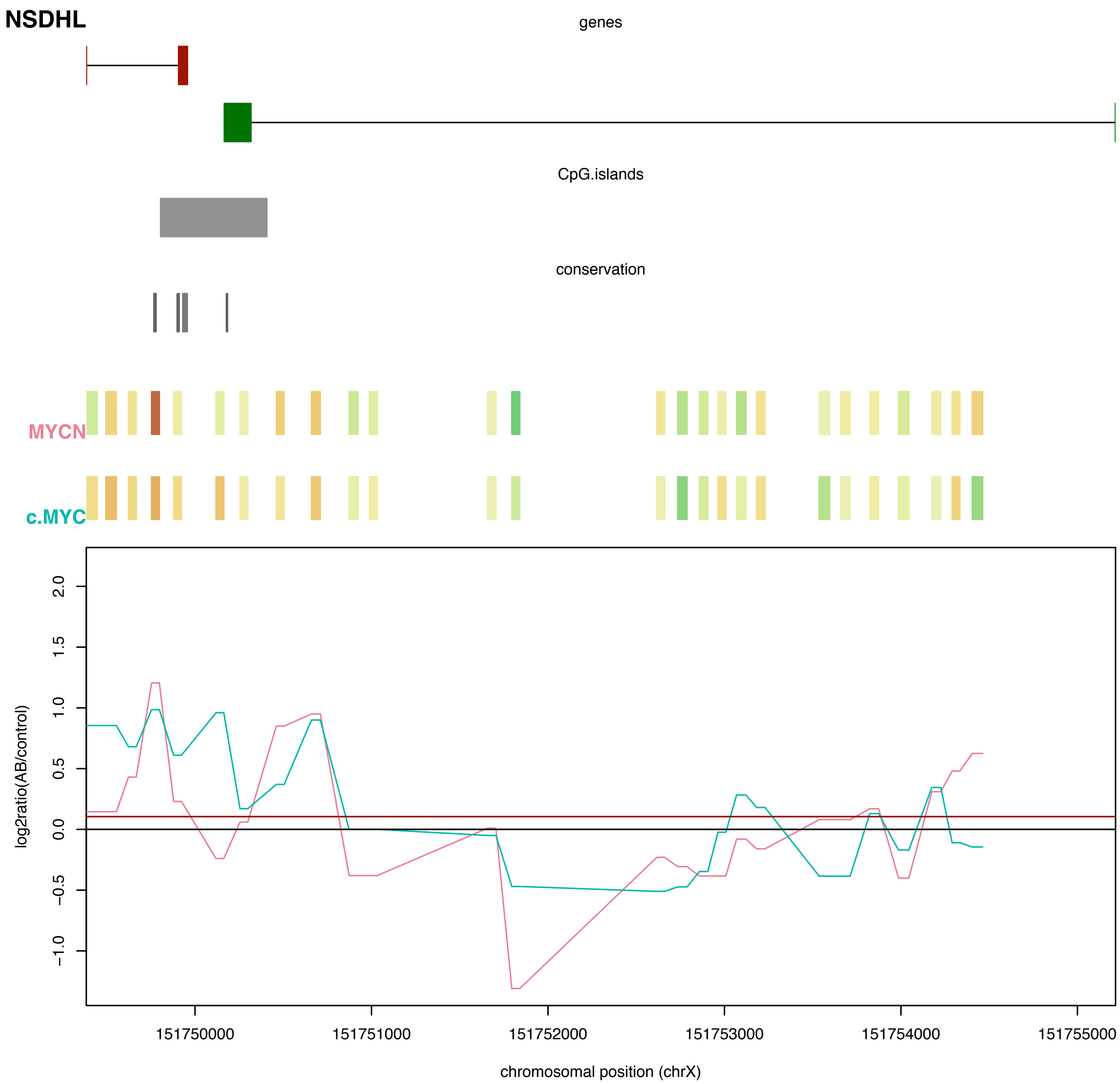
NSDHL
genes
CpG.islands
conservation
MYCN
c.MYC
log2ratio(AB/control)
2.0
1.5
1.0
0.5
0.0
−0.5
−1.0
151750000
151751000
151752000
151753000
151754000
151755000
chromosomal position (chrX)

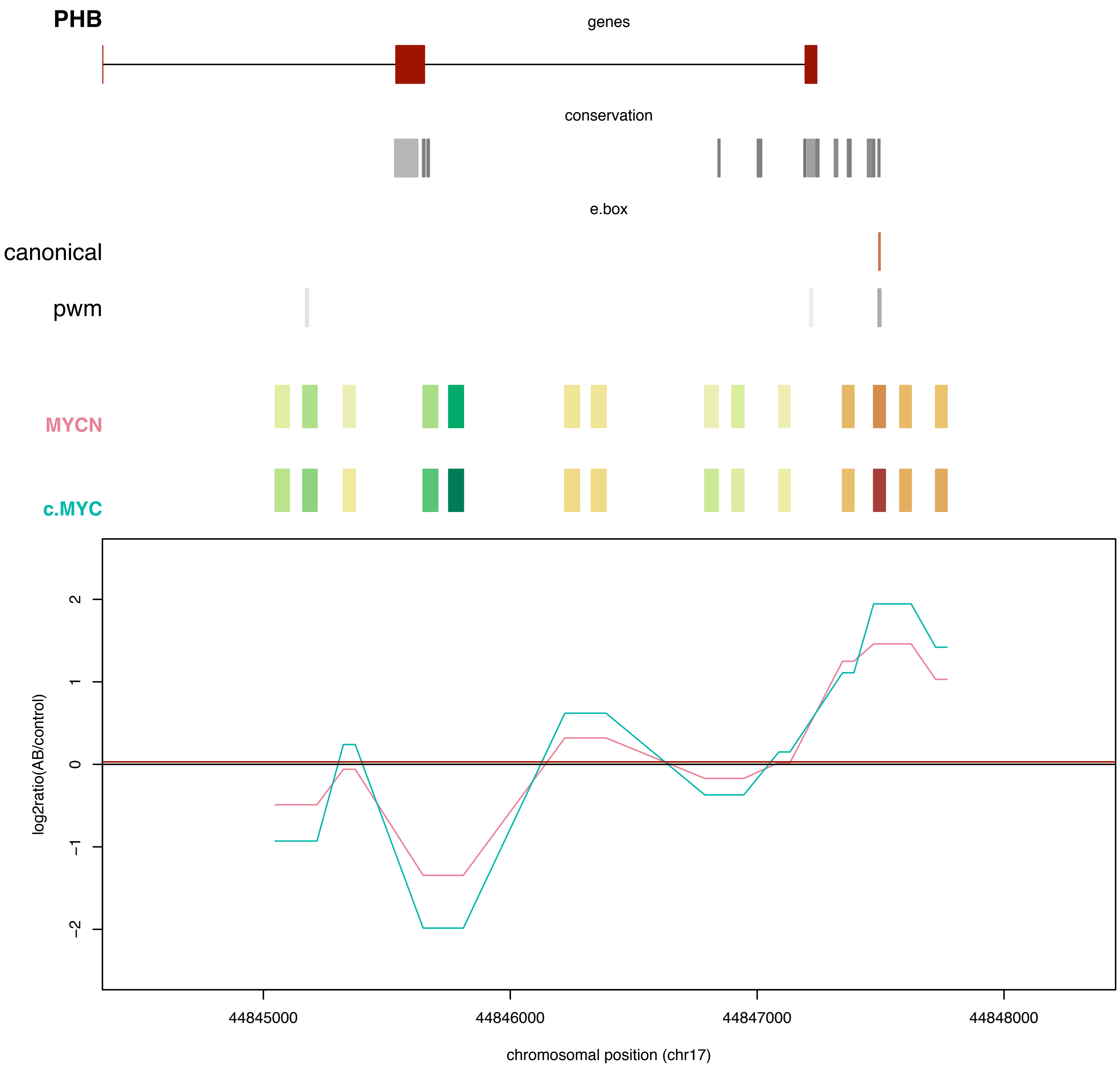
PHB
genes
conservation
e.box
canonical
pwm
MYCN
c.MYC
log2ratio(AB/control)
2
1
0
−1
−2
44845000
44846000
44847000
44848000
chromosomal position (chr17)

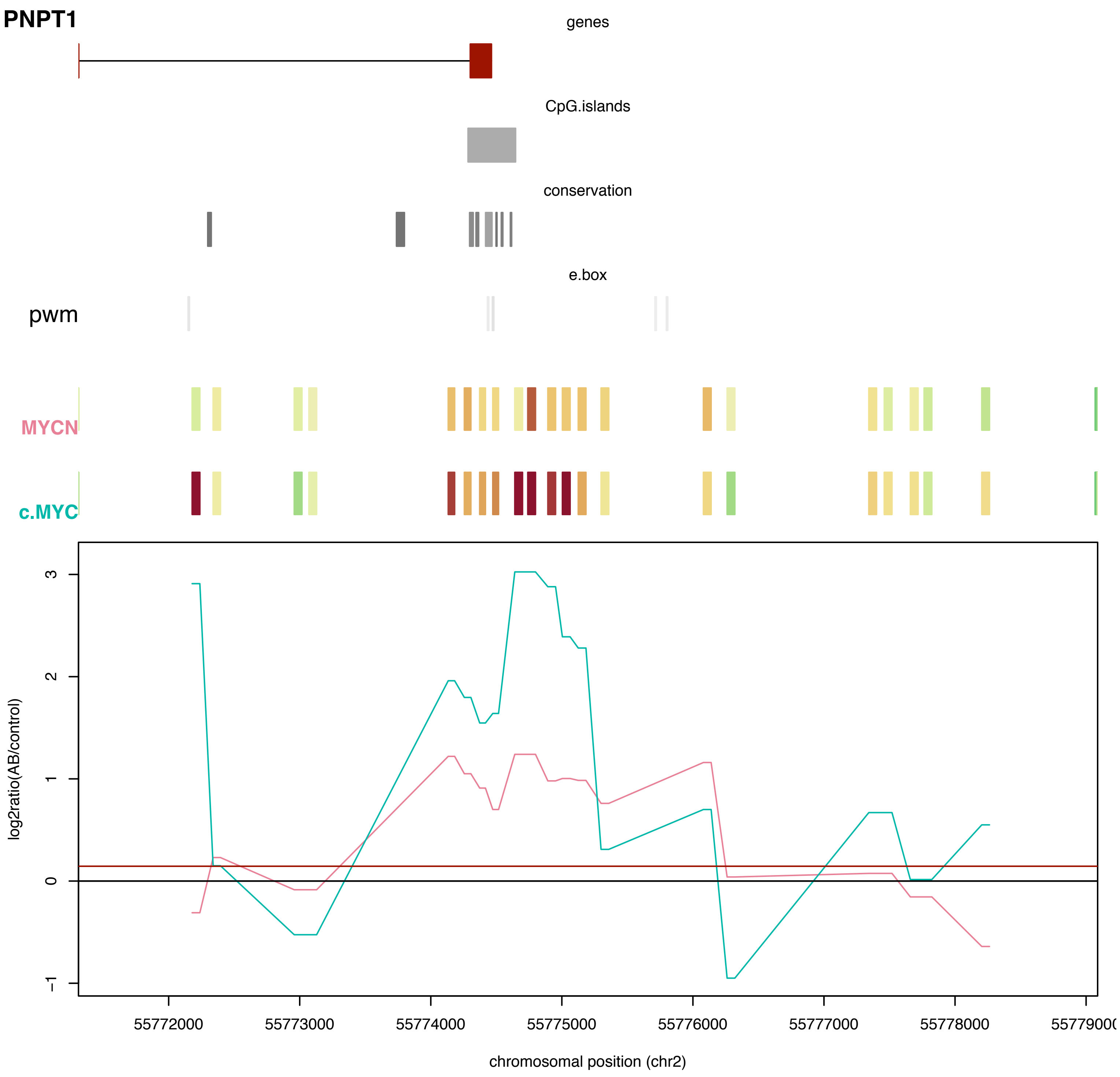
PNPT1
genes
CpG.islands
conservation
e.box
pwm
MYCN
c.MYC
log2ratio(AB/control)
3
2
1
0
−1
55772000
55773000
55774000
55775000
55776000
55777000
55778000
chromosomal position (chr2)

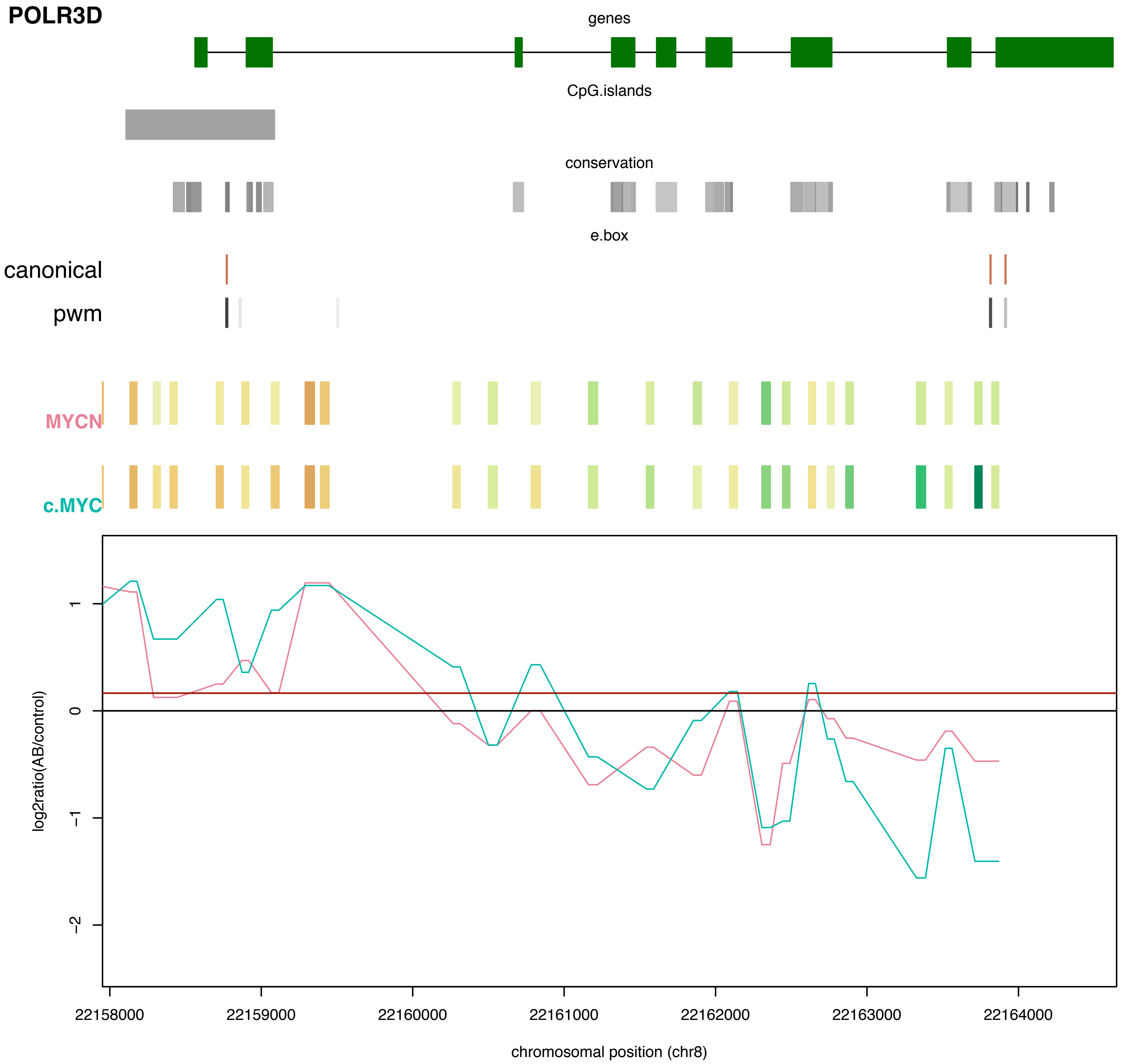

POLR3D
genes
CpG.islands
conservation
e.box
canonical
pwm
MYCN
c.MYC
log2ratio(AB/control)
1
0
−1
−2
22158000
22159000
22160000
22161000
22162000
22163000
22164000
chromosomal position (chr8)

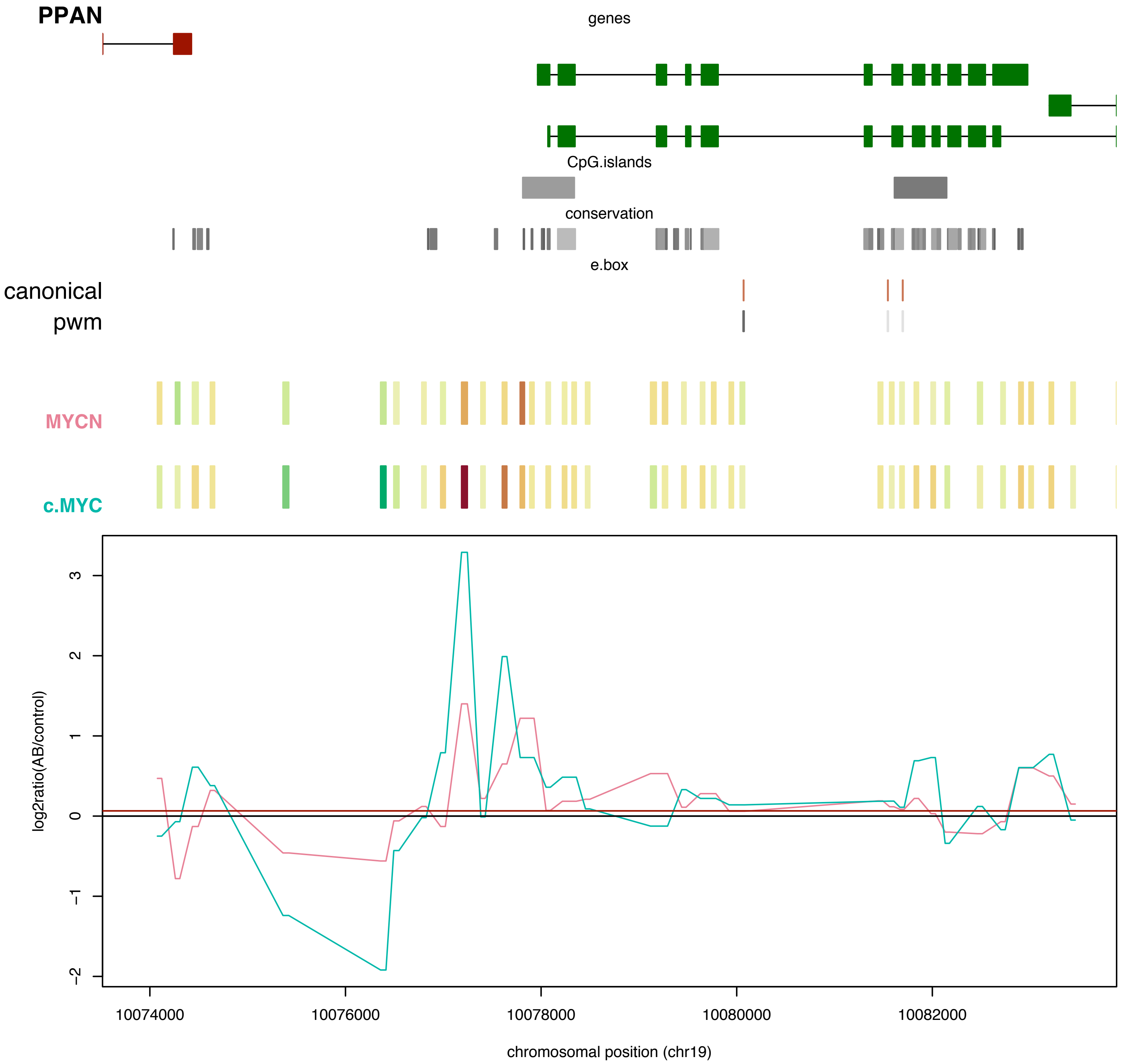
PPAN
genes
CpG.islands
conservation
e.box
canonical
pwm
MYCN
c.MYC
log2ratio(AB/control)
−2
−1
0
1
2
3
10074000
10076000
10078000
10080000
10082000
chromosomal position (chr19)

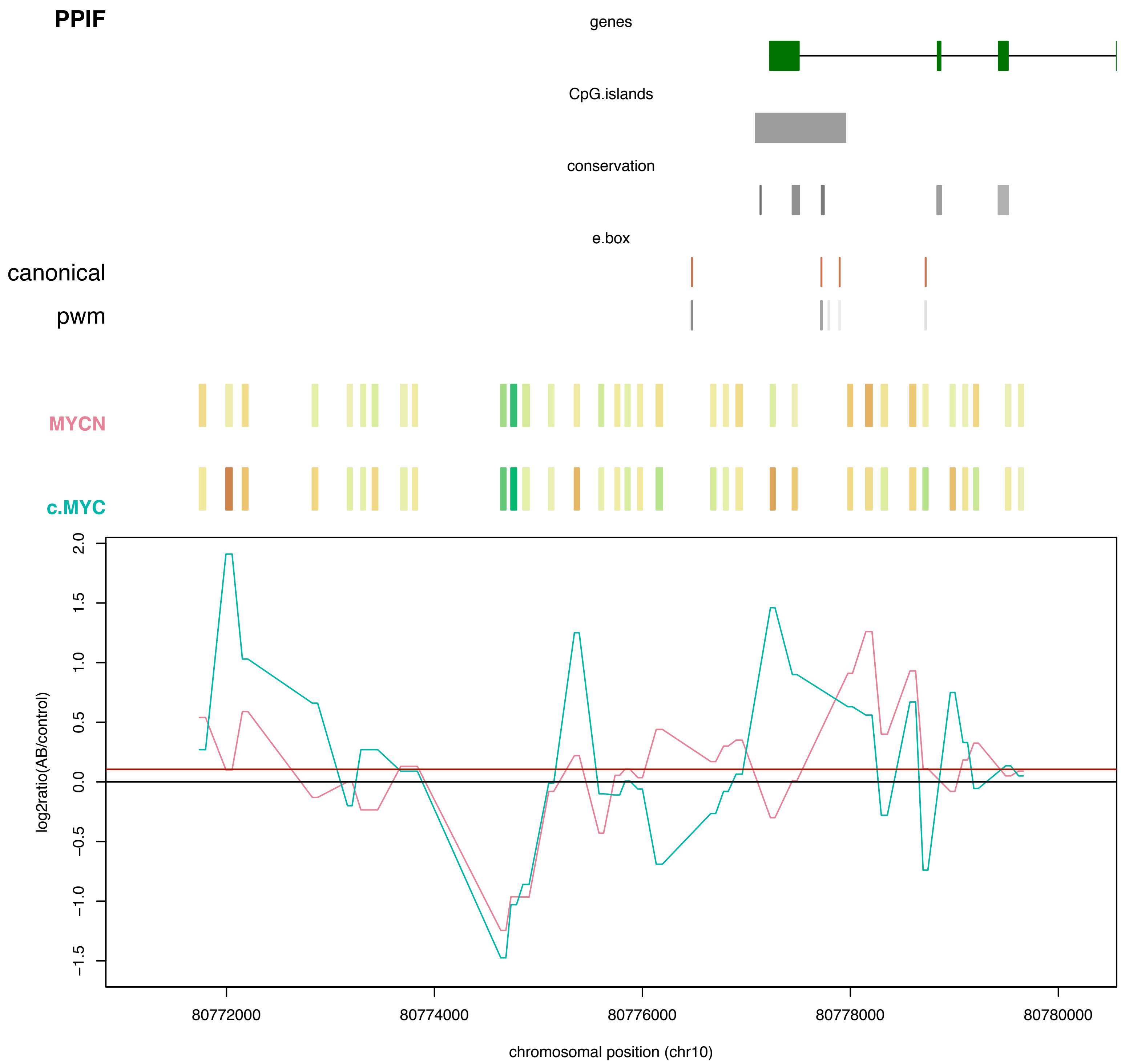

PPIF
genes
CpG.islands
conservation
e.box
canonical
pwm
MYCN
c.MYC
log2ratio(AB/control)
2.0
1.5
1.0
0.5
0.0
−0.5
−1.0
−1.5
80772000
80774000
80776000
80778000
80780000
chromosomal position (chr10)

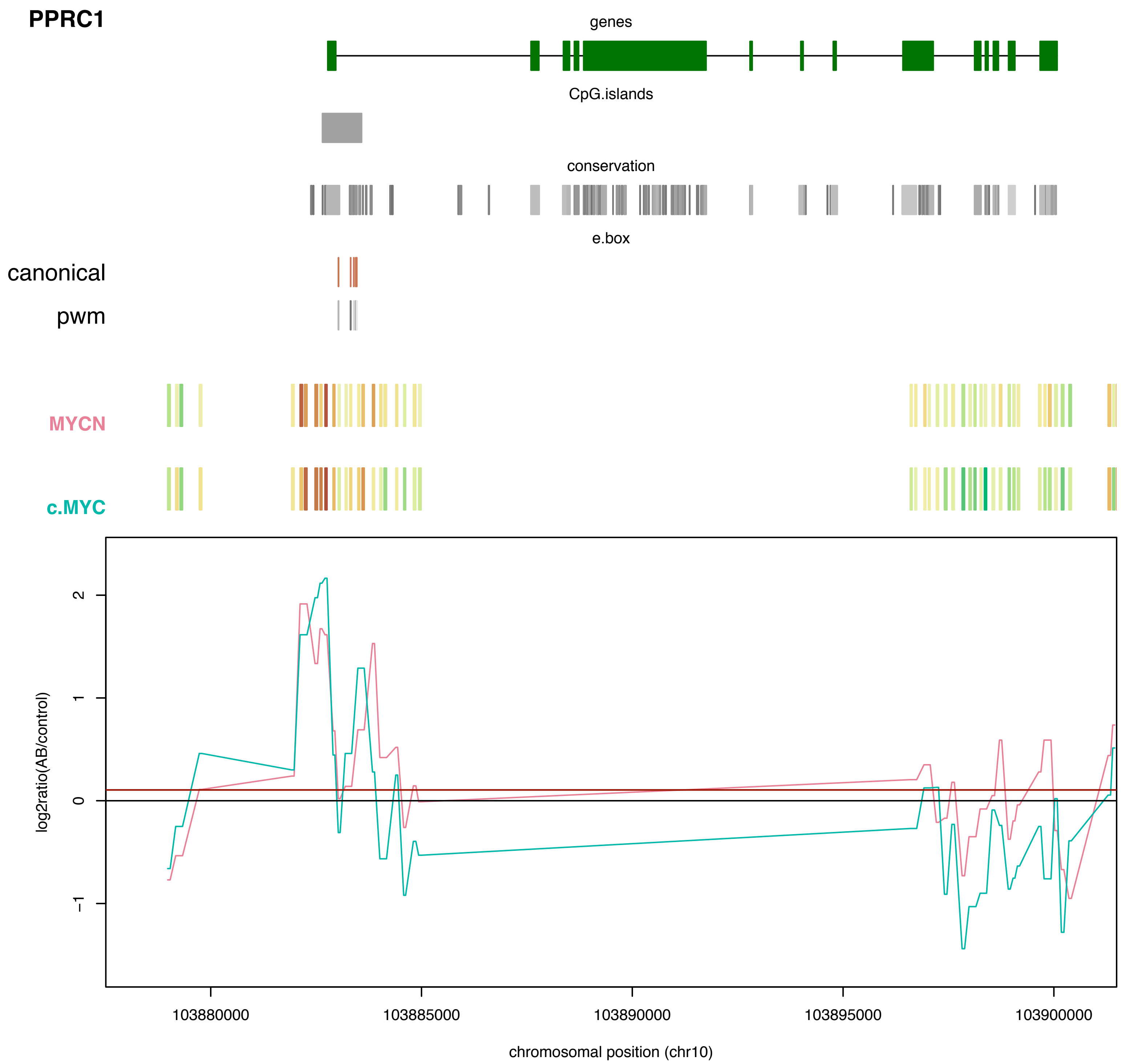

PPRC1
genes
CpG.islands
conservation
e.box
canonical
pwm
MYCN
c.MYC
log2ratio(AB/control)
2
1
0
−1
103880000
103885000
103890000
103895000
103900000
chromosomal position (chr10)

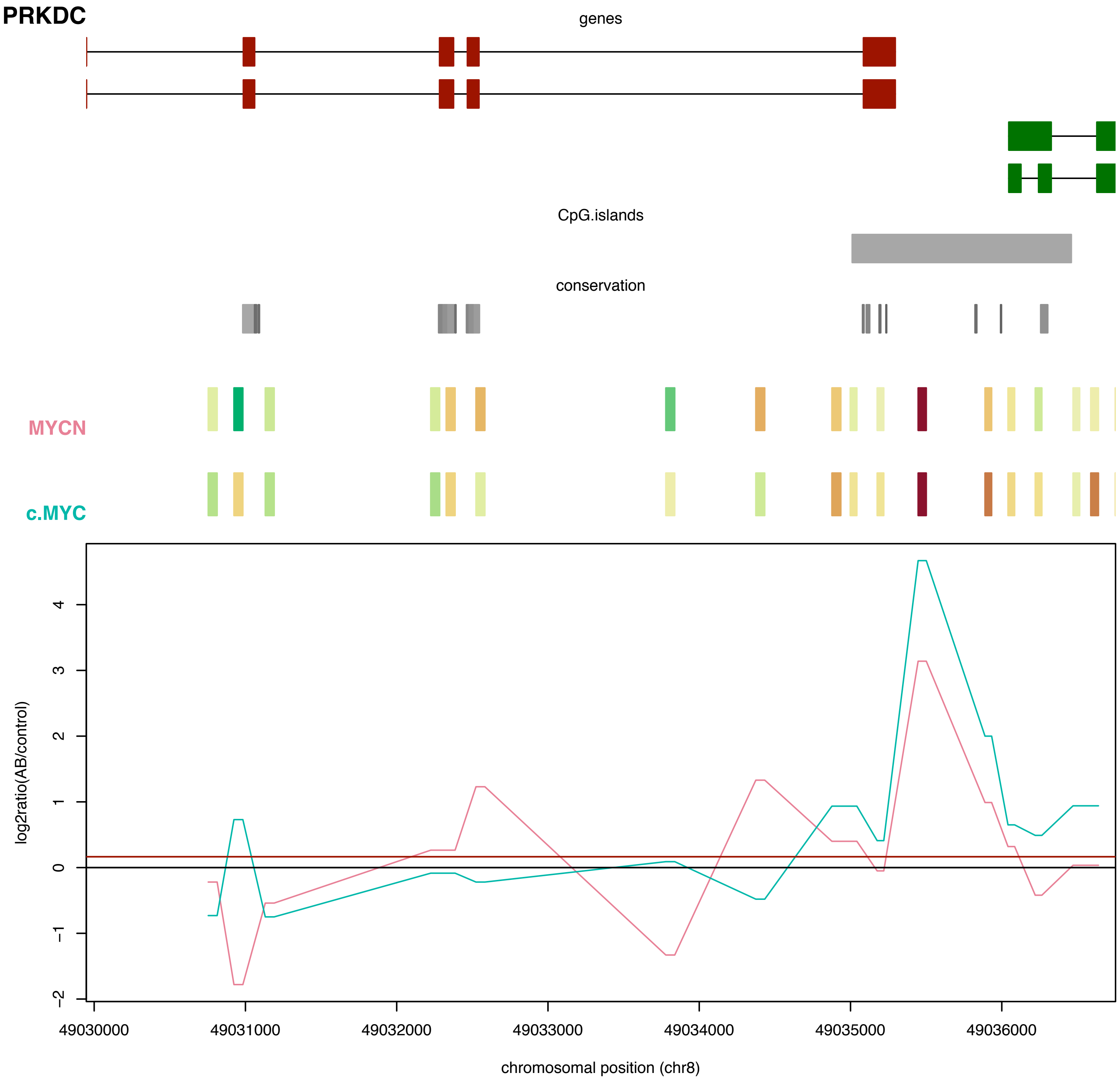

PRKDC
genes
CpG.islands
conservation
MYCN
c.MYC
log2ratio(AB/control)
4
3
2
1
0
−1
−2
49030000
49031000
49032000
49033000
49034000
49035000
49036000
chromosomal position (chr8)

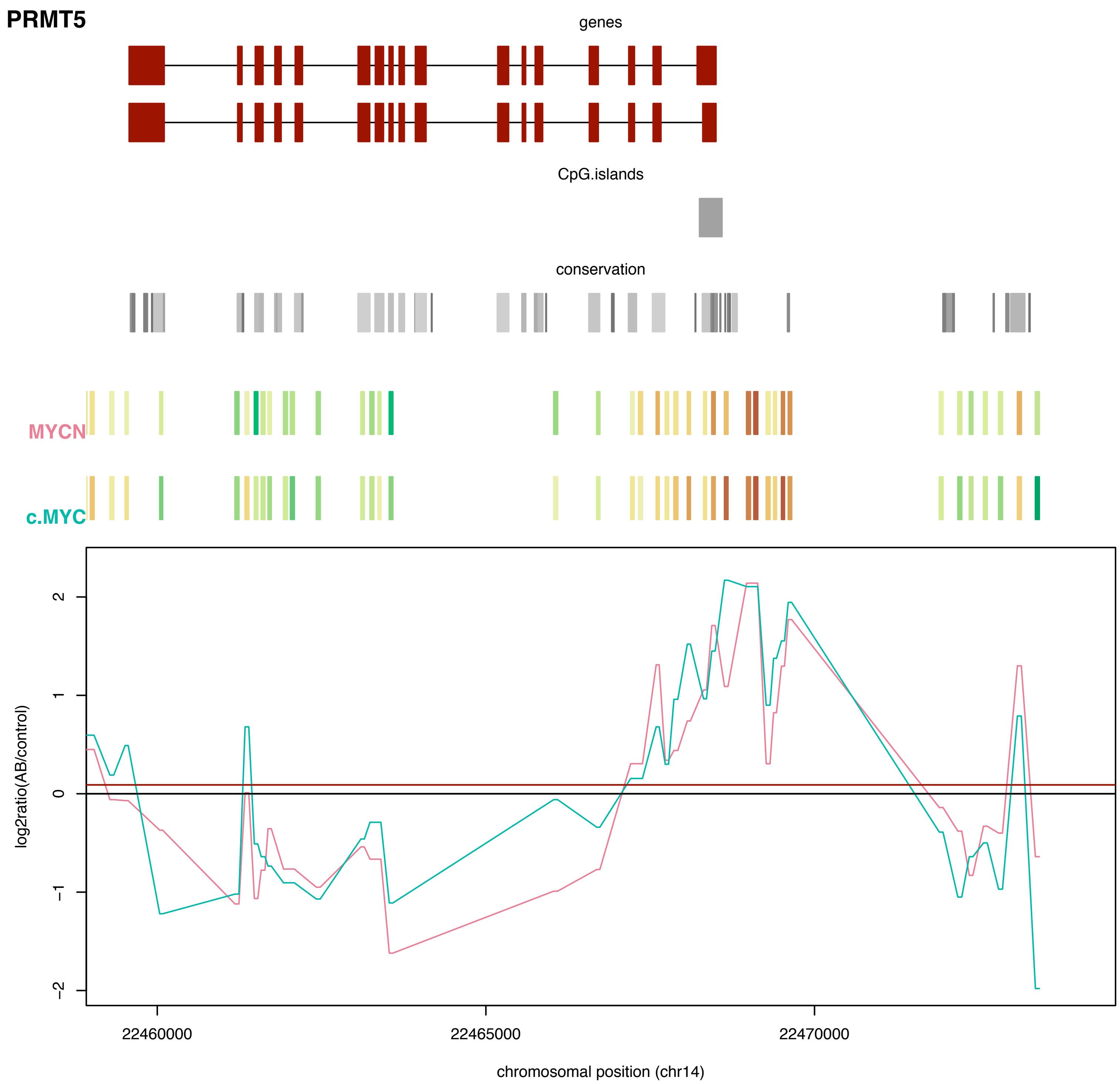

PRMT5
genes
CpG.islands
conservation
MYCN
c.MYC
log2ratio(AB/control)
2
1
0
−1
−2
22460000
22465000
22470000
chromosomal position (chr14)

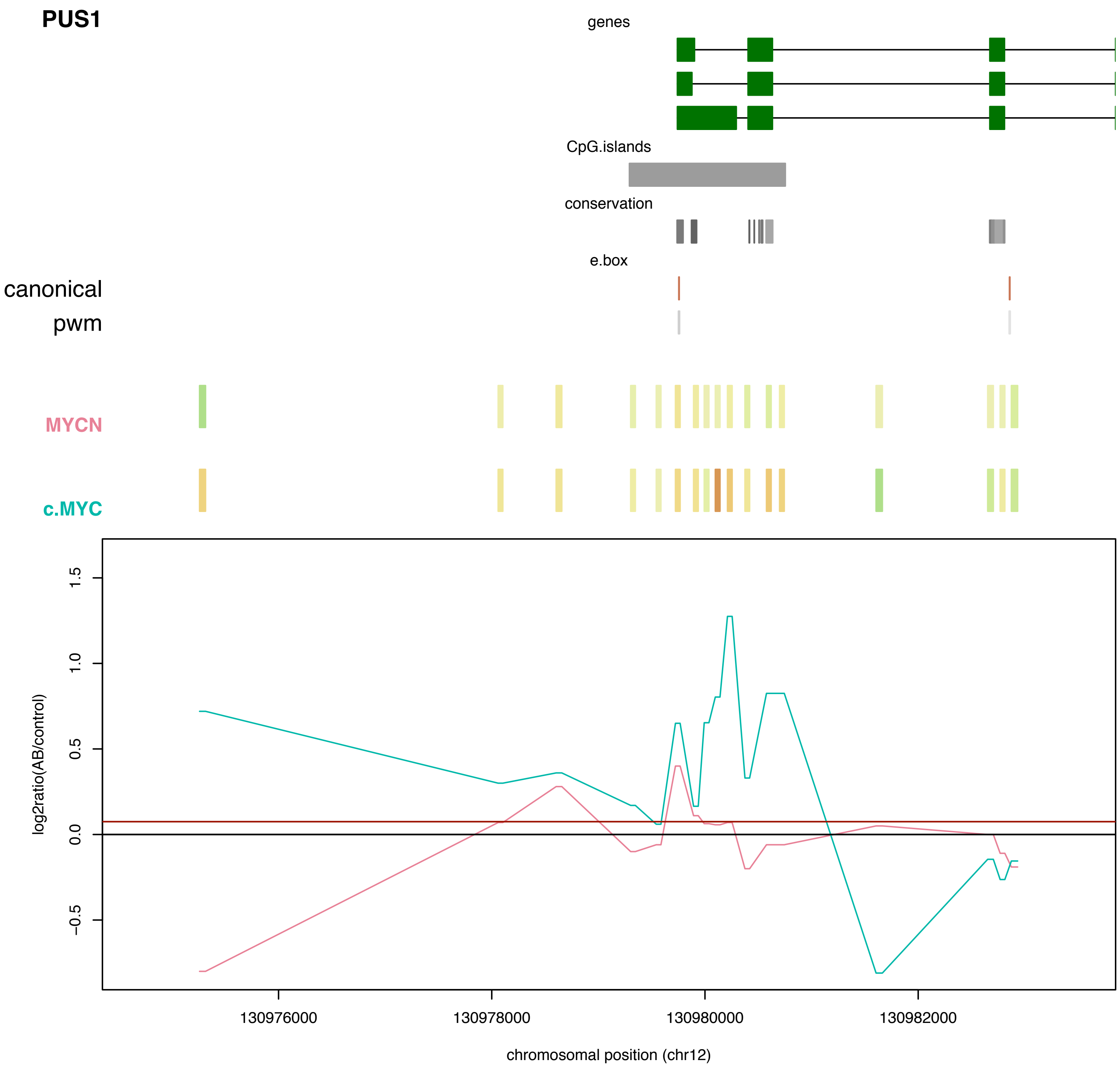
PUS1
genes
CpG.islands
conservation
e.box
canonical
pwm
MYCN
c.MYC
1.5
1.0
0.5
0.0
−0.5
log2ratio(AB/control)
130976000
130978000
130980000
130982000
chromosomal position (chr12)

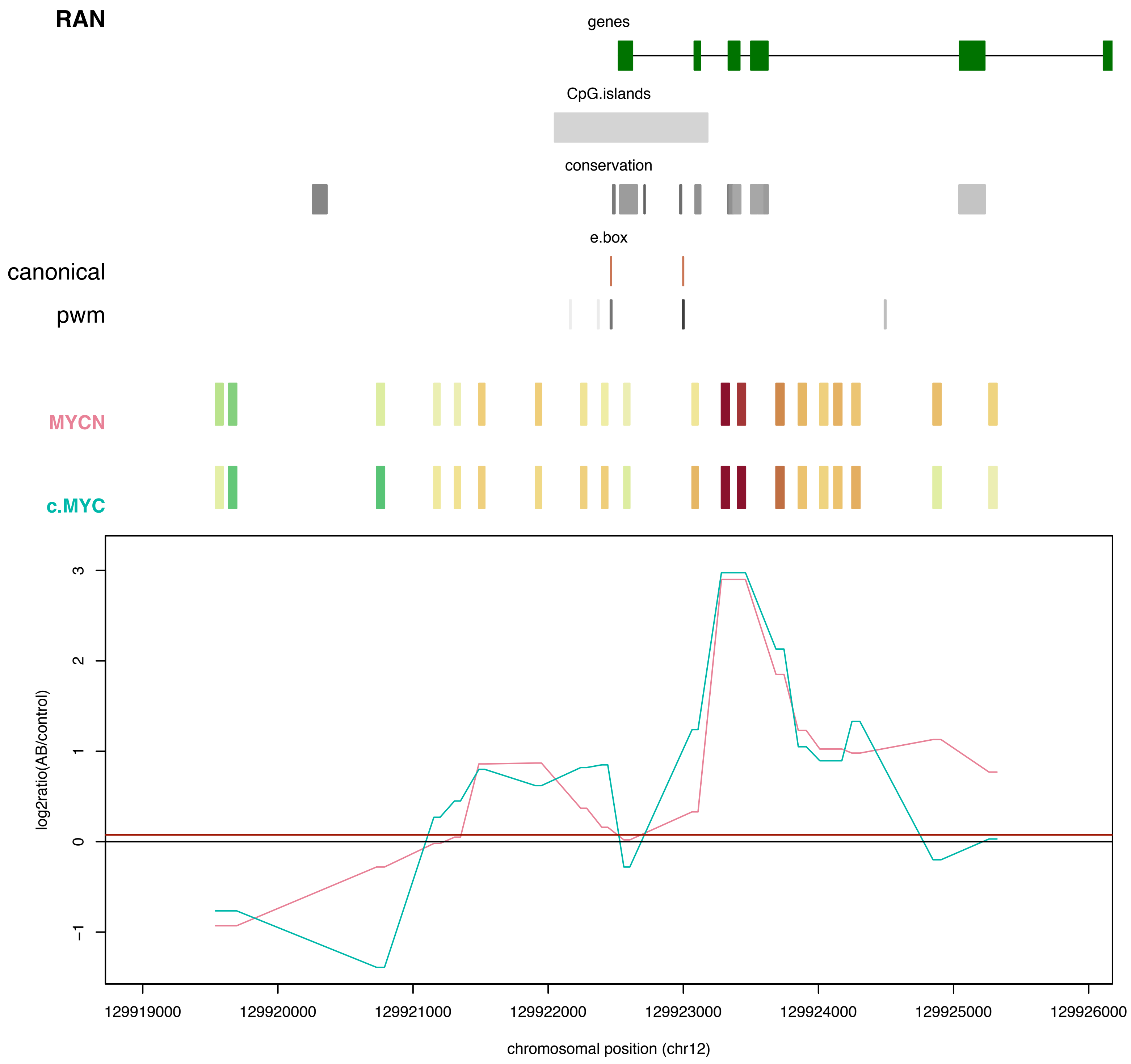

RAN
genes
CpG.islands
conservation
e.box
canonical
pwm
MYCN
c.MYC
log2ratio(AB/control)
3
2
1
0
−1
129919000
129920000
129921000
129922000
129923000
129924000
129925000
129926000
chromosomal position (chr12)

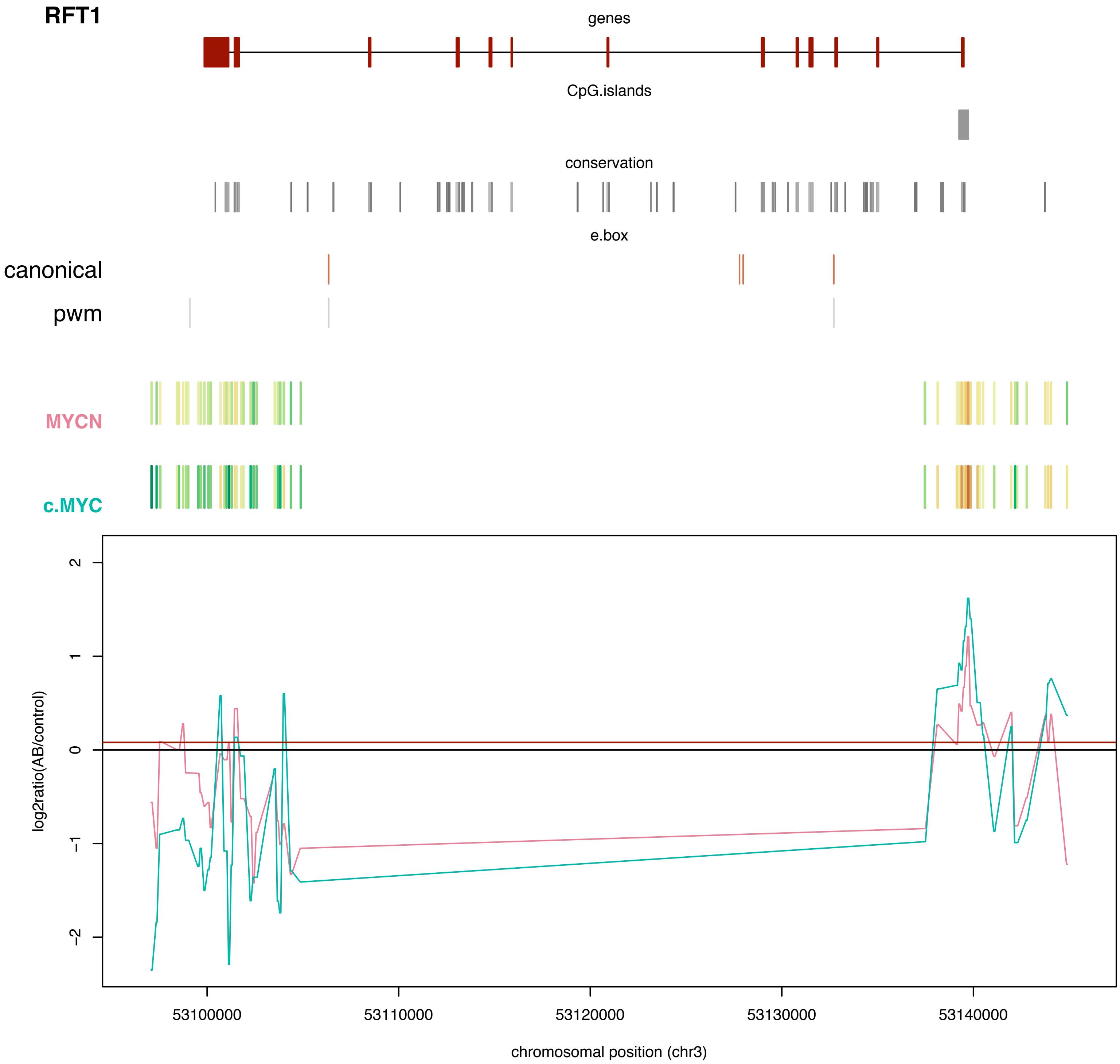

RFT1
genes
CpG.islands
conservation
e.box
canonical
pwm
MYCN
c.MYC
log2ratio(AB/control)
2
1
0
−1
−2
53100000
53110000
53120000
53130000
53140000
chromosomal position (chr3)

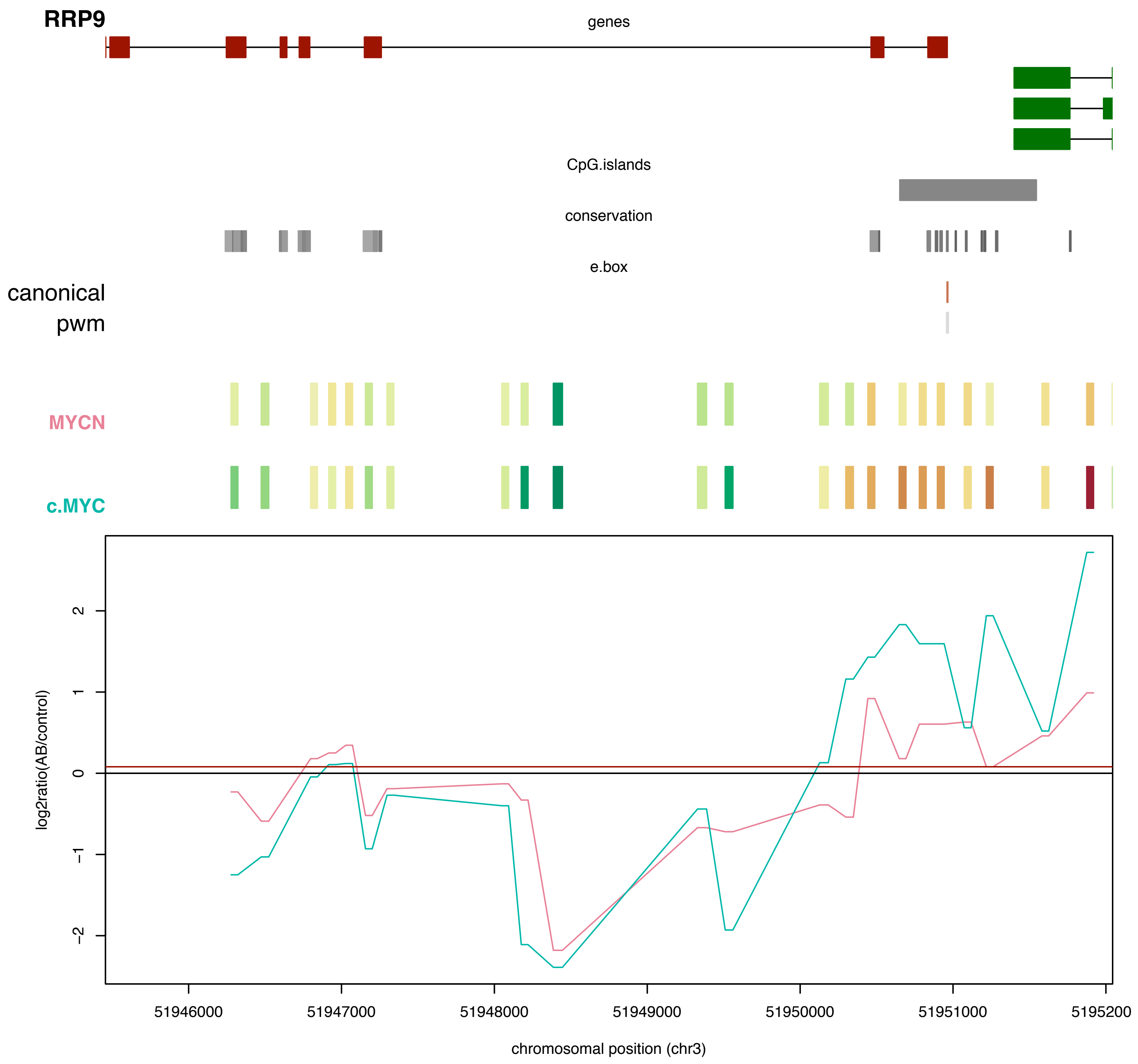

RRP9
genes
CpG.islands
conservation
e.box
canonical
pwm
MYCN
c.MYC
log2ratio(AB/control)
−2
−1
0
1
2
51946000
51947000
51948000
51949000
51950000
51951000
5195200
chromosomal position (chr3)

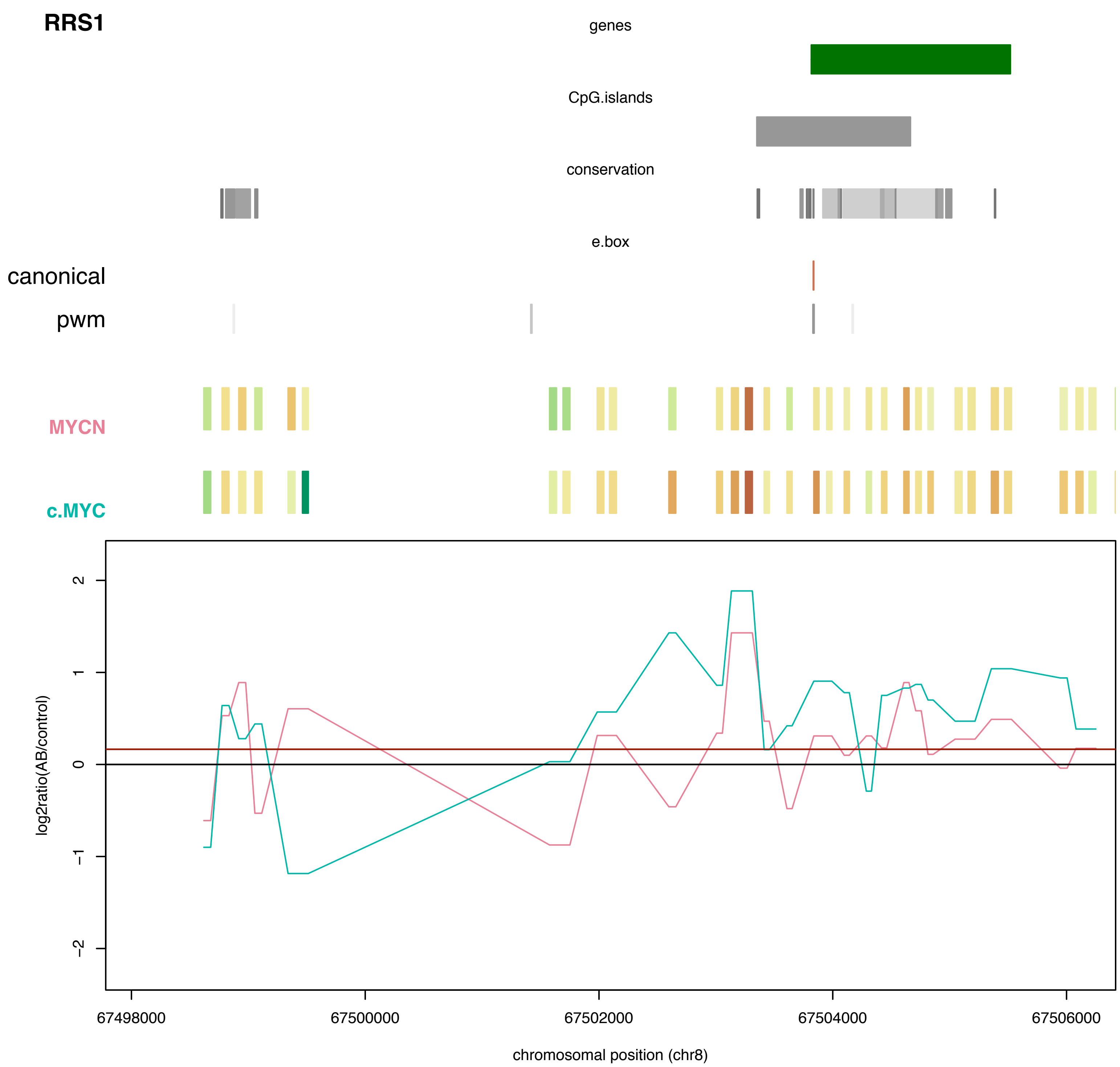

RRS1
genes
CpG.islands
conservation
e.box
canonical
pwm
MYCN
c.MYC
log2ratio(AB/control)
2
1
0
−1
−2
67498000
67500000
67502000
67504000
67506000
chromosomal position (chr8)

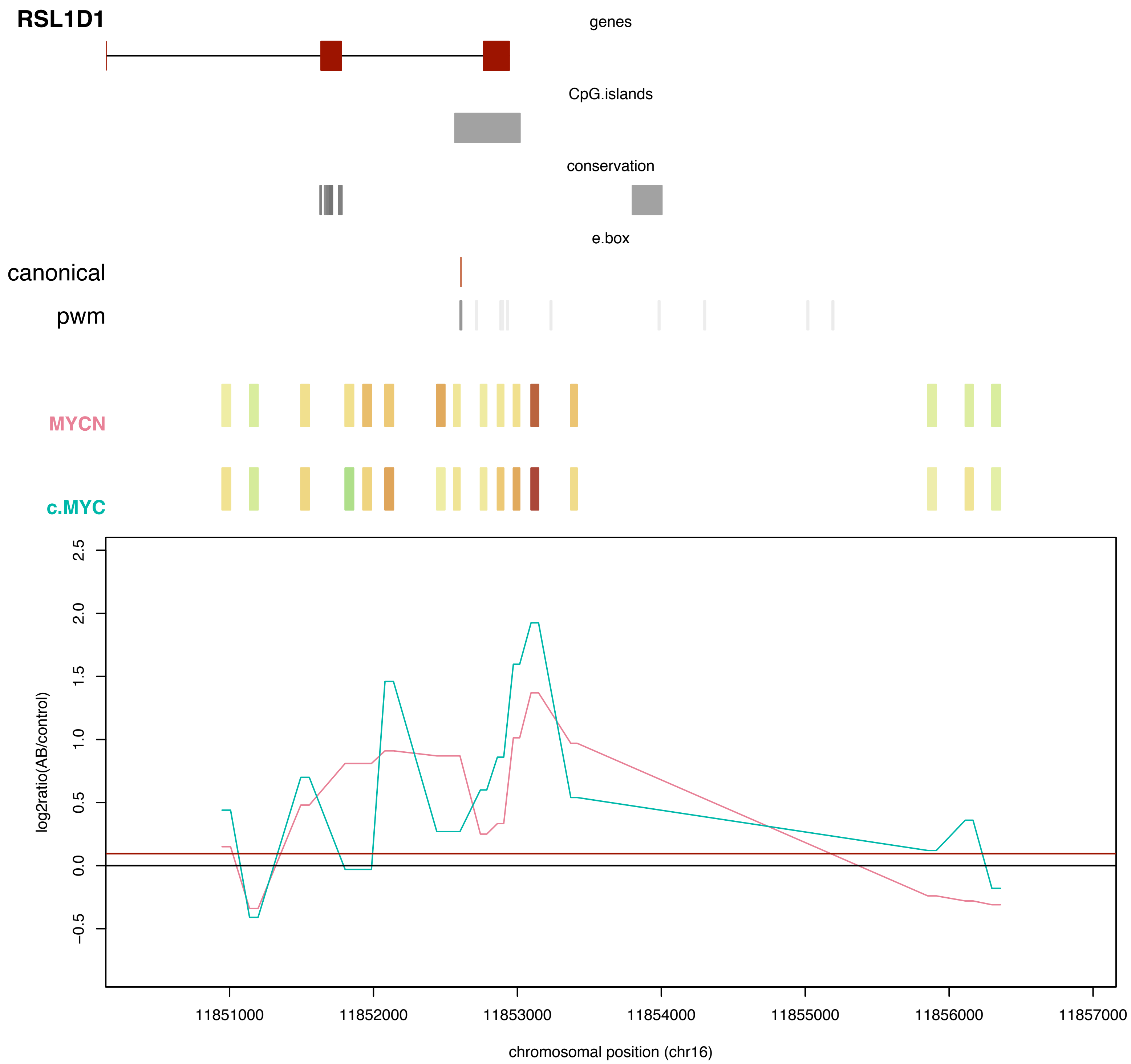

RSL1D1
genes
CpG.islands
conservation
e.box
canonical
pwm
MYCN
c.MYC
log2ratio(AB/control)
2.5
2.0
1.5
1.0
0.5
0.0
−0.5
11851000
11852000
11853000
11854000
11855000
11856000
11857000
chromosomal position (chr16)

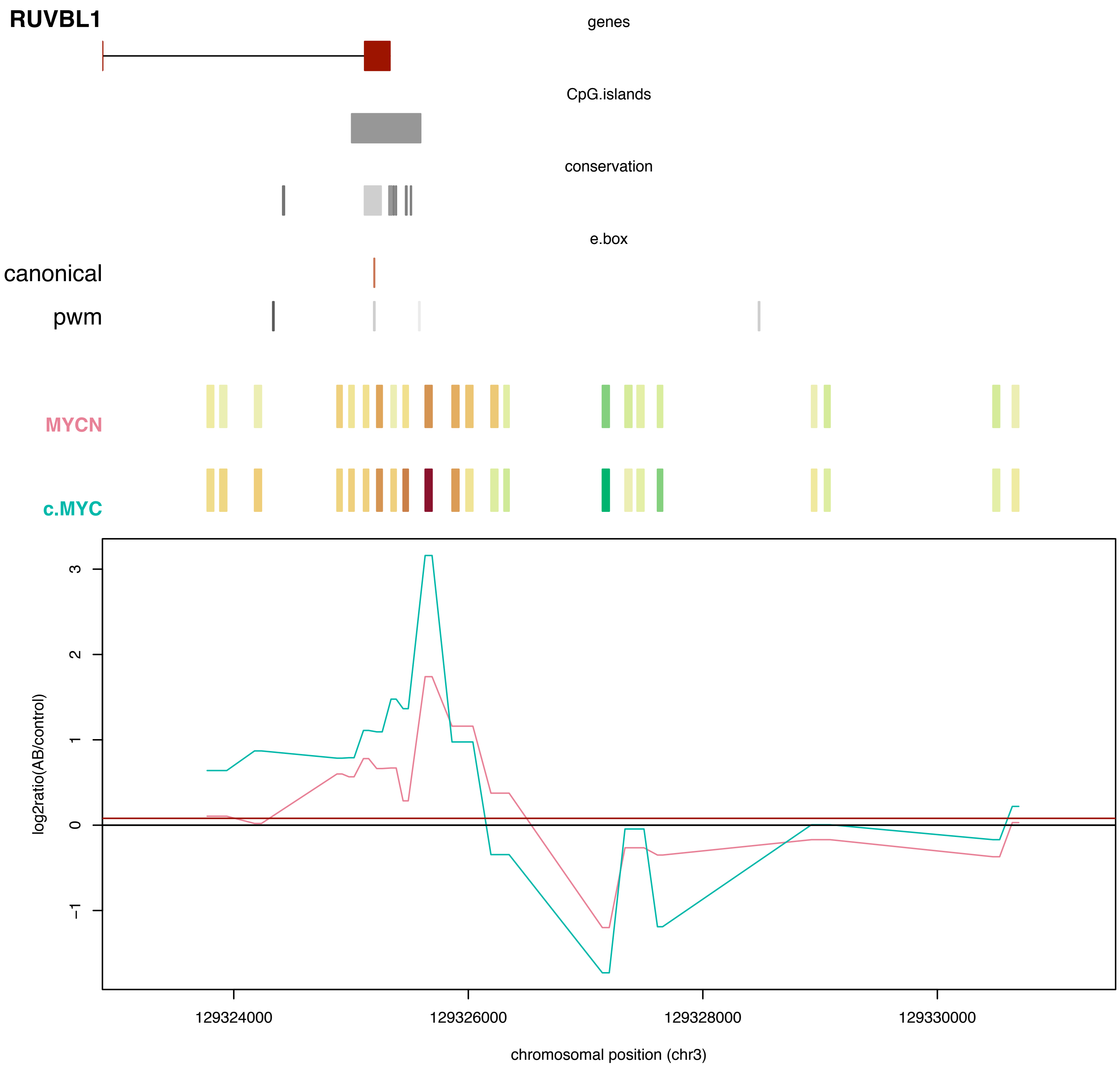

RUVBL1
genes
CpG.islands
conservation
e.box
canonical
pwm
MYCN
c.MYC
log2ratio(AB/control)
3
2
1
0
−1
129324000
129326000
129328000
129330000
chromosomal position (chr3)

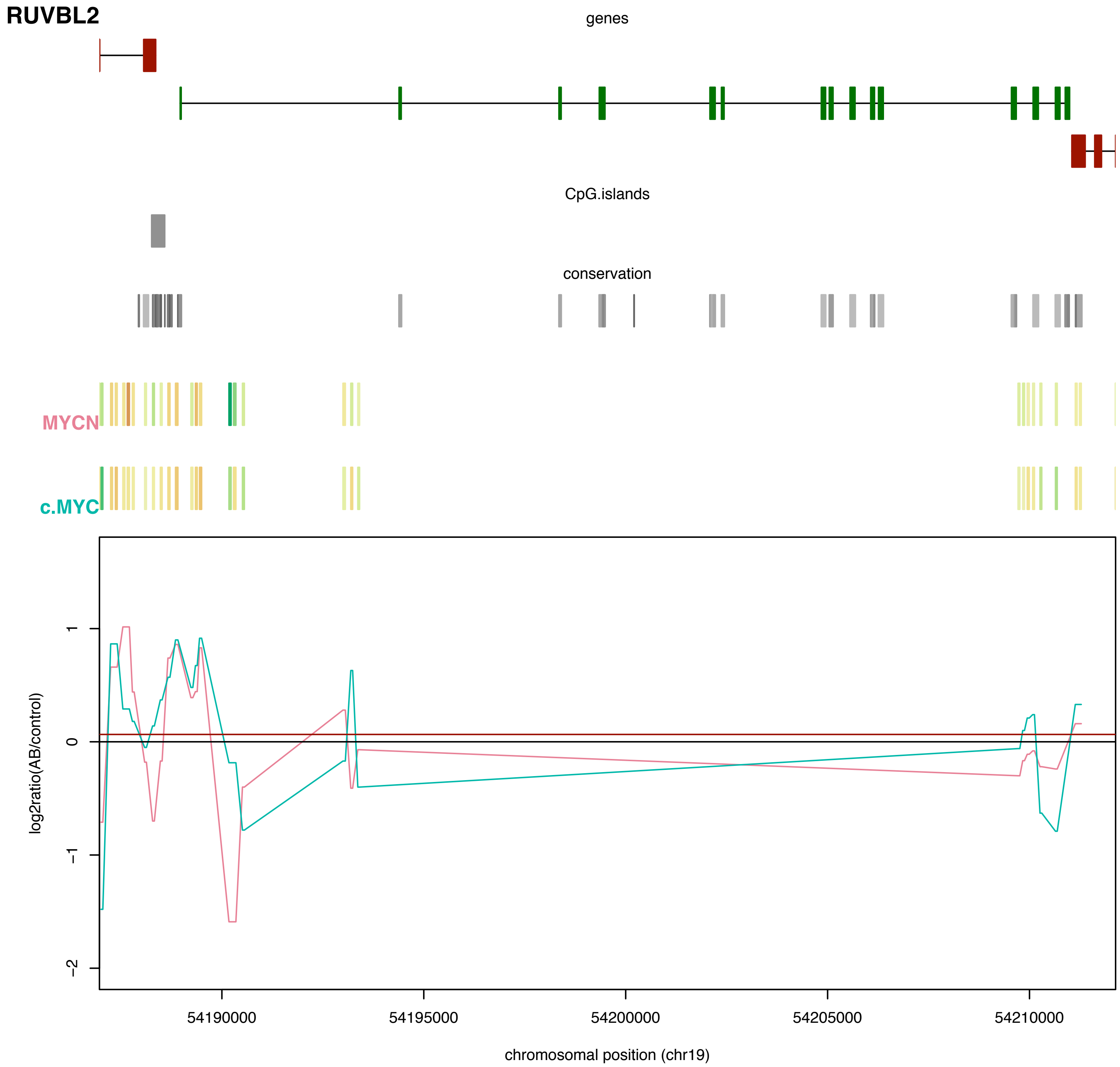

RUVBL2
genes
CpG.islands
conservation
MYCN
c.MYC
log2ratio(AB/control)
1
0
−1
−2
54190000
54195000
54200000
54205000
54210000
chromosomal position (chr19)

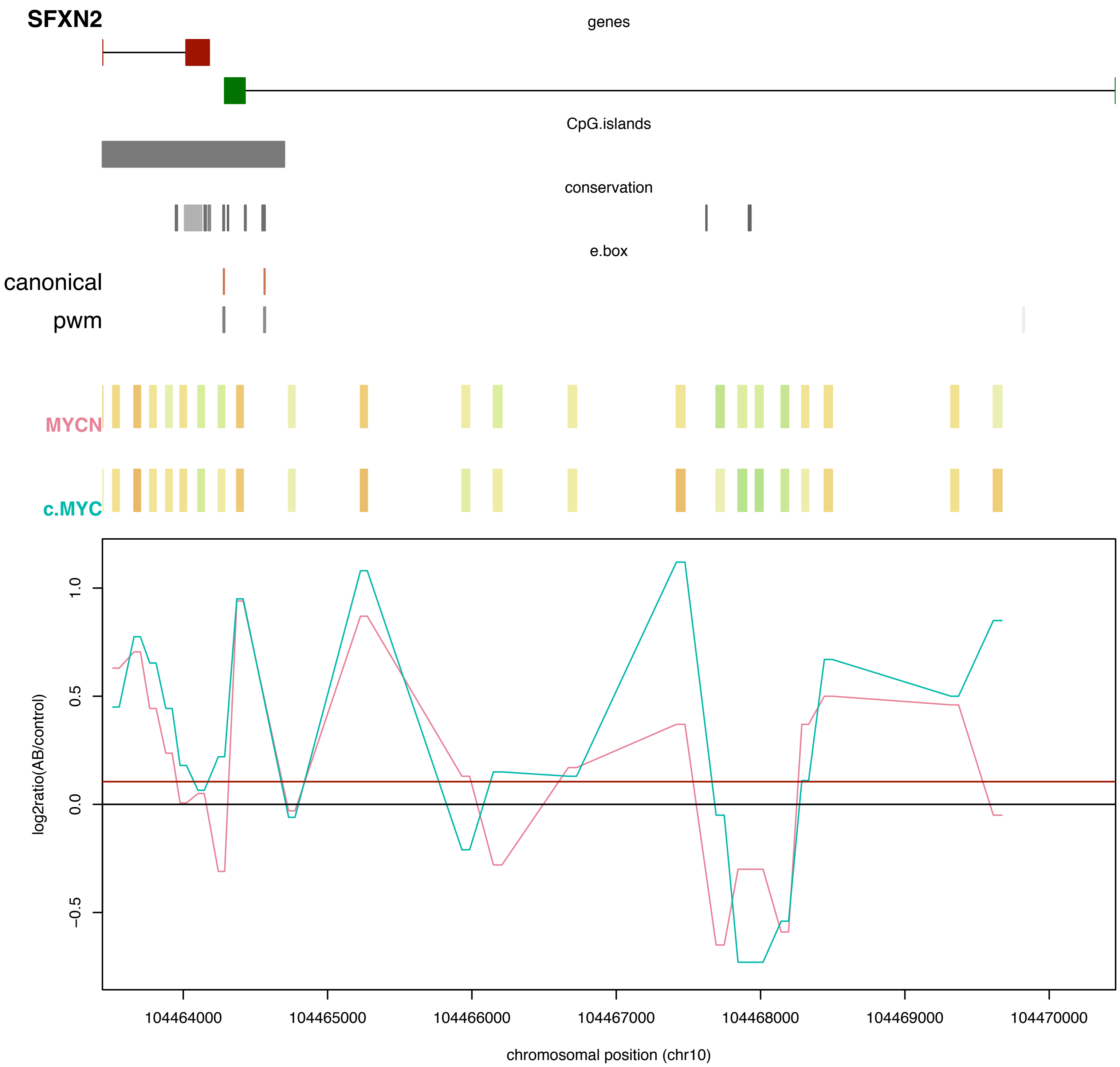

SFXN2
genes
CpG.islands
conservation
e.box
canonical
pwm
MYCN
c.MYC
log2ratio(AB/control)
1.0
0.5
0.0
−0.5
104464000
104465000
104466000
104467000
104468000
104469000
104470000
chromosomal position (chr10)

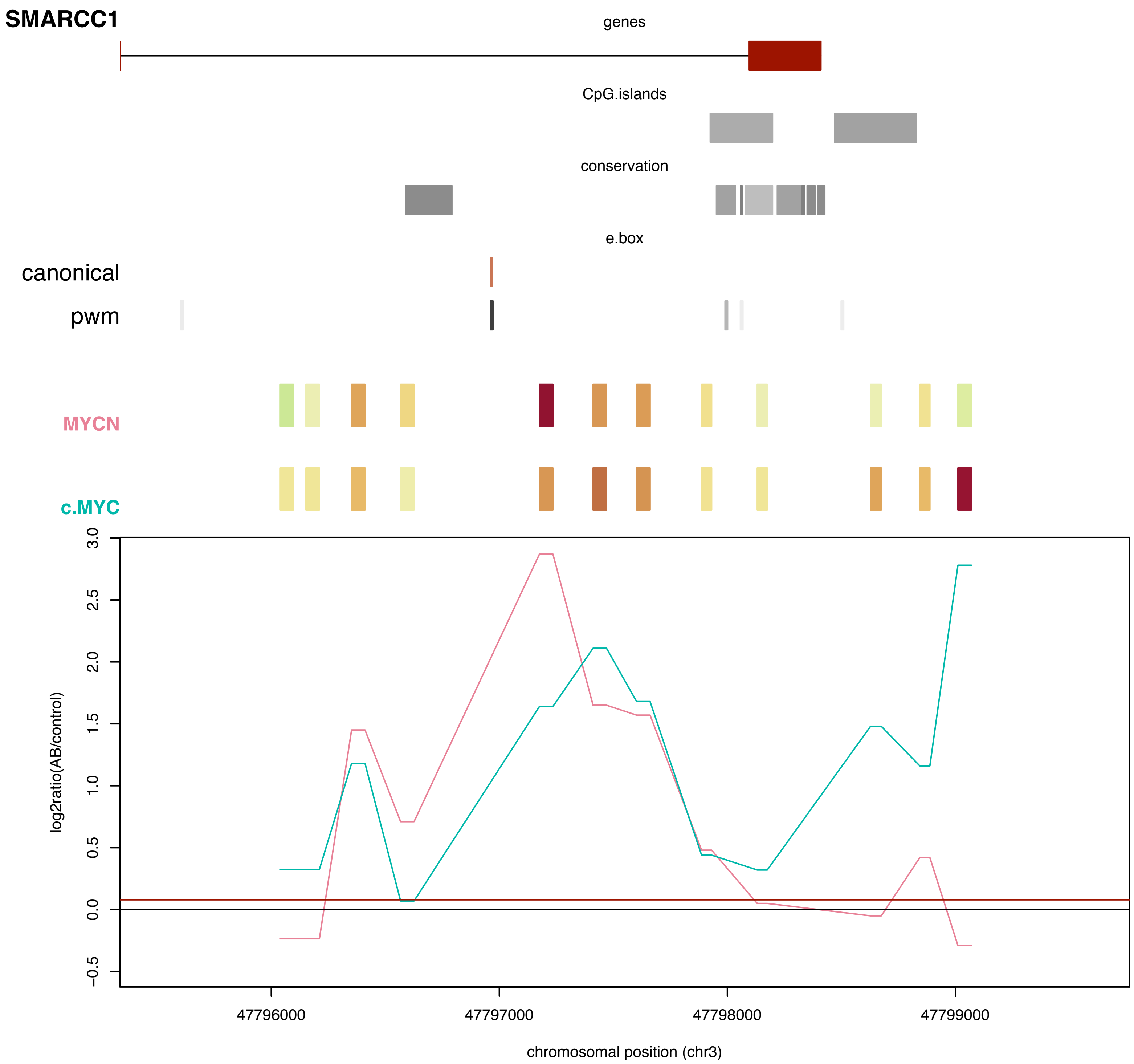

SMARCC1
genes
CpG.islands
conservation
e.box
canonical
pwm
MYCN
c.MYC
log2ratio(AB/control)
3.0
2.5
2.0
1.5
1.0
0.5
0.0
−0.5
47796000
47797000
47798000
47799000
chromosomal position (chr3)

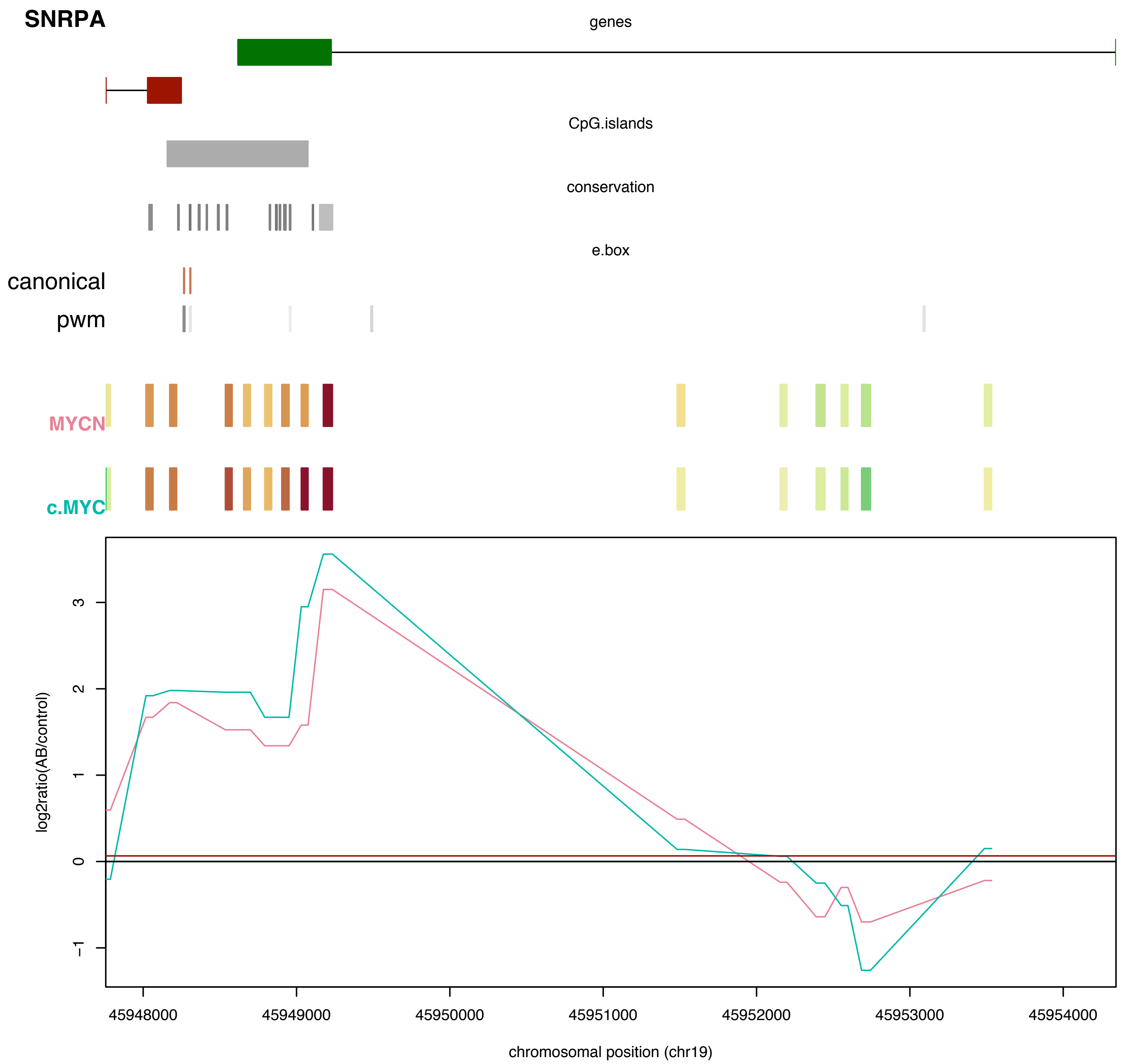
SNRPA
genes
CpG.islands
conservation
e.box
canonical
pwm
MYCN
c.MYC
log2ratio(AB/control)
3
2
1
0
−1
45948000
45949000
45950000
45951000
45952000
45953000
45954000
chromosomal position (chr19)

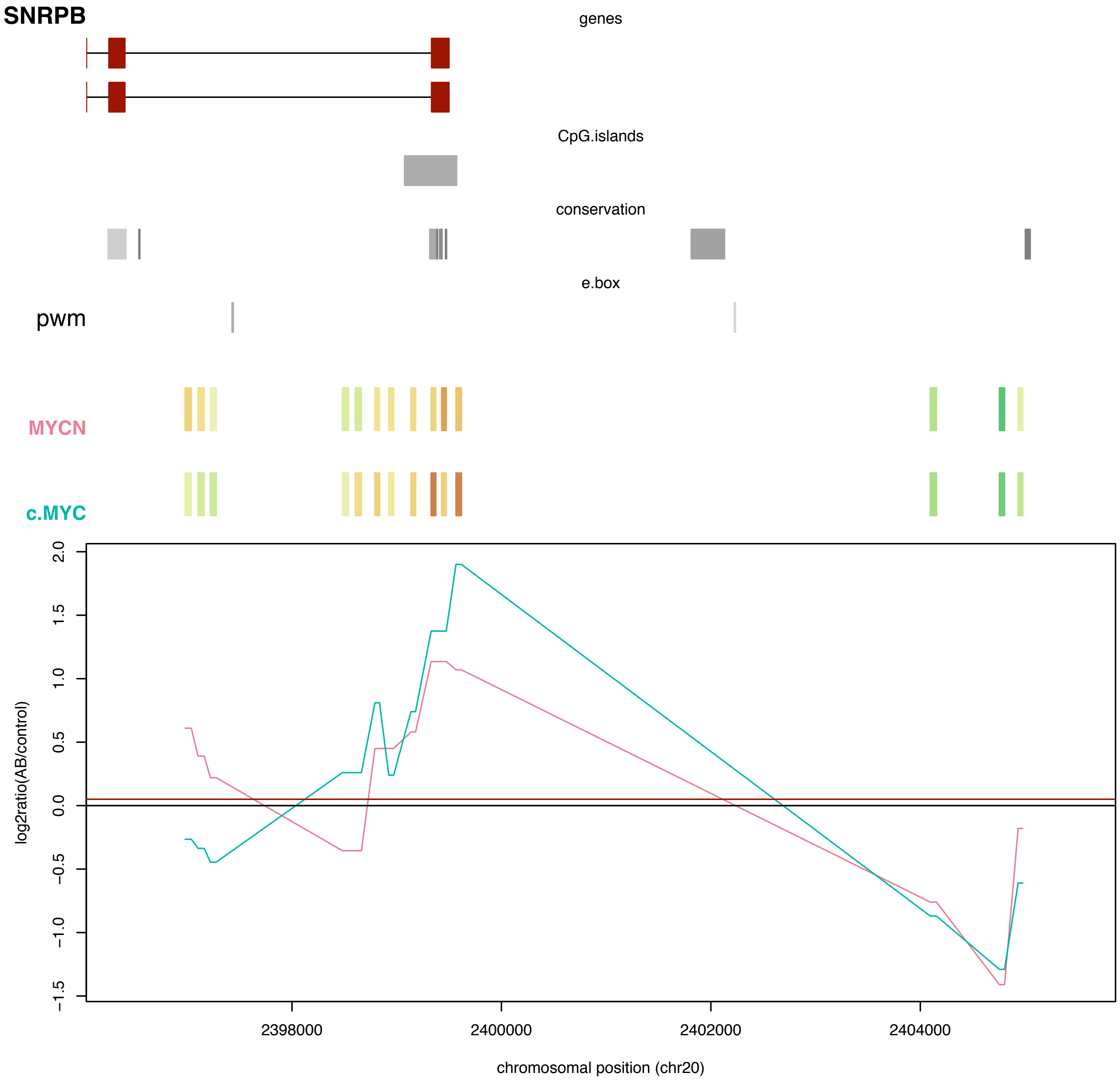

SNRPB
genes
CpG.islands
conservation
e.box
pwm
MYCN
c.MYC
log2ratio(AB/control)
2.0
1.5
1.0
0.5
0.0
−0.5
−1.0
−1.5
2398000
2400000
2402000
2404000
chromosomal position (chr20)

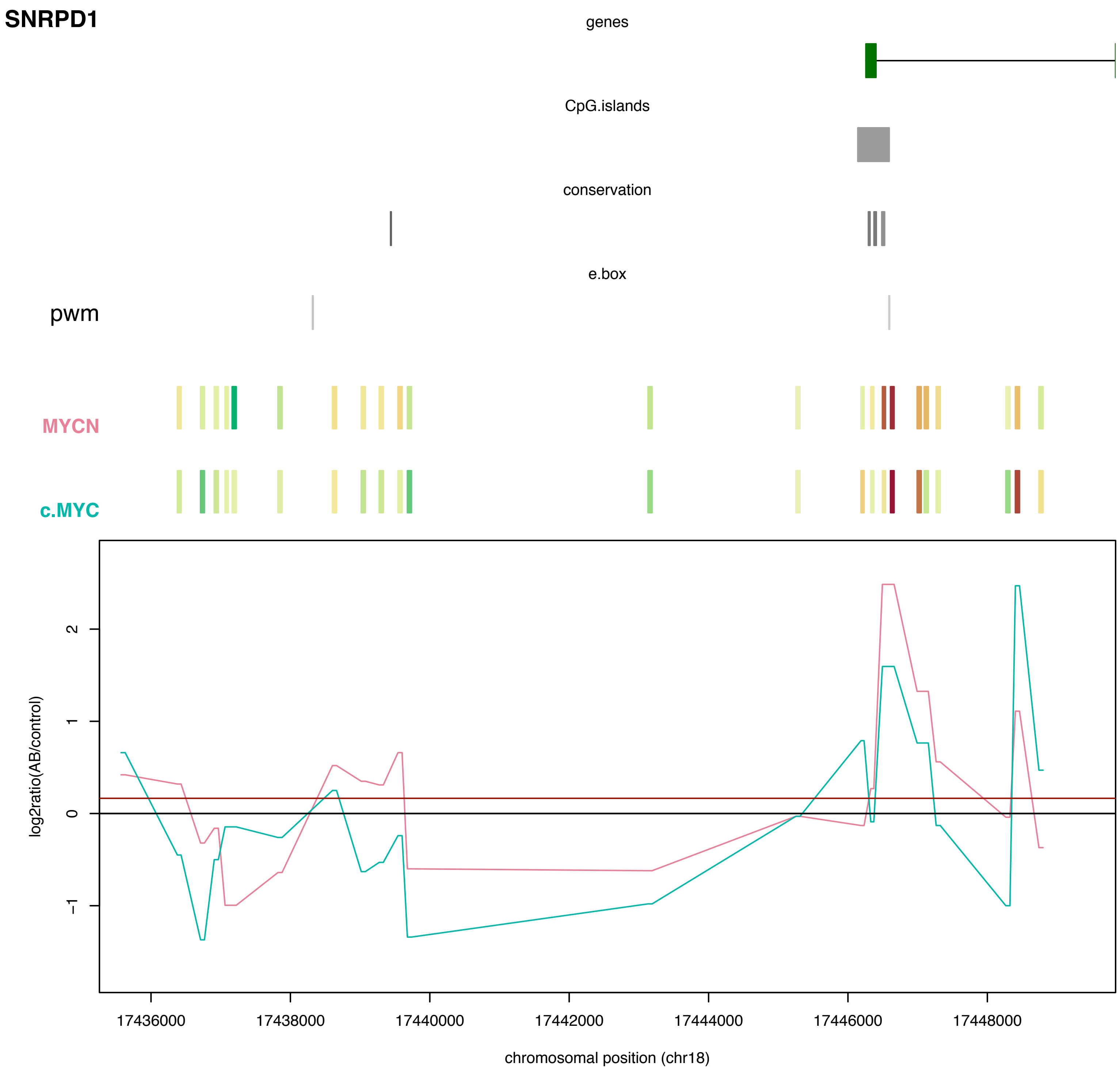

SNRPD1
genes
CpG.islands
conservation
e.box
pwm
MYCN
c.MYC
log2ratio(AB/control)
2
1
0
−1
17436000
17438000
17440000
17442000
17444000
17446000
17448000
chromosomal position (chr18)

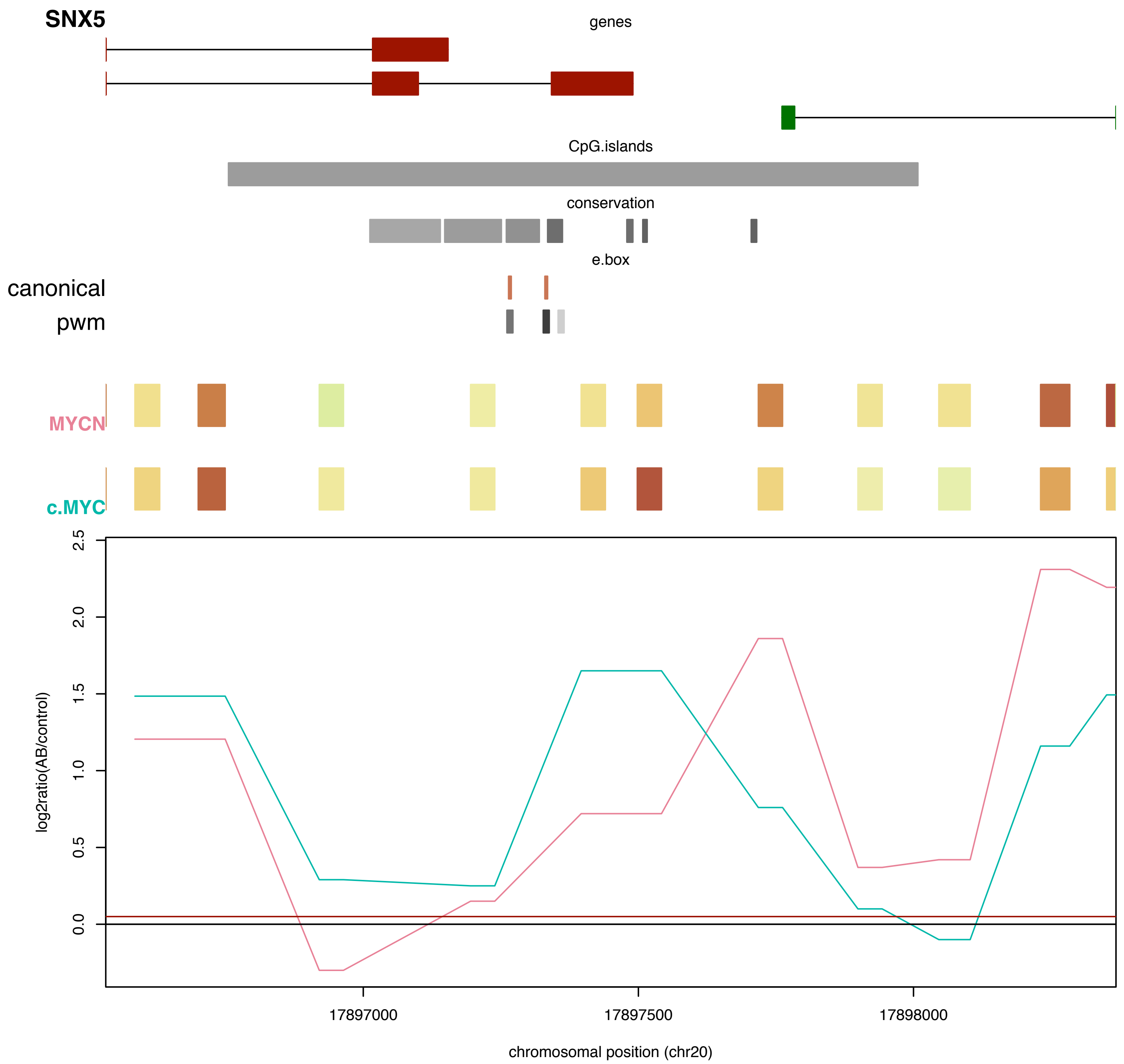

SNX5
genes
CpG.islands
conservation
e.box
canonical
pwm
MYCN
c.MYC
log2ratio(AB/control)
0.0
0.5
1.0
1.5
2.0
2.5
17897000
17897500
17898000
chromosomal position (chr20)

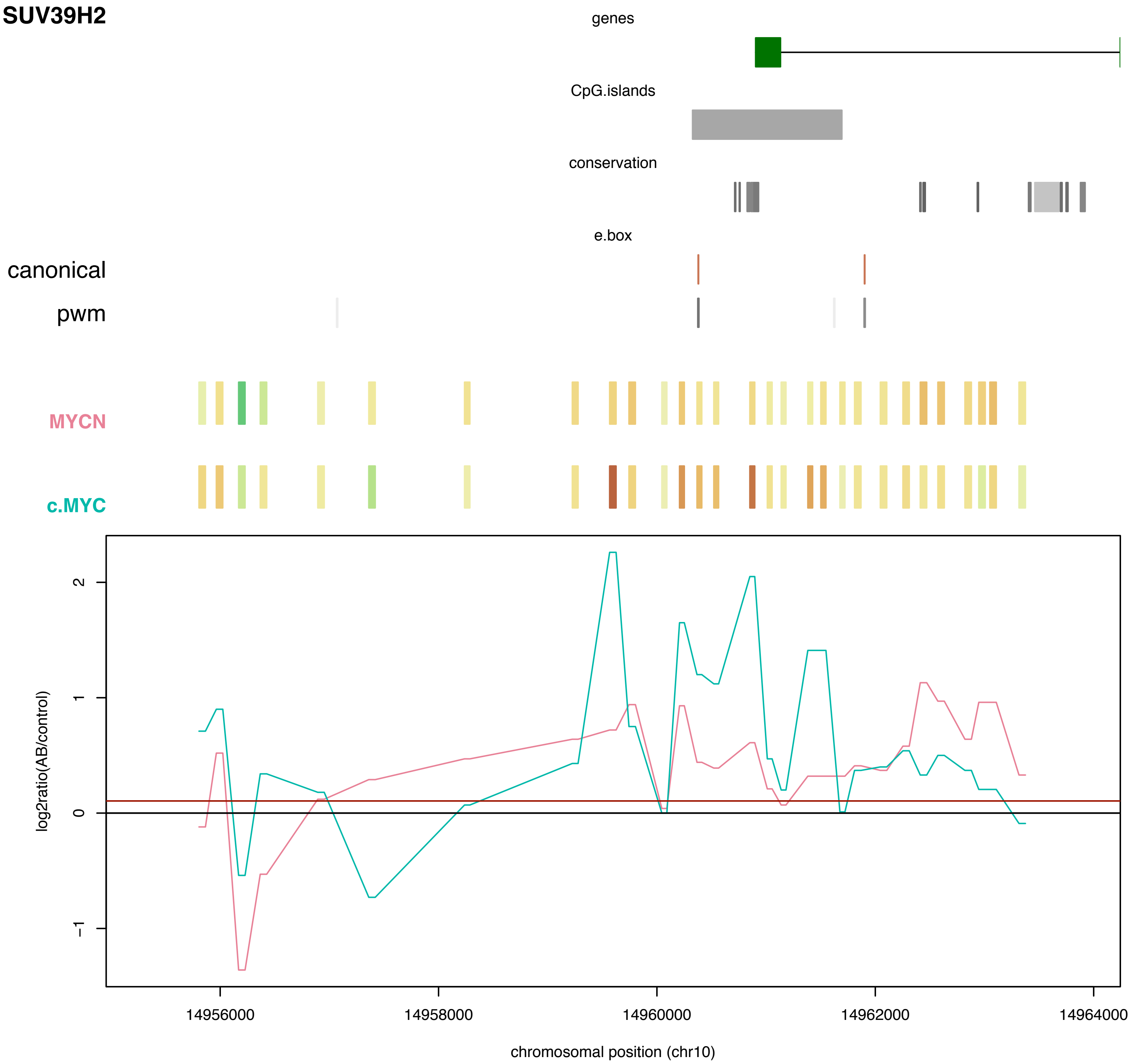

SUV39H2
genes
CpG.islands
conservation
e.box
canonical
pwm
MYCN
c.MYC
log2ratio(AB/control)
2
1
0
−1
14956000
14958000
14960000
14962000
14964000
chromosomal position (chr10)

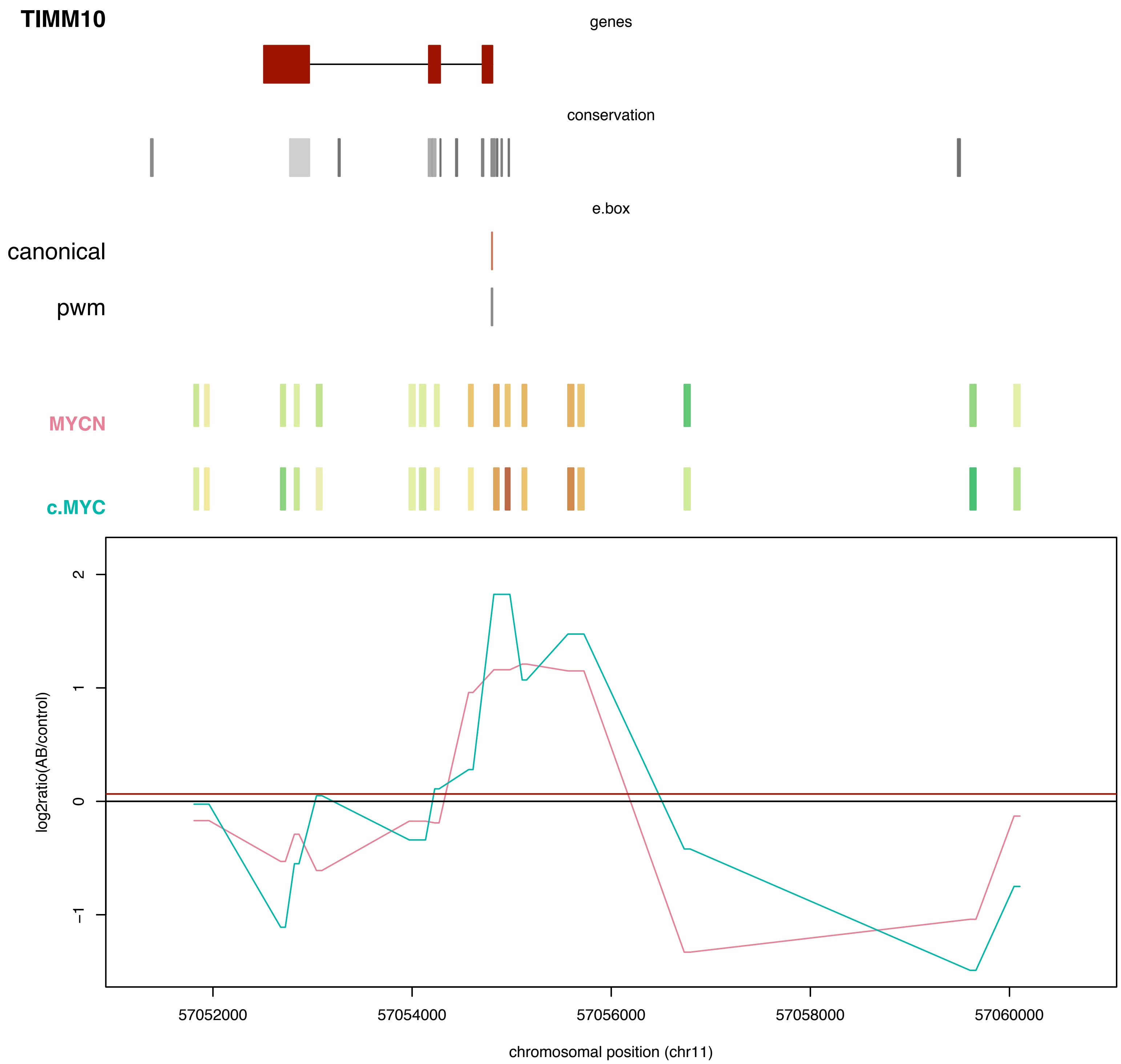
TIMM10
genes
conservation
e.box
canonical
pwm
MYCN
c.MYC
log2ratio(AB/control)
2
1
0
−1
57052000
57054000
57056000
57058000
57060000
chromosomal position (chr11)

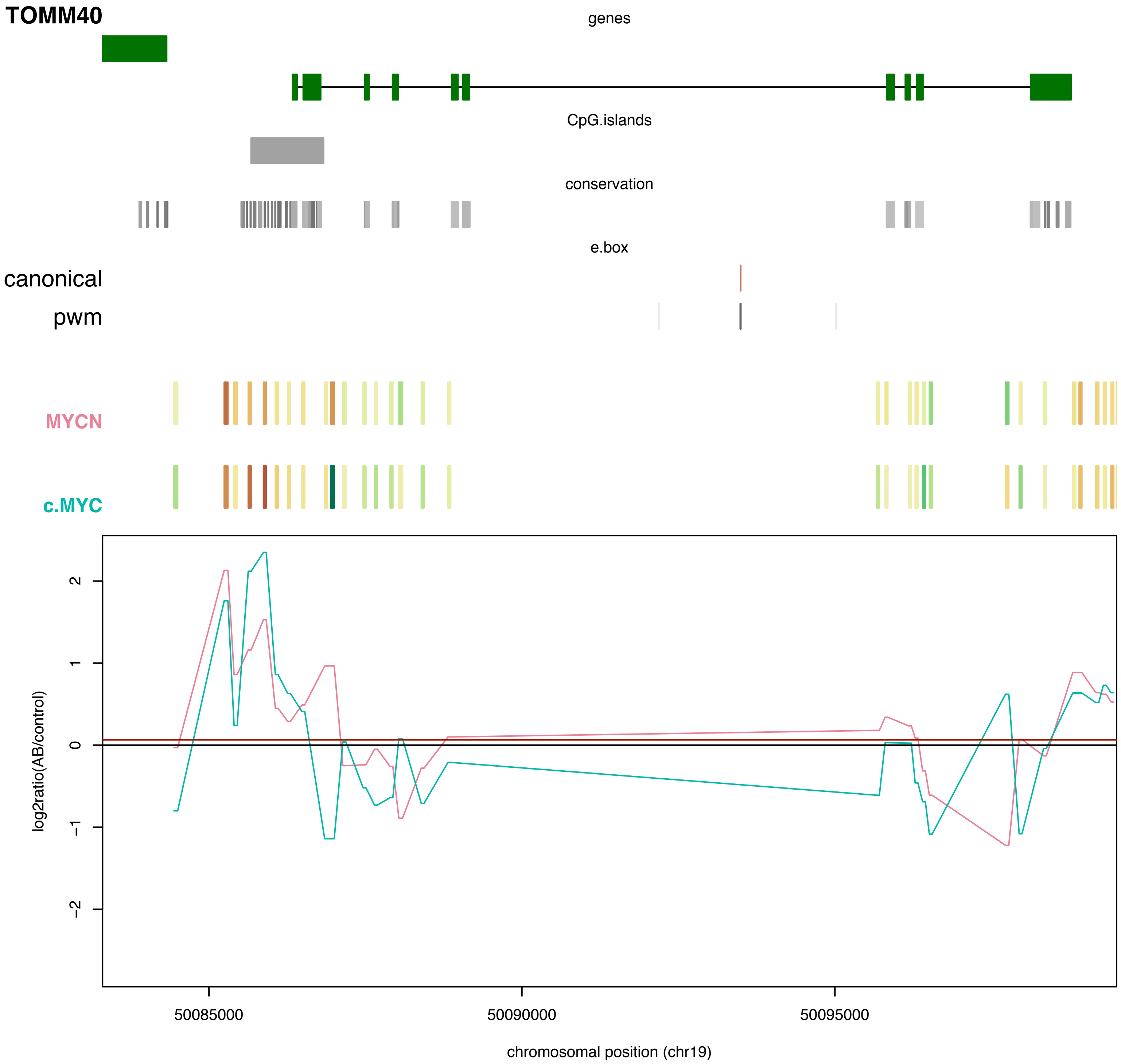

TOMM40
genes
CpG.islands
conservation
e.box
canonical
pwm
MYCN
c.MYC
log2ratio(AB/control)
2
1
0
−1
−2
50085000
50090000
50095000
chromosomal position (chr19)

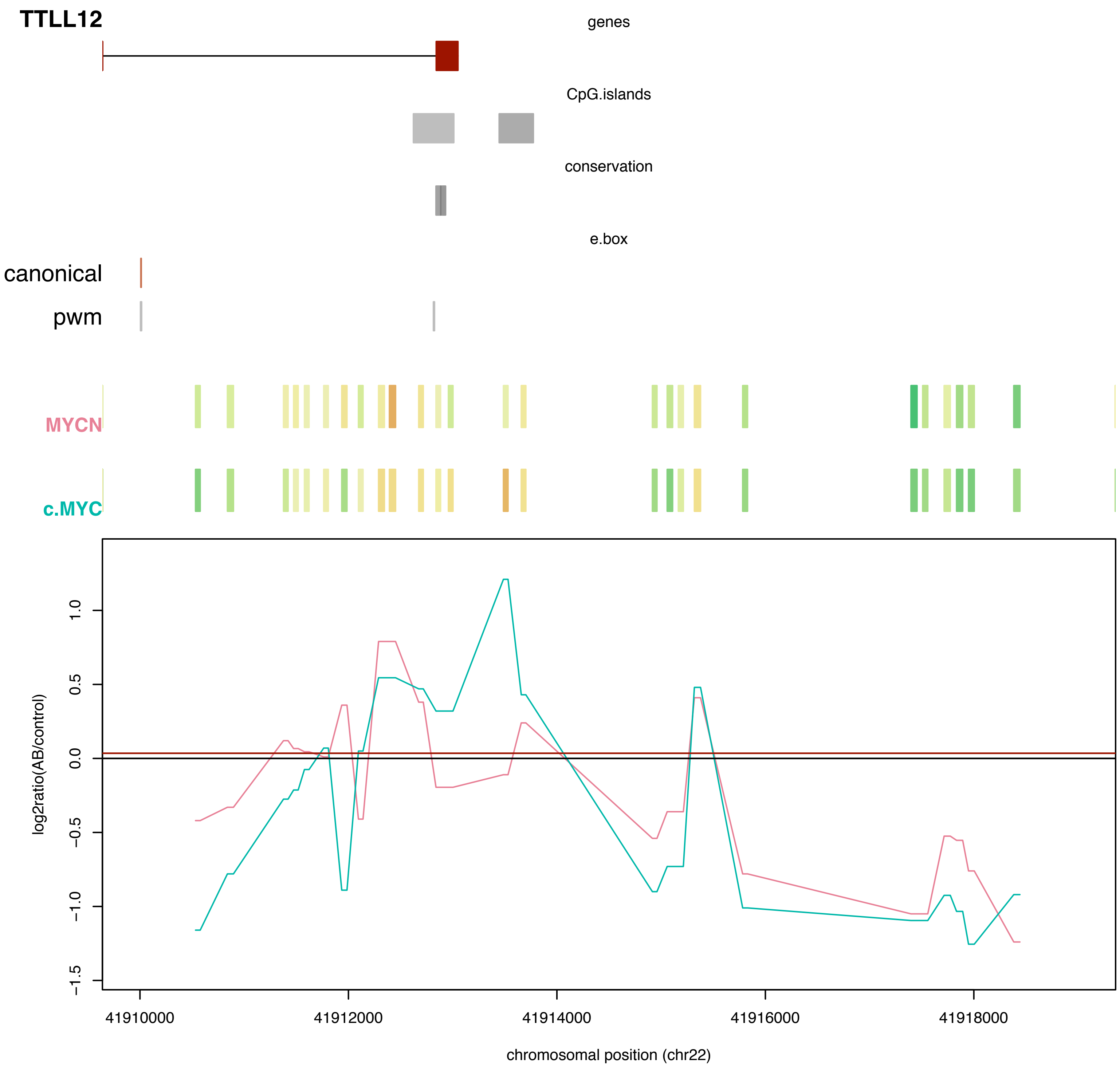

TTLL12
genes
CpG.islands
conservation
e.box
canonical
pwm
MYCN
c.MYC
log2ratio(AB/control)
1.0
0.5
0.0
−0.5
−1.0
−1.5
41910000
41912000
41914000
41916000
41918000
chromosomal position (chr22)

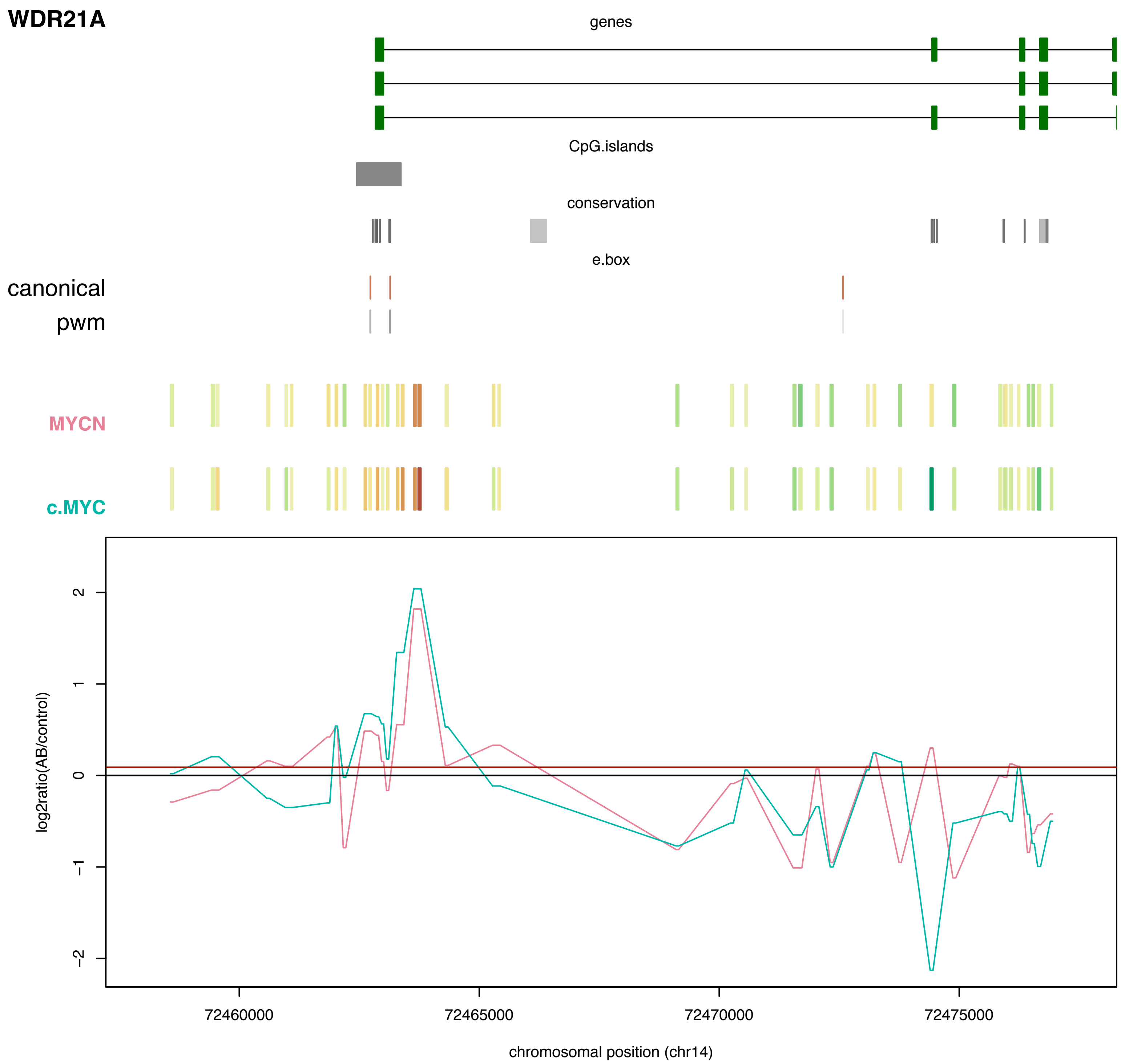
WDR21A
genes
CpG.islands
conservation
e.box
canonical
pwm
MYCN
c.MYC
log2ratio(AB/control)
2
1
0
−1
−2
72460000
72465000
72470000
72475000
chromosomal position (chr14)

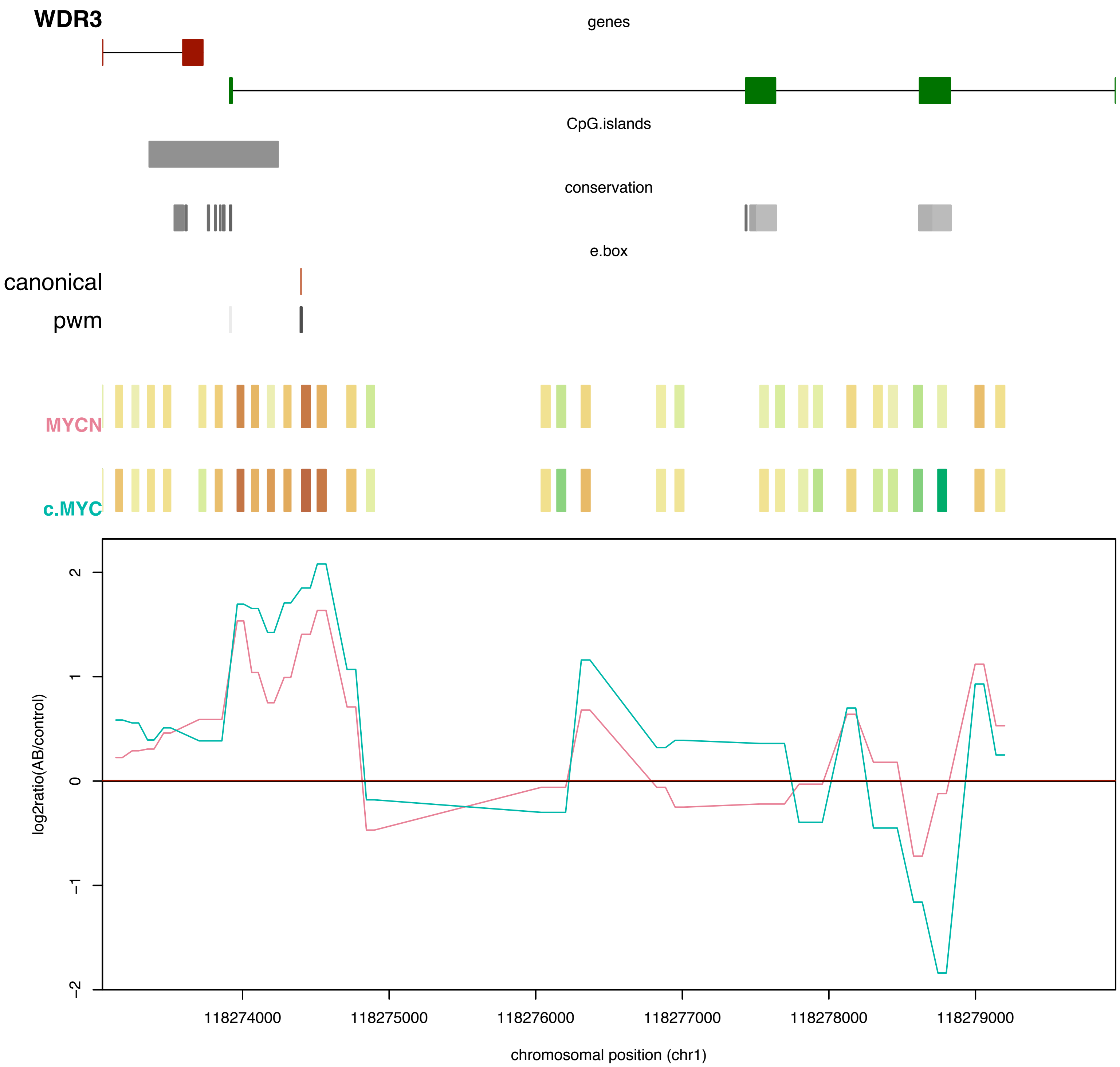

WDR3
genes
CpG.islands
conservation
e.box
canonical
pwm
MYCN
c.MYC
log2ratio(AB/control)
2
1
0
−1
−2
118274000
118275000
118276000
118277000
118278000
118279000
chromosomal position (chr1)

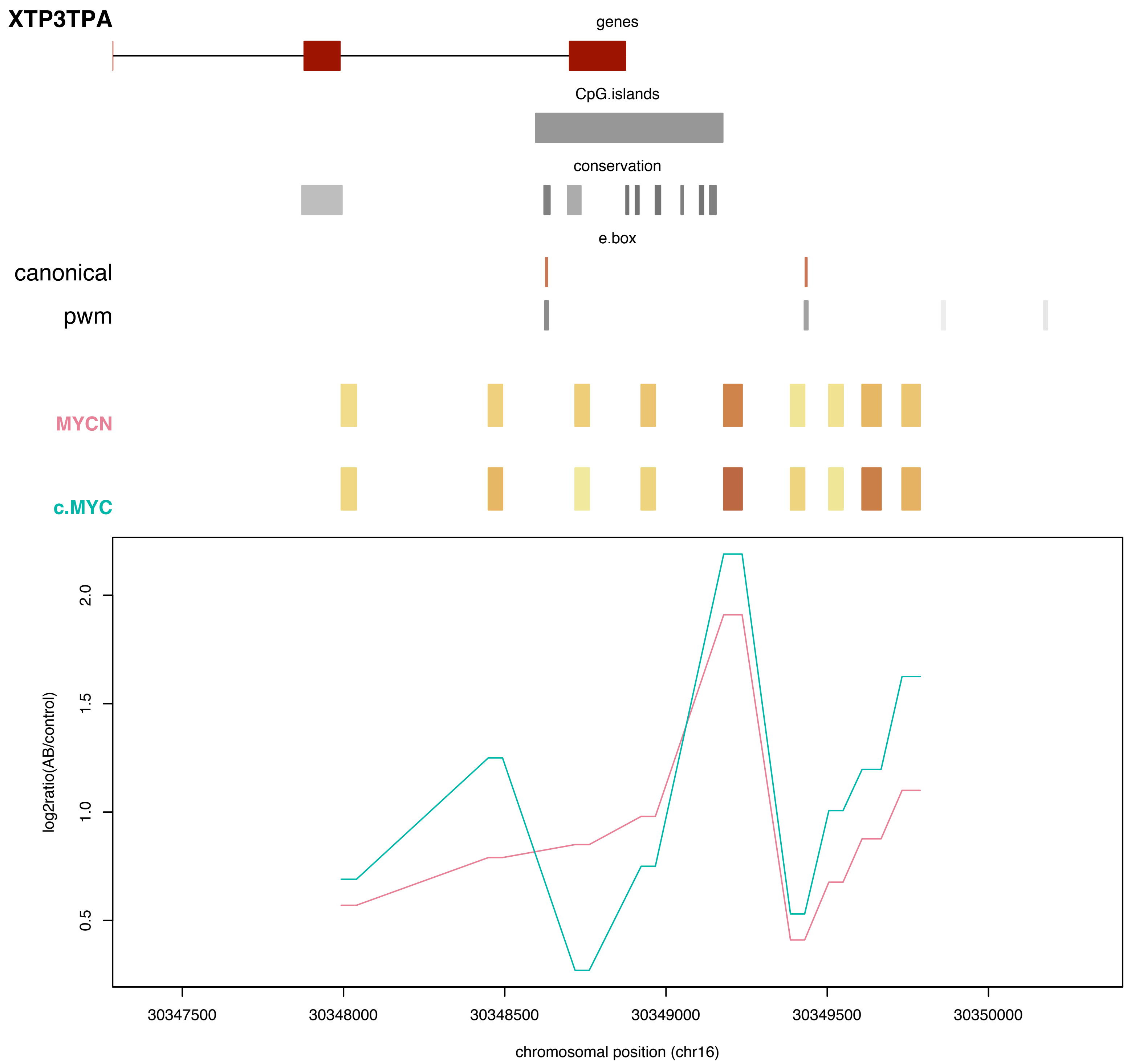
XTP3TPA
genes
CpG.islands
conservation
e.box
canonical
pwm
MYCN
c.MYC
log2ratio(AB/control)
0.5
1.0
1.5
2.0
30347500
30348000
30348500
30349000
30349500
30350000
chromosomal position (chr16)

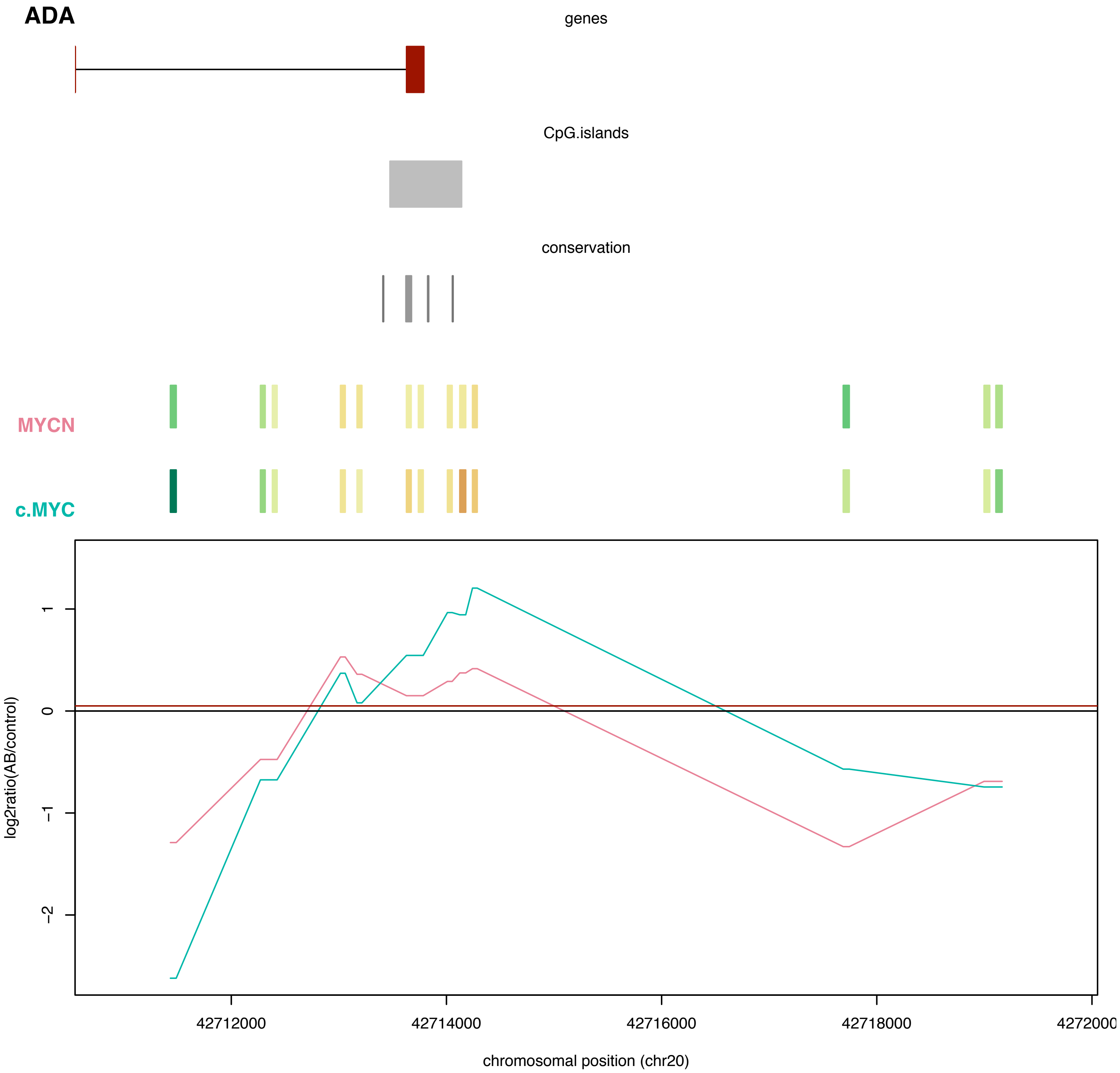

ADA
genes
CpG.islands
conservation
MYCN
c.MYC
log2ratio(AB/control)
1
0
−1
−2
42712000
42714000
42716000
42718000
4272000
chromosomal position (chr20)

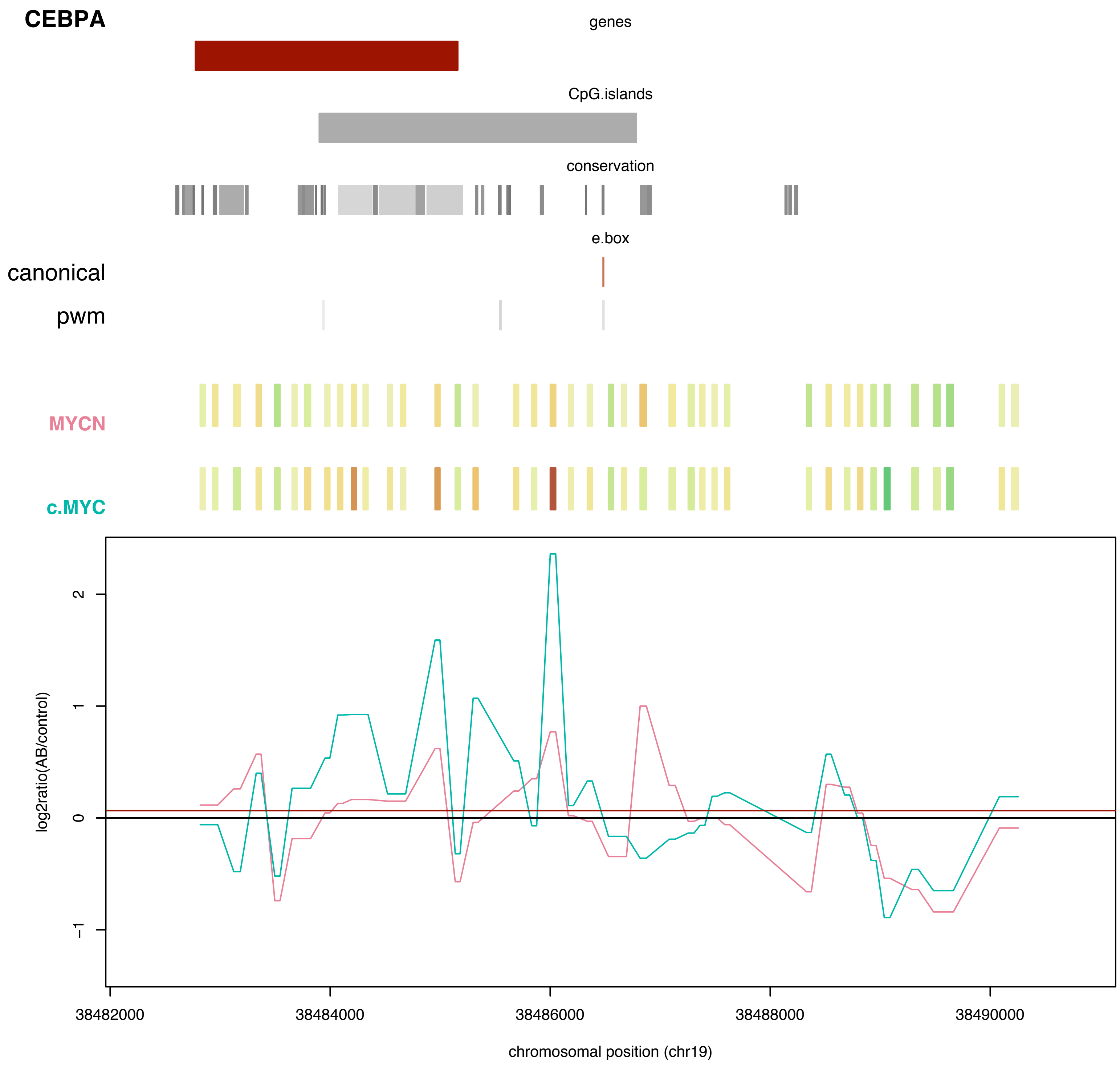

CEBPA
genes
CpG.islands
conservation
e.box
canonical
pwm
MYCN
c.MYC
log2ratio(AB/control)
2
1
0
−1
38482000
38484000
38486000
38488000
38490000
chromosomal position (chr19)

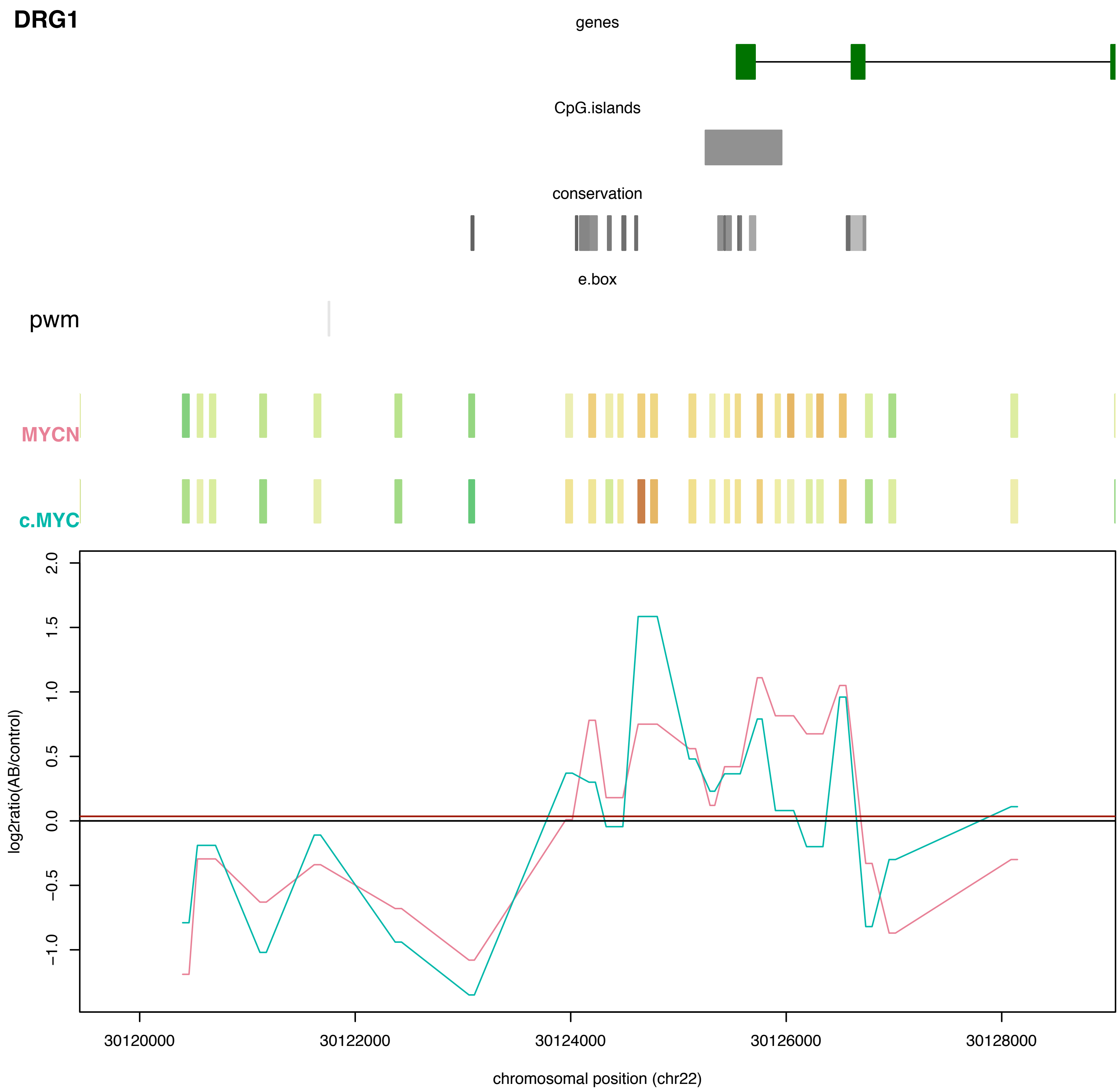

DRG1
genes
CpG.islands
conservation
e.box
pwm
MYCN
c.MYC
log2ratio(AB/control)
2.0
1.5
1.0
0.5
0.0
−0.5
−1.0
30120000
30122000
30124000
30126000
30128000
chromosomal position (chr22)

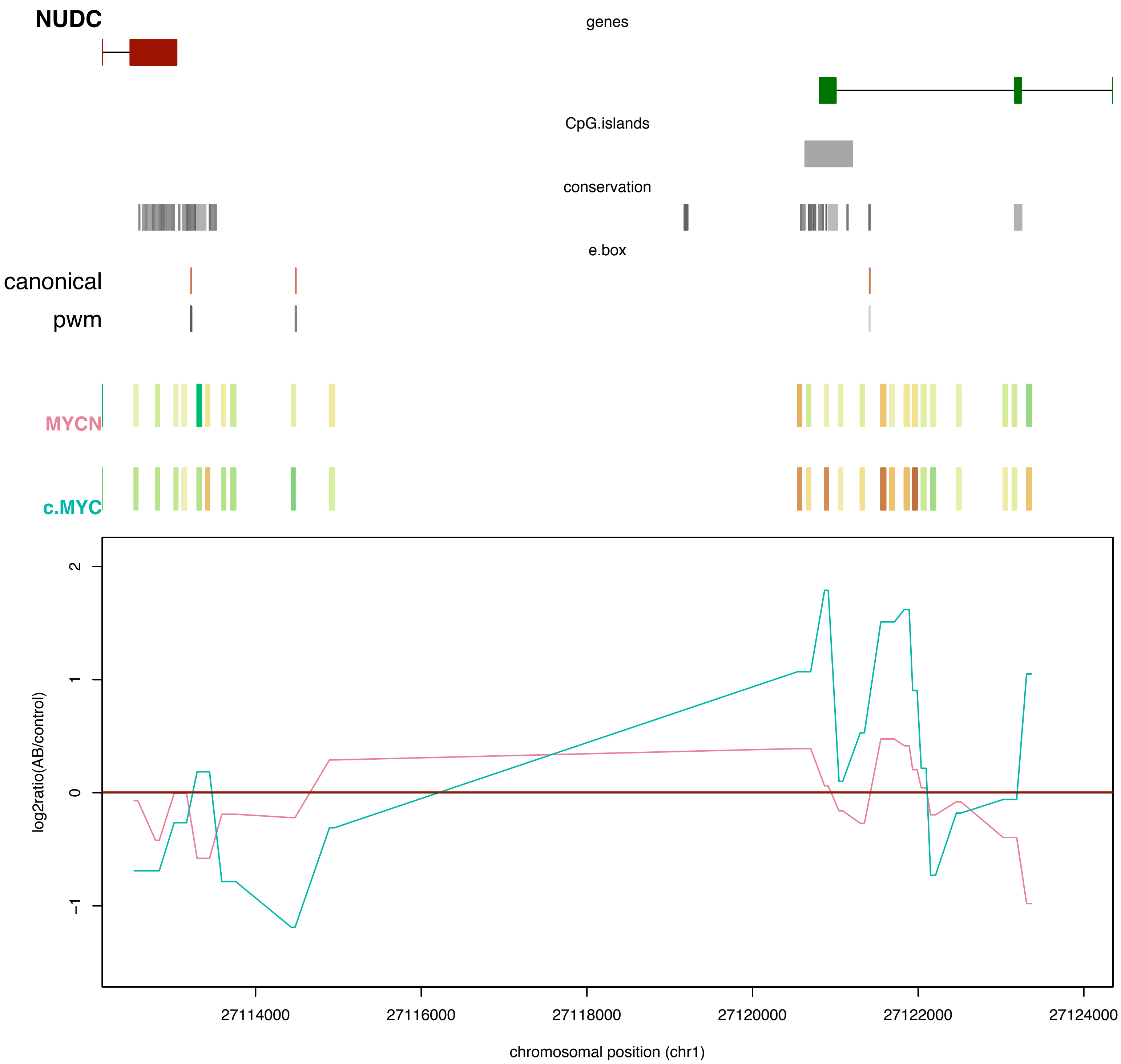

NUDC
genes
CpG.islands
conservation
e.box
canonical
pwm
MYCN
c.MYC
log2ratio(AB/control)
2
1
0
−1
27114000
27116000
27118000
27120000
27122000
27124000
chromosomal position (chr1)

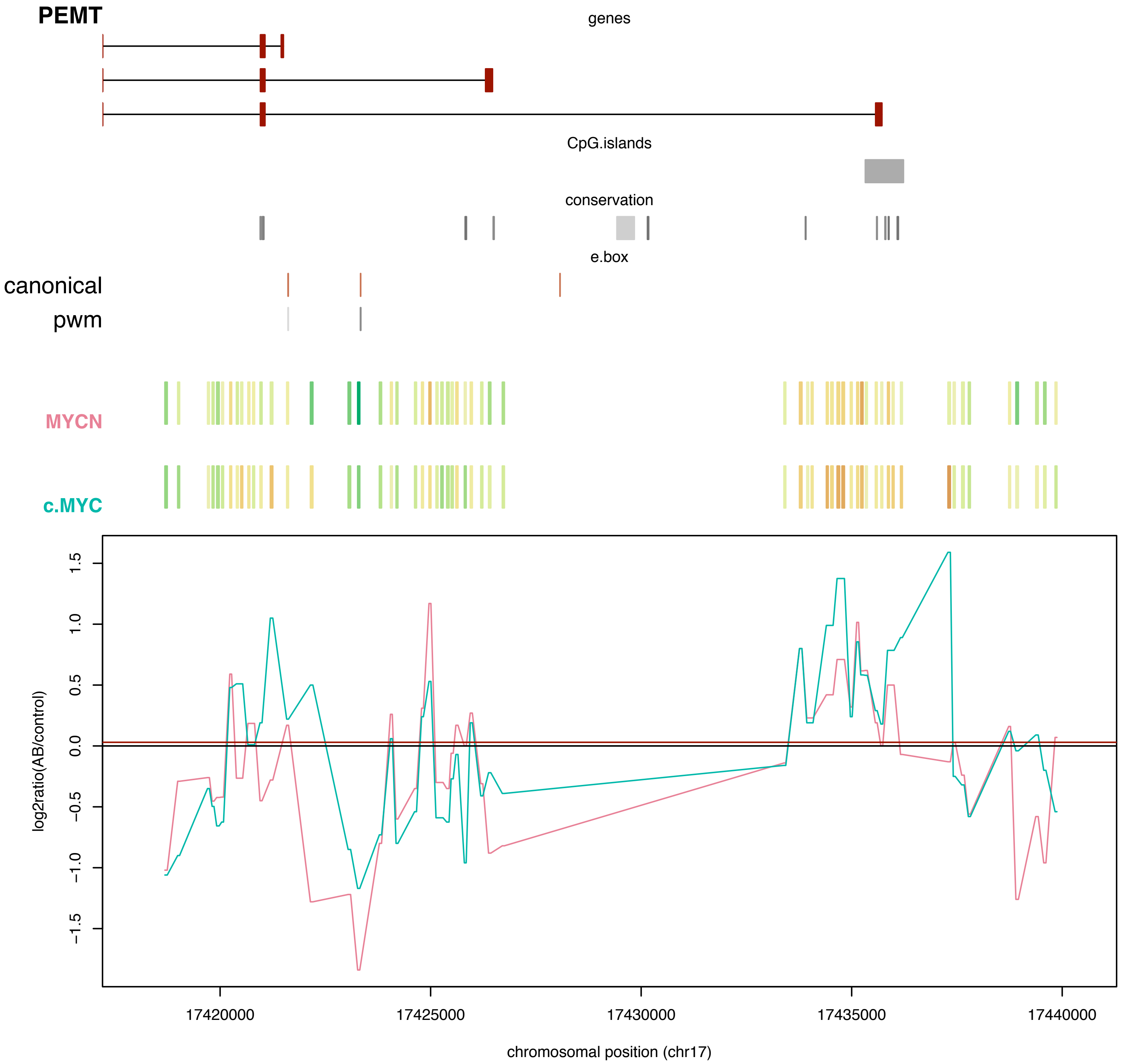
PEMT
genes
CpG.islands
conservation
e.box
canonical
pwm
MYCN
c.MYC
log2ratio(AB/control)
1.5
1.0
0.5
0.0
−0.5
−1.0
−1.5
17420000
17425000
17430000
17435000
17440000
chromosomal position (chr17)

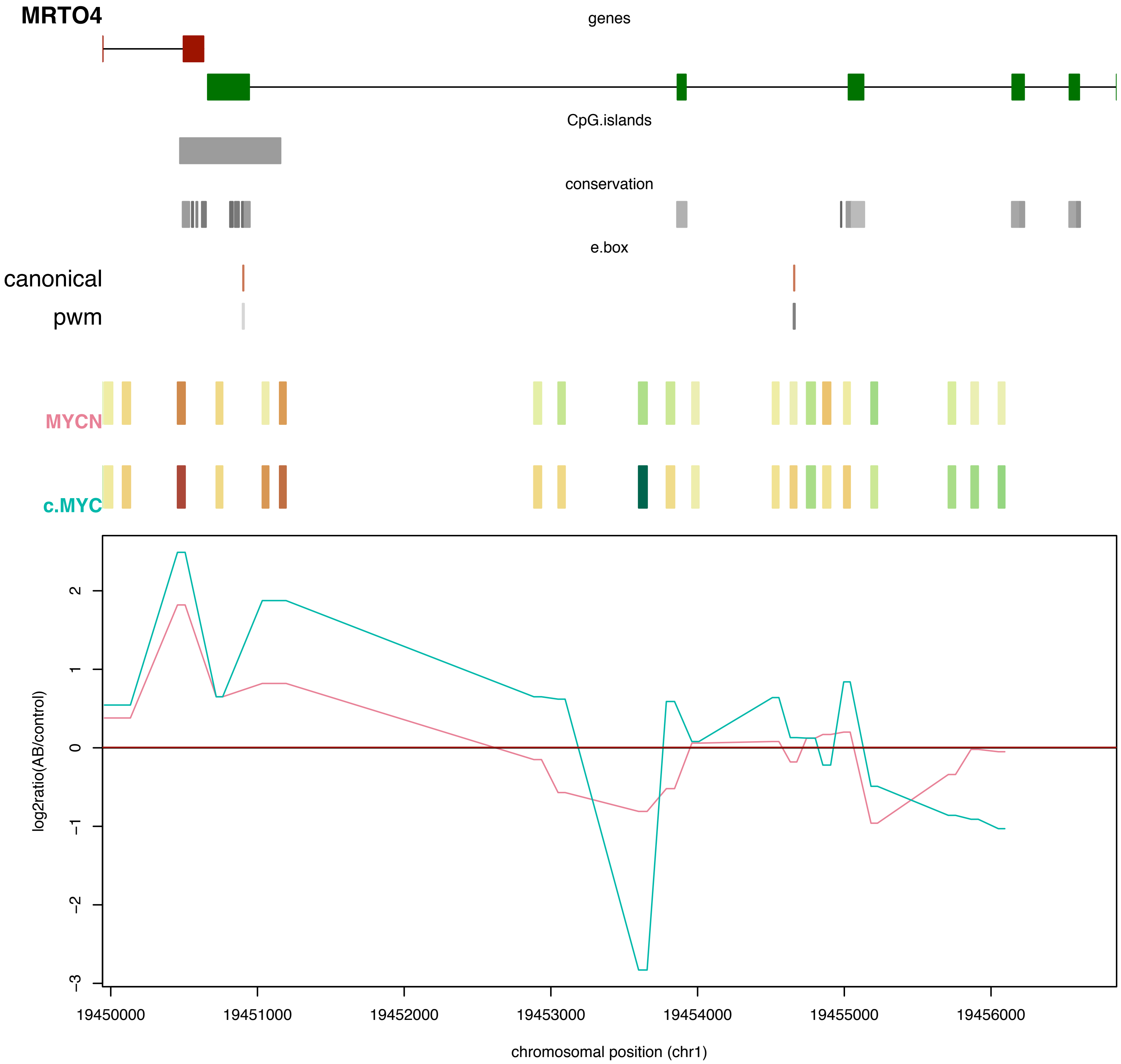
MRTO4
genes
CpG.islands
conservation
e.box
canonical
pwm
MYCN
c.MYC
log2ratio(AB/control)
2
1
0
−1
−2
−3
19450000
19451000
19452000
19453000
19454000
19455000
19456000
chromosomal position (chr1)

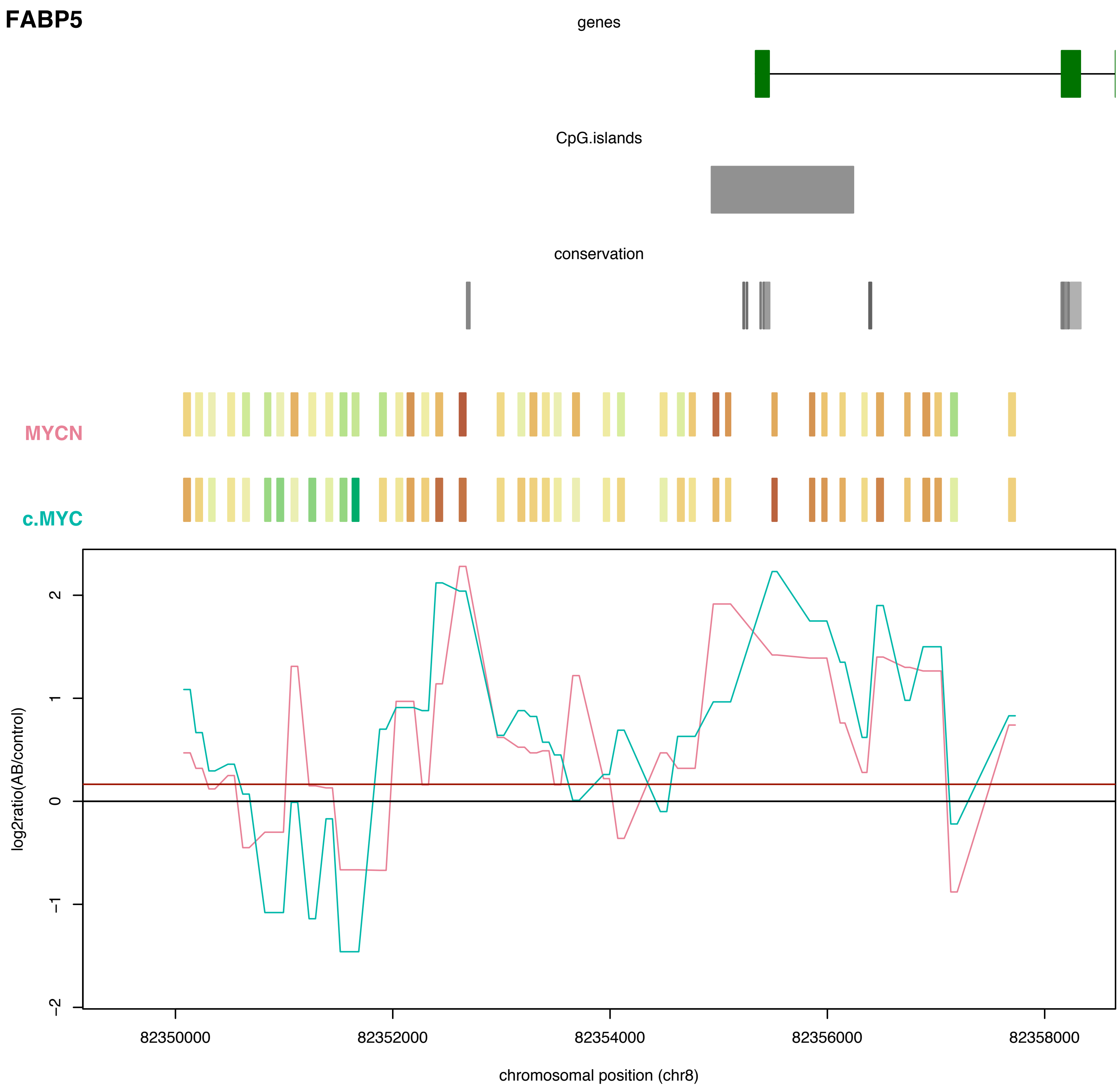

FABP5
genes
CpG.islands
conservation
MYCN
c.MYC
log2ratio(AB/control)
2
1
0
−1
−2
82350000
82352000
82354000
82356000
82358000
chromosomal position (chr8)

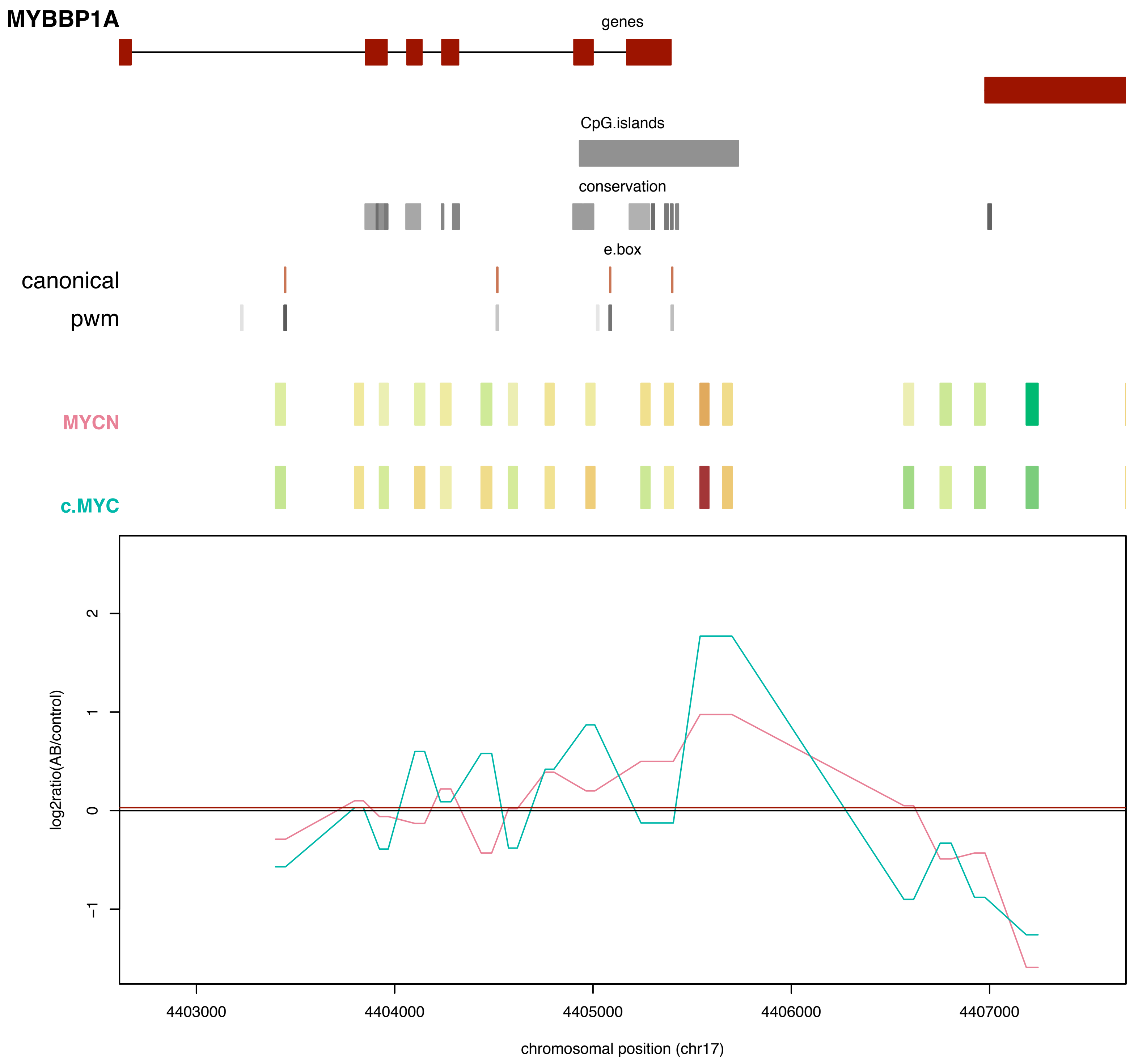

MYBBP1A
genes
CpG.islands
conservation
e.box
canonical
pwm
MYCN
c.MYC
log2ratio(AB/control)
2
1
0
−1
4403000
4404000
4405000
4406000
4407000
chromosomal position (chr17)

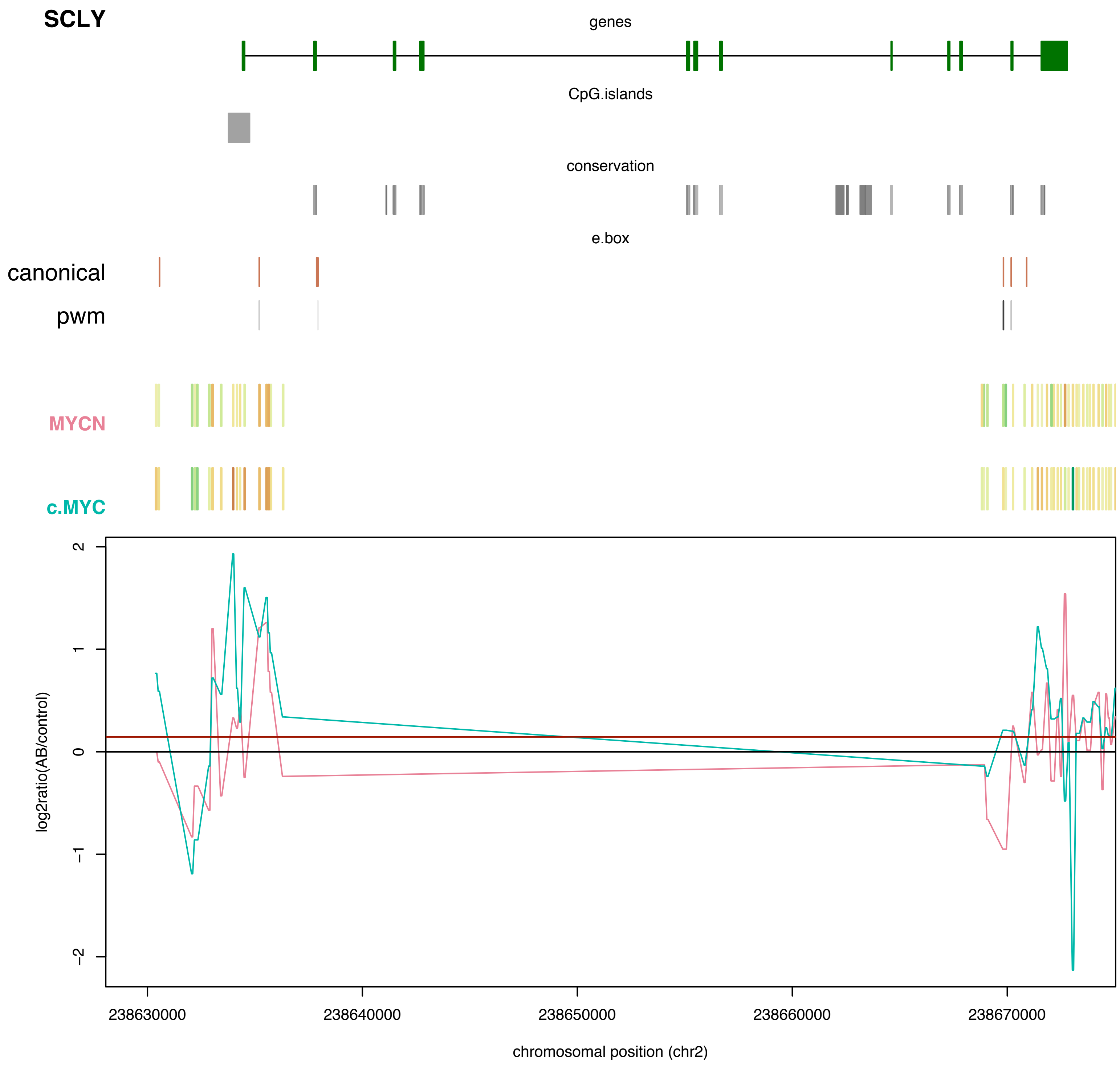

SCLY
genes
CpG.islands
conservation
e.box
canonical
pwm
MYCN
c.MYC
log2ratio(AB/control)
2
1
0
−1
−2
238630000
238640000
238650000
238660000
238670000
chromosomal position (chr2)
